# Early Feasibility Assessment: A Method for Accurately Predicting Biotherapeutic Dosing to Inform Early Drug Discovery Decisions

**DOI:** 10.3389/fphar.2022.864768

**Published:** 2022-06-08

**Authors:** Diana H. Marcantonio, Andrew Matteson, Marc Presler, John M. Burke, David R. Hagen, Fei Hua, Joshua F. Apgar

**Affiliations:** Applied BioMath, LLC, Concord, MA, United States

**Keywords:** QSP, MID3, dose prediction, mAb, biotherapeutic, mechanistic PKPD

## Abstract

The application of model-informed drug discovery and development (MID3) approaches in the early stages of drug discovery can help determine feasibility of drugging a target, prioritize between targets, or define optimal drug properties for a target product profile (TPP). However, applying MID3 in early discovery can be challenging due to the lack of pharmacokinetic (PK) and pharmacodynamic (PD) data at this stage. Early Feasibility Assessment (EFA) is the application of mechanistic PKPD models, built from first principles, and parameterized by data that is readily available early in drug discovery to make effective dose predictions. This manuscript demonstrates the ability of EFA to make accurate predictions of clinical effective doses for nine approved biotherapeutics and outlines the potential of extending this approach to novel therapeutics to impact early drug discovery decisions.

## Introduction

Dosage is fundamental to the success or failure of therapeutic agents (Paracelsus, 1538). The appropriate selection of dose is accordingly a critical component of decision making at all stages of drug development. An earlier understanding of dose, and how drug and target properties influence dose, can greatly improve the speed and quality of drug development. At later stages of development, model-informed drug discovery and development (MID3) approaches have been increasingly used to inform clinical trial dose selection with empirical and mechanistic-based models (Shen et al., 2019). These have been encouraged by the FDA through programs such as the model-informed drug development pilot program (U.S. Food and Drug Administration, 2021). Examples of clinical application include minimum anticipated biological effect (MABEL) dose calculations for first-in-human dose selection (Hu and Hansen 2013; Shen et al., 2019) and rational dose selection for pivotal trials; models are also used to justify simplified dosing for patients and providers, and to extrapolate to untested populations or dosing regimen to inform clinical decision making (Nayak et al., 2018).

Many decisions during discovery and early development can also be impacted by an understanding of the likely clinical dose and the impact of drug properties on the dose. Here, computational methods are essential because these decisions occur long before data from translational or clinical studies are available. For example, if the anticipated clinical dose could be determined to be infeasible to practically administer even assuming ideal drug properties, this could be used as a no-go criteria at the start of a new program and save significant research and development costs (Patel and Bueters 2020). Likewise, target prioritization, clinical candidate selection, preclinical study design, prediction of the impact of formulations or the route of administration can all be informed by earlier understanding of the likely clinical dose (Hu and Hansen 2013; Patel and Bueters 2020). Even in the absence of drug-specific data, understanding the potential for target burden, target turnover, or decoy receptors to impact dose could help prioritize early experiments to fill key knowledge gaps (Hu and Hansen 2013; Glassman and Balthasar 2019; Patel and Bueters 2020). Moreover, progressing efficiently through the new target and lead generation (or biotherapeutics design) stages can impact return on investment, in terms of potentially being first-in-class or best-in-class (Shulze and Ringel 2013). Lalonde et al. (2007) emphasized the importance of starting modeling before compound selection to assist in these types of decisions and continually updating models throughout the development process to aid in decision making at each stage.

Despite the increasing use of such MID3 approaches in later stage programs, the application of these approaches to early stage drug discovery decisions has been more limited. In general, the methods applied in later stage development rely on pharmacokinetic (PK) data, pharmacodynamic (PD) data, or both (PKPD data) that was collected from studies where the drug candidate was administered. This data simply is not available at the early stages of drug discovery. The challenge has been how to develop these models in the absence of PKPD data, relying on what has been previously described in literature, and then validate the results (Hu and Hansen 2013).

The application of mechanistic PKPD models to describe the pharmacology of antibody-based biotherapeutics is an opportunity to overcome these challenges. Antibody-based therapeutics often have predictable linear PK properties (Deng et al., 2011; Dong et al., 2011; Betts et al., 2018), and the impact of binding to soluble and membrane receptor targets on the non-linear PK of antibodies has been well described (Mager and Jusko 2001; Peletier and Gabrielsson 2012; Dua et al., 2015). Mechanistic models can utilize these properties, biological data from the literature on the biophysical properties of the target, and physiological parameters such as compartment volumes, cell numbers, receptor expression levels, and soluble protein concentrations to describe the intended pharmacology of biotherapeutics. Kapitanov et al. provides an example of this application of mechanistic PKPD models, in a series of case studies for antagonist mAbs. In this work, the authors use typical PK and physiological parameters in a “site of action model” to provide insight to guide early discovery decisions (Kapitanov et al., 2021). A generalization of this framework, that is validated with benchmark data, could enable the expanded use of these approaches.

This manuscript presents Early Feasibility Assessment (EFA) as a workflow for the application of mechanistic PKPD models, without fitting to PK or PD data, to predict effective dose for biotherapeutics. The process of model selection, model parameterization, and criteria definition for dose prediction are described through specific case studies. EFA is used to predict the clinical efficacious doses of nine approved biotherapeutics across a range of targets and indications. These examples demonstrate the capabilities of EFA to make relevant predictions and establish a workflow that can be applied at an early stage, even before the generation of candidate or tool molecules (Applied BioMath 2021).

## Materials and Methods

### Test Set of Drugs, Targets and Indication

A representative collection of 9 clinically approved biotherapeutics were modeled in this analysis. Drug targets include both soluble (TNFα, IL-23/IL-12, IL-23, BLyS, IgE) and membrane (HER2, EGFR, c-Met) targets. These biotherapeutics have been approved in a range of oncology and immune and inflammation (I&I) indications. The complete list of drugs, targets, and indications are provided in Table 1.

**TABLE 1:** Biotherapeutics included in EFA analysis.

Drug	Indication^a^	Target
Remicade (infliximab)	RA	TNFa
Humira (adalimumab)	RA	TNFa
Stelara (ustekinumab)	Plaque psoriasis	IL-23/IL-12
Skyrizi (risankizumab)	Plaque psoriasis	IL-23
Benlysta (belimumab)	SLE	BLyS (BAFF)
Xolair (omalizumab)	Asthma	IgE
Herceptin (trastuzumab)	Breast Cancer	HER2
Vectibix (panitumumab)	Colon Cancer	EGFR
Rybrevant (amivantamab)	NSCLC (EGFR exon 2)	EGFR/c-Met

a RA = rheumatoid arthritis, SLE = systemic lupus erythematosus.

### Model Strategy

Three different mechanistic PKPD models were used for the analyses in this manuscript. Full model descriptions are included later in the manuscript. All models are *in vivo* human models which describe drug administration, PK, target binding, and target dynamics in one or more compartments. The models were used to predict PK, target engagement, and target inhibition at different doses. Target engagement or inhibition criteria were used to define effective dose. Models were chosen according to each biotherapeutic’s pharmacology.

For soluble targets, a 1-compartment monospecific anti-ligand model was chosen. Drug interactions with soluble targets are confined to the vascular and interstitial fluid spaces, and can be sufficiently described with a one-compartment model. While one-compartment models do not accurately describe the distribution phase of typical mAb PK, the analysis focuses on inhibition at trough concentrations, which can be captured by a one-compartment model.

For membrane targets, a 2-compartment monospecific anti-receptor model was chosen. Unlike soluble targets, membrane targets are often preferentially expressed in the peripheral tissues. Antibody distribution into peripheral tissues can also be limited (Shah and Betts 2012). Physiologically-relevant representation of drug distribution into the lumped peripheral compartment, target expression in the peripheral compartment, and drug interactions with target were considered necessary to describe the drug pharmacology. While not used here, for targets with high or low tissue penetration, a three (or more) compartment model can be used with tissue specific antibody biodistribution coefficient to describe the transport (Shah and Betts 2013).

For the bispecific antibody case study, a 2-compartment bispecific anti-receptor x anti-receptor model was chosen. A 2-compartment model was chosen for more accurate representation of the membrane targets.

All models were parameterized using data obtained from literature (i.e. there is no parameter fitting). The only compound specific data was of the type typically available in early discovery (e.g. affinity, valency, etc.). Typical values, or expected ranges for these parameters can be used to apply this analysis even earlier. Detailed methods for model parameterization are described below. For each drug, a criterion for defining effective dose (e.g. 90% sustained target inhibition) was chosen. Models were then simulated to determine the dose required to achieve the criterion. This model predicted effective dose was compared to clinically approved doses for each drug. For model validation, it was assumed that dose predictions within 3-fold of the prescribed efficacious dose of drugs was sufficient for early decision making, especially for the prioritization of potential targets and to inform lead identification and optimization criteria.

### Model Structure

**FIGURE 1:**
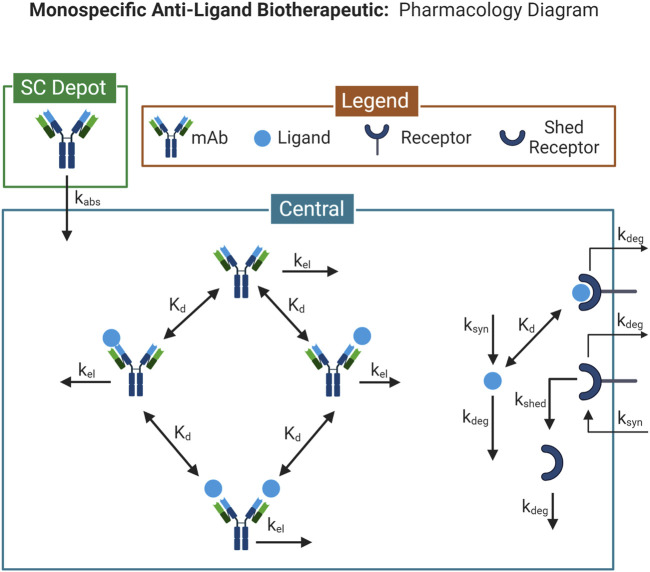
Model Diagram for the 1-compartment monospecific anti-ligand model. Diagram illustrates the species and reactions comprising the pharmacological model describing the interaction between a monospecific anti-ligand antibody and its target. Model diagram was created with BioRender.com.

The 1-compartment monospecific anti-ligand model is a single-compartment model describing drug administration, target-binding, and elimination (Figure 1). Drug administration can be described as an intravenous (IV) bolus, or subcutaneous (SC) administration with a 1st order absorption rate. Target ligand and its cognate receptor are synthesized in the compartment with a 0th order rate. Ligand binds reversibly to the receptor, specified by a monovalent equilibrium dissociation constant (Kd). Drug binds reversibly to the target ligand, specified by a separately parameterized Kd, and blocks ligand-receptor interactions. All species are eliminated through 1st order processes. This model is run using the Monospecific Anti-Ligand model in Applied BioMath Assess ™.

The 2-compartment monospecific anti-receptor model consists of a central and peripheral compartment. (Supplementary Figure S1) Drug is administered into the central compartment as an IV bolus. Target membrane receptor is synthesized through 0th order processes in both central and peripheral compartments. A soluble form of the receptor is generated through shedding from the membrane receptor by a 1st order process. Drug can reversibly bind either membrane or soluble forms of the receptor, specified by a binding Kd. Bivalent binding of drug to membrane or soluble forms of the receptor are modeled as independent binding reactions with identical Kd values. All species are eliminated with 1st order rates in both compartments. All binding interactions occur in each compartment with identical Kd values. All soluble species can transport between compartments with 1st order rates. This model is run using the Monospecific Anti-Receptor (4 compartment) model in Applied BioMath Assess ™, with the tox and disease compartments disabled.

The 2-compartment bispecific anti-receptor x anti-receptor model consists of a central and peripheral compartment. (Supplementary Figure S2) Model reactions are constructed as in the monospecific anti-receptor model, except with 2 different target receptors. Free drug can bind with either receptor, specified by independent binding Kd’s. Reversible binding of a second receptor is described as an independent binding process, parameterized by the same receptor-specific Kd. This model is run using the Bispecific Anti-Receptor x Anti-Receptor (4 compartment) model in Applied BioMath Assess ™, with the tox and disease compartments disabled.

### Model Parameterization

Drug-specific parameters, defined as elimination half-life, target binding Kd, valency, and molecular weight, were identified from reported values. Target binding affinities were identified from *in vitro* measurements in biochemical or cell-based assays. Half-life was identified from reported PK data.

Target specific parameters, defined as target concentration and target turnover rate were calculated from literature measurements. Soluble target concentrations were parameterized by plasma measurements in indication-specific patients. Soluble target half-life may be measured from pharmacokinetic measurements of exogenously administered target. Membrane target concentrations were calculated using “bottom-up” methods. Target expression was calculated as the sum of the number of cells for each cell type expressing the target x % of each cell type expressing the target x receptors per cell. Target expression was divided by the interstitial volume of each relevant compartment to determine target concentrations. Examples of data supporting inputs include, but are not limited to, immunohistochemical staining of target across tissues, quantitative or semi-quantitative flow cytometry, Scatchard analysis of ligand binding sites, functional data on target activation or knock-down, RNA expression data. Membrane target turnover rates were identified from *in vitro* cell line measurements when available. When data is not available, assumptions based on other proteins of the same family, similar structure, molecular weight, or function were used.

### Model Assumptions

For all models, compartments are assumed to be well-mixed. Non-specific elimination of the drug occurs in all compartments with equal 1st order rate constant. For the anti-ligand model, drug: target-ligand complex is assumed to eliminate at the same rate as the free drug. Ligand:receptor complex is assumed to eliminate at the same rate as free receptor. For the anti-receptor models, internalization and elimination of the membrane receptors are considered a single process. Drug:membrane receptor complexes eliminate at the same rate as free receptors. Drug:soluble-receptor complexes eliminate at the same rate as free drug. For multi-compartment models, all soluble species transport bi-directionally between compartments. Drug:soluble-receptor complexes are assumed to transport with the same rate constant as free drug. All multivalent binding interactions are assumed to be identical and independent.

### Model Software

All simulations were performed using Applied BioMath Assess ™ version 2021.12.1 (https://www.appliedbiomath.com/assess). Run files in json format, Model files, and Assess Model Reports are included in Supplementary Material.

## Results

To assess the ability of the EFA methodology to accurately translate mechanistic parameters into likely clinical doses, we performed a set of simulation studies for nine approved biotherapeutics. Because these drugs have been approved there is data on the molecular properties (e.g. affinity Kd and half-life) as well as the approved clinical dose. Obviously this data is not typically available for an early stage program. Where EFA is used in practice these parameters would be set to a typical value for the modality, or scanned over a typical range to find the critical value where the pharmacology requirements are satisfied. However, here we are looking at the ability of the model to accurately translate the mechanistic parameters to predict a likely clinical dose. To assess this, we are looking at the degree of agreement (or disagreement) of the effective dose predicted by EFA compared to the approved clinical dose.

### Case Study 1: Effective Dose Prediction for Adalimumab and Infliximab, Two Different Anti-TNFα Drugs

In case study 1, EFA was used to predict the effective dose of two well-studied anti-TNFα agents: adalimumab and infliximab for the treatment of rheumatoid arthritis (RA). Despite the shared target and indication, the two drugs have different binding and PK properties, and have different approved dose and regimen. The approved clinical dose for adalimumab in RA is 40 mg every other week administered through SC injection, although some patients not receiving methotrexate benefit from 40 mg every week. (Adalimumab, 2021) For RA patients treated with infliximab, the clinically approved dose begins at 3 mg/kg IV at 0, 2 and 6 weeks followed by a maintenance dose administered once every 8 weeks. There is a potential benefit of increasing dose to 10 mg/kg IV once a month. (Infliximab, 2013) For this analysis, model predictions are compared to the maintenance dose of infliximab. To predict these doses from first principles, drug-specific and target-specific parameters were defined for input into the model (Table 2). A 1-compartment monospecific anti-ligand model was chosen (Figure 1), focusing on the interaction of the soluble TNFα with drug within the vascular and interstitial fluid.

**TABLE 2:** Adalimumab and infliximab (TNFα) model parameters.

Parameter	Value	Unit	Reference
Drug Valency	1	-	Lim et al., 2018; Tran et al., 2017
Adalimumab Dosing Interval	2	weeks	Adalimumab, 2021
Infliximab Dosing Interval	8	weeks	Infliximab, 2013
Adalimumab Half-Life	20	days	Adalimumab, 2021; Weisman et al., 2003; Ternant et al., 2015
Infliximab Half-Life	14	days	Hemperly and Niels Vande, 2018
Adalimumab Molecular Weight	148,000	Daltons	Adalimumab, 2021
Infliximab Molecular Weight	149,100	Daltons	Infliximab, 2013
Adalimumab KD	8.6	pM	Kaymakcalan et al., 2009
Infliximab KD	4.2	pM	Kaymakcalan et al., 2009
TNF Concentration	5.73e-5	nM	Takeuchi et al., 2011
TNF Half-Life	30	min	Moritz et al., 1989
TNF:TNFR KD	19	pM	Grell et al., 1998
TNFR Concentration	0.23	nM	Bottom up calculation
TNFR receptor half-life	9	hr	Higuchi 1994
Volume	5	L	Typical volume of distribution for mAb
Body weight	70	kg	Typical body weight for man

Target-specific parameters for TNFα included in the model include the representative TNFα concentration in plasma of RA patients (Tekeuchi et al., 2011), as well as its half-life as evaluated from PK studies of recombinant TNFα (Moritz et al., 1989). Membrane TNFR1 expression levels, its turnover half-life (Higuchi 1994) and the affinity of the TNFα:TNFR complex (Grell et al., 1998) were also included in the model. The TNFR1 expression level was calculated from the bottom-up approach described in the methods. (See Supplementary Material for detailed calculations.) TNFR1 is broadly expressed in all human tissues (Holbrook et al., 2019), so TNFR1 expressing cells were calculated assuming a high percentage of nucleated cells in the human body express the receptor (Sender et al., 2016). Absolute expression levels (receptors/cell) were determined from Scatchard analysis of TNFα binding sites (Imamura et al., 1987; Michishita et al., 1990).

Drug specific parameters included valency, target-binding affinity, and the drug half-life. The effective valency of adalimumab and infliximab was considered 1, based on observation of 1:1, 2:2 and 3:3 complexes of the bivalent antibodies to the TNFα homotrimer (Tran et al., 2017; Lim et al., 2018). The affinity of each drug to TNFα was taken from Kinexa measurements, with a Kd of 8.6 pM for adalimumab and 4.2 p.m. for infliximab (Kaymakcalan et al., 2009). Drug PK parameters (half-life of linear elimination) were 20 days for adalimumab (Weisman et al., 2003; Ternant et al., 2015) and 14 days for infliximab (Hemperly and Niels Vande, 2018). The absorption half-life for SC administration of adalimumab was assumed to be 2.5 days based on typical values for antibodies (Kagan, 2014).

**FIGURE 2:**
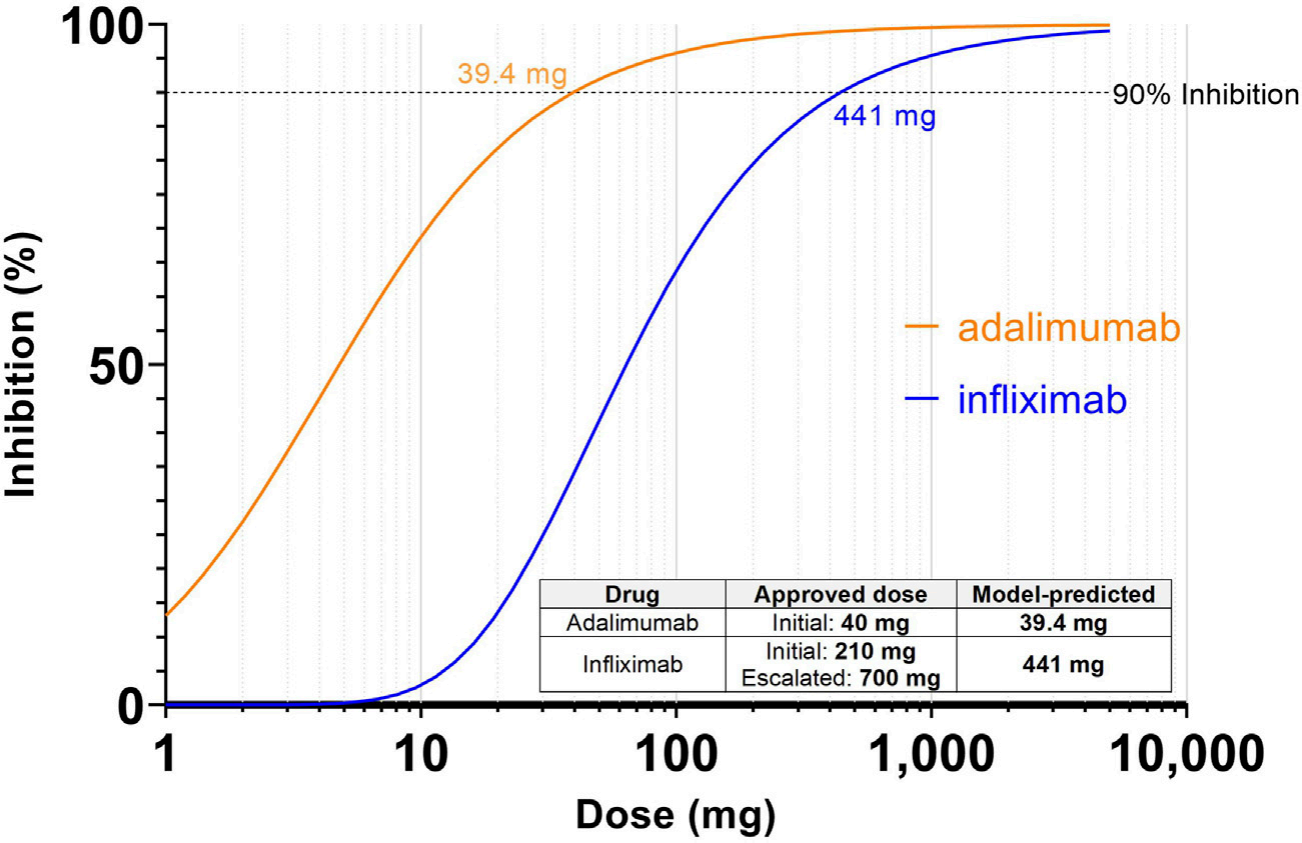
Simulations predicting dose to achieve 90% inhibition of TNF:TNFR complex for adalimumab and infliximab. Inhibition is defined as reduction from the pretreatment target ligand:receptor binding at the steady state trough. Inset table shows the model-predicted dose based on inhibition vs. clinically approved dose for each drug. For infliximab, the milligram dosage assumes a 70 kg patient.

Inhibition of pretreatment target ligand:receptor binding of >90% was selected as the target inhibition criteria for effective dose prediction in case study 1, where the complex of TNFα and TNFR is held at 90% lower than the pre-treatment levels for the entirety of the dosing interval, after 7 successive doses. Simulations were performed to assess the dose that achieves the target criteria. The model predicted that inhibition of pre-treatment TNFα:TNFR binding by adalimumab reaches 90% inhibition at 39.4 mg Q2W for a nominal patient at steady state (Figure 2). This corresponds closely to the clinically approved initial dosage of 40 mg every other week, and the estimated bioavailable dose of 25.6 mg based on 64% bioavailability. (Adalimumab, 2021) Likewise, infliximab reaches 90% inhibition at 6.3 mg/kg Q8W in the model (Figure 2), which also corresponds closely to the clinically approved dosage of 3 mg/kg to start, with a ramp up to 10 mg/kg if needed. The relationship between dose and trough target inhibition can also be observed in Figure 2. The model predicts ∼2-fold lower dose would be required to sustain 85% inhibition, while ∼2-fold higher dose would be required to sustain 95% inhibition.

Interestingly, these model predicted effective doses are significantly higher than the dose that might be predicted from a more straightforward exposure vs. potency comparison. At 39.4 mg, the trough concentration of adalimumab is predicted to be 93.0 nM which is over 10,000 times the Kd (8.6 pM). The model provides an explanation for this shift—when the drug binds to TNFα there is an increase of total TNFα levels from baseline. This has been shown to occur due to half-life extension effects where the short-lived soluble targets form longer-lived complexes with the administered antibodies (Finkelman et al., 1993; Charles et al., 1999; Berkhout et al., 2019). As a result a higher trough concentration than might be expected is required to achieve the desired inhibition level. This type of interaction between binding and total target levels demonstrated the advantage of applying a mechanistic PKPD model for dose predictions.

### Sensitivity of Effective Dose Predictions for Adalimumab and Infliximab

To assess the sensitivity of model-predicted effective dose on the input parameters, a one-at-a-time parameter scan was performed. The model was simulated with each parameter individually varied 3-fold up and down, while all other parameters were held constant at their nominal value. The parameters were ranked based on the fold-difference between the maximum and minimum dose predicted to achieve 90% inhibition. Parameters that resulted in a greater than 3-fold range of predicted effective dose were further examined.

For both infliximab and adalimumab, target binding affinity (Kd), ligand half-life, drug molecular weight, and compartment volume were identified as sensitive parameters. (Supplementary Tables S1, S2) When varied over an order of magnitude, the resulting effective dose prediction ranged by greater than 3-fold. Drug molecular weight has a direct effect on molar drug concentrations, but is well-defined for antibody-based biotherapeutics. Systemic compartment volume is defined as the volume of distribution of the drug and has a direct effect on drug concentrations. For monoclonal antibodies, the volume of distribution is relatively well-defined (Pearson et al., 1995; Ovacik and Lin 2018). Target binding affinity was identified as an important drug-specific parameter. For both infliximab and adalimumab, binding Kd to TNFα was well-described in the literature and an unlikely source of uncertainty. Drug half-life was only identified as a sensitive parameter for infliximab. This is because infliximab is dosed less frequently (Q8W) than adalimumab (Q2W). This result highlights how the dosing regimen can affect drug parameter sensitivities.

The ligand half-life was the only sensitive target-specific parameter, while ligand concentration was not identified as sensitive. The model provides an explanation for this, as the ligand half-life will impact the degree of ligand accumulation over baseline due to half-life extension effects of drug binding. The fold-increase in ligand levels, rather than absolute baseline concentration, has a larger impact on predicted effective dose. For TNFα, the ligand half-life was identified from PK studies of recombinant TNFα. In the absence of such information, the model analysis suggests that measurements of ligand half-life may be a greater priority during drug development.

### Case Study 2: Effective Dose Prediction for Amivantamab, an Anti-EGFR, Anti-c-Met Bispecific Antibody

In this case example, analysis was extended to a bispecific antibody (BsAb), amivantamab, which is approved for the treatment of patients with non-small cell lung cancer (NSCLC) with EGFR exon 20 insertion mutations. The approved clinical dose for patients under 80 kg body weight is 1050 mg administered weekly for the first 4 weeks, and every 2 weeks thereafter. (Rybrevant, 2021) Amivantamab targets epidermal growth factor receptor (EGFR) and hepatocyte growth factor receptor (c-Met) (Haura et al., 2019). A 2-compartment bispecific anti-receptor x anti-receptor model was chosen for this analysis.

Target-specific parameters included in the model were membrane receptor expression levels in the central and peripheral compartments, and membrane receptor turnover half-life. In addition, soluble c-Met is known to be elevated in patient plasma (Gao et al., 2016), so soluble receptor concentration and turnover were also included in the model. EGFR and c-Met expression levels were calculated from the bottom-up approach described in the methods. (See Supplementary Material for detailed calculations.) Briefly, EGFR expression on monocytes, macrophages, skin keratinocytes, tumor cells, and in various epithelial tissues were identified from functional and IHC staining data (Real et al., 1986; Yano et al., 2003; Chen et al., 2016). Absolute expression levels ranged from 50,000 to >400,000 receptors per cell based on reported values from quantitative flow cytometry assays (Sandoval et al., 2012; Jarantow et al., 2015). Assumptions based on relative expression from semi-quantitative flow cytometry and IHC staining data were used to fill in any data gaps. c-Met expressing tissues and absolutely expression levels were similarly identified (Di Renzo et al., 1991; Bozkaya et al., 2012; Ma et al., 2008; Molnarfi et al., 2012; Panke et al., 2013; Jarantow et al., 2015; Kim et al., 2019). EGFR and c-Met receptor turnover half-lives were parameterized from *in vitro* cell line assays (Li et al., 2008; Sigismund et al., 2008; DaSilva et al., 2020). Target-specific parameters are listed in Table 3.

**TABLE 3:** EGFR and c-met target parameters.

Parameter	Value	Unit	Reference
EGFR expression central	4.57E-02	nmoles	Bottom up calculation
EGFR expression peripheral	1.47E+01	nmoles	Bottom up calculation
EGFR receptor half-life	5	hours	Sigismund et al., 2008
Met expression central	3.20E-02	nmoles	Bottom up calculation
Met expression peripheral	5.86E+00	nmoles	Bottom up calculation
Met receptor half-life	4	hours	Li et al., 2008; Da Silva et al., 2020
soluble Met concentration	5.9	nM	Rosen et al., 2017; Gao et al., 2016
soluble Met half-life	48	hours	Estimate based on protein molecular weight; Li et al., 2017
Central compartment volume	3	L	Plasma volume; Shah and Betts, 2012
Peripheral Compartment volume	13	L	Interstitial volume of peripheral tissues; Shah and Betts, 2012
Body weight	70	kg	Typical body weight for man

**FIGURE 3:**
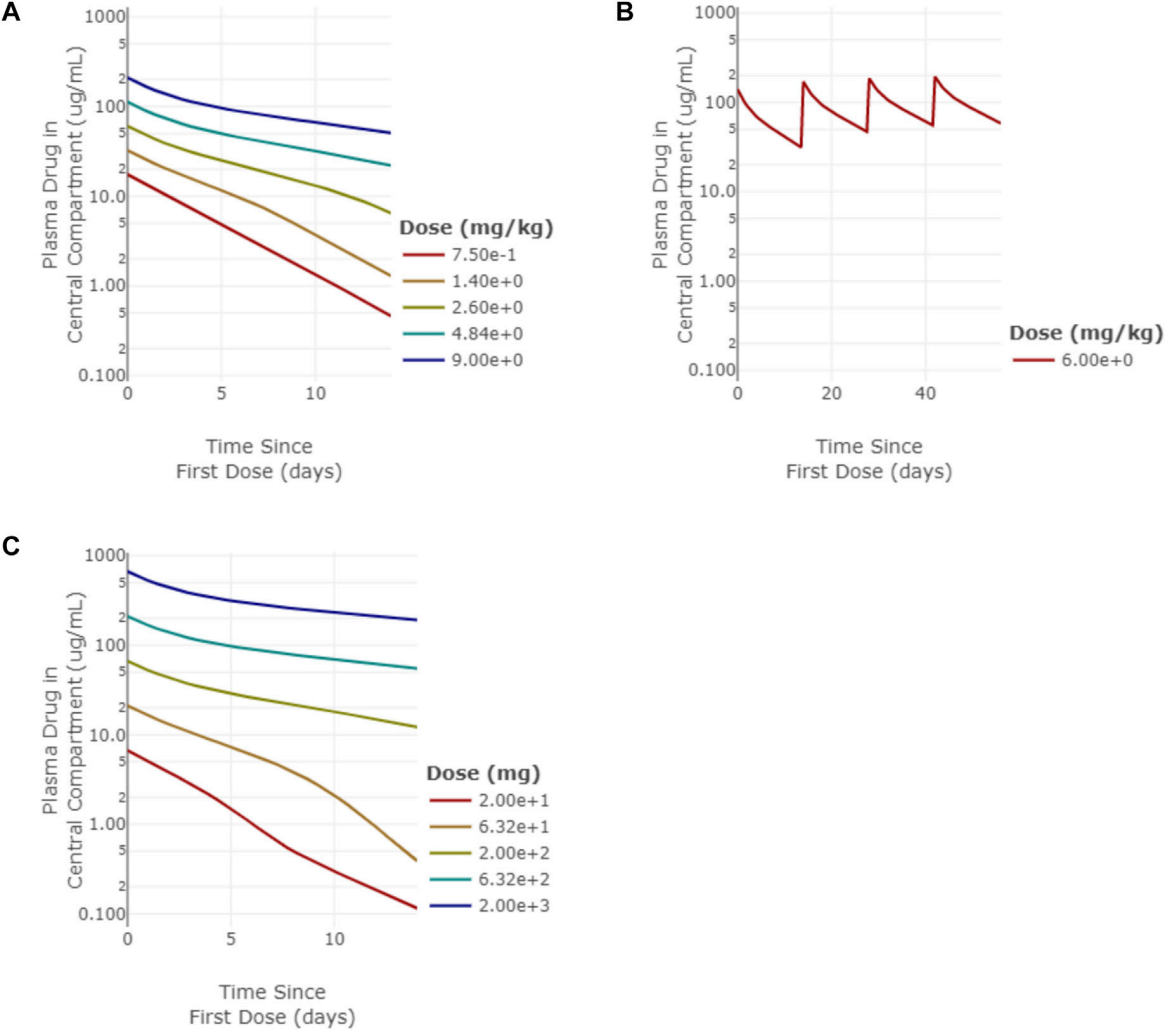
Model simulations of panitumumb and emibetuzumab pharmacokinetics (PK). **(A)** single-dose PK for panitumumab from 0.75–9 mg/kg simulated out to 14 days, **(B)** multi-dose PK for 6 mg/kg Q2W panitumumab simulated out to 64 days, **(C)** single-dose PK for emibetuzumab for 20–2000 mg doses simulated out to 14 days.

PK data from panitumumab, an anti-EGFR monoclonal antibody (mAb), and emibetuzumab (also known as LY2875358), an anti-c-Met mAb, were used to benchmark the target expression estimates since they both exhibit non-linear PK due to target mediated drug disposition (TMDD). For membrane targets such as EGFR and c-Met, the target mediated clearance can impact drug exposure, which then impacts target engagement (Stein and Peletier, 2018). A 2-compartment monospecific anti-receptor model was used to simulate pharmacokinetics (PK) and target engagement (TE) for each of the mAbs using their respective target parameters. Drug-specific parameters are listed in Table 4. Panitumumab target binding affinity (Kd = 0.05 nM) and drug half-life (half-life of linear elimination = 16 days) were taken from literature (Yang et al., 2001; Ma et al., 2009). Simulated PK agreed well with clinical PK measurements. Linear clearance was predicted at doses above 2.5 mg/kg for panitumumab. (Figure 3A). Model simulations of 6 mg/kg Q2W IV panitumumab (Figure 3B) projected peak and trough concentrations of 185 μg/ml and 54 μg/ml, respectively, after 3 doses, while reported values are 213 ± 59 and 39 ± 14 μg/ml (Ma et al., 2009). Since near complete inhibition of EGFR has been shown necessary to induce cell cycle arrest or cell death (Park and Lemmon 2012), a target engagement criteria of >98% in the peripheral compartment was chosen to predict effective dose. The dose projected to achieve >98% sustained target engagement for panitumumab was 162 mg Q2W, which is within 3-fold of the approved dose of 6 mg/kg every 14 days (*i.e.* 420 mg assuming 70 kg man). Emibetuzumab target affinity (Kd = 0.1 nM) and linear PK parameters (half-life of linear elimination = 16 days) were taken from literature (Liu et al., 2014; Rosen et al., 2017). Model simulations of emibetuzumab predicted linear clearance at 700 mg and above, consistent with clinical measurements. (Figure 3C) (Rosen et al., 2017). Model also predicted >98% target engagement at doses 105 mg Q2W and higher, consistent with pharmacodynamic measurements demonstrating saturation of the increase of soluble c-Met at the 210 mg Q2W dose level (Rosen et al., 2017).

**TABLE 4:** Drug specific model parameters for panitumumab, emibetuzumab, amivantamab.

Parameter	Value	Unit	Reference
Panitumumab Valency	2	-	Yang et al., 2001; Ma et al., 2009
Panitumumab Dosing Interval	2	weeks	Ma et al., 2009
Panitumumab Half-Life	16	days	Yang et al., 2001; Ma et al., 2009
Panitumumab KD for EGFR	0.05	nM	Yang et al., 2001; Ma et al., 2009
Emibetuzumab Valency	-	2	Liu et al., 2014; Rosen et al., 2017
Emibetuzumab Dosing Interval	2	weeks	Rosen et al. (2017)
Emibetuzumab Half-Life	16	days	Liu et al., 2014; Rosen et al., 2017
Emibetuzumab KD for c-Met	0.1	nM	Liu et al., 2014; Rosen et al., 2017
Amivantamab Valency	-	1	Jarantow et al., 2015
Amivantamab Dosing Interval	2	weeks	Rybrevant, 2021
Amivantamab Half-Life	11	days	Rybrevant, 2021
Amivantamab KD for EGFR	1.4	nM	Jarantow et al. (2015)
Amivantamab KD for c-Met	0.04	nM	Jarantow et al. (2015)
Drug Molecular Weight	150,000	Daltons	Assumed typical mAb MW for all drugs
Pdist12	0.19	-	Partition coefficient between central and peripheral compartments assumed typical (Betts et al., 2018)
Tdist12	35	hours	Half-life of intercompartmental clearance between central and peripheral compartments assumed typical (Betts et al., 2018)

**FIGURE 4:**
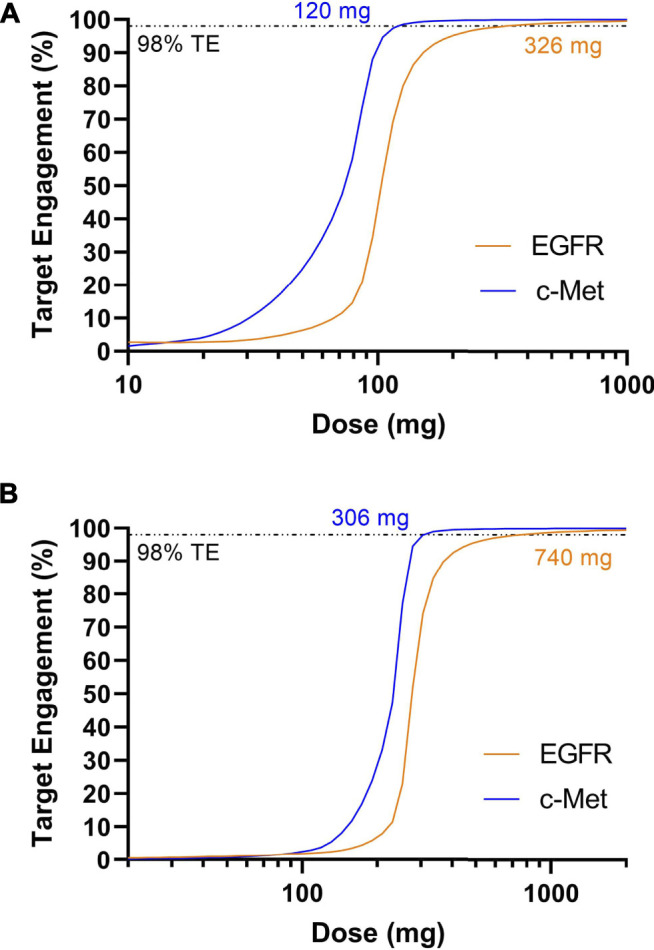
Model predicted trough target engagement for amivantamab dosed Q1W **(A)** or Q2W **(B)**. 98% target engagement for EGFR was achieved at 326 mg Q1W or 740 mg Q2W. 98% target engagement for c-Met was achieved first at 120 mg Q1W and 306 mg Q2W.

Next, dosing of amivantamab was simulated using the benchmarked target parameters. JNJ-61186372 binding to EGFR has a Kd ∼1.4 nM; binding to c-Met has a Kd ∼0.04 nM (Jarantow et al., 2015). The half-life of amivantamab was reported to be approximately 11 days (Rybrevant, 2021) Once again, a target engagement of >98% for both targets was set as criteria for the effective dose. The model predicted 326 mg Q1W or 740 mg Q2W is required to achieve sustained target engagement >98% for both targets (Figure 4). This dose prediction, generated with minimal data, is consistent with the 1050 mg Q2W dosing after the initial 4 weeks.

### Case Study 3: Application of EFA to Predict Effective Dose of 6 Additional Biotherapeutic Drugs

The methodology described in case studies 1-2 was extended to predict effective dose of 6 additional biotherapeutic drugs targeting a range of soluble or membrane-bound targets. Simulations were run using the drug-specific and target-parameters for a total of 9 biotherapeutics. Targets include TNFα, IL-23/IL-2, BLyS (BAFF), IgE for soluble targets and HER2, EGFR, and EGFR/c-Met for membrane-bound targets. Drug-specific and target-specific parameters were obtained from the literature as described in Methods and are listed in Supplementary Tables S3–S7. Approved doses and regimens for the respective indications in RA, plaque psoriasis, systemic lupus erythematosus (SLE), asthma, breast cancer, colon cancer, and non-small cell lung cancer (NSCLC) were collated for comparison to model predictions (Table 5).

**TABLE 5:** Effective dose predictions for a panel of biotherapeutics.

Drug	Model predicted dose		Clinically approved dose	
	Model in Applied BioMath Assess ™	ID90/TE98 (mg)^a^	Dose (mg)	Schedule^a^
Remicade (infliximab)	Monospecific anti-ligand	441	210	8 weeks IV
Humira (adalimumab)	Monospecific anti-ligand	39.4	40	2 weeks SC
Stelara (ustekinumab)	Monospecific anti-ligand	22.4	45	12 weeks SC
Skyrizi (risankizumab)	Monospecific anti-ligand	273	150	12 weeks SC
		37.1	150	4 weeks SC
Benlysta (belimumab)	Monospecific anti-ligand	252	200	1 week SC
		1700	700	4 weeks IV
Xolair (omalizumab)	Monospecific anti-ligand	330	225	2 weeks SC
Herceptin (trastuzumab)	Monospecific anti-receptor (4 compartment)	79.0	140	1 week IV
Vectibix (panitumumab)	Monospecific anti-receptor (4 compartment)	162	420	2 weeks IV
Rybrevant (amivantamab)	Bispecific anti-receptor x anti-receptor (4 compartment)	740	1050	2 weeks IV

a ID90 = dose to achieve 90% inhibition, TE98 = dose to achieve 98% target engagement, SC = subcutaneous administration, IV = intravenous administration.

**FIGURE 5:**
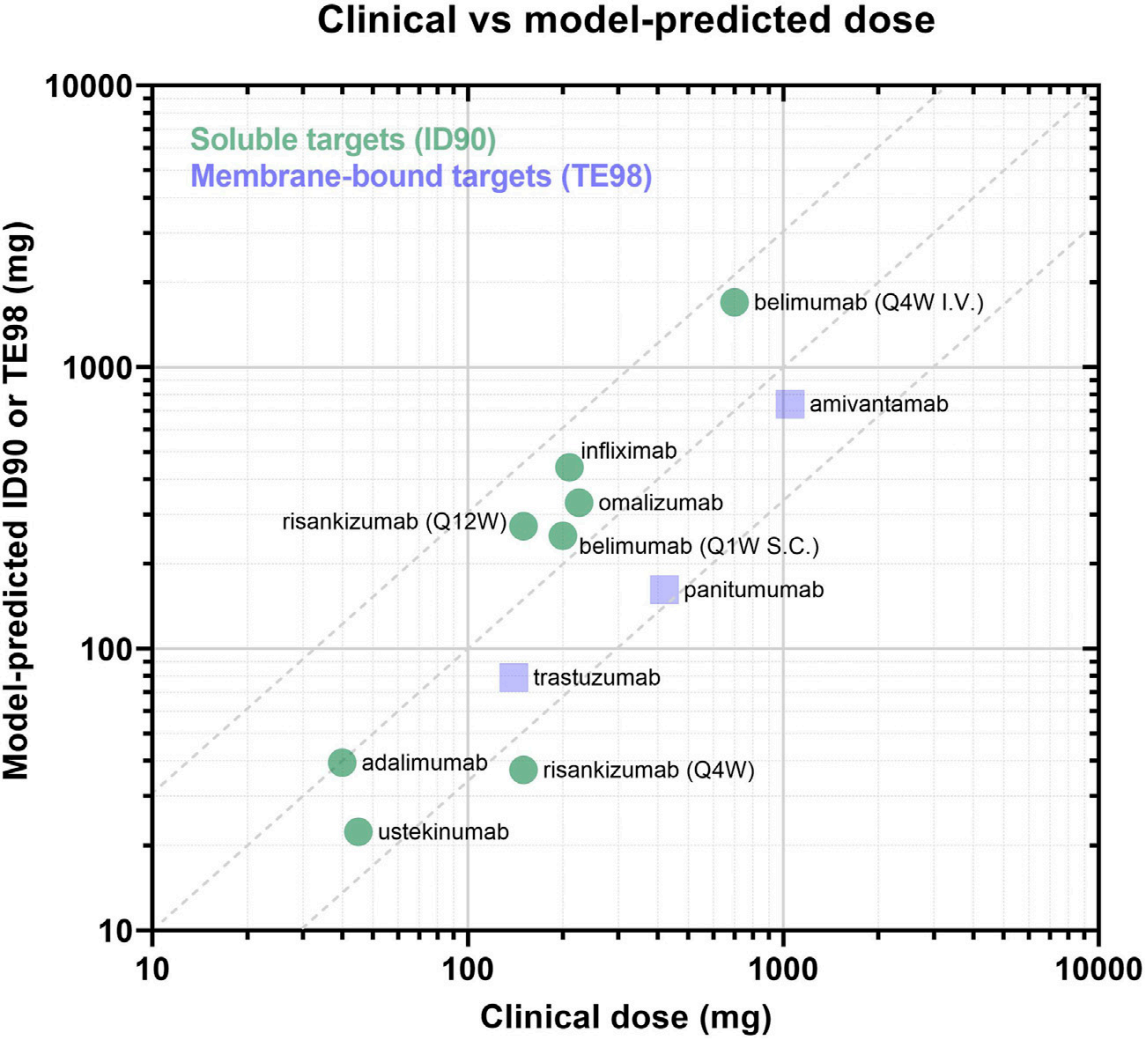
Panel of model-predicted vs. clinically approved dose. Drugs with soluble targets are displayed as green circles and are evaluated based on ID90 (i.e., inhibition of baseline ligand:receptor complex). Drugs with membrane-bound targets are displayed in purple squares and are evaluated by TE98 (i.e., percent of target bound by drug). Dotted lines define the region where model-predicted effective doses fall within 3-fold of the clinically-approved doses.

Across the panel of drugs, the model-informed effective doses based either on 90% inhibition (soluble targets) or 98% target engagement (membrane targets) criteria were largely within 3-fold of the clinically approved doses (Figure 5; Table 5), for the diverse soluble targets (e.g., cytokines, IgE) as well as surface receptors (e.g., HER2). There appears to be a trend towards systematic overprediction of the doses for soluble targets and underprediction of the doses for membrane targets (Figure 5).

Overall, the analysis serves as a proof-of-principle that EFA with mechanistic PKPD modeling approaches can predict the effective doses with sufficient accuracy to inform drug design decisions and evaluation of the feasibility of drug targets and disease areas before PK and PD data are available for the drug.

## Discussion

At early stages of drug discovery and development, PKPD data required to inform clinical dosing is not yet available. To generate such data may involve the development of reagents, tool molecules, and assays, which is both costly and time consuming. Once that data is collected, it may suggest a different lead optimization strategy than was originally planned which can cause further delays. In this manuscript, Early Feasibility Assessment (EFA) is demonstrated, based upon integrating data that is available at an early stage, including in-house *in vitro* experiments and literature, into a mechanistic PKPD model of target binding to predict PK (including TMDD), target engagement, and effective dose. EFA centers on defining a notion of “dose feasibility,” that a drug may be administered with a reasonable dosing regimen and conceivably achieve a therapeutic impact. Note that feasibility is distinct from efficacy which requires both a significant pharmacological impact and a meaningful biological response to the impact. However, there is still utility in using feasibility as a decision making criteria, to ensure that molecules are developed with the best chance to test the therapeutic hypothesis, and resources are not spent developing molecules unlikely to modulate the target to a sufficient degree to be drug candidates.

Two detailed case study examples, extended to a total of 9 biotherapeutics, were presented, demonstrating the ability of EFA to make clinically relevant predictions of effective dose. The predicted effective doses in this work were generally within ∼3-fold of the clinically-approved dose. Factors not considered in these models, such as tolerability, can impact the final clinical dose; however, generating dose estimates within 3-fold during the early stages of a program can be useful for various decisions early in drug discovery, including target prioritization, optimal drug properties for a target product profile (TPP), prioritization of different drug concepts. Using validated mechanistic PKPD models parameterized from literature and *in vitro* measurements, questions about target druggability, ease of engineering a lead molecule with required drug properties, feasibility of novel drug concepts can be answered.

In all of the cases presented, the models were parameterized by data that should be available to an early program. It is significant that target-specific parameters can be identified from the “bottom-up” calculations leveraging literature data; however, there is uncertainty and biological variability in these values that should be considered. When extending this approach to novel targets, this uncertainty is higher. A sensitivity analysis by examining the impact of dose predictions over a range of target parameters can determine if they are important to the conclusions and help prioritize potential experiments that will minimize risk during drug development. For infliximab and adalimumab, a parameter scan identified TNFα half-life as a sensitive parameter that can impact the model-predicted effective dose, while varying TNFα concentration, TNF Receptor concentration, and TNF Receptor half-life had minimal impact. For a novel program, this result would suggest that measurement of TNFα half-life should be prioritized for accurate dose predictions during later stages of drug development.

When using EFA for early stage programs, drug-specific parameters (such as affinity and half-life) may be theoretical targets as part of a TPP. While drug-specific PK parameters were in this analysis, mAbs generally display similar linear pharmacokinetics, which enables predictions to be made using assumed standard values, or derived from measurements made in preclinical model species (Deng et al., 2011; Dong et al., 2011; Betts et al., 2018). This approach is not limited to mAbs, but can be extended to other biotherapeutics with well-described PK and binding behavior. As demonstrated by the infliximab and adalimumab parameter scans, the sensitivity of effective dose predictions to drug design parameters can depend on the desired dosing interval. For a novel therapeutic, a sensitivity analysis on how much drug properties impact dose predictions can inform ease of the development and potential need for additional drug optimization through affinity maturation or half-life extension, for example.

Finally, there can be uncertainty in defining the criterion for efficacy, which is based on an understanding of the intended mechanism of action for each of these biotherapeutics. In the analyses presented here, the dose predicted to sustain >90% target inhibition was comparable to the clinically approved doses for all the biotherapeutics against soluble targets. For the membrane receptor targets discussed, sustained, near complete, target engagement is hypothesized to be necessary for therapeutic efficacy, and a criterion of >98% target engagement was used. When applying EFA to novel targets and drug concepts, an understanding of the intended mechanism of action is necessary, and an exploration of the impact of different metrics of efficacy may be warranted.

When performing EFA, model selection must be carefully considered. In each of the case studies presented in this manuscript, the selected models were built on first-principles that captured the key pharmacological mechanisms for each of the drugs. Drugs binding to a soluble target vs. a membrane receptor target require different models which are associated with different assumptions. For drugs targeting soluble factors, the binding and elimination of drug-bound target is an important factor to consider mechanistically. For drugs targeting membrane receptors, the elimination of drugs through target binding was captured mechanistically. For more complex biotherapeutic modalities where models of similar scale that capture the pharmacology exist, it would be reasonable to apply this type of analysis. For example, a model of T-cell engagers that describes crosslinking of target receptor and CD3 receptors on T-cells as a model endpoint is available in Applied BioMath Assess ™, and similar models have been reported in literature (Chen et al., 2021). This analysis could potentially be extended to questions of therapeutic index by comparing model endpoints in disease and toxicity compartments, for example. Striking a pragmatic balance between mechanistic detail and the cost or complexity of parameterizing a model is a defining feature of EFA.

The focus of this manuscript is on antibody therapeutics (mAbs, BsAbs) where typical PK properties such as half-life and biodistribution, and pharmacology parameters such as binding affinity are well known. mAbs are a large and growing category of new drugs approved each year—as of December 2019, there were 79 therapeutic mAbs approved, with 18 approved between 2018 and 2019 (Lu et al., 2020). In 2021, the 100th antibody was approved (Mullard 2021). There is the potential to expand this methodology to other pharmacologies (*e.g.* ADCs, LNPs, peptides, oncolytic viruses, etc.) if reasonable ranges for these parameters can be determined *a priori*, or in combination with methods that allow the prediction of PK properties such as *in vitro in vivo* correlation (IVIC).

Overall, the application of EFA at the early stages of a program, before the major clinical costs are incurred, has great potential to realize efficiencies and reduce attrition in drug development. By excluding targets that don’t have a chance of “druggability” early, resources can be prioritized for those programs that may be more likely to succeed. By identifying parameters that strongly impact an eventual clinical dose, programs can also identify knowledge gaps that, once filled, could reduce program risk. As prioritized programs progress, preclinical data on the drug candidate(s) binding mechanisms and pharmacokinetics should be incorporated into these models. Additional complexity in terms of biological mechanisms, downstream pharmacology can also be incorporated. These updated models can then enable decisions at later stages of drug development, such as lead selection, first-in-human dose selection, and recommended phase 2 dosing.

## Data Availability

The original contributions presented in the study are included in the article/Supplementary Material, further inquiries can be directed to the corresponding author.
