# Supplementary material for: Early Feasibility Assessment: A Method for Accurately Predicting Biotherapeutic Dosing to Inform Early Drug Discovery Decisions
Source: Front Pharmacol. 2022 Jun 8;13:864768. doi: 10.3389/fphar.2022.864768 (PMC9214263; doi:10.3389/fphar.2022.864768)
Supplement: Supplementary file 2 [file DataSheet3.pdf]

Supplementary Material

1 Supplementary Figures and Tables

1.1 Supplementary Figures

Figure S1. Monospecific Anti-Receptor Model Diagram. Figure was created using BioRender.com.

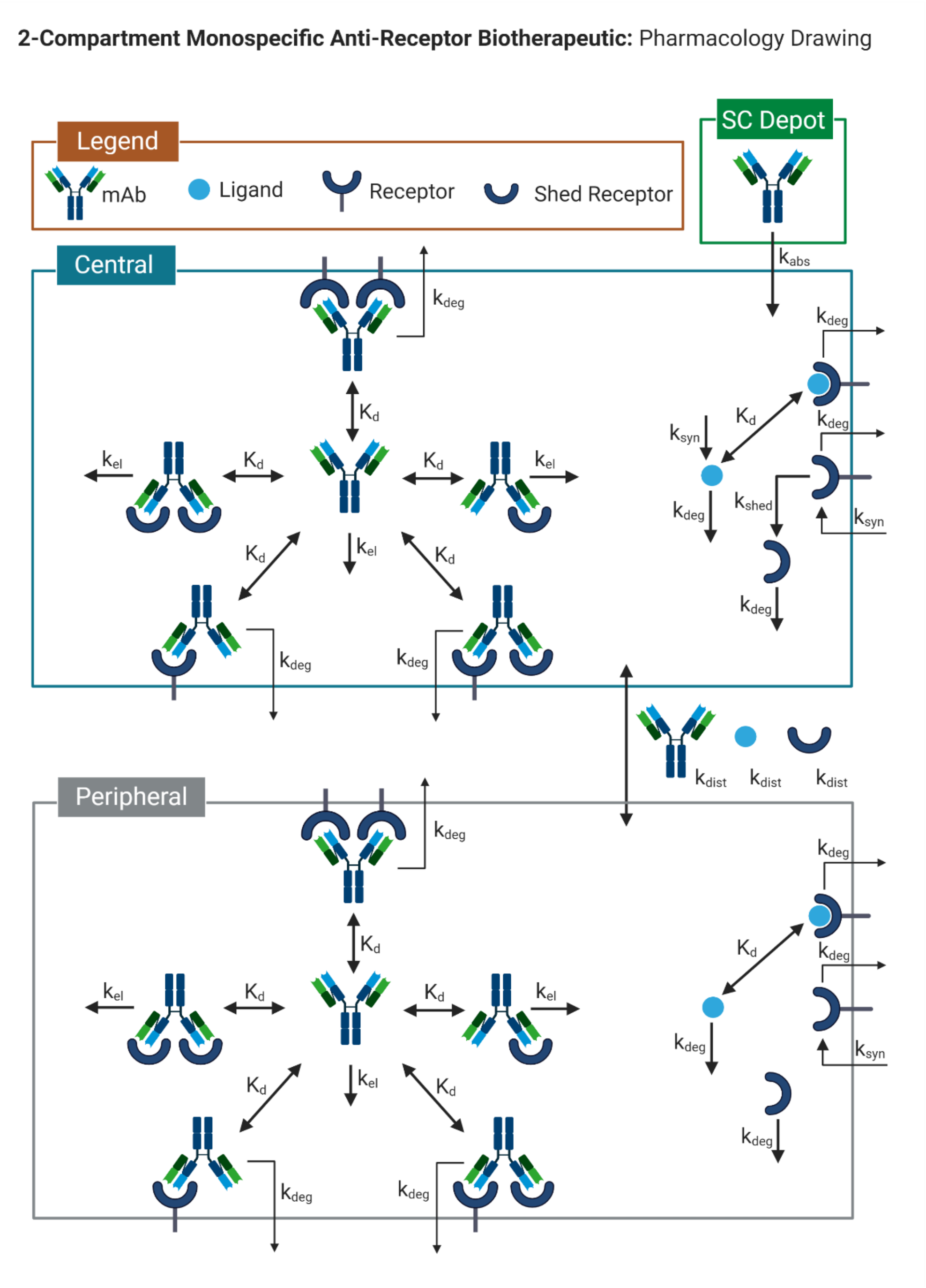

Figure S2. Bispecific Anti-Receptor x Anti-Receptor Model Diagram. Figure was created using BioRender.com.

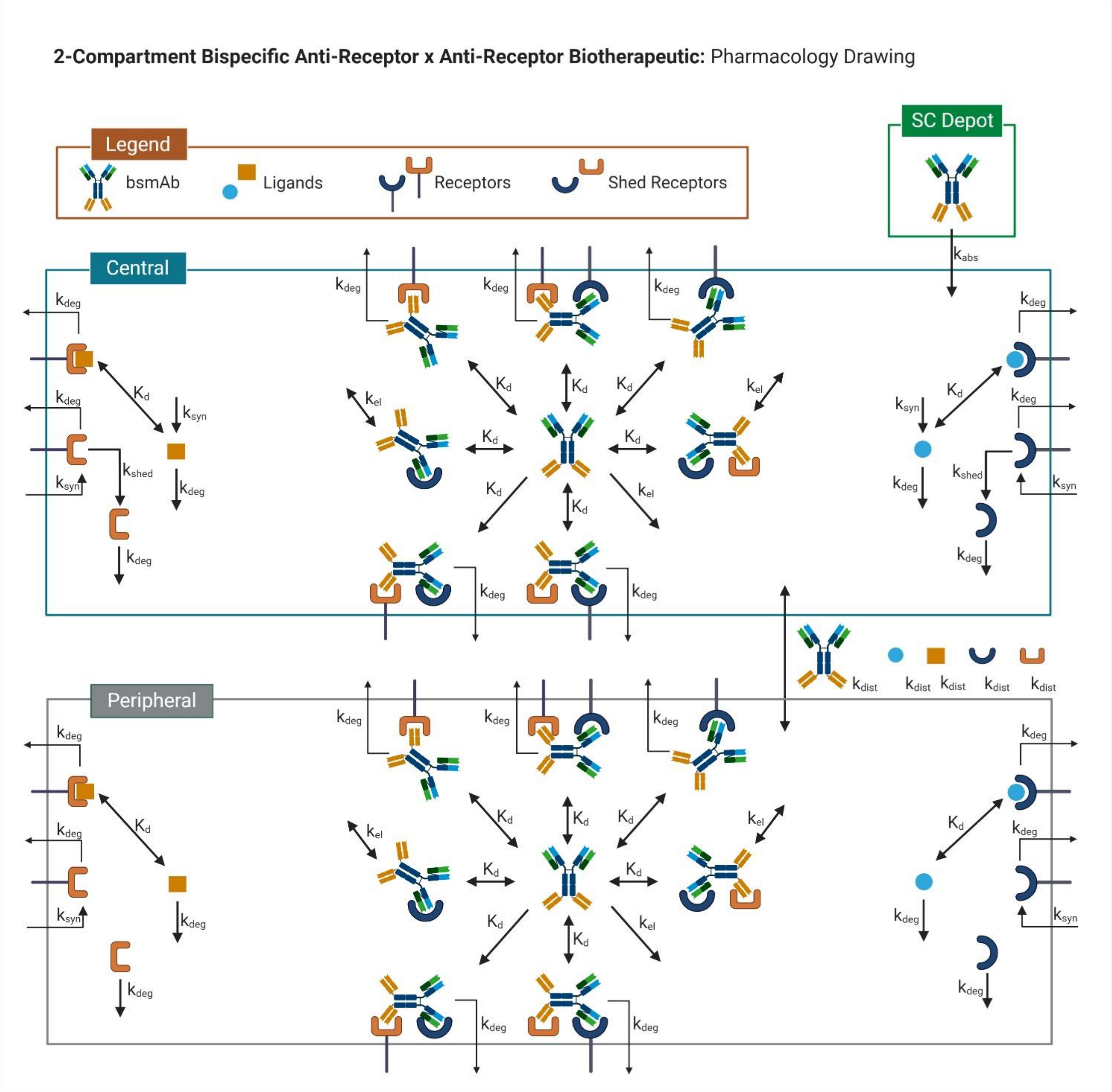

1.2 Supplementary Tables

Table S1. Adalimumab Parameter Scan Results

Parameter Description	Unit	Scan Range	ID90 Range (mg)	ID90 Fold-range
Adalimumab molecular weight	Da	49333 - 444000	13.1 - 118	9.0
Volume	L	1.67 - 15	13.1 - 118	8.9
Adalimumab KD	nM	0.0027 - 0.024	14.6 - 114	7.8
TNF Half-Life	min	10 - 90	19.5 - 99.4	5.1
Adalimumab Half-Life	days	6.67 - 60	35.2 - 57.0	1.6
TNFR Concentration	nM	0.077 - 0.69	39.4 - 58.4	1.5
TNFR receptor half-life	min	180 - 1620	34.7 - 44.0	1.3
TNF:TNFR KD	nM	0.006 - 0.057	35 - 43.7	1.2
SC Absorption half-life	days	0.83 - 7.5	36.5 - 42.8	1.2
TNF Concentration	nM	1.92E-05 - 1.73E-04	39.4 - 39.6	1.0

Table S2. Infliximab Parameter Scan Results

Parameter Description	Unit	Scan Range	ID90 Range (mg)	ID90 Fold-range
Infliximab Half-Life	days	4.67 - 42	128 - 40000	310
Infliximab molecular weight	Da	49667 - 447000	148 - 1330	9.0
Volume	L	1.67 - 15	148 - 1330	9.0
Infliximab KD	nM	0.0014 - 0.0126	182 - 1230	6.8
TNF Half-Life	min	10 - 90	219 - 1120	5.1
SC Absorption half-life	days	0.83 - 7.5	270 - 509	1.9

TNFR Concentration	nM	0.077 - 0.69	444 - 658	1.5
TNFR receptor half-life	min	180 - 1620	390 - 496	1.3
TNF:TNFR KD	nM	0.006 - 0.057	395 - 492	1.2
TNF Concentration	nM	1.92E-05 - 1.73E-04	444 - 446	1.0

Table S3. Ustekinumab (IL12/ IL23) Model Parameters

Parameter	Value	Unit	Reference	Notes
Drug Valency	2	-	Benson (2011) PMID: 22123062	Through isothermal titration calorimetry analysis, ustekinumab was shown to bind IL-12 and IL-23 equally, with the expected 2:1 antigen-to-antibody stoichiometry.
Drug Dosing Interval	12	weeks	Stelara Label	45 mg (>60 to ≤100 kg subjects) or 90mg (≥100 kg) s.c. initially and 4 weeks later, followed by 45 mg every 12 weeks*
Drug Half Life	21.6	days	Zhu (2009) PMID: 19179295	Consistent with Gottlieb 2007 and Kauffman 2004
Drug Molecular Weight	148,600	Da	CAS #: 815610-63-0	
Ustekinumab KD	1	pM	Luo (2010) PMID: 20691190	Exceeds the isothermal titration calorimetry limits. Assume < 10 pM as 1 pM
p-40 Concentration	1.9	pmols		Bottom-up estimate

p-40 Half Life	1.5	hrs	Lotze (1985) PMID: 3871099	IL-12: 5-10 h (Lotze 1996 PMID: 8958977) (which may be high and influenced by engineered properties of recombinant IL-12); IL-23 not found. Assume 1.5 h (based on IL-2)
p-40:Receptor KD	10	pM	Ma (2001) PMID: 11680011	Both high-affinity (Kd=5–20 pM, 100–1000 sites per cell) and low- affinity (Kd=2–6 nM, 1000–5000 sites per cell).
Receptor Concentration	61	pmols		Bottom-up estimate
Receptor Internalization	2	hrs		Standard assumption
Volume	5	L	-	Standard PK assumption. The typical volume of distribution for antibody drugs is 5L.
Body Weight	70	kg	-	Standard PK assumption.

Table S4. Risankizumab (IL23) Model Parameters

Parameter	Value	Unit	Reference	Notes
Drug Valency	2	-	assumed as monoclonal antibody	
Drug Dosing Interval	12	weeks	Gordon (2018) PMID: 30097359	150 mg s.c.at weeks 0, 4, 16, 28, and 40 (Ph3 dose); assume longest last interval

Drug Half Life	27	days	Suleiman (2019) PMID: 30123942	Includes patients with psoriasis and Crohn's disease
Drug Molecular Weight	145,610	Da	KEGG entry https://www.genome.jp/dbget-bin/www_bget?dr:D11052	
Risankizumab KD	1	pM	Singh (2015) PMID: 25905918	assumed < 10 pM, from Table 1; use 1 pM
p-19 Concentration	872	pM		Bottom up-estimate
p-19 Half Life	1.5	hr	Lotze (1985) PMID 3871099	Assumed similar to IL-2
p-19:Receptor KD	1.6	nM	Parham (2002) PMID: 12023369	Estimated Kd range (0.3-3nM) based on ELISA binding assay; Figure 4
Receptor Concentration	4.4	nmols		Bottom-up
Receptor Internalization	2	h		Standard Assumption
Volume	5	L	-	Standard PK assumption. The typical volume of distribution for antibody drugs is 5L.

Body Weight	70	kg	-	Standard PK assumption.

Table S5. Belimumab (BAFF) Model Parameters

Parameter	Value	Unit	Reference	Notes
Drug Valency	1	-	Shin (2018) PMID: 29572471	BAFF forms a homotrimer and binds to belimumab with 3:3 stoichiometry
Drug Dosing Interval	7	days	Benlysta label	10 mg/kg i.v. BIW x3 then Q4W; or 200 mg s.c. QW; use the 1 week sc
Drug Half Life	19.4	days	SumR BLA; PharmR BLA	19.4 d (10mg/kg dose); linear PK
Drug Molecular Weight	147,000	Da	Goldenberg (2011) PMID: 21785542	
Belimumab KD	274	pM	PharmaR BLA	
BAFF Concentration	1.47	nmols		SLE patient BAFF levels central and peripheral compartments are 0.307 and 1.16 nmoles, respectively. Total body soluble BAFF is 1.47 nmoles
BAFF Half Life	60	min	Moritz (1989) PMID: 2720707	60 min (54-70 min) based on TNFalpha at the higher doses, 11 -17 mins at 0.04 mg/m2)
BAFF:Receptor KD	15; 1550; 1.3	KD	Day (2005) PMID: 15697217; Cachero (2006) PMID: 16475789; Hymowitz (2005) PMID: 15542592	BAFF binds to BAFF-R, BCMA, and TACI with 15-16, 1550, and 1.3 nM KDs, respectively

Receptor Concentration	290	pmols		Total BAFF-R, BCMA, and TACI in SLE patient blood is 5.7, 0.36, and 0.24 pmoles, respectively. Total BAFF-R, BCMA, and TACI in the peripheral compartment of SLE patients is 260, 16, and 11 pmoles, respectively. Total body BAFF binding partner is 290 pmoles.
Receptor Internalization	2	h	Standard Assumption	
Volume	5	L	-	Standard PK assumption. The typical volume of distribution for antibody drugs is 5L.
Body Weight	70	kg	-	Standard PK assumption.

Table S6. Omalizumab (IgE) Model Parameters

Parameter	Value	Unit	Reference	Notes
Drug Valency	2	-	Davies (2017) PMID: 28438838	Crystal studies suggest omalizumab can bind with a 2:1 stoichiometry to target, or sometimes 2:2 or 3:3
Drug Dosing Interval	14	days	Omalizumab label	150-375 mg s.c. Q2W or Q4W. *Dose varies by weight and IgE serum levels.* For 70kg patient, dosed 150 and 300mg for 30-100 and 100-200 IU/mL IgE, respectively.* 1 IU/mL is equal to 2.4ng/mL.**
Drug Half Life	26	days	Omalizumab label	

Drug Molecular Weight	149000	Da	Omalizumab label	
Omalizumab KD	20	pM	Chang (2000) PMID: 10657120; Kolbinger (1993) 8309946	0.1 nM, but can be higher affinity due to avidity of anti-IgE:IgE immune complexes (2:2 or 3:3)§‡, assume 20 pM
IgE Concentration	40	nmols		Use IgE plasma concentration corresponding to measured receptor density in same set of patients (222 IU/ml or 533ng/ml #, range within study <20–500 IU/ml †). free IgE levels in central and peripheral are 8.4 and 32 nmoles, respectively. May be a high estimate due to serum dilution. Also, levels of membrane-bound IgE on B cells not found and assumed to be zero. Total body free IgE is 40 nmoles
IgE Half Life	2	days	Corne (1997) PMID: 9062345	Complex half-lives may be about 10 days
IgE:Receptor KD	0.12	nM	Miller (1989) PMID: 2523561	FCεRI binding in transfected cells

Receptor Concentration	3.9	nmols		Bottom up calculation of FCεRI expression on basophils and mast Cells. Assume levels of other FCεRI expressing cells (dendritic cells, Langerhans cells, macrophages, and eosinophils) are negligible. FCεRI expression on basophils in central and peripheral compartments is 0.054 and 2.4 pmoles, respectively. FCεRI expression on mast cells in peripheral compartment is 1.4 nmoles. Total body FCεRI expression is 3.9 nmoles. NOTE: Treatment with the anti-lgE mAbs results in a marked down-regulation of FcrRl on basophils. Median pretreatment densities of FcrRl were -220,000 receptors per basophil and after 3 mo of treatment, the densities had decreased to a median of 8,300 receptors per basophil MacGlashan (1997) PMID: 9013989
Receptor Internalization	2	hr	standard assumption	Try both occupancy of soluble IgE (no receptor turnover) and IgE/FcεRI inh (2 h). IgE binding to FcεRI thought to inhibit endocytosis in mast cells and basophils but not monocytes or dendritic cells.
Volume	5	L	-	Standard PK assumption. The typical volume of distribution for antibody drugs is 5L.
Body Weight	70	kg	-	Standard PK assumption.

Table S7. Trastuzumab (HER2) Model Parameters

Parameter	Value	Unit	Reference	Notes

Drug Valency	2	-	assumed as monoclonal antibody	
Drug Dosing Interval	7	days	Herceptin Label	For metastatic HER2-Overexpressing Breast Cancer: Initial dose of 4 mg/kg followed by weekly doses of 2mg/kg
Drug Half Life	25	days	Quartino (2019) PMID: 30467591	From pop-PK analysis
Drug Molecular Weight	145531	Da		
Trastuzumab KD	0.1	nM	Li (2013) PMID: 24046294; Troise (2008) PMID:18795950	Apparent Kd from binding to ErbB2-ECD. Values can vary depending on assay.
HER2 expression central	3.60E-04	nM	You (2008) PMID: 18577250, Suzuki (2015) PMID: 25655677	bottom-up calculation
HER2 expression peripheral	5.89E-02	nM	Li (2019) PMID: 31185215, Onsum (2013) PMID: 24035511, Ruiz (2018) PMID: 30282693	bottom-up calculation
HER2 receptor half-life	24	hr	Pereira (2018) PMID: 30510281	in vitro assay in the presence of cyclohexamide
soluble Her2 receptor expression central	7	nM	Baselga (2001) PMID: 11521722	Patient plasma levels. Wide range observed. High levels result in higher clearance of mAb
soluble Her2 receptor	7	nM	Assumed equal to central	

expression peripheral				
soluble Her2 receptor half-life	1	hr	Betts (2020) PMID: 32710210, Li (2014) PMID: 24477089	Estimates from PBPK models
Central compartment volume	3	L	Shah and Betts 2012	Plasma volume
Peripheral compartment volume	13	L	Shah and Betts 2012	Interstitial volume of peripheral tissues
Body weight	70	kg	-	Standard PK assumption.
