# Supplementary material for: Early Feasibility Assessment: A Method for Accurately Predicting Biotherapeutic Dosing to Inform Early Drug Discovery Decisions
Source: Front Pharmacol. 2022 Jun 8;13:864768. doi: 10.3389/fphar.2022.864768 (PMC9214263; doi:10.3389/fphar.2022.864768)
Supplement: Supplementary file 3 [file DataSheet2.ZIP › Model run files_json and reports/README.pdf]

Supplementary Material:

Model Files And Simulation Results for: Early Feasibility Assessment: a method for accurately predicting biotherapeutic dosing to inform early drug discovery decisions

Diana H Marcantonio[1†], Andrew Matteson[1†], Marc Presler[1†], John M Burke[1], David R Hagen[1], Fei Hua[1], Joshua F Apgar[1*]

[1]Applied BioMath, LLC, Concord, MA, USA

[†] These authors have contributed equally to this work and share first authorship.
*** Correspondence:**
Joshua F. Apgar
Applied BioMath, LLC
561 Virginia Road, Suite 220
Concord, MA 01742
apgar@appliedbiomath.com

Applied BioMath Assess Models:

The Applied BioMath Assess model files for the examples used in this article are provided. The models contain descriptions of the:

- Parameters (explicitly set in each scenario)
- Relationships (computed parameters)
- Compartments
- States
- Routes
- Reactions
- Outputs

All reactions are mass action reactions described as either a zeroth, first or second order rate law (depending on the number of reactants). All rate laws are mass action and proceed as the product of the concentration of the reactants times a forward rate constant.

Table: Types of reactions in model files and their corresponding rate law		
**Reaction Type**	**Reaction Scheme**	**Rate Expression**
Zeroth-order	0 -> A, k	k
First-order	A -> B, k	[A] k
Second-order	A + B -> C, k	[A][B] k

The models provided are for the following models that cover all of the examples in the manuscript:

- one_compartment_anti_ligand
- four_compartment_anti_receptor
- four_compartment_anti_receptor_bispecific

Case Studies:

The Assess Model Reports contain the specific parameters used in each case study as well as the plots of the simulations from each scenario. A run file for each report is also provided with the same name of the report with the extension .json. The following table contains the mapping of the drug name to the Assess Model Report and the Model File.

Table: Mapping of Drugs to Assess Model Report File and Assess Model File		
**Drug**	**Assess Model Report**	**Assess Model File**
**Adalimumab**	Adalimumab_Sensitivity_Analysis.pdf	one_compartment_anti_ligand
**Amivantamab**	Amivantamab_CaseStudy.pdf	four_compartment_anti_receptor_bispecific
**Emibetuzumab**	Emibetuzumab_CaseStudy.pdf	four_compartment_anti_receptor
**Herceptin**	Herceptin_CaseStudy.pdf	four_compartment_anti_receptor
**Infliximab**	Infliximab_Sensitivity_Analysis.pdf	one_compartment_anti_ligand
**Panitumumab**	Panitumumab_CaseStudy.pdf	four_compartment_anti_receptor
**Multiple**	All_Anti-Ligand_CaseStudies.pdf	one_compartment_anti_ligand
