# Supplementary material for: Early Feasibility Assessment: A Method for Accurately Predicting Biotherapeutic Dosing to Inform Early Drug Discovery Decisions
Source: Front Pharmacol. 2022 Jun 8;13:864768. doi: 10.3389/fphar.2022.864768 (PMC9214263; doi:10.3389/fphar.2022.864768)
Supplement: Supplementary file 3 [file DataSheet2.ZIP › Model run files_json and reports/one_compartment_anti_ligand.pdf]

```
# Title: One Compartment Anti Ligand Model
# File: one_compartment_anti_ligand
# Author: apgar@appliedbiomath.com
# Website: https://www.appliedbiomath.com/assess
#
# (C)  Applied BioMath, LLC, 2022
# All rights reserved
#
# Notice: Applied BioMath, LLC ("Applied BioMath") retains and
# reserves all rights, title, and interest in and to all Applied
# BioMath-developed methodologies, technologies, and techniques
# embodied by this model file. Applied BioMath's delivery or other
# providing of access to this file  shall not be construed as
# conveying ownership or licensing of any rights, title, or interest
# in or to any such  methodologies, technologies, or techniques.
#

% parameters
kon 0.001
interval 14
dose 100
mab_kd_1 0.1
dose_count 7
mw_1 150000
el_half_1 28
abs_half 2.5
BW 70
volume_central 5
drug_valency_1 2
lig_half_1 30.0
rec_half_1 60
shed_half_1 30.0
lig_rec_kd_1 1
lig_css_1_central 0.05
rec_css_1_central 0.016605390671738465
shed_css_1_central 0
cell_diameter_um 10
cell_density_mL_central 1000000
scale_half_Ab_R1_central 1
scale_kd_Ab_T1_central 1
scale_half_Ab_central 1
mL_per_L 1000
```

```
uL_per_L 1000000
mg_per_g 1000
um2_per_dm2 10000000000
nmol_per_mol 1000000000.0
SECONDS_PER_DAY 86400
SECONDS_PER_HOUR 3600
SECONDS_PER_MINUTE 60
pi 3.141592653589793

% relationships
ugml_per_nM == mw_1 / 1000000
kabs == log(2) / (abs_half * SECONDS_PER_DAY)
kclear_Ab == log(2) / (el_half_1 * SECONDS_PER_DAY)
area_per_cell_um2 == 4 * pi * (cell_diameter_um / 2) ^ 2
area_per_cell == area_per_cell_um2 / um2_per_dm2
kclear_Ab_central == kclear_Ab / scale_half_Ab_central
cell_density_central == cell_density_mL_central * mL_per_L
total_cells_central == cell_density_central * volume_central
area_central == total_cells_central * area_per_cell
kon1_Ab_T1 == kon
kon2_Ab_T1 == floor(drug_valency_1 / 2) * kon
koff_Ab_T1 == mab_kd_1 * kon
kon_L1_R1 == kon
koff_L1_R1 == lig_rec_kd_1 * kon
kclear_R1 == log(2) / (rec_half_1 * SECONDS_PER_MINUTE)
kclear_L1 == log(2) / (lig_half_1 * SECONDS_PER_MINUTE)
kclear_S1 == log(2) / (shed_half_1 * SECONDS_PER_MINUTE)
kclear_L1_R1 == kclear_R1
ksynth_L1 == kon_L1_R1 * L1_central_0 * R1_central_0 / volume_central
- koff_L1_R1 * L1_R1_central_0 + kclear_L1 * L1_central_0
kshed_R1 == kclear_S1 * S1_central_0 / R1_central_0
kclear_Ab_R1_central == kclear_R1 / scale_half_Ab_R1_central
koff_Ab_T1_central == koff_Ab_T1 * scale_kd_Ab_T1_central
total_R1_central == rec_css_1_central * volume_central
L1_central_0 == lig_css_1_central * volume_central
S1_central_0 == shed_css_1_central * volume_central
L1_R1_central_0 == (kon_L1_R1 * L1_central_0 * total_R1_central /
volume_central) / (kon_L1_R1 * L1_central_0 / volume_central +
koff_L1_R1 + kclear_R1)
R1_central_0 == total_R1_central - L1_R1_central_0
activity_1_central_0 == L1_R1_central_0
ksynth_R1_central == kon_L1_R1 * L1_central_0 * R1_central_0 /
volume_central - koff_L1_R1 * L1_R1_central_0 + kclear_R1 *
```

```
R1_central_0 + kshed_R1 * R1_central_0

% compartments
depot 0 1
central 3 volume_central
central_membrane 2 area_central

% states depot
Ab_depot

% states central
L1_central L1_central_0

% states central_membrane
R1_central R1_central_0
L1_R1_central L1_R1_central_0

% states central
S1_central S1_central_0
Ab_00_central
Ab_0L_central
Ab_L0_central
Ab_LL_central

% routes
IV ([])@([]) Ab_00_central=1
SC ([])@([]) Ab_depot=1

% reactions
Ab_depot -> Ab_00_central, kabs
0 -> L1_central, ksynth_L1
L1_central -> 0, kclear_L1
0 -> R1_central, ksynth_R1_central
R1_central -> 0, kclear_R1
L1_R1_central -> 0, kclear_L1_R1
R1_central -> S1_central, kshed_R1
S1_central -> 0, kclear_S1
L1_central + R1_central -> L1_R1_central, kon_L1_R1
L1_R1_central -> L1_central + R1_central, koff_L1_R1
Ab_00_central -> 0, kclear_Ab_central
L1_central + Ab_00_central -> Ab_0L_central, kon2_Ab_T1
Ab_0L_central -> L1_central + Ab_00_central, koff_Ab_T1_central
Ab_0L_central -> 0, kclear_Ab_central
```

```
L1_central + Ab_00_central -> Ab_L0_central, kon1_Ab_T1
Ab_L0_central -> L1_central + Ab_00_central, koff_Ab_T1_central
Ab_L0_central -> 0, kclear_Ab_central
L1_central + Ab_0L_central -> Ab_LL_central, kon1_Ab_T1
Ab_LL_central -> L1_central + Ab_0L_central, koff_Ab_T1_central
L1_central + Ab_L0_central -> Ab_LL_central, kon2_Ab_T1
Ab_LL_central -> L1_central + Ab_L0_central, koff_Ab_T1_central
Ab_LL_central -> 0, kclear_Ab_central

% outputs
free_1_central L1_central + L1_R1_central
active_1_central L1_R1_central
engaged_1_central Ab_0L_central + Ab_L0_central + Ab_LL_central * 2
total_1_central R1_central + L1_R1_central
activity_1_central L1_R1_central
free_drug_central Ab_00_central / volume_central
soluble_drug_central (Ab_00_central + Ab_0L_central + Ab_L0_central +
Ab_LL_central) / volume_central
```
