# Supplementary material for: Early Feasibility Assessment: A Method for Accurately Predicting Biotherapeutic Dosing to Inform Early Drug Discovery Decisions
Source: Front Pharmacol. 2022 Jun 8;13:864768. doi: 10.3389/fphar.2022.864768 (PMC9214263; doi:10.3389/fphar.2022.864768)
Supplement: Supplementary file 3 [file DataSheet2.ZIP › Model run files_json and reports/four_compartment_anti_receptor.pdf]

```
# Title: Four Compartment Anti Receptor Model
# File: four_compartment_anti_receptor
# Author: apgar@appliedbiomath.com
# Website: https://www.appliedbiomath.com/assess
#
# (C)  Applied BioMath, LLC, 2022
# All rights reserved
#
# Notice: Applied BioMath, LLC ("Applied BioMath") retains and
# reserves all rights, title, and interest in and to all Applied
# BioMath-developed methodologies, technologies, and techniques
# embodied by this model file. Applied BioMath's delivery or other
# providing of access to this file  shall not be construed as
# conveying ownership or licensing of any rights, title, or interest
# in or to any such  methodologies, technologies, or techniques.
#

% parameters
kon 0.001
interval 14
dose 100
mab_kd_1 0.1
dose_count 7
mw_1 150000
el_half_1 28
abs_half 2.5
BW 70
volume_central 2.5
volume_peripheral 12.8
volume_disease 0.1
volume_tox 0.1
Tdist_Ab_hr_peripheral 30
Tdist_Ab_hr_disease 30
Tdist_Ab_hr_tox 30
Pdist_Ab_peripheral 0.190625
Pdist_Ab_disease 0.3
Pdist_Ab_tox 0.3
drug_valency_1 2
lig_half_1 30.0
rec_half_1 60
shed_half_1 30.0
lig_rec_kd_1 1
```

```
lig_css_1_central 0.05
lig_css_1_peripheral 0.05
lig_css_1_disease 0.05
lig_css_1_tox 0.05
rec_css_1_central 0.016605390671738465
rec_css_1_peripheral 0.016605390671738465
rec_css_1_disease 0.016605390671738465
rec_css_1_tox 0.016605390671738465
shed_css_1_central 0
shed_css_1_peripheral 0
shed_css_1_disease 0
shed_css_1_tox 0
Tdist_L1_hr_peripheral 30
Tdist_L1_hr_disease 30
Tdist_L1_hr_tox 30
Tdist_S1_hr_peripheral 30
Tdist_S1_hr_disease 30
Tdist_S1_hr_tox 30
cell_diameter_um 10
cell_density_mL_central 1000000
cell_density_mL_peripheral 1000000
cell_density_mL_disease 1000000
cell_density_mL_tox 1000000
scale_half_Ab_R1_central 1
scale_half_Ab_R1_peripheral 1
scale_half_Ab_R1_disease 1
scale_half_Ab_R1_tox 1
scale_kd_Ab_T1_central 1
scale_kd_Ab_T1_peripheral 1
scale_kd_Ab_T1_disease 1
scale_kd_Ab_T1_tox 1
scale_half_Ab_central 1
scale_half_Ab_peripheral 1
scale_half_Ab_disease 1
scale_half_Ab_tox 1
mL_per_L 1000
uL_per_L 1000000
mg_per_g 1000
um2_per_dm2 10000000000
nmol_per_mol 1000000000.0
SECONDS_PER_DAY 86400
SECONDS_PER_HOUR 3600
SECONDS_PER_MINUTE 60
```

```
pi 3.141592653589793


% relationships
ugml_per_nM == mw_1 / 1000000
kabs == log(2) / (abs_half * SECONDS_PER_DAY)
kclear_Ab == log(2) / (el_half_1 * SECONDS_PER_DAY)
area_per_cell_um2 == 4 * pi * (cell_diameter_um / 2) ^ 2
area_per_cell == area_per_cell_um2 / um2_per_dm2
Tdist_Ab_peripheral == Tdist_Ab_hr_peripheral * SECONDS_PER_HOUR
kout_Ab_peripheral == (log(2) / Tdist_Ab_peripheral) *
Pdist_Ab_peripheral / (Pdist_Ab_peripheral + (volume_central /
volume_peripheral))
kin_Ab_peripheral == (log(2) / Tdist_Ab_peripheral) / (1 +
Pdist_Ab_peripheral * volume_peripheral / volume_central)
Tdist_Ab_disease == Tdist_Ab_hr_disease * SECONDS_PER_HOUR
kout_Ab_disease == (log(2) / Tdist_Ab_disease) * Pdist_Ab_disease /
(Pdist_Ab_disease + (volume_central / volume_disease))
kin_Ab_disease == (log(2) / Tdist_Ab_disease) / (1 + Pdist_Ab_disease
* volume_disease / volume_central)
Tdist_Ab_tox == Tdist_Ab_hr_tox * SECONDS_PER_HOUR
kout_Ab_tox == (log(2) / Tdist_Ab_tox) * Pdist_Ab_tox / (Pdist_Ab_tox
+ (volume_central / volume_tox))
kin_Ab_tox == (log(2) / Tdist_Ab_tox) / (1 + Pdist_Ab_tox *
volume_tox / volume_central)
kclear_Ab_central == kclear_Ab / scale_half_Ab_central
cell_density_central == cell_density_mL_central * mL_per_L
total_cells_central == cell_density_central * volume_central
area_central == total_cells_central * area_per_cell
kclear_Ab_peripheral == kclear_Ab / scale_half_Ab_peripheral
cell_density_peripheral == cell_density_mL_peripheral * mL_per_L
total_cells_peripheral == cell_density_peripheral * volume_peripheral
area_peripheral == total_cells_peripheral * area_per_cell
kclear_Ab_disease == kclear_Ab / scale_half_Ab_disease
cell_density_disease == cell_density_mL_disease * mL_per_L
total_cells_disease == cell_density_disease * volume_disease
area_disease == total_cells_disease * area_per_cell
kclear_Ab_tox == kclear_Ab / scale_half_Ab_tox
cell_density_tox == cell_density_mL_tox * mL_per_L
total_cells_tox == cell_density_tox * volume_tox
area_tox == total_cells_tox * area_per_cell
kon1_Ab_T1 == kon
kon2_Ab_T1 == floor(drug_valency_1 / 2) * kon
```

```
koff_Ab_T1 == mab_kd_1 * kon
kon_L1_R1 == kon
koff_L1_R1 == lig_rec_kd_1 * kon
kclear_R1 == log(2) / (rec_half_1 * SECONDS_PER_MINUTE)
kclear_L1 == log(2) / (lig_half_1 * SECONDS_PER_MINUTE)
kclear_S1 == log(2) / (shed_half_1 * SECONDS_PER_MINUTE)
kclear_L1_R1 == kclear_R1
ksynth_L1 == kon_L1_R1 * L1_central_0 * R1_central_0 / volume_central
- koff_L1_R1 * L1_R1_central_0 + kclear_L1 * L1_central_0
kshed_R1 == kclear_S1 * S1_central_0 / R1_central_0
kclear_Ab_R1_central == kclear_R1 / scale_half_Ab_R1_central
koff_Ab_T1_central == koff_Ab_T1 * scale_kd_Ab_T1_central
total_R1_central == rec_css_1_central * volume_central
L1_central_0 == lig_css_1_central * volume_central
S1_central_0 == shed_css_1_central * volume_central
L1_R1_central_0 == (kon_L1_R1 * L1_central_0 * total_R1_central /
volume_central) / (kon_L1_R1 * L1_central_0 / volume_central +
koff_L1_R1 + kclear_R1)
R1_central_0 == total_R1_central - L1_R1_central_0
activity_1_central_0 == L1_R1_central_0
ksynth_R1_central == kon_L1_R1 * L1_central_0 * R1_central_0 /
volume_central - koff_L1_R1 * L1_R1_central_0 + kclear_R1 *
R1_central_0 + kshed_R1 * R1_central_0
kclear_Ab_R1_peripheral == kclear_R1 / scale_half_Ab_R1_peripheral
koff_Ab_T1_peripheral == koff_Ab_T1 * scale_kd_Ab_T1_peripheral
total_R1_peripheral == rec_css_1_peripheral * volume_peripheral
L1_peripheral_0 == lig_css_1_peripheral * volume_peripheral
S1_peripheral_0 == shed_css_1_peripheral * volume_peripheral
L1_R1_peripheral_0 == (kon_L1_R1 * L1_peripheral_0 *
total_R1_peripheral / volume_peripheral) / (kon_L1_R1 *
L1_peripheral_0 / volume_peripheral + koff_L1_R1 + kclear_R1)
R1_peripheral_0 == total_R1_peripheral - L1_R1_peripheral_0
activity_1_peripheral_0 == L1_R1_peripheral_0
ksynth_R1_peripheral == kon_L1_R1 * L1_peripheral_0 * R1_peripheral_0
/ volume_peripheral - koff_L1_R1 * L1_R1_peripheral_0 + kclear_R1 *
R1_peripheral_0
Tdist_L1_peripheral == Tdist_L1_hr_peripheral * SECONDS_PER_HOUR
Pdist_L1_peripheral == lig_css_1_peripheral / lig_css_1_central
Q_L1_peripheral == log(2) / Tdist_L1_peripheral
kout_L1_peripheral == Q_L1_peripheral * Pdist_L1_peripheral /
(Pdist_L1_peripheral + volume_central / volume_peripheral)
kin_L1_peripheral == Q_L1_peripheral / (1 + Pdist_L1_peripheral *
volume_peripheral / volume_central)
```

```
Tdist_S1_peripheral == Tdist_S1_hr_peripheral * SECONDS_PER_HOUR
Pdist_S1_peripheral == shed_css_1_peripheral / (shed_css_1_central +
1e-16)
Q_S1_peripheral == log(2) / Tdist_S1_peripheral
kout_S1_peripheral == Q_S1_peripheral * Pdist_S1_peripheral /
(Pdist_S1_peripheral + volume_central / volume_peripheral)
kin_S1_peripheral == Q_S1_peripheral / (1 + Pdist_S1_peripheral *
volume_peripheral / volume_central)
kclear_Ab_R1_disease == kclear_R1 / scale_half_Ab_R1_disease
koff_Ab_T1_disease == koff_Ab_T1 * scale_kd_Ab_T1_disease
total_R1_disease == rec_css_1_disease * volume_disease
L1_disease_0 == lig_css_1_disease * volume_disease
S1_disease_0 == shed_css_1_disease * volume_disease
L1_R1_disease_0 == (kon_L1_R1 * L1_disease_0 * total_R1_disease /
volume_disease) / (kon_L1_R1 * L1_disease_0 / volume_disease +
koff_L1_R1 + kclear_R1)
R1_disease_0 == total_R1_disease - L1_R1_disease_0
activity_1_disease_0 == L1_R1_disease_0
ksynth_R1_disease == kon_L1_R1 * L1_disease_0 * R1_disease_0 /
volume_disease - koff_L1_R1 * L1_R1_disease_0 + kclear_R1 *
R1_disease_0
Tdist_L1_disease == Tdist_L1_hr_disease * SECONDS_PER_HOUR
Pdist_L1_disease == lig_css_1_disease / lig_css_1_central
Q_L1_disease == log(2) / Tdist_L1_disease
kout_L1_disease == Q_L1_disease * Pdist_L1_disease /
(Pdist_L1_disease + volume_central / volume_disease)
kin_L1_disease == Q_L1_disease / (1 + Pdist_L1_disease *
volume_disease / volume_central)
Tdist_S1_disease == Tdist_S1_hr_disease * SECONDS_PER_HOUR
Pdist_S1_disease == shed_css_1_disease / (shed_css_1_central + 1e-16)
Q_S1_disease == log(2) / Tdist_S1_disease
kout_S1_disease == Q_S1_disease * Pdist_S1_disease /
(Pdist_S1_disease + volume_central / volume_disease)
kin_S1_disease == Q_S1_disease / (1 + Pdist_S1_disease *
volume_disease / volume_central)
kclear_Ab_R1_tox == kclear_R1 / scale_half_Ab_R1_tox
koff_Ab_T1_tox == koff_Ab_T1 * scale_kd_Ab_T1_tox
total_R1_tox == rec_css_1_tox * volume_tox
L1_tox_0 == lig_css_1_tox * volume_tox
S1_tox_0 == shed_css_1_tox * volume_tox
L1_R1_tox_0 == (kon_L1_R1 * L1_tox_0 * total_R1_tox / volume_tox) /
(kon_L1_R1 * L1_tox_0 / volume_tox + koff_L1_R1 + kclear_R1)
R1_tox_0 == total_R1_tox - L1_R1_tox_0
```

```
activity_1_tox_0 == L1_R1_tox_0
ksynth_R1_tox == kon_L1_R1 * L1_tox_0 * R1_tox_0 / volume_tox -
koff_L1_R1 * L1_R1_tox_0 + kclear_R1 * R1_tox_0
Tdist_L1_tox == Tdist_L1_hr_tox * SECONDS_PER_HOUR
Pdist_L1_tox == lig_css_1_tox / lig_css_1_central
Q_L1_tox == log(2) / Tdist_L1_tox
kout_L1_tox == Q_L1_tox * Pdist_L1_tox / (Pdist_L1_tox +
volume_central / volume_tox)
kin_L1_tox == Q_L1_tox / (1 + Pdist_L1_tox * volume_tox /
volume_central)
Tdist_S1_tox == Tdist_S1_hr_tox * SECONDS_PER_HOUR
Pdist_S1_tox == shed_css_1_tox / (shed_css_1_central + 1e-16)
Q_S1_tox == log(2) / Tdist_S1_tox
kout_S1_tox == Q_S1_tox * Pdist_S1_tox / (Pdist_S1_tox +
volume_central / volume_tox)
kin_S1_tox == Q_S1_tox / (1 + Pdist_S1_tox * volume_tox /
volume_central)

% compartments
depot 0 1
central 3 volume_central
central_membrane 2 area_central
peripheral 3 volume_peripheral
peripheral_membrane 2 area_peripheral
disease 3 volume_disease
disease_membrane 2 area_disease
tox 3 volume_tox
tox_membrane 2 area_tox

% states depot
Ab_depot

% states central
L1_central L1_central_0

% states central_membrane
R1_central R1_central_0
L1_R1_central L1_R1_central_0

% states central
S1_central S1_central_0
Ab_00_central
```

```
% states central_membrane
Ab_0R_central

% states central
Ab_0S_central

% states central_membrane
Ab_R0_central
Ab_RR_central
Ab_RS_central

% states central
Ab_S0_central

% states central_membrane
Ab_SR_central

% states central
Ab_SS_central

% states peripheral
L1_peripheral L1_peripheral_0

% states peripheral_membrane
R1_peripheral R1_peripheral_0
L1_R1_peripheral L1_R1_peripheral_0

% states peripheral
S1_peripheral S1_peripheral_0
Ab_00_peripheral

% states peripheral_membrane
Ab_0R_peripheral

% states peripheral
Ab_0S_peripheral

% states peripheral_membrane
Ab_R0_peripheral
Ab_RR_peripheral
Ab_RS_peripheral

% states peripheral
```

```
Ab_S0_peripheral

% states peripheral_membrane
Ab_SR_peripheral

% states peripheral
Ab_SS_peripheral

% states disease
L1_disease L1_disease_0

% states disease_membrane
R1_disease R1_disease_0
L1_R1_disease L1_R1_disease_0

% states disease
S1_disease S1_disease_0
Ab_00_disease

% states disease_membrane
Ab_0R_disease

% states disease
Ab_0S_disease

% states disease_membrane
Ab_R0_disease
Ab_RR_disease
Ab_RS_disease

% states disease
Ab_S0_disease

% states disease_membrane
Ab_SR_disease

% states disease
Ab_SS_disease

% states tox
L1_tox L1_tox_0

% states tox_membrane
```

```
R1_tox R1_tox_0
L1_R1_tox L1_R1_tox_0

% states tox
S1_tox S1_tox_0
Ab_00_tox

% states tox_membrane
Ab_0R_tox

% states tox
Ab_0S_tox

% states tox_membrane
Ab_R0_tox
Ab_RR_tox
Ab_RS_tox

% states tox
Ab_S0_tox

% states tox_membrane
Ab_SR_tox

% states tox
Ab_SS_tox

% routes
IV ([])@([]) Ab_00_central=1
SC ([])@([]) Ab_depot=1

% reactions
Ab_depot -> Ab_00_central, kabs
0 -> L1_central, ksynth_L1
L1_central -> 0, kclear_L1
0 -> R1_central, ksynth_R1_central
R1_central -> 0, kclear_R1
L1_R1_central -> 0, kclear_L1_R1
R1_central -> S1_central, kshed_R1
S1_central -> 0, kclear_S1
L1_central + R1_central -> L1_R1_central, kon_L1_R1
L1_R1_central -> L1_central + R1_central, koff_L1_R1
Ab_00_central -> 0, kclear_Ab_central
```

```
R1_central + Ab_00_central -> Ab_0R_central, kon2_Ab_T1
Ab_0R_central -> R1_central + Ab_00_central, koff_Ab_T1_central
Ab_0R_central -> 0, kclear_Ab_R1_central
S1_central + Ab_00_central -> Ab_0S_central, kon2_Ab_T1
Ab_0S_central -> S1_central + Ab_00_central, koff_Ab_T1_central
Ab_0S_central -> 0, kclear_Ab_central
R1_central + Ab_00_central -> Ab_R0_central, kon1_Ab_T1
Ab_R0_central -> R1_central + Ab_00_central, koff_Ab_T1_central
Ab_R0_central -> 0, kclear_Ab_R1_central
R1_central + Ab_0R_central -> Ab_RR_central, kon1_Ab_T1
Ab_RR_central -> R1_central + Ab_0R_central, koff_Ab_T1_central
R1_central + Ab_R0_central -> Ab_RR_central, kon2_Ab_T1
Ab_RR_central -> R1_central + Ab_R0_central, koff_Ab_T1_central
Ab_RR_central -> R1_central, kclear_Ab_R1_central
Ab_RR_central -> R1_central, kclear_Ab_R1_central
R1_central + Ab_0S_central -> Ab_RS_central, kon1_Ab_T1
Ab_RS_central -> R1_central + Ab_0S_central, koff_Ab_T1_central
S1_central + Ab_R0_central -> Ab_RS_central, kon2_Ab_T1
Ab_RS_central -> S1_central + Ab_R0_central, koff_Ab_T1_central
Ab_RS_central -> 0, kclear_Ab_R1_central
S1_central + Ab_00_central -> Ab_S0_central, kon1_Ab_T1
Ab_S0_central -> S1_central + Ab_00_central, koff_Ab_T1_central
Ab_S0_central -> 0, kclear_Ab_central
S1_central + Ab_0R_central -> Ab_SR_central, kon1_Ab_T1
Ab_SR_central -> S1_central + Ab_0R_central, koff_Ab_T1_central
R1_central + Ab_S0_central -> Ab_SR_central, kon2_Ab_T1
Ab_SR_central -> R1_central + Ab_S0_central, koff_Ab_T1_central
Ab_SR_central -> 0, kclear_Ab_R1_central
S1_central + Ab_0S_central -> Ab_SS_central, kon1_Ab_T1
Ab_SS_central -> S1_central + Ab_0S_central, koff_Ab_T1_central
S1_central + Ab_S0_central -> Ab_SS_central, kon2_Ab_T1
Ab_SS_central -> S1_central + Ab_S0_central, koff_Ab_T1_central
Ab_SS_central -> 0, kclear_Ab_central
0 -> R1_peripheral, ksynth_R1_peripheral
R1_peripheral -> 0, kclear_R1
L1_R1_peripheral -> L1_peripheral, kclear_L1_R1
L1_peripheral + R1_peripheral -> L1_R1_peripheral, kon_L1_R1
L1_R1_peripheral -> L1_peripheral + R1_peripheral, koff_L1_R1
L1_central -> L1_peripheral, kout_L1_peripheral
L1_peripheral -> L1_central, kin_L1_peripheral
S1_central -> S1_peripheral, kout_S1_peripheral
S1_peripheral -> S1_central, kin_S1_peripheral
Ab_00_peripheral -> 0, kclear_Ab_peripheral
```

```
Ab_00_central -> Ab_00_peripheral, kout_Ab_peripheral
Ab_00_peripheral -> Ab_00_central, kin_Ab_peripheral
R1_peripheral + Ab_00_peripheral -> Ab_0R_peripheral, kon2_Ab_T1
Ab_0R_peripheral -> R1_peripheral + Ab_00_peripheral,
koff_Ab_T1_peripheral
Ab_0R_peripheral -> 0, kclear_Ab_R1_peripheral
S1_peripheral + Ab_00_peripheral -> Ab_0S_peripheral, kon2_Ab_T1
Ab_0S_peripheral -> S1_peripheral + Ab_00_peripheral,
koff_Ab_T1_peripheral
Ab_0S_peripheral -> 0, kclear_Ab_peripheral
Ab_0S_central -> Ab_0S_peripheral, kout_Ab_peripheral
Ab_0S_peripheral -> Ab_0S_central, kin_Ab_peripheral
R1_peripheral + Ab_00_peripheral -> Ab_R0_peripheral, kon1_Ab_T1
Ab_R0_peripheral -> R1_peripheral + Ab_00_peripheral,
koff_Ab_T1_peripheral
Ab_R0_peripheral -> 0, kclear_Ab_R1_peripheral
R1_peripheral + Ab_0R_peripheral -> Ab_RR_peripheral, kon1_Ab_T1
Ab_RR_peripheral -> R1_peripheral + Ab_0R_peripheral,
koff_Ab_T1_peripheral
R1_peripheral + Ab_R0_peripheral -> Ab_RR_peripheral, kon2_Ab_T1
Ab_RR_peripheral -> R1_peripheral + Ab_R0_peripheral,
koff_Ab_T1_peripheral
Ab_RR_peripheral -> R1_peripheral, kclear_Ab_R1_peripheral
Ab_RR_peripheral -> R1_peripheral, kclear_Ab_R1_peripheral
R1_peripheral + Ab_0S_peripheral -> Ab_RS_peripheral, kon1_Ab_T1
Ab_RS_peripheral -> R1_peripheral + Ab_0S_peripheral,
koff_Ab_T1_peripheral
S1_peripheral + Ab_R0_peripheral -> Ab_RS_peripheral, kon2_Ab_T1
Ab_RS_peripheral -> S1_peripheral + Ab_R0_peripheral,
koff_Ab_T1_peripheral
Ab_RS_peripheral -> 0, kclear_Ab_R1_peripheral
S1_peripheral + Ab_00_peripheral -> Ab_S0_peripheral, kon1_Ab_T1
Ab_S0_peripheral -> S1_peripheral + Ab_00_peripheral,
koff_Ab_T1_peripheral
Ab_S0_peripheral -> 0, kclear_Ab_peripheral
Ab_S0_central -> Ab_S0_peripheral, kout_Ab_peripheral
Ab_S0_peripheral -> Ab_S0_central, kin_Ab_peripheral
S1_peripheral + Ab_0R_peripheral -> Ab_SR_peripheral, kon1_Ab_T1
Ab_SR_peripheral -> S1_peripheral + Ab_0R_peripheral,
koff_Ab_T1_peripheral
R1_peripheral + Ab_S0_peripheral -> Ab_SR_peripheral, kon2_Ab_T1
Ab_SR_peripheral -> R1_peripheral + Ab_S0_peripheral,
koff_Ab_T1_peripheral
```

```
Ab_SR_peripheral -> 0, kclear_Ab_R1_peripheral
S1_peripheral + Ab_0S_peripheral -> Ab_SS_peripheral, kon1_Ab_T1
Ab_SS_peripheral -> S1_peripheral + Ab_0S_peripheral,
koff_Ab_T1_peripheral
S1_peripheral + Ab_S0_peripheral -> Ab_SS_peripheral, kon2_Ab_T1
Ab_SS_peripheral -> S1_peripheral + Ab_S0_peripheral,
koff_Ab_T1_peripheral
Ab_SS_peripheral -> 0, kclear_Ab_peripheral
Ab_SS_central -> Ab_SS_peripheral, kout_Ab_peripheral
Ab_SS_peripheral -> Ab_SS_central, kin_Ab_peripheral
0 -> R1_disease, ksynth_R1_disease
R1_disease -> 0, kclear_R1
L1_R1_disease -> L1_disease, kclear_L1_R1
L1_disease + R1_disease -> L1_R1_disease, kon_L1_R1
L1_R1_disease -> L1_disease + R1_disease, koff_L1_R1
L1_central -> L1_disease, kout_L1_disease
L1_disease -> L1_central, kin_L1_disease
S1_central -> S1_disease, kout_S1_disease
S1_disease -> S1_central, kin_S1_disease
Ab_00_disease -> 0, kclear_Ab_disease
Ab_00_central -> Ab_00_disease, kout_Ab_disease
Ab_00_disease -> Ab_00_central, kin_Ab_disease
R1_disease + Ab_00_disease -> Ab_0R_disease, kon2_Ab_T1
Ab_0R_disease -> R1_disease + Ab_00_disease, koff_Ab_T1_disease
Ab_0R_disease -> 0, kclear_Ab_R1_disease
S1_disease + Ab_00_disease -> Ab_0S_disease, kon2_Ab_T1
Ab_0S_disease -> S1_disease + Ab_00_disease, koff_Ab_T1_disease
Ab_0S_disease -> 0, kclear_Ab_disease
Ab_0S_central -> Ab_0S_disease, kout_Ab_disease
Ab_0S_disease -> Ab_0S_central, kin_Ab_disease
R1_disease + Ab_00_disease -> Ab_R0_disease, kon1_Ab_T1
Ab_R0_disease -> R1_disease + Ab_00_disease, koff_Ab_T1_disease
Ab_R0_disease -> 0, kclear_Ab_R1_disease
R1_disease + Ab_0R_disease -> Ab_RR_disease, kon1_Ab_T1
Ab_RR_disease -> R1_disease + Ab_0R_disease, koff_Ab_T1_disease
R1_disease + Ab_R0_disease -> Ab_RR_disease, kon2_Ab_T1
Ab_RR_disease -> R1_disease + Ab_R0_disease, koff_Ab_T1_disease
Ab_RR_disease -> R1_disease, kclear_Ab_R1_disease
Ab_RR_disease -> R1_disease, kclear_Ab_R1_disease
R1_disease + Ab_0S_disease -> Ab_RS_disease, kon1_Ab_T1
Ab_RS_disease -> R1_disease + Ab_0S_disease, koff_Ab_T1_disease
S1_disease + Ab_R0_disease -> Ab_RS_disease, kon2_Ab_T1
Ab_RS_disease -> S1_disease + Ab_R0_disease, koff_Ab_T1_disease
```

```
Ab_RS_disease -> 0, kclear_Ab_R1_disease
S1_disease + Ab_00_disease -> Ab_S0_disease, kon1_Ab_T1
Ab_S0_disease -> S1_disease + Ab_00_disease, koff_Ab_T1_disease
Ab_S0_disease -> 0, kclear_Ab_disease
Ab_S0_central -> Ab_S0_disease, kout_Ab_disease
Ab_S0_disease -> Ab_S0_central, kin_Ab_disease
S1_disease + Ab_0R_disease -> Ab_SR_disease, kon1_Ab_T1
Ab_SR_disease -> S1_disease + Ab_0R_disease, koff_Ab_T1_disease
R1_disease + Ab_S0_disease -> Ab_SR_disease, kon2_Ab_T1
Ab_SR_disease -> R1_disease + Ab_S0_disease, koff_Ab_T1_disease
Ab_SR_disease -> 0, kclear_Ab_R1_disease
S1_disease + Ab_0S_disease -> Ab_SS_disease, kon1_Ab_T1
Ab_SS_disease -> S1_disease + Ab_0S_disease, koff_Ab_T1_disease
S1_disease + Ab_S0_disease -> Ab_SS_disease, kon2_Ab_T1
Ab_SS_disease -> S1_disease + Ab_S0_disease, koff_Ab_T1_disease
Ab_SS_disease -> 0, kclear_Ab_disease
Ab_SS_central -> Ab_SS_disease, kout_Ab_disease
Ab_SS_disease -> Ab_SS_central, kin_Ab_disease
0 -> R1_tox, ksynth_R1_tox
R1_tox -> 0, kclear_R1
L1_R1_tox -> L1_tox, kclear_L1_R1
L1_tox + R1_tox -> L1_R1_tox, kon_L1_R1
L1_R1_tox -> L1_tox + R1_tox, koff_L1_R1
L1_central -> L1_tox, kout_L1_tox
L1_tox -> L1_central, kin_L1_tox
S1_central -> S1_tox, kout_S1_tox
S1_tox -> S1_central, kin_S1_tox
Ab_00_tox -> 0, kclear_Ab_tox
Ab_00_central -> Ab_00_tox, kout_Ab_tox
Ab_00_tox -> Ab_00_central, kin_Ab_tox
R1_tox + Ab_00_tox -> Ab_0R_tox, kon2_Ab_T1
Ab_0R_tox -> R1_tox + Ab_00_tox, koff_Ab_T1_tox
Ab_0R_tox -> 0, kclear_Ab_R1_tox
S1_tox + Ab_00_tox -> Ab_0S_tox, kon2_Ab_T1
Ab_0S_tox -> S1_tox + Ab_00_tox, koff_Ab_T1_tox
Ab_0S_tox -> 0, kclear_Ab_tox
Ab_0S_central -> Ab_0S_tox, kout_Ab_tox
Ab_0S_tox -> Ab_0S_central, kin_Ab_tox
R1_tox + Ab_00_tox -> Ab_R0_tox, kon1_Ab_T1
Ab_R0_tox -> R1_tox + Ab_00_tox, koff_Ab_T1_tox
Ab_R0_tox -> 0, kclear_Ab_R1_tox
R1_tox + Ab_0R_tox -> Ab_RR_tox, kon1_Ab_T1
Ab_RR_tox -> R1_tox + Ab_0R_tox, koff_Ab_T1_tox
```

```
R1_tox + Ab_R0_tox -> Ab_RR_tox, kon2_Ab_T1
Ab_RR_tox -> R1_tox + Ab_R0_tox, koff_Ab_T1_tox
Ab_RR_tox -> R1_tox, kclear_Ab_R1_tox
Ab_RR_tox -> R1_tox, kclear_Ab_R1_tox
R1_tox + Ab_0S_tox -> Ab_RS_tox, kon1_Ab_T1
Ab_RS_tox -> R1_tox + Ab_0S_tox, koff_Ab_T1_tox
S1_tox + Ab_R0_tox -> Ab_RS_tox, kon2_Ab_T1
Ab_RS_tox -> S1_tox + Ab_R0_tox, koff_Ab_T1_tox
Ab_RS_tox -> 0, kclear_Ab_R1_tox
S1_tox + Ab_00_tox -> Ab_S0_tox, kon1_Ab_T1
Ab_S0_tox -> S1_tox + Ab_00_tox, koff_Ab_T1_tox
Ab_S0_tox -> 0, kclear_Ab_tox
Ab_S0_central -> Ab_S0_tox, kout_Ab_tox
Ab_S0_tox -> Ab_S0_central, kin_Ab_tox
S1_tox + Ab_0R_tox -> Ab_SR_tox, kon1_Ab_T1
Ab_SR_tox -> S1_tox + Ab_0R_tox, koff_Ab_T1_tox
R1_tox + Ab_S0_tox -> Ab_SR_tox, kon2_Ab_T1
Ab_SR_tox -> R1_tox + Ab_S0_tox, koff_Ab_T1_tox
Ab_SR_tox -> 0, kclear_Ab_R1_tox
S1_tox + Ab_0S_tox -> Ab_SS_tox, kon1_Ab_T1
Ab_SS_tox -> S1_tox + Ab_0S_tox, koff_Ab_T1_tox
S1_tox + Ab_S0_tox -> Ab_SS_tox, kon2_Ab_T1
Ab_SS_tox -> S1_tox + Ab_S0_tox, koff_Ab_T1_tox
Ab_SS_tox -> 0, kclear_Ab_tox
Ab_SS_central -> Ab_SS_tox, kout_Ab_tox
Ab_SS_tox -> Ab_SS_central, kin_Ab_tox

% outputs
free_1_central R1_central + L1_R1_central
active_1_central L1_R1_central + Ab_0R_central + Ab_R0_central +
Ab_RR_central * 2 + Ab_RS_central + Ab_SR_central
engaged_1_central Ab_0R_central + Ab_R0_central + Ab_RR_central * 2 +
Ab_RS_central + Ab_SR_central
total_1_central R1_central + L1_R1_central + Ab_0R_central +
Ab_R0_central + Ab_RR_central * 2 + Ab_RS_central + Ab_SR_central
activity_1_central L1_R1_central
free_1_peripheral R1_peripheral + L1_R1_peripheral
active_1_peripheral L1_R1_peripheral + Ab_0R_peripheral +
Ab_R0_peripheral + Ab_RR_peripheral * 2 + Ab_RS_peripheral +
Ab_SR_peripheral
engaged_1_peripheral Ab_0R_peripheral + Ab_R0_peripheral +
Ab_RR_peripheral * 2 + Ab_RS_peripheral + Ab_SR_peripheral
total_1_peripheral R1_peripheral + L1_R1_peripheral +
```

```
Ab_0R_peripheral + Ab_R0_peripheral + Ab_RR_peripheral * 2 +
Ab_RS_peripheral + Ab_SR_peripheral
activity_1_peripheral L1_R1_peripheral
free_1_disease R1_disease + L1_R1_disease
active_1_disease L1_R1_disease + Ab_0R_disease + Ab_R0_disease +
Ab_RR_disease * 2 + Ab_RS_disease + Ab_SR_disease
engaged_1_disease Ab_0R_disease + Ab_R0_disease + Ab_RR_disease * 2 +
Ab_RS_disease + Ab_SR_disease
total_1_disease R1_disease + L1_R1_disease + Ab_0R_disease +
Ab_R0_disease + Ab_RR_disease * 2 + Ab_RS_disease + Ab_SR_disease
activity_1_disease L1_R1_disease
free_1_tox R1_tox + L1_R1_tox
active_1_tox L1_R1_tox + Ab_0R_tox + Ab_R0_tox + Ab_RR_tox * 2 +
Ab_RS_tox + Ab_SR_tox
engaged_1_tox Ab_0R_tox + Ab_R0_tox + Ab_RR_tox * 2 + Ab_RS_tox +
Ab_SR_tox
total_1_tox R1_tox + L1_R1_tox + Ab_0R_tox + Ab_R0_tox + Ab_RR_tox *
2 + Ab_RS_tox + Ab_SR_tox
activity_1_tox L1_R1_tox
free_drug_central Ab_00_central / volume_central
soluble_drug_central (Ab_00_central + Ab_0S_central + Ab_S0_central +
Ab_SS_central) / volume_central
free_drug_peripheral Ab_00_peripheral / volume_peripheral
soluble_drug_peripheral (Ab_00_peripheral + Ab_0S_peripheral +
Ab_S0_peripheral + Ab_SS_peripheral) / volume_peripheral
free_drug_disease Ab_00_disease / volume_disease
soluble_drug_disease (Ab_00_disease + Ab_0S_disease + Ab_S0_disease +
Ab_SS_disease) / volume_disease
free_drug_tox Ab_00_tox / volume_tox
soluble_drug_tox (Ab_00_tox + Ab_0S_tox + Ab_S0_tox + Ab_SS_tox) /
volume_tox
```
