# Supplementary material for: Early Feasibility Assessment: A Method for Accurately Predicting Biotherapeutic Dosing to Inform Early Drug Discovery Decisions
Source: Front Pharmacol. 2022 Jun 8;13:864768. doi: 10.3389/fphar.2022.864768 (PMC9214263; doi:10.3389/fphar.2022.864768)
Supplement: Supplementary file 3 [file DataSheet2.ZIP › Model run files_json and reports/All_Anti-Ligand_CaseStudies.pdf]

revolutionizing drug invention

Anti-Ligand mAb Case Studies generated with Applied BioMath Assess™

Model: Monospecific Anti-Ligand

Date: 2022-03-10T21:55:07.349Z

Software Version: 2022.2.2

This report does not fall within the scope of the United States Food and Drug Administration Good Laboratory Practice or Good Clinical Practice Regulations.

1) Summary

The goal of this study is to determine the feasibility of a drug concept for a target by varying parameters of a Quantitative Systems Pharmacology (QSP) model. By performing simulations over a range of drug and target parameters, this study supports comparison of the effects of dose amount, dose frequency, route of administration, drug design criteria, and target characteristics. This assessment can aid in the prediction of difficulty of discovering and formulation a New Biological Entity that satisfies a Target Product Profile.

The analyses explore the model conditions under which one or more criteria are satisfied. Depending on the kind of analysis, the feasibility of a scenario is defined to be either:

- the dose that exactly satisfies the criteria targets,
- a range of a parameter that satisfies the criteria targets,
- or a region of a parameter grid that satisfies the criteria targets.

The model used in these analyses is: Monospecific Anti-Ligand - A biologic that binds to a soluble ligand target and prevents the ligand from binding to its cognate-receptor. The molecule can be mono- or bivalent. This is a one-compartment model and there is an option for +/- soluble receptor.

 © Applied BioMath LLC 2017-2021

Scenario Table

Description	Route	Interval (undefined)	Dose (mg)	Drug:Target KD (nM)	Molecular Weight (Da)	Biologic first order T 1/2 (days)	Volume (L)	Effective Valency (undefined)	Ligand T 1/2 (min)	Receptor T 1/2 (min)	L:R Affinity (nM)	Ligand CSS (nM)	Receptor CSS (nM)	Scan Parameter	Criterion	Result
Adalimumab	SC	14	-	0.008	148000	20	5	1	30	540	0.019	0.0000575	0.23	: Dose (mg) [1 - 2000]	Last Inhibition ≥ 90 %	Dose ≥ 39.4 mg
Infliximab	SC	60	-	0.0042	149000	14	5	1	30	540	0.019	0.0000575	0.23	: Dose (mg) [1 - 2000]	Last Inhibition ≥ 90 %	Dose ≥ 444 mg
Ustekinumab	SC	90	-	0.001	148600	21.6	5	2	90	120	0.01	0.00038	0.0122	: Dose (mg) [1 - 2000]	Last Inhibition ≥ 90 %	Dose ≥ 27.5 mg
Risankizumab, Q12W	SC	90	-	0.001	145610	27	5	2	90	120	1.6	0.174	0.88	: Dose (mg) [1 - 2000]	Last Inhibition ≥ 90 %	Dose ≥ 324 mg
Risankizumab, Q4W	SC	30	-	0.001	145610	27	5	2	90	120	1.6	0.174	0.88	: Dose (mg) [1 - 2000]	Last Inhibition ≥ 90 %	Dose ≥ 41.0 mg
Belimumab, Q1W SC	SC	7	-	0.274	147000	19.4	5	1	60	120	15	0.294	0.058	: Dose (mg) [1 - 2000]	Last Inhibition ≥ 90 %	Dose ≥ 252 mg
Belimumab, Q1W SC	IV	30	-	0.274	147000	19.4	5	1	60	120	15	0.294	0.058	: Dose (mg) [1 - 2000]	Last Inhibition ≥ 90 %	Dose ≥ 1.90e+3 mg
Omalizumab	SC	14	-	0.02	149000	26	5	2	2880	15	0.12	8	0.78	: Dose (mg) [1 - 2000]	Last Inhibition ≥ 90 %	Dose ≥ 330 mg

© Applied BioMath LLC 2017-2021

2) Results

2.0) Scenario: Adalimumab

One dimensional scan over parameter, Dose, between 1 and 2000 using log spacing.

2.0.1) Model Parameters

Table 2.0.1

Symbol	Parameter ID	Value	Unit
τ	interval	14	-
D	dose	100	mg
$K_{D,\mathrm{L}}$	mab_kd_1	0.008	nM
N_{doses}	dose_count	7	-
MW	mw_1	148000	Da
$t_{1/2}$	el_half_1	20	days
$t_{1/2,\mathrm{a}}$	abs_half	2.5	days
BW	BW	70	kg
V	volume_central	5	L
$\mathrm{Valency}$	drug_valency_1	1	-
$t_{1/2,\mathrm{L}}$	lig_half_1	30	min
$t_{1/2,\mathrm{R}}$	rec_half_1	540	min
$t_{1/2,\mathrm{sR}}$	shed_half_1	0.5	hr
$K_{D,\mathrm{L:R}}$	lig_rec_kd_1	0.019	nM
$C_{SS,\mathrm{L}}$	lig_css_1_central	0.0000575	nM
$C_{SS,\mathrm{R}}$	rec_css_1_central	0.23	nM
$C_{SS,\mathrm{sR}}$	shed_css_1_central	0	nM
$\mathrm{Density}_{\mathrm{cells}}$	cell_density_mL_central	1000000	#/mL

Parameter ID	Value
Scan Parameter 1	Dose
Lower Limit 1	1
Upper Limit 1	2000
N1	11
Scale 1	log

 © Applied BioMath LLC 2017-2021

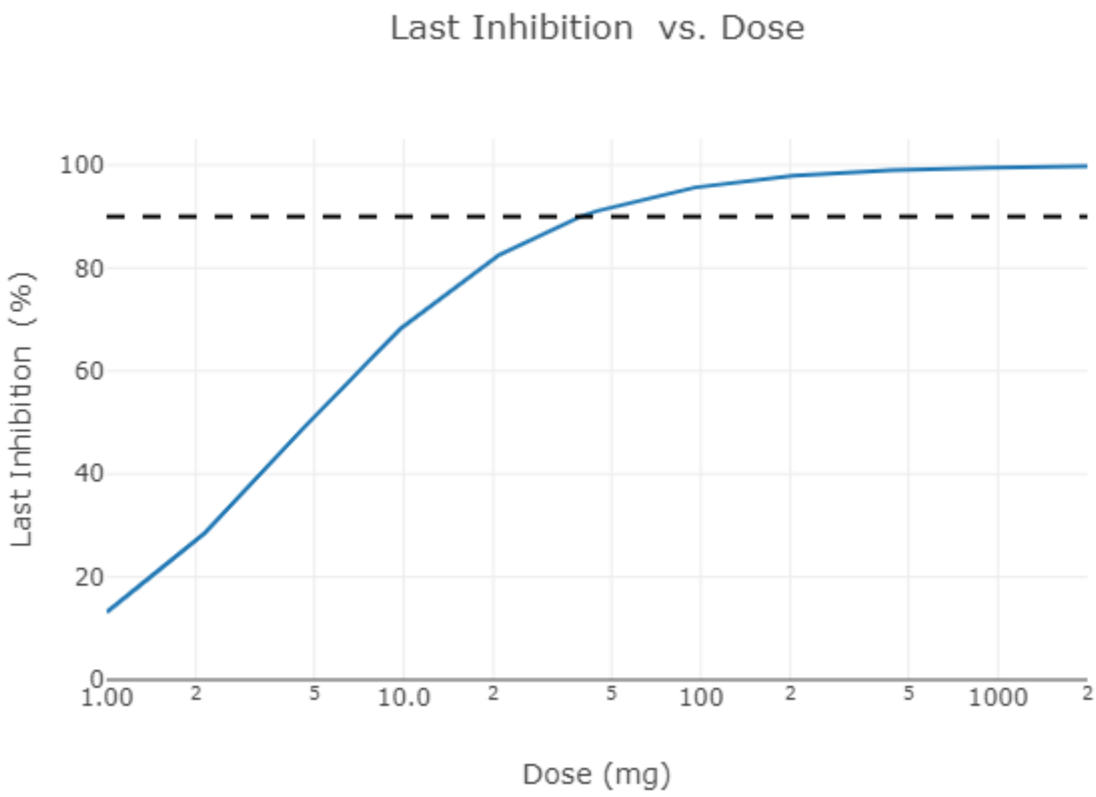

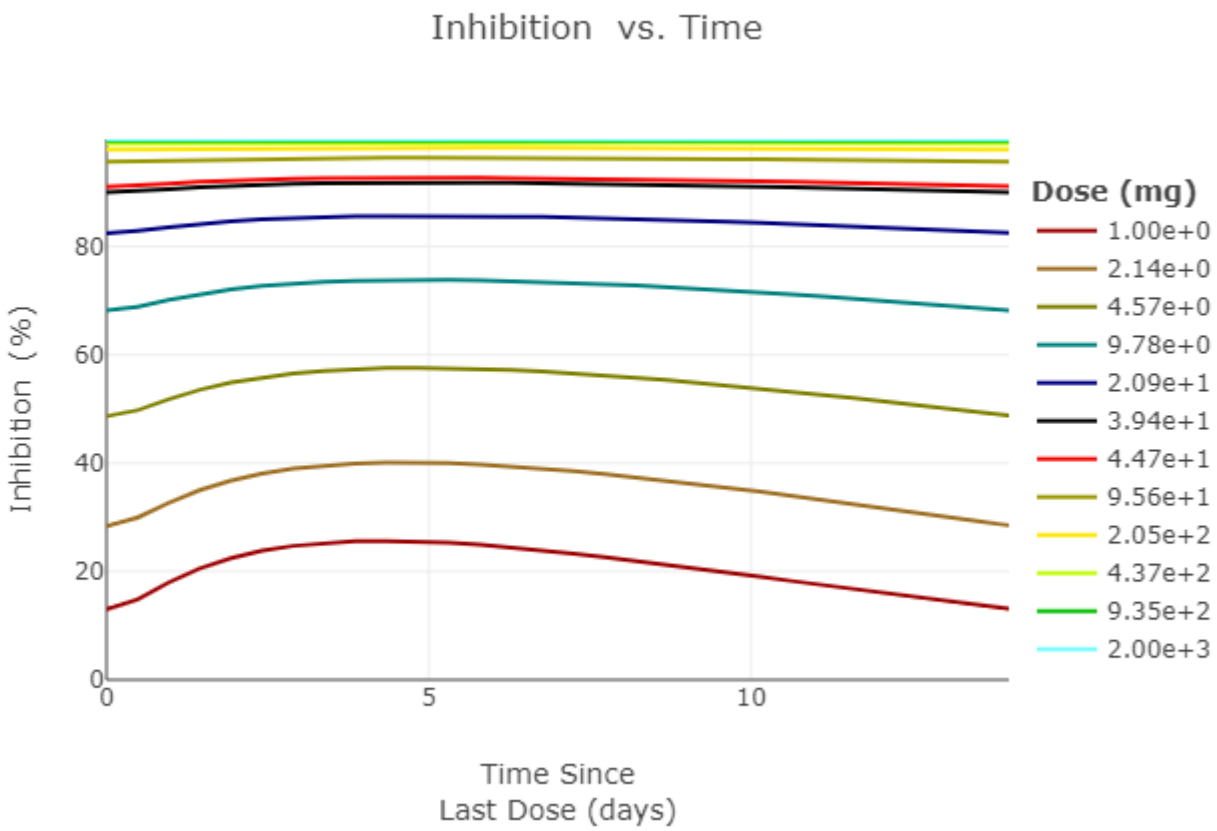

 © Applied BioMath LLC 2017-2021

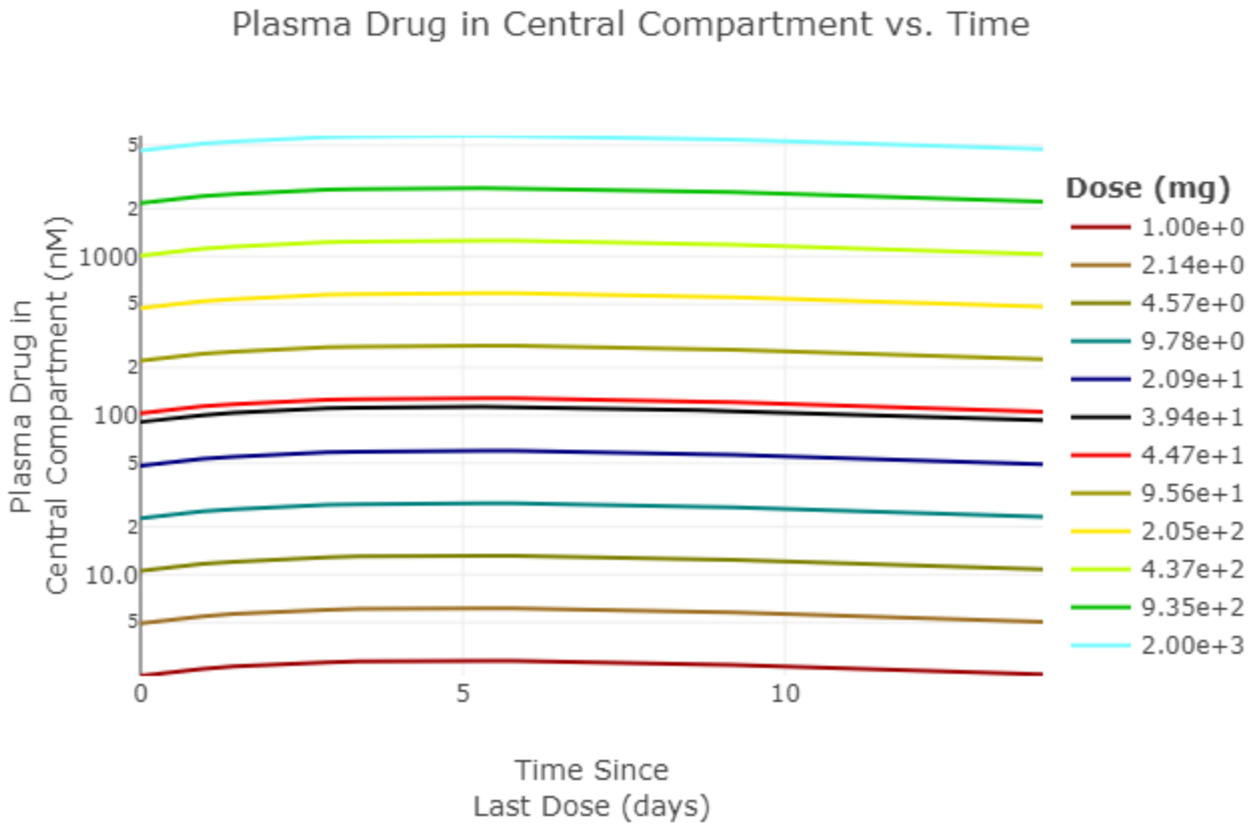

2.1) Scenario: Infliximab

One dimensional scan over parameter, Dose, between 1 and 2000 using log spacing.

2.1.1) Model Parameters

Table 2.1.1

Symbol	Parameter ID	Value	Unit
τ	interval	60	-
D	dose	100	mg
$K_{D,\mathrm{L}}$	mab_kd_1	0.0042	nM
N_{doses}	dose_count	7	-
MW	mw_1	149000	Da
$t_{1/2}$	el_half_1	14	days
$t_{1/2,\mathrm{a}}$	abs_half	2.5	days
BW	BW	70	kg
V	volume_central	5	L
Valency	drug_valency_1	1	-
$t_{1/2,\mathrm{L}}$	lig_half_1	30	min
$t_{1/2,\mathrm{R}}$	rec_half_1	540	min

$t_{1/2,\mathrm{sR}}$	shed_half_1	0.5	hr
$K_{D,\mathrm{L:R}}$	lig_rec_kd_1	0.019	nM
$C_{SS,\mathrm{L}}$	lig_css_1_central	0.0000575	nM
$C_{SS,\mathrm{R}}$	rec_css_1_central	0.23	nM
$C_{SS,\mathrm{sR}}$	shed_css_1_central	0	nM
$\mathrm{Density}_{\mathrm{cells}}$	cell_density_mL_central	1000000	#/mL

Parameter ID	Value
Scan Parameter 1	Dose
Lower Limit 1	1
Upper Limit 1	2000
N1	11
Scale 1	log

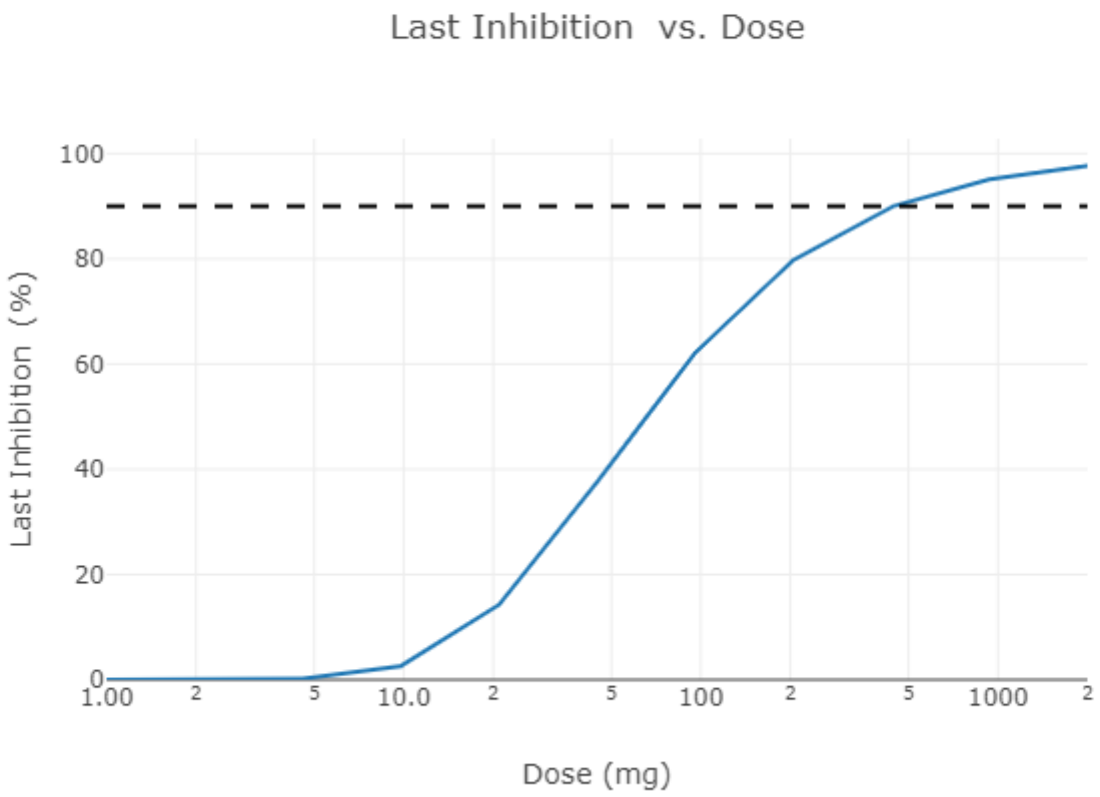

 © Applied BioMath LLC 2017-2021

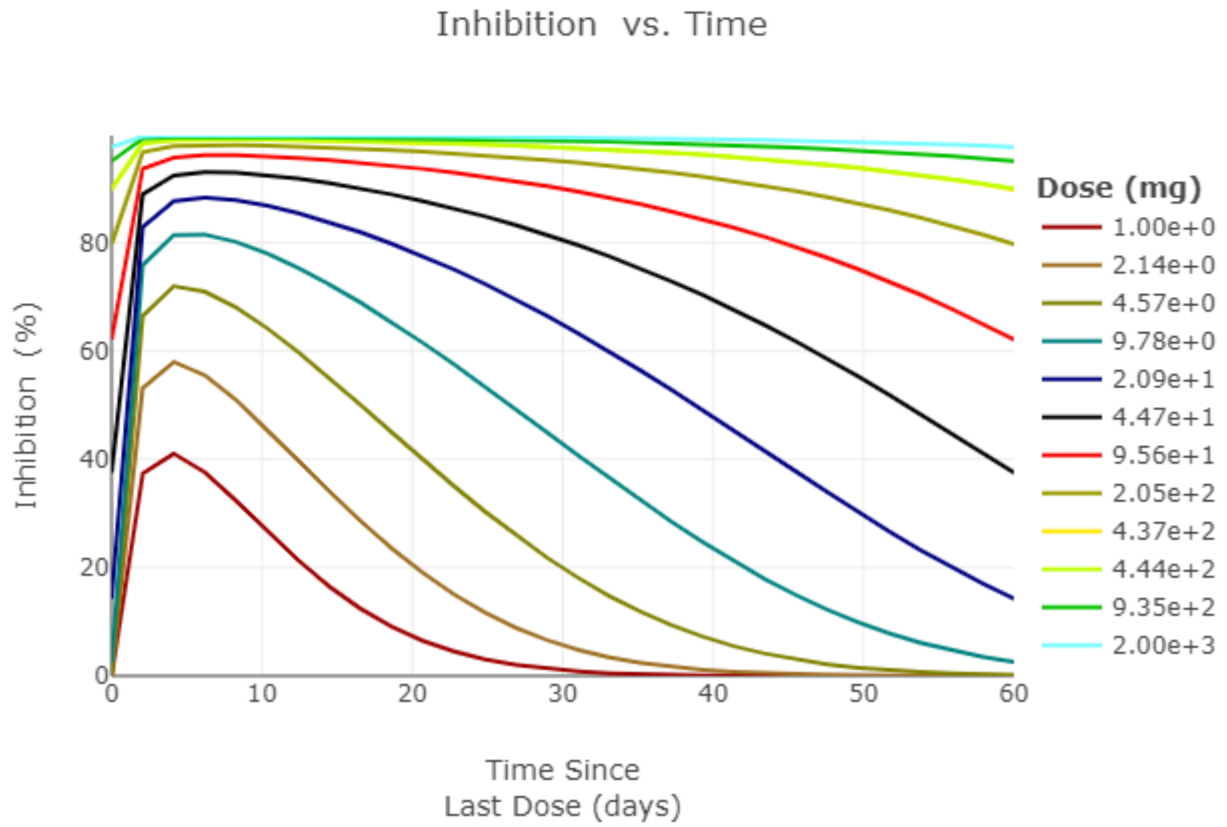

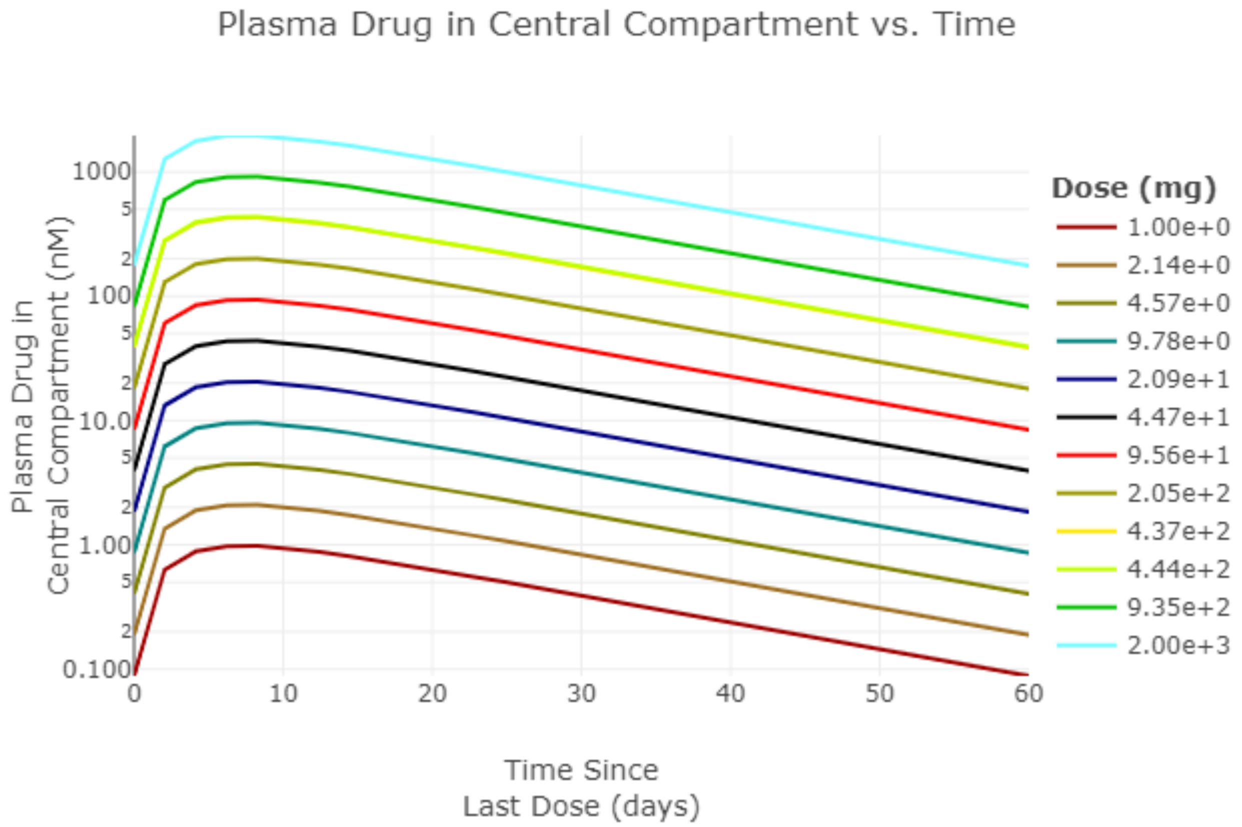

 © Applied BioMath LLC 2017-2021

2.2) Scenario: Ustekinumab

One dimensional scan over parameter, Dose, between 1 and 2000 using log spacing.

2.2.1) Model Parameters

Table 2.2.1

Symbol	Parameter ID	Value	Unit
τ	interval	90	-
D	dose	100	mg
$K_{D,\mathrm{L}}$	mab_kd_1	0.001	nM
N_{doses}	dose_count	7	-
MW	mw_1	148600	Da
$t_{1/2}$	el_half_1	21.6	days
$t_{1/2,\mathrm{a}}$	abs_half	2.5	days
BW	BW	70	kg
V	volume_central	5	L
$\mathrm{Valency}$	drug_valency_1	2	-
$t_{1/2,\mathrm{L}}$	lig_half_1	90	min
$t_{1/2,\mathrm{R}}$	rec_half_1	120	min
$t_{1/2,\mathrm{sR}}$	shed_half_1	0.5	hr
$K_{D,\mathrm{L:R}}$	lig_rec_kd_1	0.01	nM
$C_{SS,\mathrm{L}}$	lig_css_1_central	0.00038	nM
$C_{SS,\mathrm{R}}$	rec_css_1_central	0.0122	nM
$C_{SS,\mathrm{sR}}$	shed_css_1_central	0	nM
$\mathrm{Density}_{\mathrm{cells}}$	cell_density_mL_central	1000000	#/mL

Parameter ID	Value
Scan Parameter 1	Dose
Lower Limit 1	1
Upper Limit 1	2000
N1	11
Scale 1	log

 © Applied BioMath LLC 2017-2021

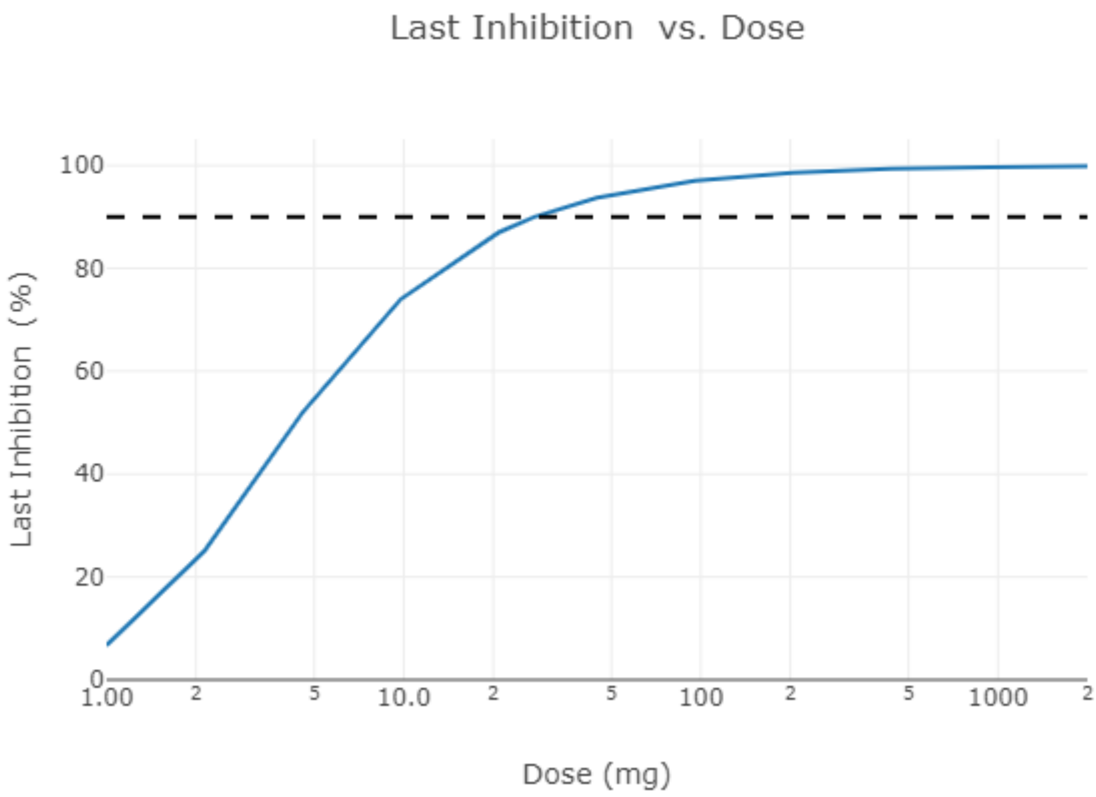

Last Inhibition vs. Dose
Last Inhibition (%)
100
80
60
40
20
0
1.00
2
5
10.0
2
5
100
2
5
1000
2
Dose (mg)

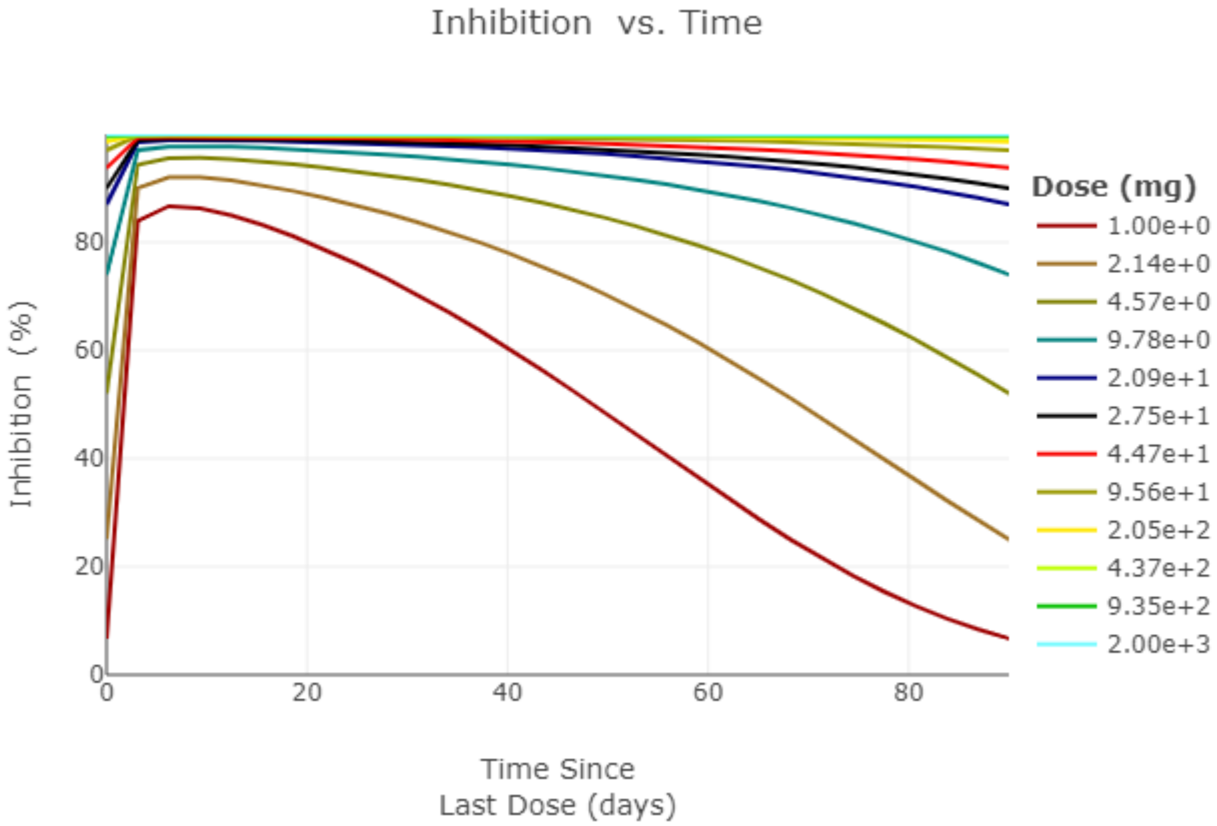

Inhibition vs. Time
Inhibition (%)
80
60
40
20
0
0
20
40
60
80
Time Since
Last Dose (days)
Dose (mg)
1.00e+0
2.14e+0
4.57e+0
9.78e+0
2.09e+1
2.75e+1
4.47e+1
9.56e+1
2.05e+2
4.37e+2
9.35e+2
2.00e+3

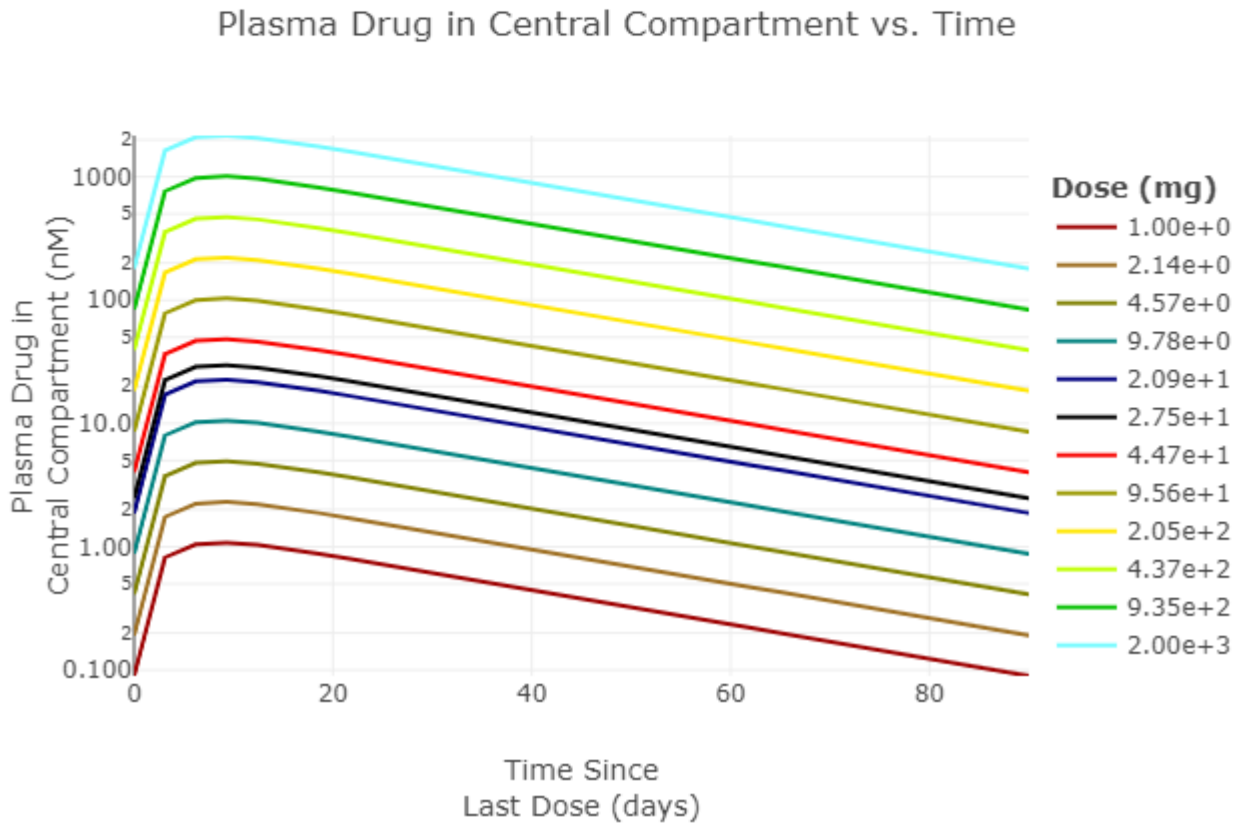

2.3) Scenario: Risankizumab, Q12W

One dimensional scan over parameter, Dose, between 1 and 2000 using log spacing.

2.3.1) Model Parameters

Table 2.3.1

Symbol	Parameter ID	Value	Unit
τ	interval	90	-
D	dose	100	mg
$K_{D,\mathrm{L}}$	mab_kd_1	0.001	nM
N_{doses}	dose_count	7	-
MW	mw_1	145610	Da
$t_{1/2}$	el_half_1	27	days
$t_{1/2,\mathrm{a}}$	abs_half	2.5	days
BW	BW	70	kg
V	volume_central	5	L
$\mathrm{Valency}$	drug_valency_1	2	-
$t_{1/2,\mathrm{L}}$	lig_half_1	90	min
$t_{1/2,\mathrm{R}}$	rec_half_1	120	min

 © Applied BioMath LLC 2017-2021

$t_{1/2,\mathrm{sR}}$	shed_half_1	0.5	hr
$K_{D,\mathrm{L:R}}$	lig_rec_kd_1	1.6	nM
$C_{SS,\mathrm{L}}$	lig_css_1_central	0.174	nM
$C_{SS,\mathrm{R}}$	rec_css_1_central	0.88	nM
$C_{SS,\mathrm{sR}}$	shed_css_1_central	0	nM
$\mathrm{Density}_{\mathrm{cells}}$	cell_density_mL_central	1000000	#/mL

Parameter ID	Value
Scan Parameter 1	Dose
Lower Limit 1	1
Upper Limit 1	2000
N1	11
Scale 1	log

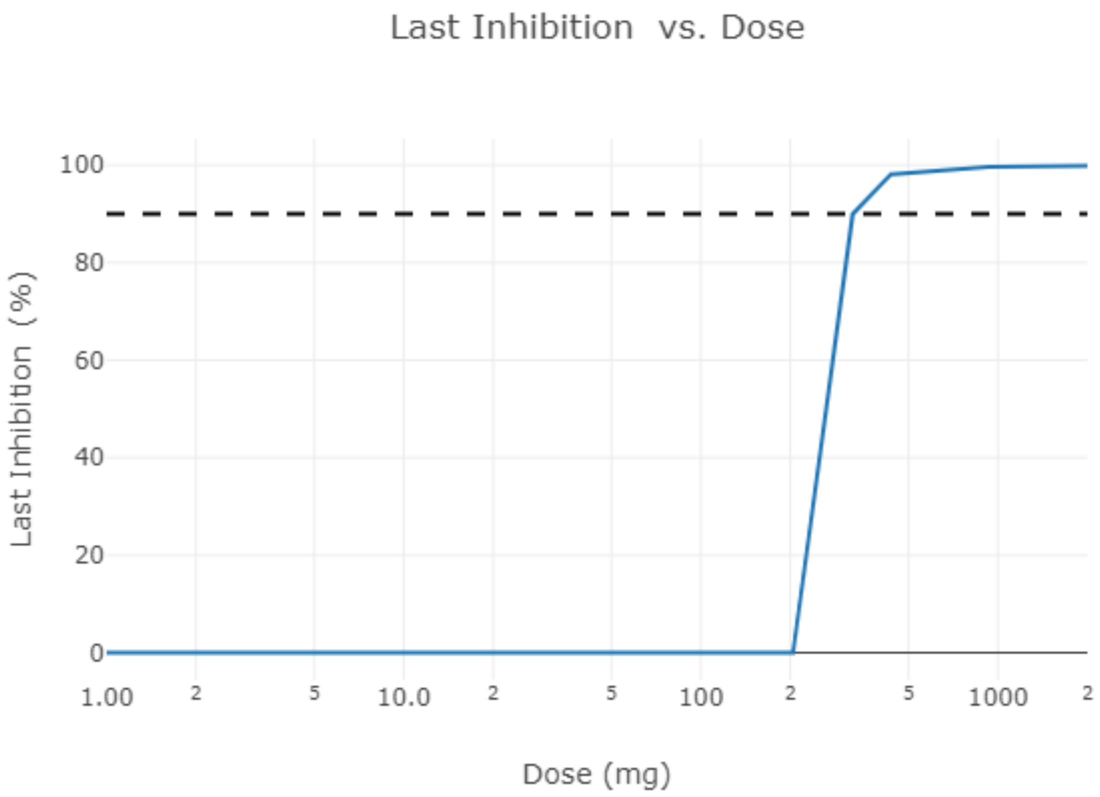

 © Applied BioMath LLC 2017-2021

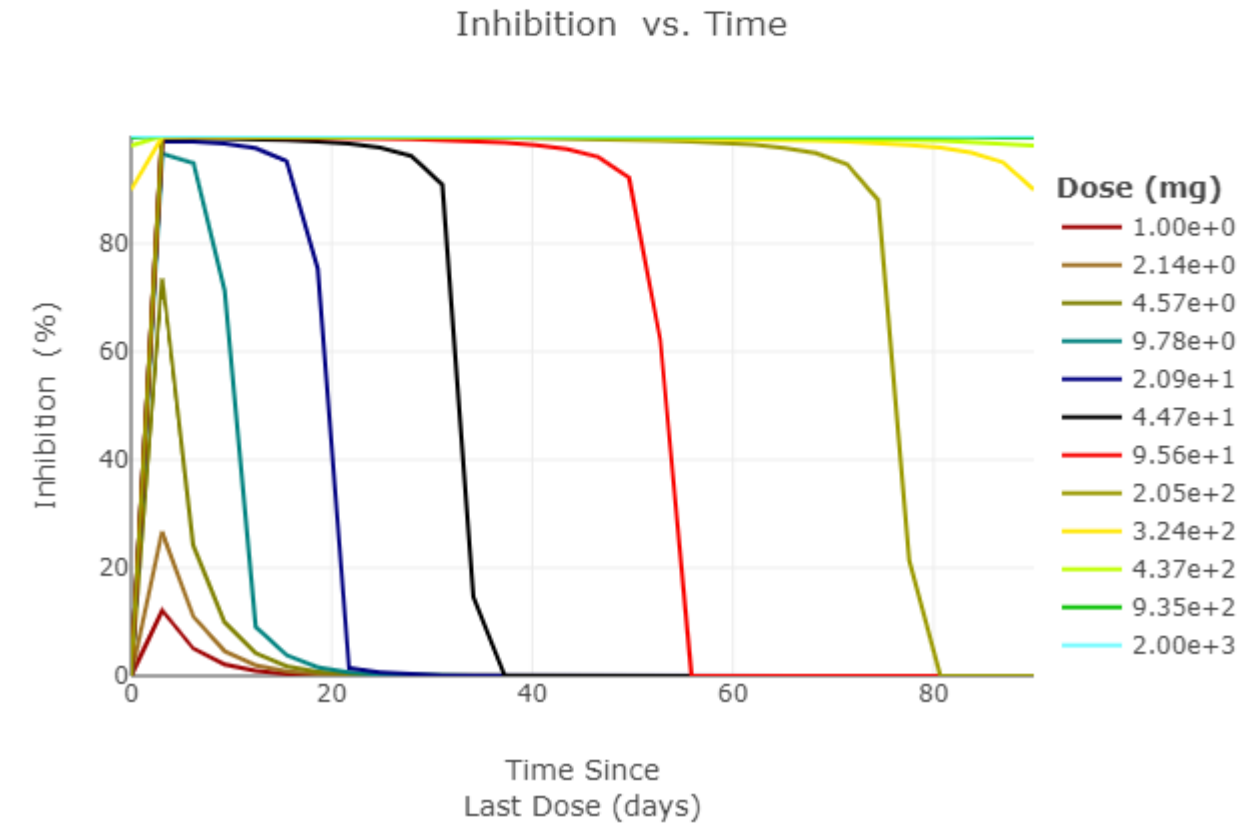
Inhibition vs. Time
Inhibition (%)
0
20
40
60
80
0
20
40
60
80
Time Since
Last Dose (days)
Dose (mg)
1.00e+0
2.14e+0
4.57e+0
9.78e+0
2.09e+1
4.47e+1
9.56e+1
2.05e+2
3.24e+2
4.37e+2
9.35e+2
2.00e+3

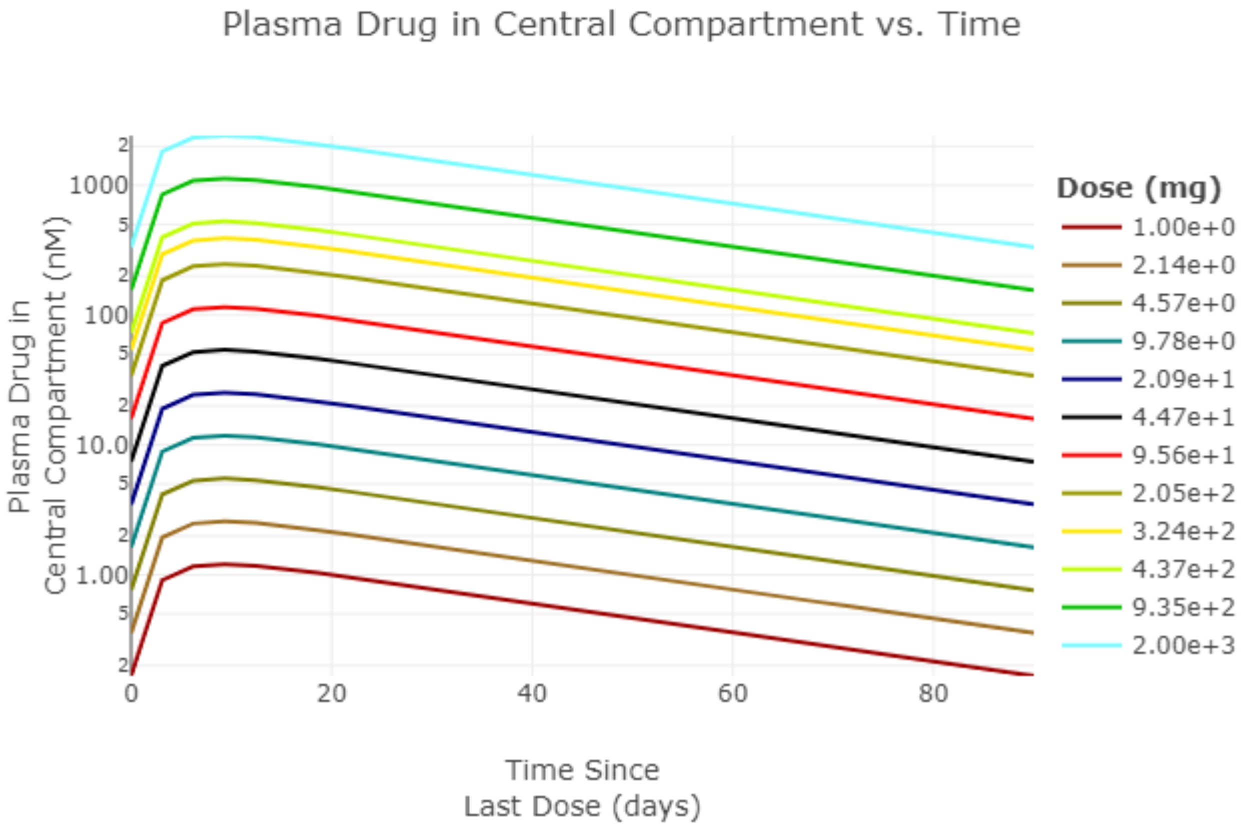
Plasma Drug in Central Compartment vs. Time
Plasma Drug in
Central Compartment (nM)
1000
100
10.0
1.00
0
20
40
60
80
Time Since
Last Dose (days)
Dose (mg)
1.00e+0
2.14e+0
4.57e+0
9.78e+0
2.09e+1
4.47e+1
9.56e+1
2.05e+2
3.24e+2
4.37e+2
9.35e+2
2.00e+3

2.4) Scenario: Risankizumab, Q4W

One dimensional scan over parameter, Dose, between 1 and 2000 using log spacing.

2.4.1) Model Parameters

Table 2.4.1

Symbol	Parameter ID	Value	Unit
τ	interval	30	-
D	dose	100	mg
$K_{D,\mathrm{L}}$	mab_kd_1	0.001	nM
N_{doses}	dose_count	7	-
MW	mw_1	145610	Da
$t_{1/2}$	el_half_1	27	days
$t_{1/2,\mathrm{a}}$	abs_half	2.5	days
BW	BW	70	kg
V	volume_central	5	L
$\mathrm{Valency}$	drug_valency_1	2	-
$t_{1/2,\mathrm{L}}$	lig_half_1	90	min
$t_{1/2,\mathrm{R}}$	rec_half_1	120	min
$t_{1/2,\mathrm{sR}}$	shed_half_1	0.5	hr
$K_{D,\mathrm{L:R}}$	lig_rec_kd_1	1.6	nM
$C_{ss,\mathrm{L}}$	lig_css_1_central	0.174	nM
$C_{ss,\mathrm{R}}$	rec_css_1_central	0.88	nM
$C_{ss,\mathrm{sR}}$	shed_css_1_central	0	nM
$\mathrm{Density}_{\mathrm{cells}}$	cell_density_mL_central	1000000	#/mL

Parameter ID	Value
Scan Parameter 1	Dose
Lower Limit 1	1
Upper Limit 1	2000
N1	11
Scale 1	log

 © Applied BioMath LLC 2017-2021

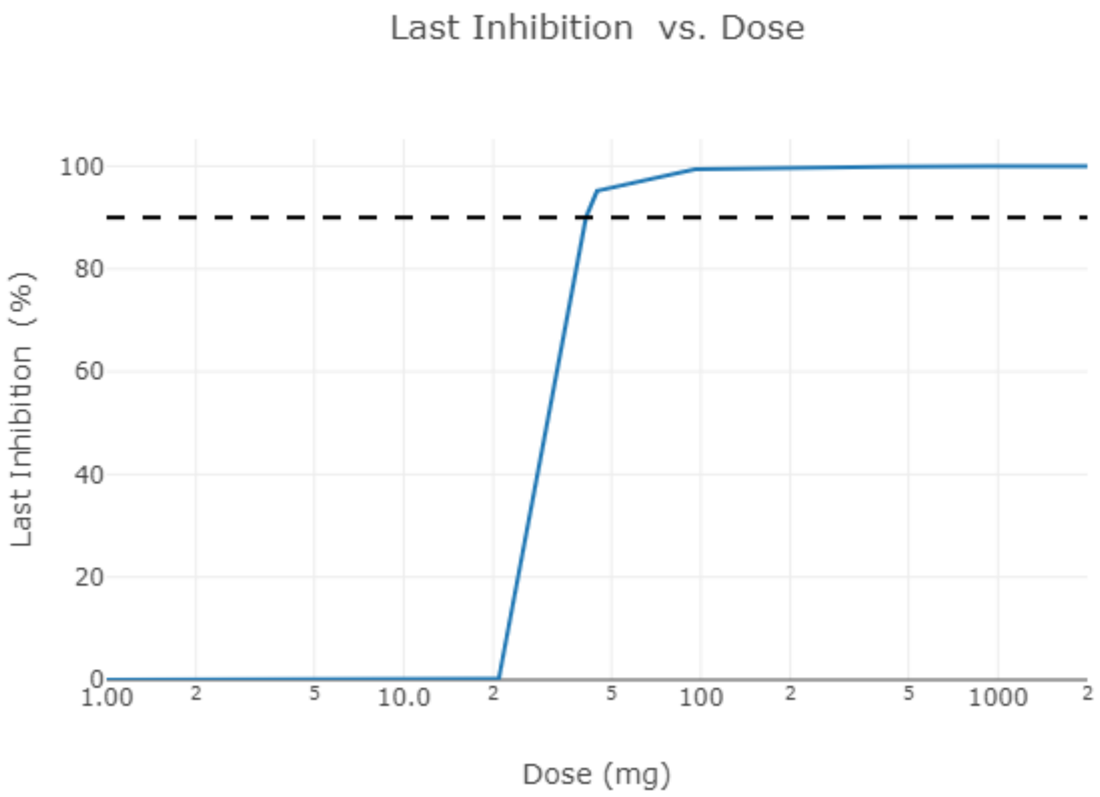
Last Inhibition vs. Dose
Last Inhibition (%)
100
80
60
40
20
0
1.00
2
5
10.0
2
5
100
2
5
1000
2
Dose (mg)

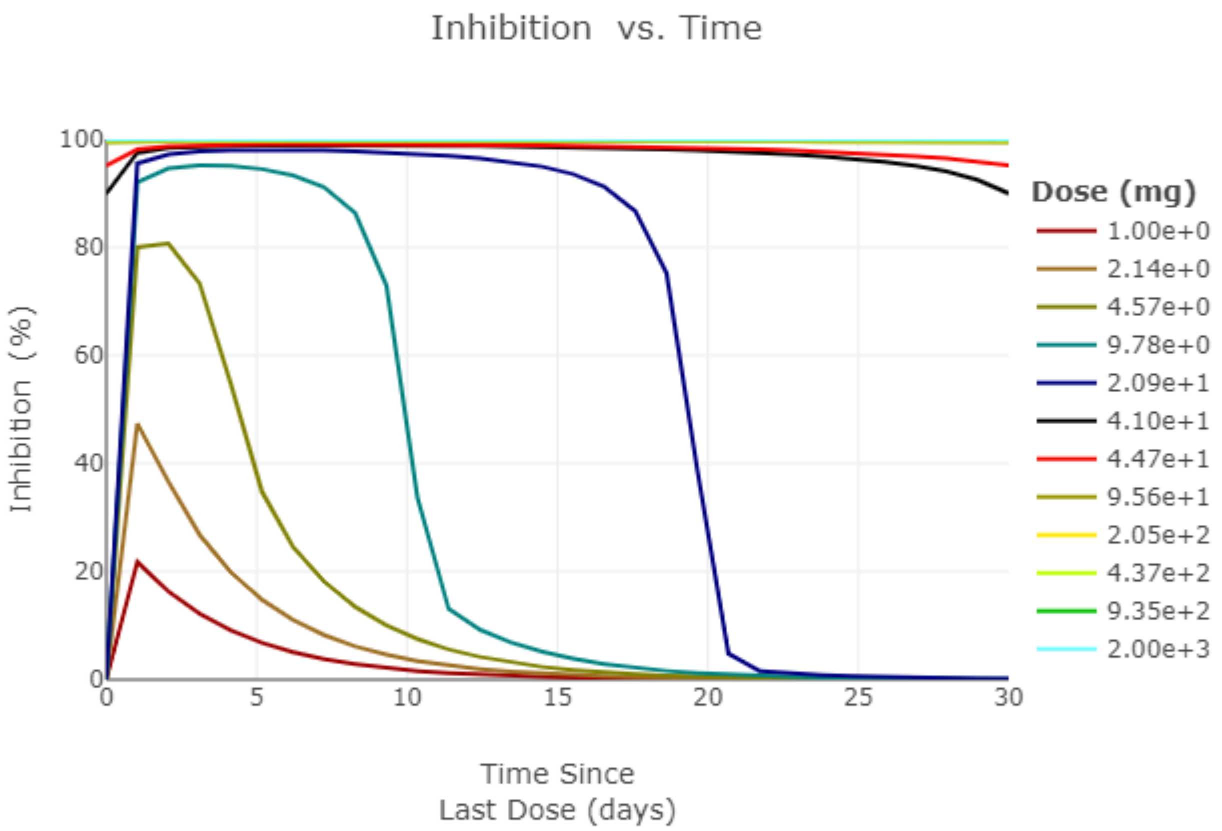
Inhibition vs. Time
Inhibition (%)
100
80
60
40
20
0
0
5
10
15
20
25
30
Time Since
Last Dose (days)
Dose (mg)
1.00e+0
2.14e+0
4.57e+0
9.78e+0
2.09e+1
4.10e+1
4.47e+1
9.56e+1
2.05e+2
4.37e+2
9.35e+2
2.00e+3

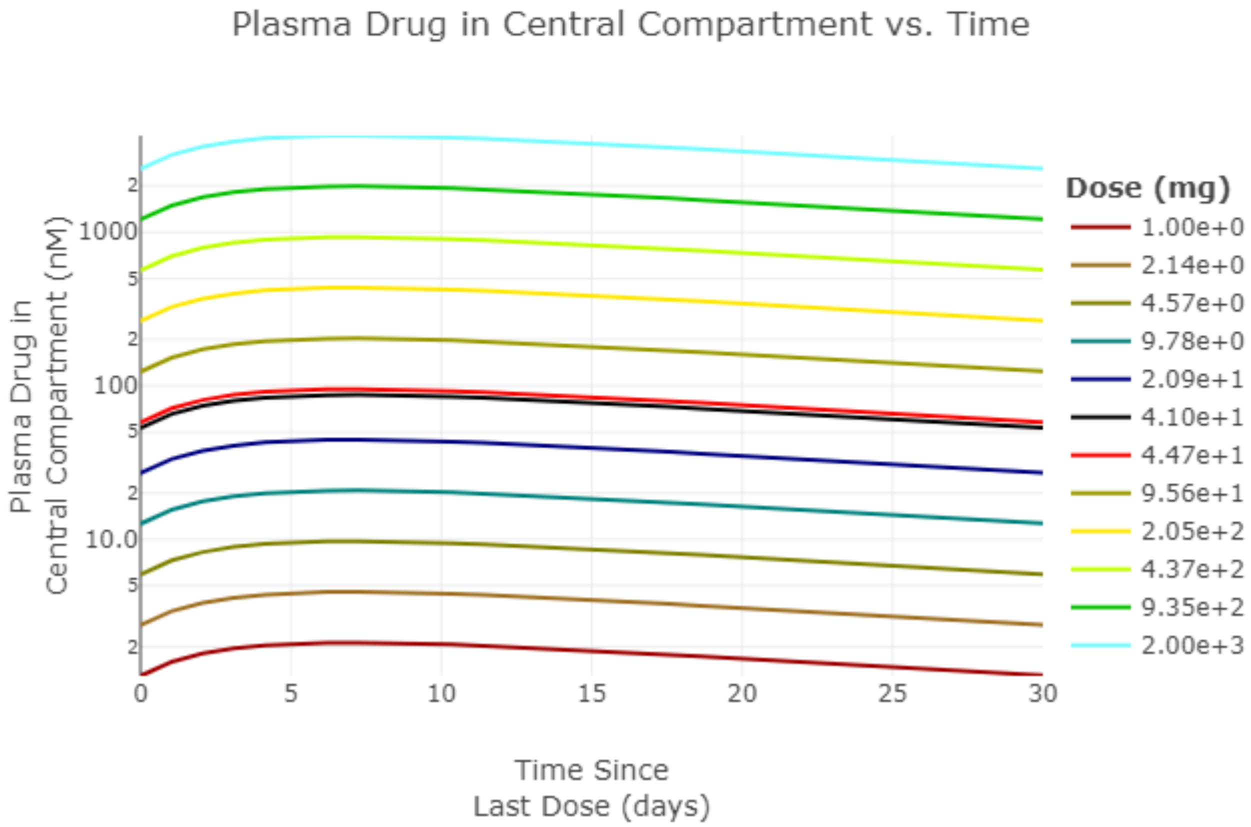

2.5) Scenario: Belimumab, Q1W SC

One dimensional scan over parameter, Dose, between 1 and 2000 using log spacing.

2.5.1) Model Parameters

Table 2.5.1

Symbol	Parameter ID	Value	Unit
τ	interval	7	-
D	dose	100	mg
$K_{D,\mathrm{L}}$	mab_kd_1	0.274	nM
N_{doses}	dose_count	7	-
MW	mw_1	147000	Da
$t_{1/2}$	el_half_1	19.4	days
$t_{1/2,\mathrm{a}}$	abs_half	2.5	days
BW	BW	70	kg
V	volume_central	5	L
Valency	drug_valency_1	1	-
$t_{1/2,\mathrm{L}}$	lig_half_1	60	min
$t_{1/2,\mathrm{R}}$	rec_half_1	120	min

 © Applied BioMath LLC 2017-2021

$t_{1/2,\mathrm{sR}}$	shed_half_1	0.5	hr
$K_{D,\mathrm{L:R}}$	lig_rec_kd_1	15	nM
$C_{SS,\mathrm{L}}$	lig_css_1_central	0.294	nM
$C_{SS,\mathrm{R}}$	rec_css_1_central	0.058	nM
$C_{SS,\mathrm{sR}}$	shed_css_1_central	0	nM
$\mathrm{Density}_{\mathrm{cells}}$	cell_density_mL_central	1000000	#/mL

Parameter ID	Value
Scan Parameter 1	Dose
Lower Limit 1	1
Upper Limit 1	2000
N1	11
Scale 1	log

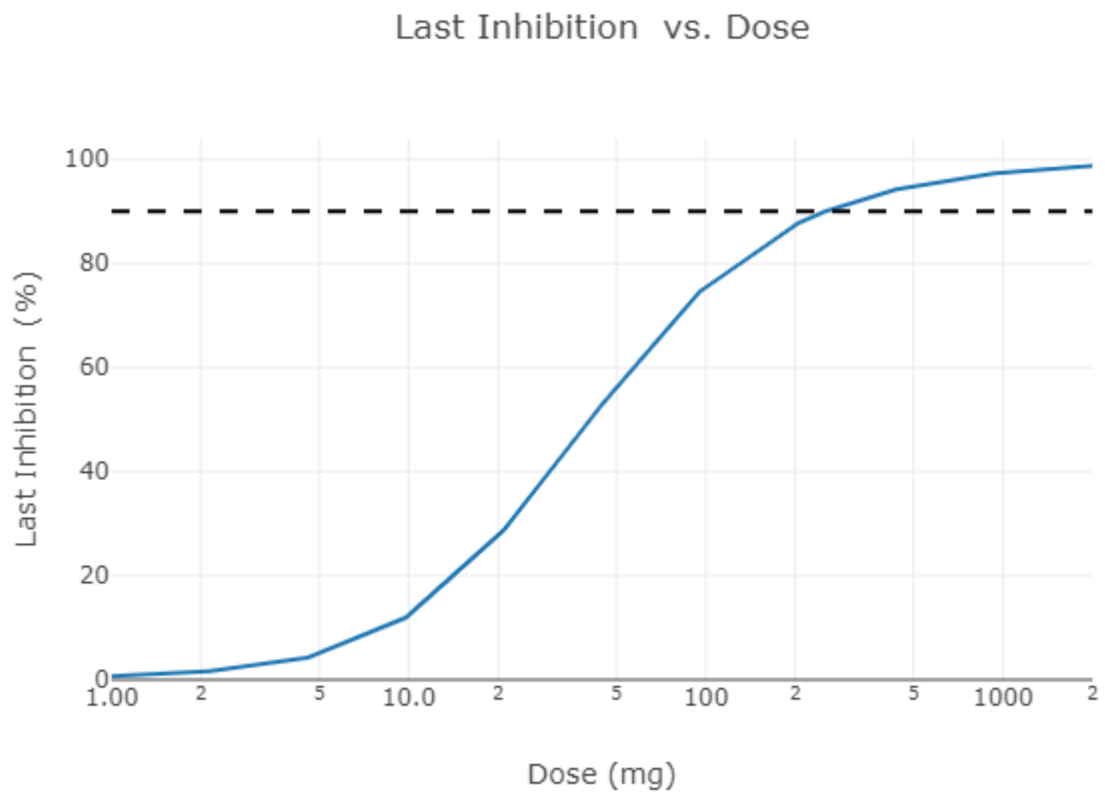

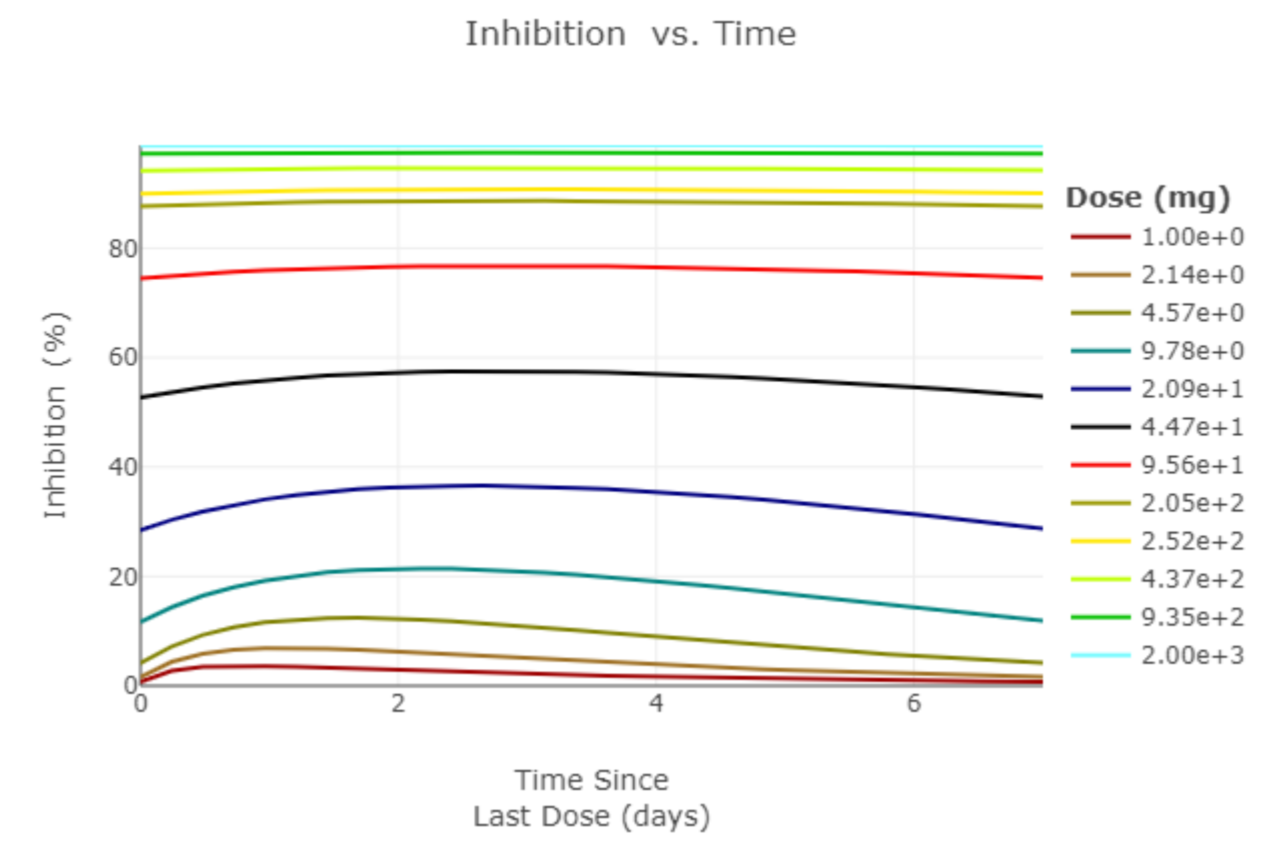

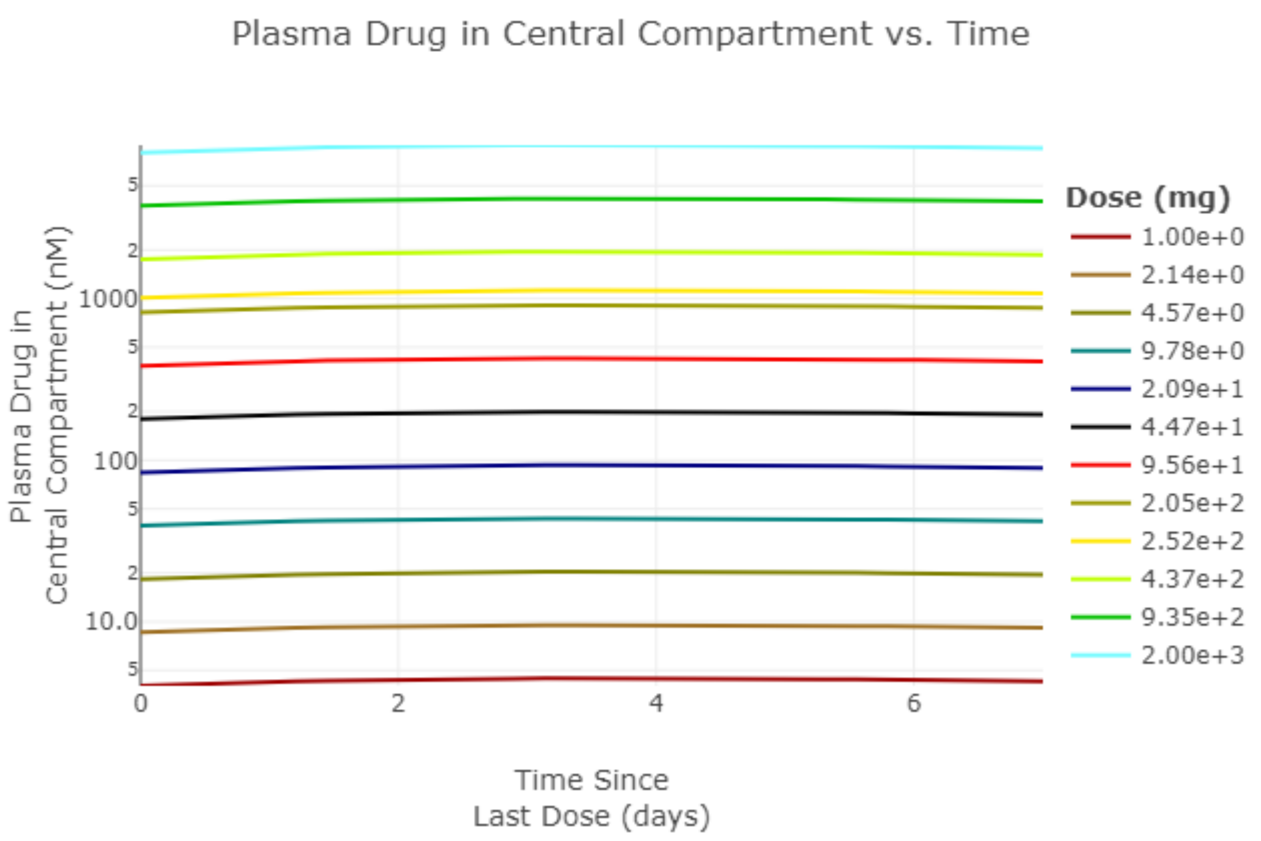

 © Applied BioMath LLC 2017-2021

2.6) Scenario: Belimumab, Q1W SC

One dimensional scan over parameter, Dose, between 1 and 2000 using log spacing.

2.6.1) Model Parameters

Table 2.6.1

Symbol	Parameter ID	Value	Unit
τ	interval	30	-
D	dose	100	mg
$K_{D,\mathrm{L}}$	mab_kd_1	0.274	nM
N_{doses}	dose_count	7	-
MW	mw_1	147000	Da
$t_{1/2}$	el_half_1	19.4	days
$t_{1/2,\mathrm{a}}$	abs_half	2.5	days
BW	BW	70	kg
V	volume_central	5	L
$\mathrm{Valency}$	drug_valency_1	1	-
$t_{1/2,\mathrm{L}}$	lig_half_1	60	min
$t_{1/2,\mathrm{R}}$	rec_half_1	120	min
$t_{1/2,\mathrm{sR}}$	shed_half_1	0.5	hr
$K_{D,\mathrm{L:R}}$	lig_rec_kd_1	15	nM
$C_{SS,\mathrm{L}}$	lig_css_1_central	0.294	nM
$C_{SS,\mathrm{R}}$	rec_css_1_central	0.058	nM
$C_{SS,\mathrm{sR}}$	shed_css_1_central	0	nM
$\mathrm{Density}_{\mathrm{cells}}$	cell_density_mL_central	1000000	#/mL

Parameter ID	Value
Scan Parameter 1	Dose
Lower Limit 1	1
Upper Limit 1	2000
N1	11
Scale 1	log

 © Applied BioMath LLC 2017-2021

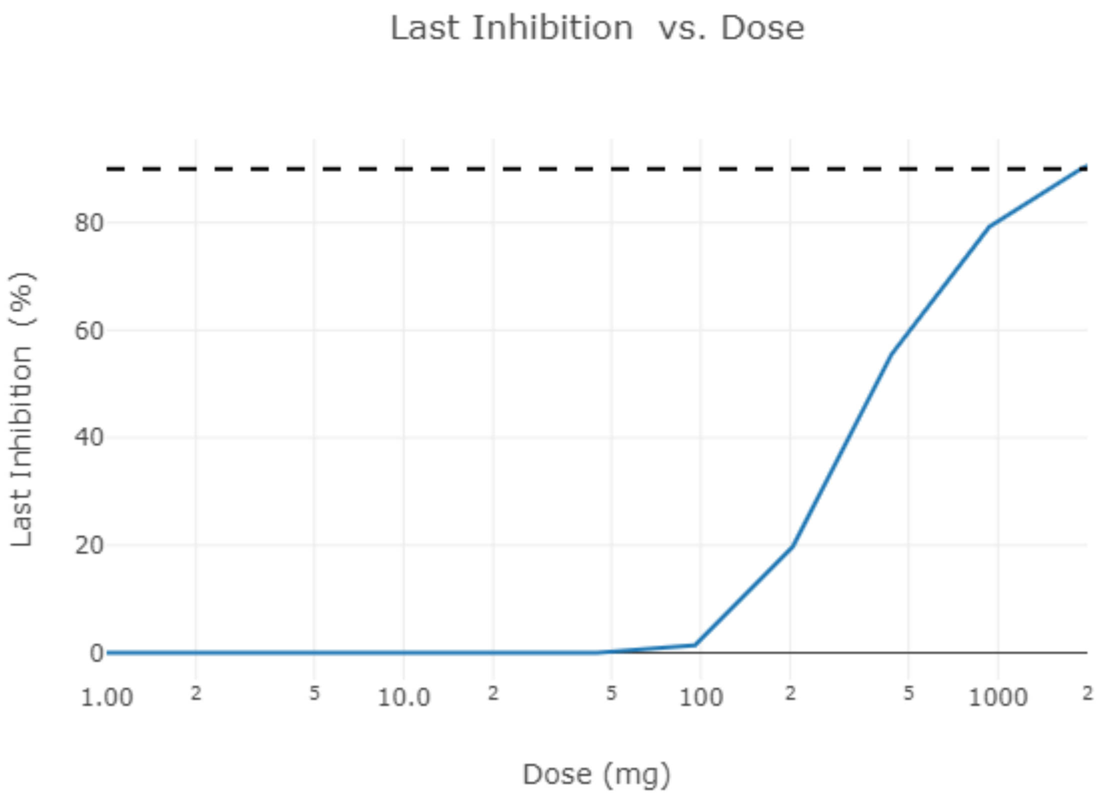

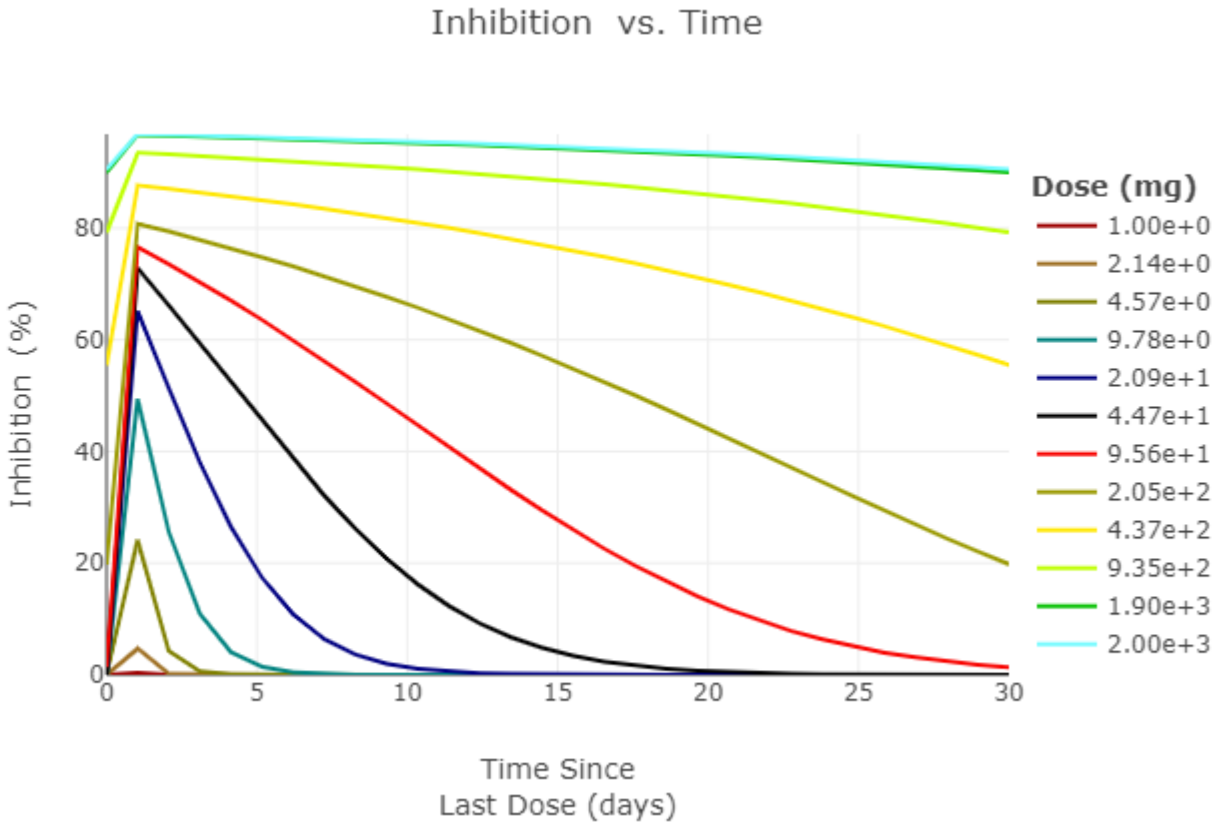

 © Applied BioMath LLC 2017-2021

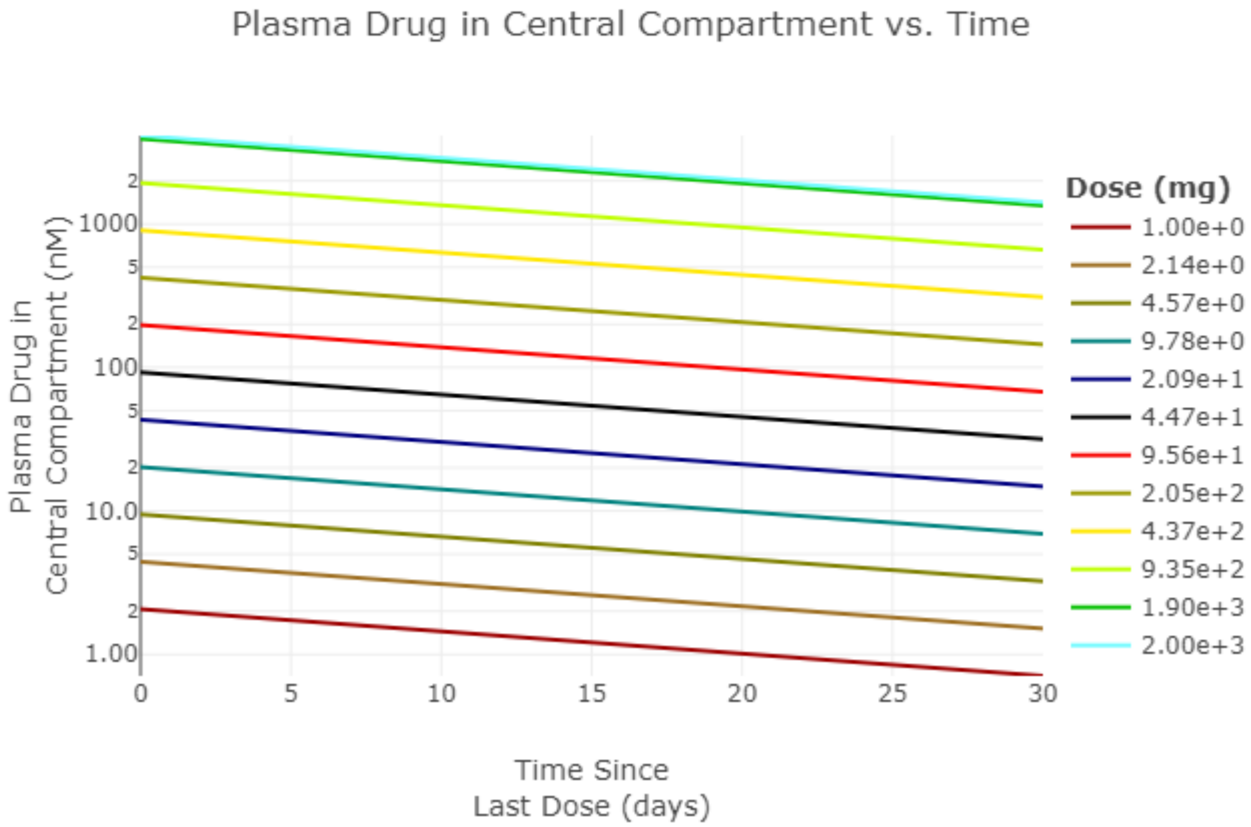

2.7) Scenario: Omalizumab

One dimensional scan over parameter, Dose, between 1 and 2000 using log spacing.

2.7.1) Model Parameters

Table 2.7.1

Symbol	Parameter ID	Value	Unit
τ	interval	14	-
D	dose	100	mg
$K_{D,\mathrm{L}}$	mab_kd_1	0.02	nM
N_{doses}	dose_count	7	-
MW	mw_1	149000	Da
$t_{1/2}$	el_half_1	26	days
$t_{1/2,\mathrm{a}}$	abs_half	2.5	days
BW	BW	70	kg
V	volume_central	5	L
Valency	drug_valency_1	2	-
$t_{1/2,\mathrm{L}}$	lig_half_1	2880	min
$t_{1/2,\mathrm{R}}$	rec_half_1	15	min

$t_{1/2,\mathrm{sR}}$	shed_half_1	0.5	hr
$K_{D,\mathrm{L:R}}$	lig_rec_kd_1	0.12	nM
$C_{SS,\mathrm{L}}$	lig_css_1_central	8	nM
$C_{SS,\mathrm{R}}$	rec_css_1_central	0.78	nM
$C_{SS,\mathrm{sR}}$	shed_css_1_central	0	nM
$\mathrm{Density}_{\mathrm{cells}}$	cell_density_mL_central	1000000	#/mL

Parameter ID	Value
Scan Parameter 1	Dose
Lower Limit 1	1
Upper Limit 1	2000
N1	11
Scale 1	log

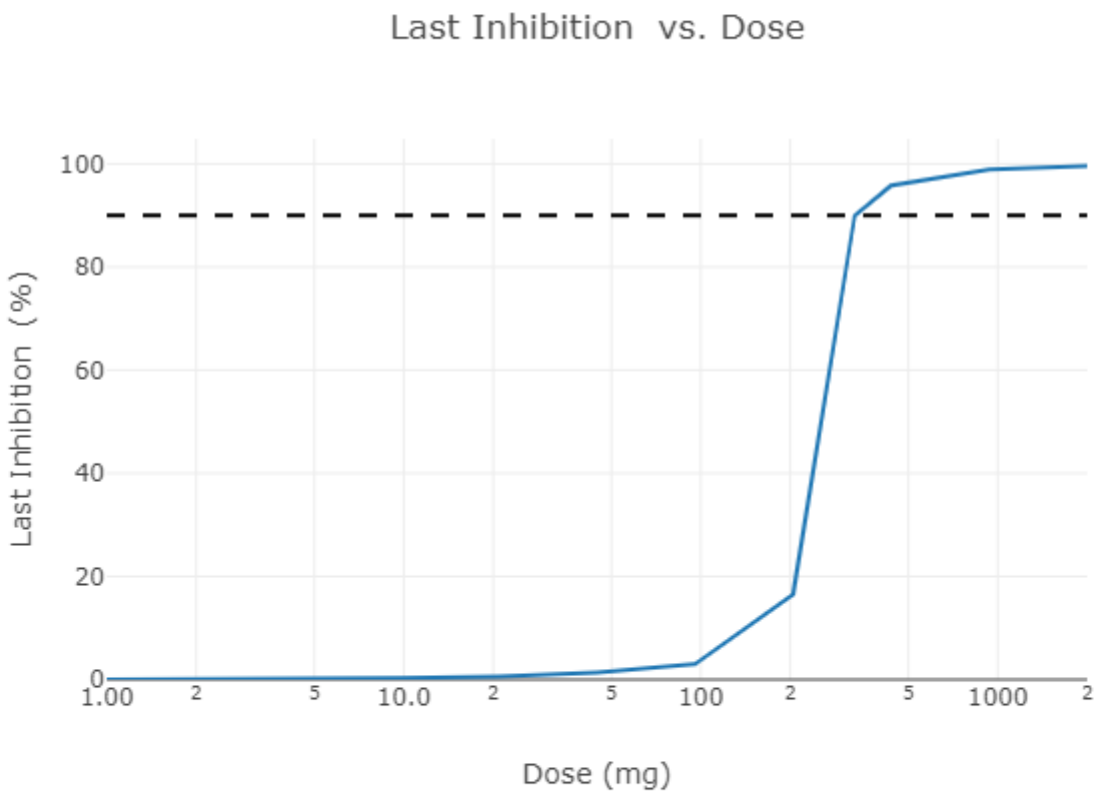

 © Applied BioMath LLC 2017-2021

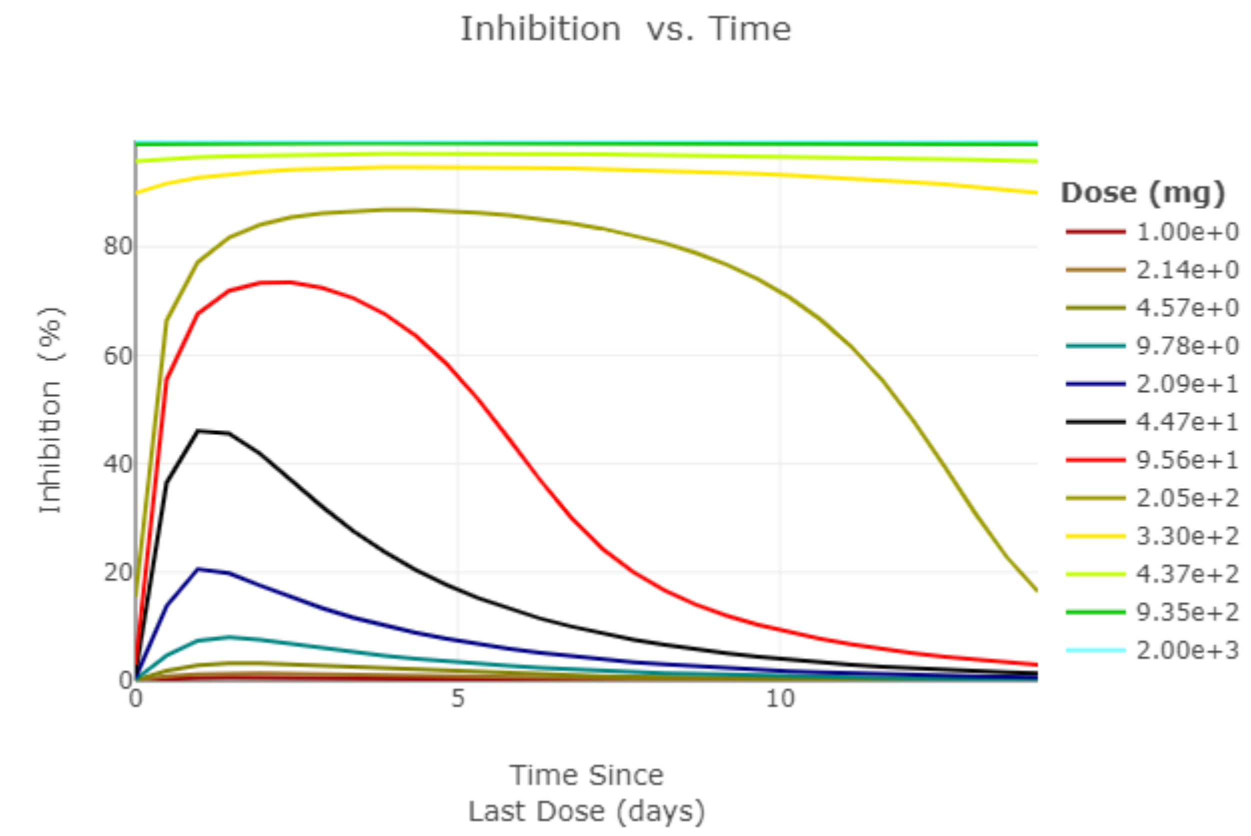

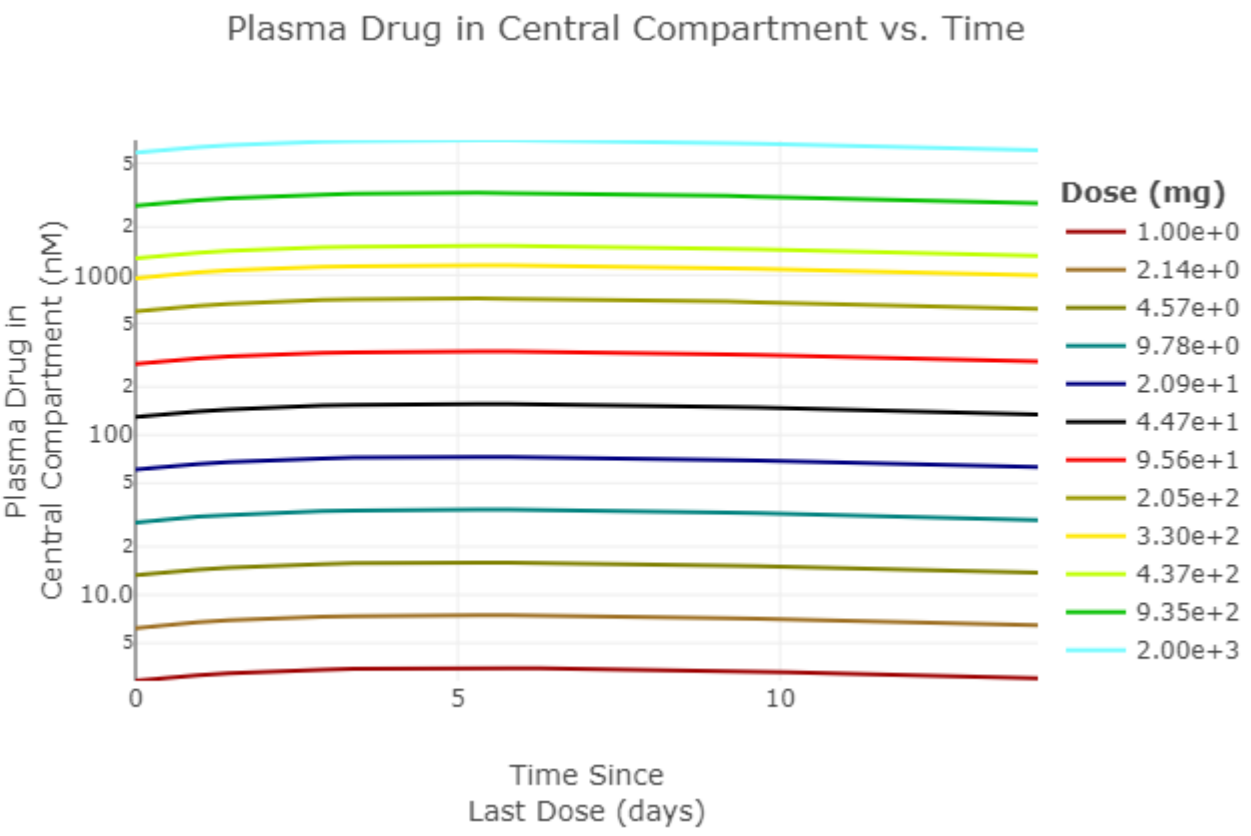

 © Applied BioMath LLC 2017-2021
