# Supplementary material for: Early Feasibility Assessment: A Method for Accurately Predicting Biotherapeutic Dosing to Inform Early Drug Discovery Decisions
Source: Front Pharmacol. 2022 Jun 8;13:864768. doi: 10.3389/fphar.2022.864768 (PMC9214263; doi:10.3389/fphar.2022.864768)
Supplement: Supplementary file 3 [file DataSheet2.ZIP › Model run files_json and reports/Panitumumab_CaseStudy.pdf]

revolutionizing drug invention

Panitumumab Case Study
generated with
Applied BioMath Assess™

Model: Monospecific Anti-Receptor (4-Compartment)

Date: 2022-03-29T18:54:03.708Z

Software Version: 2022.3.2

This report does not fall within the scope of the United States Food and Drug Administration Good Laboratory Practice or Good Clinical Practice Regulations.

© Applied BioMath LLC 2017-2021

1) Summary

The goal of this study is to determine the feasibility of a drug concept for a target by varying parameters of a Quantitative Systems Pharmacology (QSP) model. By performing simulations over a range of drug and target parameters, this study supports comparison of the effects of dose amount, dose frequency, route of administration, drug design criteria, and target characteristics. This assessment can aid in the prediction of difficulty of discovering and formulation a New Biological Entity that satisfies a Target Product Profile.

The analyses explore the model conditions under which one or more criteria are satisfied. Depending on the kind of analysis, the feasibility of a scenario is defined to be either:

- the dose that exactly satisfies the criteria targets,
- a range of a parameter that satisfies the criteria targets,
- or a region of a parameter grid that satisfies the criteria targets.

The model used in these analyses is: Monospecific Anti-Receptor (4-Compartment) - A biologic that binds to a cell surface receptor target and either (1) acts as a competitive inhibitor by blocking the cognate-ligand from binding to its receptor or (2) acts as a receptor agonist. The molecule can be mono- or bivalent. This is a four-compartment model with a central, peripheral, disease and tox compartments. There is an option for +/- soluble receptor.

 © Applied BioMath LLC 2017-2021

Scenario Table

Description	Route	Interval (null)	Dose (mg)	L:R Affinity (nM)	Ligand CSS disease (nM)	Ligand CSS tox (nM)	Receptor CSS disease (#/cell)	Receptor CSS tox (#/cell)	Scan Parameter	Criterion	Result
Panitumumab	IV	14	-	1	0.05	0.05	10000	10000	: Dose (mg) [100 - 440]	Last Target Engagement Peripheral ≥ 98 %	Dose ≥ 162 mg

© Applied BioMath LLC 2017-2021

2) Results

2.0) Scenario: Panitumumab

One dimensional scan over parameter, Dose, between 100 and 440 using linear spacing.

2.0.1) Model Parameters

Table 2.0.1

Symbol	Parameter ID	Value	Unit
τ	interval	14	-
D	dose	100	mg
$K_{D,\mathrm{R}}$	mab_kd_1	0.05	nM
N_{doses}	dose_count	7	-
MW	mw_1	150000	Da
$t_{1/2}$	el_half_1	16	days
$t_{1/2,\mathrm{a}}$	abs_half	2.5	days
BW	BW	70	kg
V	volume_central	3	L
$V_{\mathrm{peripheral}}$	volume_peripheral	13	L
V_{disease}	volume_disease	0.1	L
V_{tox}	volume_tox	0.1	L
$T_{\mathrm{dist,peripheral}}$	Tdist_Ab_hr_peripheral	35	hr
$T_{\mathrm{dist,disease}}$	Tdist_Ab_hr_disease	30	hr
$T_{\mathrm{dist,tox}}$	Tdist_Ab_hr_tox	30	hr
$P_{\mathrm{dist,peripheral}}$	Pdist_Ab_peripheral	0.190625	-
$P_{\mathrm{dist,disease}}$	Pdist_Ab_disease	0	-
$P_{\mathrm{dist,tox}}$	Pdist_Ab_tox	0	-
Valency	drug_valency_1	2	-
$t_{1/2,\mathrm{L}}$	lig_half_1	1000	hr
$t_{1/2,\mathrm{R}}$	rec_half_1	5	hr
$t_{1/2,\mathrm{sR}}$	shed_half_1	1000	hr
$K_{D,\mathrm{L:R}}$	lig_rec_kd_1	1	nM
$C_{SS,\mathrm{L,central}}$	lig_css_1_central	0.0001	nM
$C_{SS,\mathrm{L,peripheral}}$	lig_css_1_peripheral	0	nM

 © Applied BioMath LLC 2017-2021

$C_{SS,\mathrm{L,disease}}$	lig_css_1_disease	0.05	nM
$C_{SS,\mathrm{L,tox}}$	lig_css_1_tox	0.05	nM
$C_{SS,\mathrm{R,central}}$	rec_css_1_central	0.0152	nM
$C_{SS,\mathrm{R,peripheral}}$	rec_css_1_peripheral	1.13	nM
$C_{SS,\mathrm{R,disease}}$	rec_css_1_disease	10000	#/cell
$C_{SS,\mathrm{R,tox}}$	rec_css_1_tox	10000	#/cell
$C_{SS,\mathrm{sR,central}}$	shed_css_1_central	0	nM
$C_{SS,\mathrm{sR,peripheral}}$	shed_css_1_peripheral	0	nM
$C_{SS,\mathrm{sR,disease}}$	shed_css_1_disease	0	nM
$C_{SS,\mathrm{sR,tox}}$	shed_css_1_tox	0	nM
$T_{\mathrm{dist,L,peripheral}}$	Tdist_L1_hr_peripheral	30	hr
$T_{\mathrm{dist,L,disease}}$	Tdist_L1_hr_disease	30	hr
$T_{\mathrm{dist,L,tox}}$	Tdist_L1_hr_tox	30	hr
$T_{\mathrm{dist,sR,peripheral}}$	Tdist_S1_hr_peripheral	30	hr
$T_{\mathrm{dist,sR,disease}}$	Tdist_S1_hr_disease	30	hr
$T_{\mathrm{dist,sR,tox}}$	Tdist_S1_hr_tox	30	hr
$\mathrm{Density}_{\mathrm{cells,central}}$	cell_density_mL_central	1000000	#/mL
$\mathrm{Density}_{\mathrm{cells,peripheral}}$	cell_density_mL_peripheral	1000000	#/mL
$\mathrm{Density}_{\mathrm{cells,disease}}$	cell_density_mL_disease	1000000	#/mL
$\mathrm{Density}_{\mathrm{cells,tox}}$	cell_density_mL_tox	1000000	#/mL
$\mathrm{Scale}_{t_{1/2,\mathrm{R}},\mathrm{D:R,central}}$	scale_half_Ab_R1_central	1	-
$\mathrm{Scale}_{t_{1/2,\mathrm{R}},\mathrm{D:R,peripheral}}$	scale_half_Ab_R1_peripheral	1	-
$\mathrm{Scale}_{t_{1/2,\mathrm{R}},\mathrm{D:R,disease}}$	scale_half_Ab_R1_disease	1	-
$\mathrm{Scale}_{t_{1/2,\mathrm{R}},\mathrm{D:R,tox}}$	scale_half_Ab_R1_tox	1	-
$\mathrm{Scale}_{K_{D,\mathrm{R}},\mathrm{central}}$	scale_kd_Ab_T1_central	1	-
$\mathrm{Scale}_{K_{D,\mathrm{R}},\mathrm{peripheral}}$	scale_kd_Ab_T1_peripheral	1	-
$\mathrm{Scale}_{K_{D,\mathrm{R}},\mathrm{disease}}$	scale_kd_Ab_T1_disease	1	-
$\mathrm{Scale}_{K_{D,\mathrm{R}},\mathrm{tox}}$	scale_kd_Ab_T1_tox	1	-
$\mathrm{Scale}_{t_{1/2},\mathrm{central}}$	scale_half_Ab_central	1	-
$\mathrm{Scale}_{t_{1/2},\mathrm{peripheral}}$	scale_half_Ab_peripheral	1	-
$\mathrm{Scale}_{t_{1/2},\mathrm{disease}}$	scale_half_Ab_disease	1	-
$\mathrm{Scale}_{t_{1/2},\mathrm{tox}}$	scale_half_Ab_tox	1	-

Parameter ID	Value
Scan Parameter 1	Dose

 © Applied BioMath LLC 2017-2021

Lower Limit 1	100
Upper Limit 1	440
N1	3
Scale 1	linear

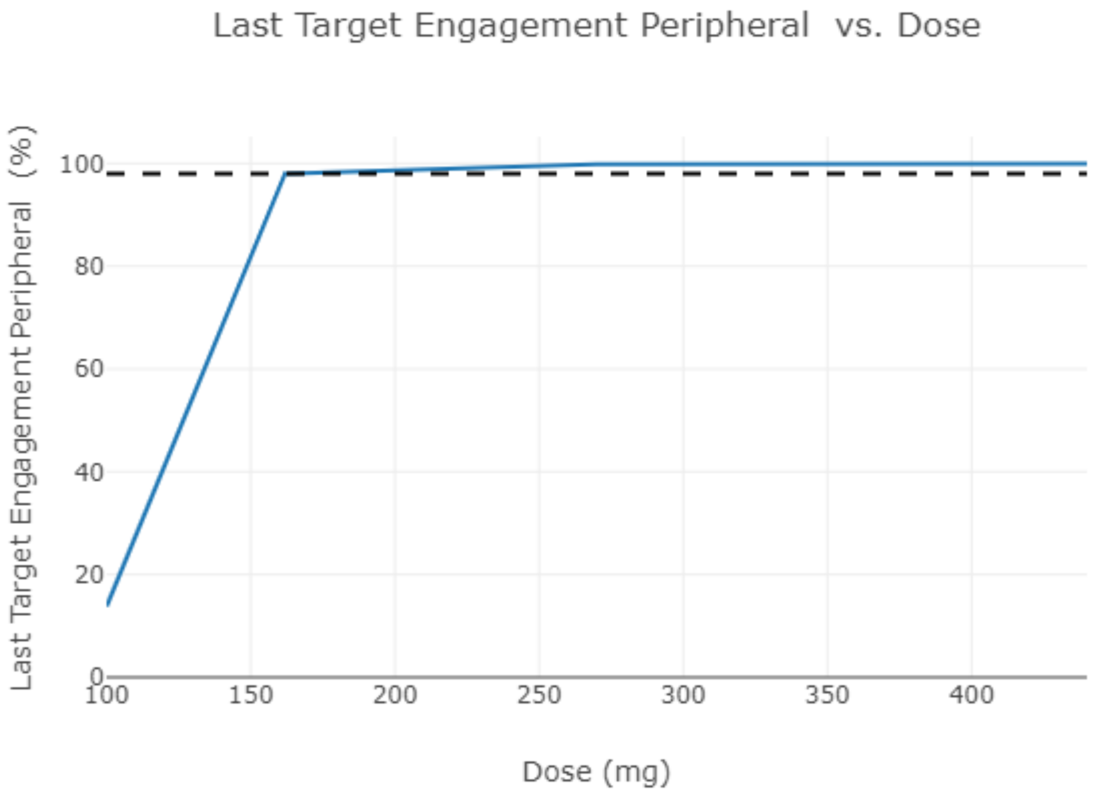

 © Applied BioMath LLC 2017-2021

Target Engagement Peripheral vs. Time

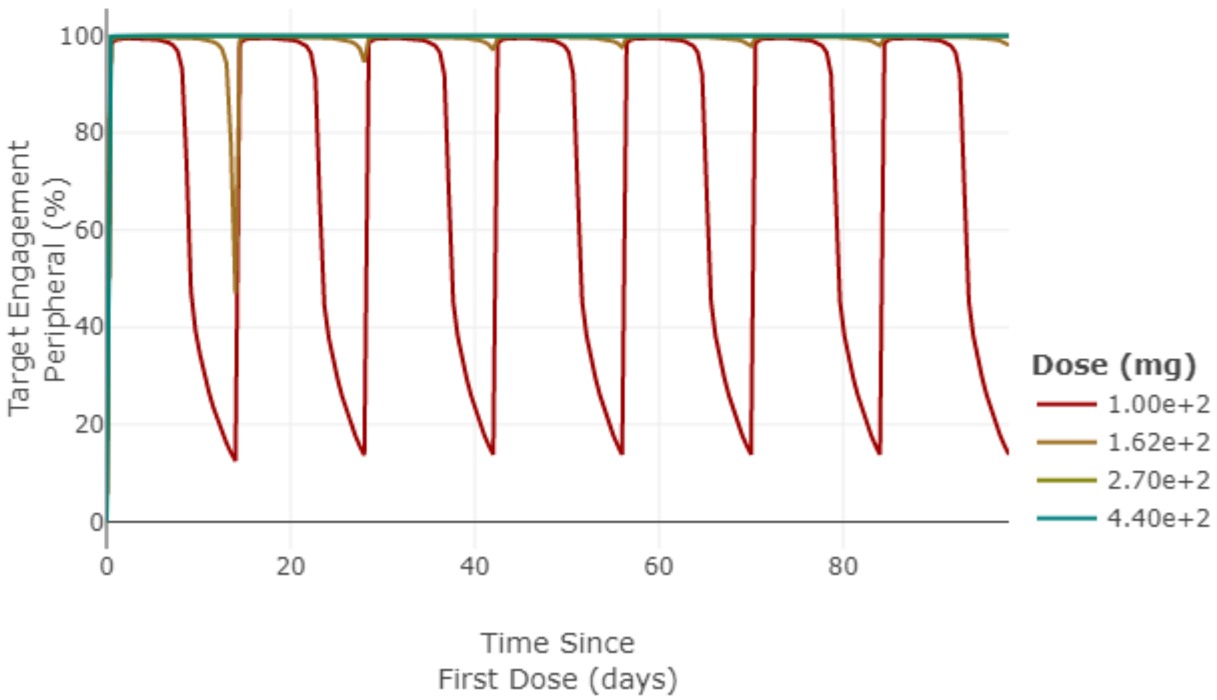

Plasma Drug in Central Compartment vs. Time

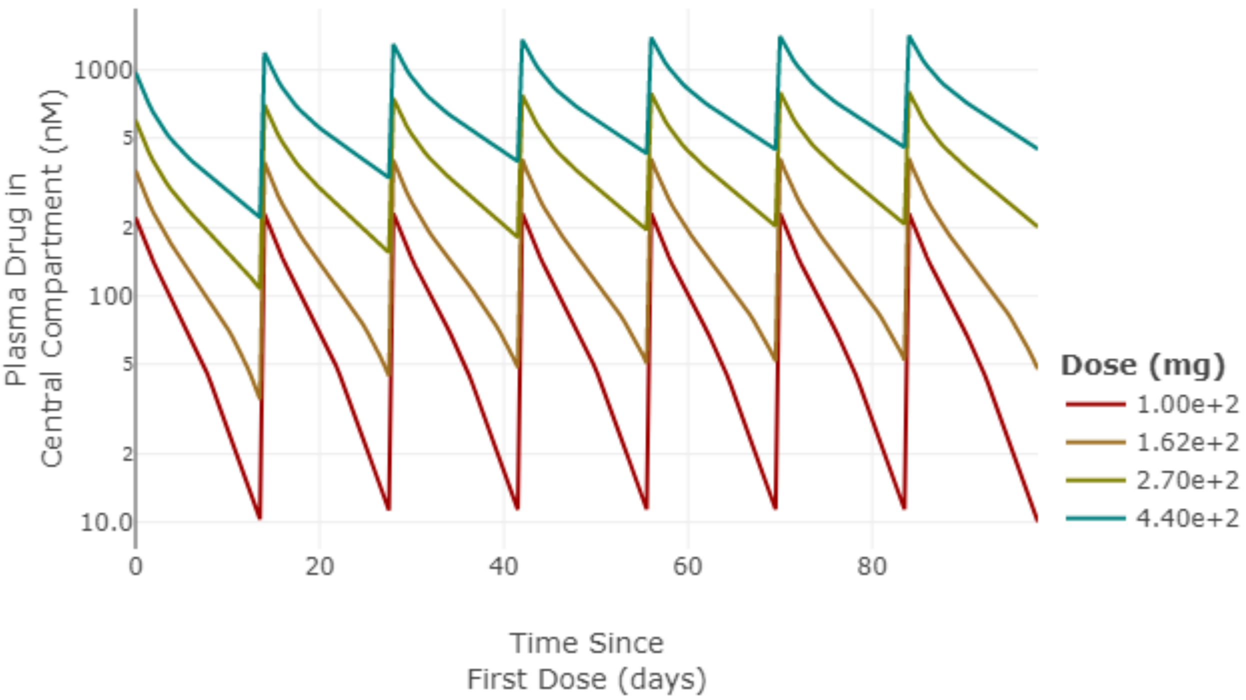

 © Applied BioMath LLC 2017-2021
