# Supplementary material for: Early Feasibility Assessment: A Method for Accurately Predicting Biotherapeutic Dosing to Inform Early Drug Discovery Decisions
Source: Front Pharmacol. 2022 Jun 8;13:864768. doi: 10.3389/fphar.2022.864768 (PMC9214263; doi:10.3389/fphar.2022.864768)
Supplement: Supplementary file 3 [file DataSheet2.ZIP › Model run files_json and reports/Infliximab_Sensitivity_Analysis.pdf]

Infliximab Parameter Scans
generated with
Applied BioMath Assess™

Model: Monospecific Anti-Ligand

Date: 2022-03-10T22:01:11.792Z

Software Version: 2022.2.2

This report does not fall within the scope of the United States Food and Drug Administration Good Laboratory Practice or Good Clinical Practice Regulations.

# 1) Summary

The goal of this study is to determine the feasibility of a drug concept for a target by varying parameters of a Quantitative Systems Pharmacology (QSP) model. By performing simulations over a range of drug and target parameters, this study supports comparison of the effects of dose amount, dose frequency, route of administration, drug design criteria, and target characteristics. This assessment can aid in the prediction of difficulty of discovering and formulation a New Biological Entity that satisfies a Target Product Profile.

The analyses explore the model conditions under which one or more criteria are satisfied. Depending on the kind of analysis, the feasibility of a scenario is defined to be either:

- the dose that exactly satisfies the criteria targets,
- a range of a parameter that satisfies the criteria targets,
- or a region of a parameter grid that satisfies the criteria targets.

The model used in these analyses is: Monospecific Anti-Ligand - A biologic that binds to a soluble ligand target and prevents the ligand from binding to its cognate-receptor. The molecule can be mono- or bivalent. This is a one-compartment model and there is an option for +/- soluble receptor.

 © Applied BioMath LLC 2017-2021 

## Scenario Table

| Description | Route | Dose (mg) | Drug:Target KD (nM) | Molecular Weight (Da) | Biologic first order T 1/2 (days) | SC absorption T 1/2 (days) | Volume (L) | Effective Valency (undefined) | Ligand T 1/2 (min) | Receptor T 1/2 (min) | L:R Affinity (nM) | Ligand CSS (nM) | Receptor CSS (nM) | Scan Parameter | Criterion | Result |
|---|---|---|---|---|---|---|---|---|---|---|---|---|---|---|---|---|
| Nominal | SC | - | 0.0042 | 149000 | 14 | 2.5 | 5 | 1 | 30 | 540 | 0.019 | 0.0000575 | 0.23 | : Dose (mg) [1 - 50000] | Last Inhibition ≥ 90 % | Dose ≥ 444 mg |
| abs_half_high | SC | - | 0.0042 | 149000 | 14 | 0.83 | 5 | 1 | 30 | 540 | 0.019 | 0.0000575 | 0.23 | : Dose (mg) [1 - 50000] | Last Inhibition ≥ 90 % | Dose ≥ 509 mg |
| abs_half_low | SC | - | 0.0042 | 149000 | 14 | 7.5 | 5 | 1 | 30 | 540 | 0.019 | 0.0000575 | 0.23 | : Dose (mg) [1 - 50000] | Last Inhibition ≥ 90 % | Dose ≥ 270 mg |
| valency_high | SC | - | 0.0042 | 149000 | 14 | 2.5 | 5 | 2 | 30 | 540 | 0.019 | 0.0000575 | 0.23 | : Dose (mg) [1 - 50000] | Last Inhibition ≥ 90 % | Dose ≥ 222 mg |
| drug halflife_high | SC | - | 0.0042 | 149000 | 42 | 2.5 | 5 | 1 | 30 | 540 | 0.019 | 0.0000575 | 0.23 | : Dose (mg) [1 - 50000] | Last Inhibition ≥ 90 % | Dose ≥ 128 mg |
| drug halflife_low | SC | - | 0.0042 | 149000 | 4.67 | 2.5 | 5 | 1 | 30 | 540 | 0.019 | 0.0000575 | 0.23 | : Dose (mg) [1 - 50000] | Last Inhibition ≥ 90 % | Dose ≥ 4.00e+4 mg |
| TNF_conc_high | SC | - | 0.0042 | 149000 | 14 | 2.5 | 5 | 1 | 30 | 540 | 0.019 | 0.000173 | 0.23 | : Dose (mg) [1 - 50000] | Last Inhibition ≥ 90 % | Dose ≥ 446 mg |
| TNF_conc_low | SC | - | 0.0042 | 149000 | 14 | 2.5 | 5 | 1 | 30 | 540 | 0.019 | 0.0000192 | 0.23 | : Dose (mg) [1 - 50000] | Last Inhibition ≥ 90 % | Dose ≥ 444 mg |
| ligand_halflife_high | SC | - | 0.0042 | 149000 | 14 | 2.5 | 5 | 1 | 90 | 540 | 0.019 | 0.0000575 | 0.23 | : Dose (mg) [1 - 50000] | Last Inhibition ≥ 90 % | Dose ≥ 219 mg |
| ligand_halflife_low | SC | - | 0.0042 | 149000 | 14 | 2.5 | 5 | 1 | 10 | 540 | 0.019 | 0.0000575 | 0.23 | : Dose (mg) [1 - 50000] | Last Inhibition ≥ 90 % | Dose ≥ 1.12e+3 mg |


© Applied BioMath LLC 2017-2021

| | | | | | | | | | | | | | | | | |
|---|---|---|---|---|---|---|---|---|---|---|---|---|---|---|---|---|
| ligand:rec affinity high | SC | - | 0.0042 | 149000 | 14 | 2.5 | 5 | 1 | 30 | 540 | 0.006 | 0.0000575 | 0.23 | : Dose (mg) [1 - 50000] | Last Inhibition ≥ 90 % | Dose ≥ 492 mg |
| ligand:rec affinity low | SC | - | 0.0042 | 149000 | 14 | 2.5 | 5 | 1 | 30 | 540 | 0.057 | 0.0000575 | 0.23 | : Dose (mg) [1 - 50000] | Last Inhibition ≥ 90 % | Dose ≥ 395 mg |
| drug_affinity_high | SC | - | 0.0014 | 149000 | 14 | 2.5 | 5 | 1 | 30 | 540 | 0.019 | 0.0000575 | 0.23 | : Dose (mg) [1 - 50000] | Last Inhibition ≥ 90 % | Dose ≥ 182 mg |
| drug_affinity_low | SC | - | 0.0126 | 149000 | 14 | 2.5 | 5 | 1 | 30 | 540 | 0.019 | 0.0000575 | 0.23 | : Dose (mg) [1 - 50000] | Last Inhibition ≥ 90 % | Dose ≥ 1.23e+3 mg |
| receptor_conc_high | SC | - | 0.0042 | 149000 | 14 | 2.5 | 5 | 1 | 30 | 540 | 0.019 | 0.0000575 | 0.69 | : Dose (mg) [1 - 50000] | Last Inhibition ≥ 90 % | Dose ≥ 658 mg |
| receptor_conc_low | SC | - | 0.0042 | 149000 | 14 | 2.5 | 5 | 1 | 30 | 540 | 0.019 | 0.0000575 | 0.23 | : Dose (mg) [1 - 50000] | Last Inhibition ≥ 90 % | Dose ≥ 444 mg |
| rec_halflife_high | SC | - | 0.0042 | 149000 | 14 | 2.5 | 5 | 1 | 30 | 1620 | 0.019 | 0.0000575 | 0.23 | : Dose (mg) [1 - 50000] | Last Inhibition ≥ 90 % | Dose ≥ 390 mg |
| rec_halflife_low | SC | - | 0.0042 | 149000 | 14 | 2.5 | 5 | 1 | 30 | 180 | 0.019 | 0.0000575 | 0.23 | : Dose (mg) [1 - 50000] | Last Inhibition ≥ 90 % | Dose ≥ 496 mg |
| Volume_high | SC | - | 0.0042 | 149000 | 14 | 2.5 | 15 | 1 | 30 | 540 | 0.019 | 0.0000575 | 0.23 | : Dose (mg) [1 - 50000] | Last Inhibition ≥ 90 % | Dose ≥ 1.33e+3 mg |
| Volume_low | SC | - | 0.0042 | 149000 | 14 | 2.5 | 1.67 | 1 | 30 | 540 | 0.019 | 0.0000575 | 0.23 | : Dose (mg) [1 - 50000] | Last Inhibition ≥ 90 % | Dose ≥ 148 mg |
| MW_high | SC | - | 0.0042 | 447000 | 14 | 2.5 | 5 | 1 | 30 | 540 | 0.019 | 0.0000575 | 0.23 | : Dose (mg) [1 - 50000] | Last Inhibition ≥ 90 % | Dose ≥ 1.33e+3 mg |
| MW_low | SC | - | 0.0042 | 49666.67 | 14 | 2.5 | 5 | 1 | 30 | 540 | 0.019 | 0.0000575 | 0.23 | : Dose (mg) [1 - 50000] | Last Inhibition ≥ 90 % | Dose ≥ 148 mg |


© Applied BioMath LLC 2017-2021

# 2) Results

## 2.0) Scenario: Nominal

One dimensional scan over parameter, Dose, between 1 and 50000 using log spacing.

### 2.0.1) Model Parameters

Table 2.0.1

| Symbol | Parameter ID | Value | Unit |
|---|---|---|---|
| $\tau$ | interval | 60 | - |
| $D$ | dose | 100 | mg |
| $K_{D,\mathrm{L}}$ | mab_kd_1 | 0.0042 | nM |
| $N_{\mathrm{doses}}$ | dose_count | 7 | - |
| $MW$ | mw_1 | 149000 | Da |
| $t_{1/2}$ | el_half_1 | 14 | days |
| $t_{1/2,\mathrm{a}}$ | abs_half | 2.5 | days |
| $BW$ | BW | 70 | kg |
| $V$ | volume_central | 5 | L |
| $\mathrm{Valency}$ | drug_valency_1 | 1 | - |
| $t_{1/2,\mathrm{L}}$ | lig_half_1 | 30 | min |
| $t_{1/2,\mathrm{R}}$ | rec_half_1 | 540 | min |
| $t_{1/2,\mathrm{sR}}$ | shed_half_1 | 0.5 | hr |
| $K_{D,\mathrm{L:R}}$ | lig_rec_kd_1 | 0.019 | nM |
| $C_{SS,\mathrm{L}}$ | lig_css_1_central | 0.0000575 | nM |
| $C_{SS,\mathrm{R}}$ | rec_css_1_central | 0.23 | nM |
| $C_{SS,\mathrm{sR}}$ | shed_css_1_central | 0 | nM |
| $\mathrm{Density}_{\mathrm{cells}}$ | cell_density_mL_central | 1000000 | #/mL |

| Parameter ID | Value |
|---|---|
| Scan Parameter 1 | Dose |
| Lower Limit 1 | 1 |
| Upper Limit 1 | 50000 |
| N1 | 6 |
| Scale 1 | log |

 © Applied BioMath LLC 2017-2021 

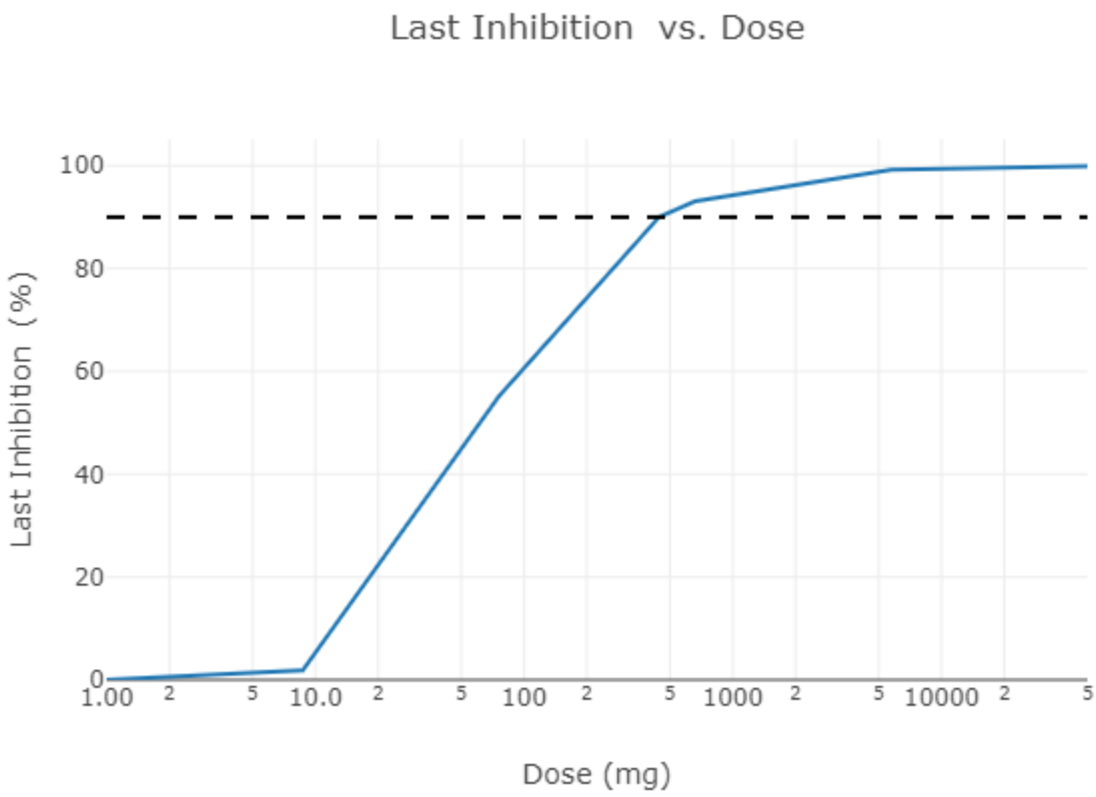


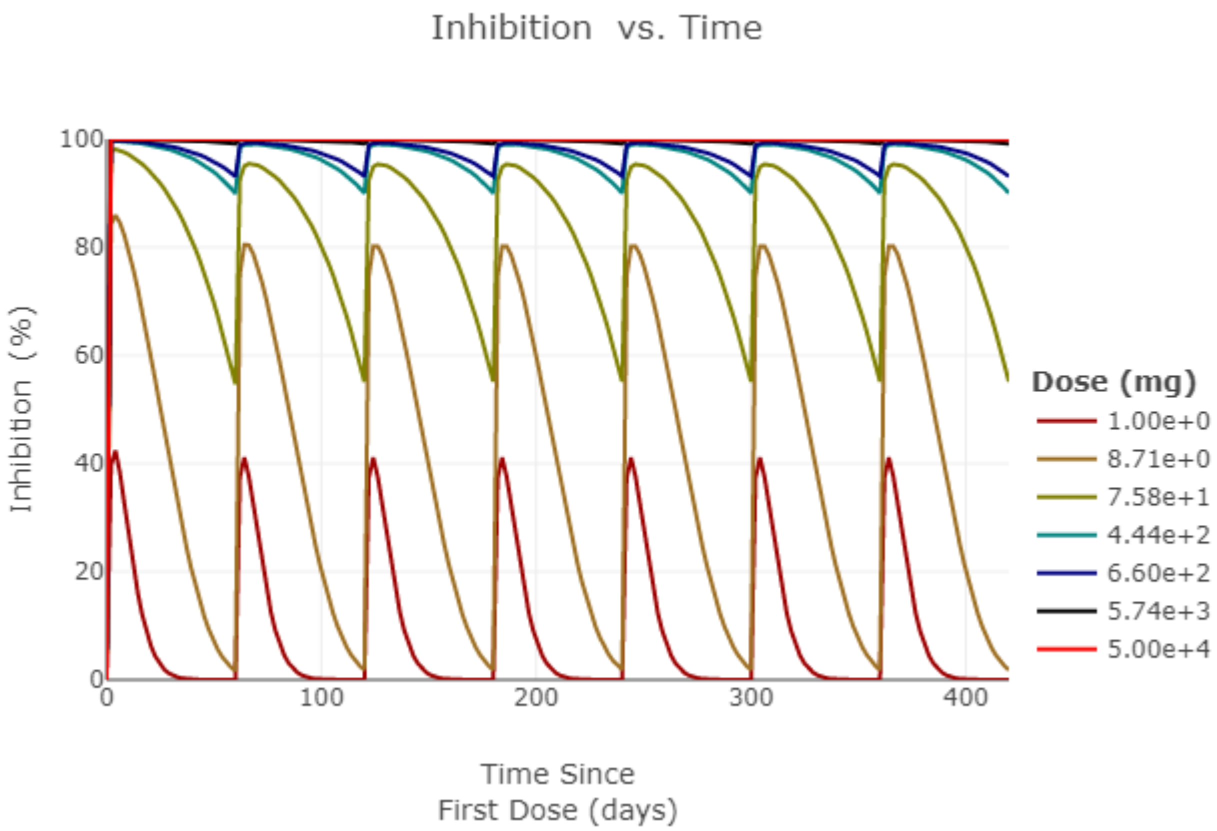


 © Applied BioMath LLC 2017-2021 

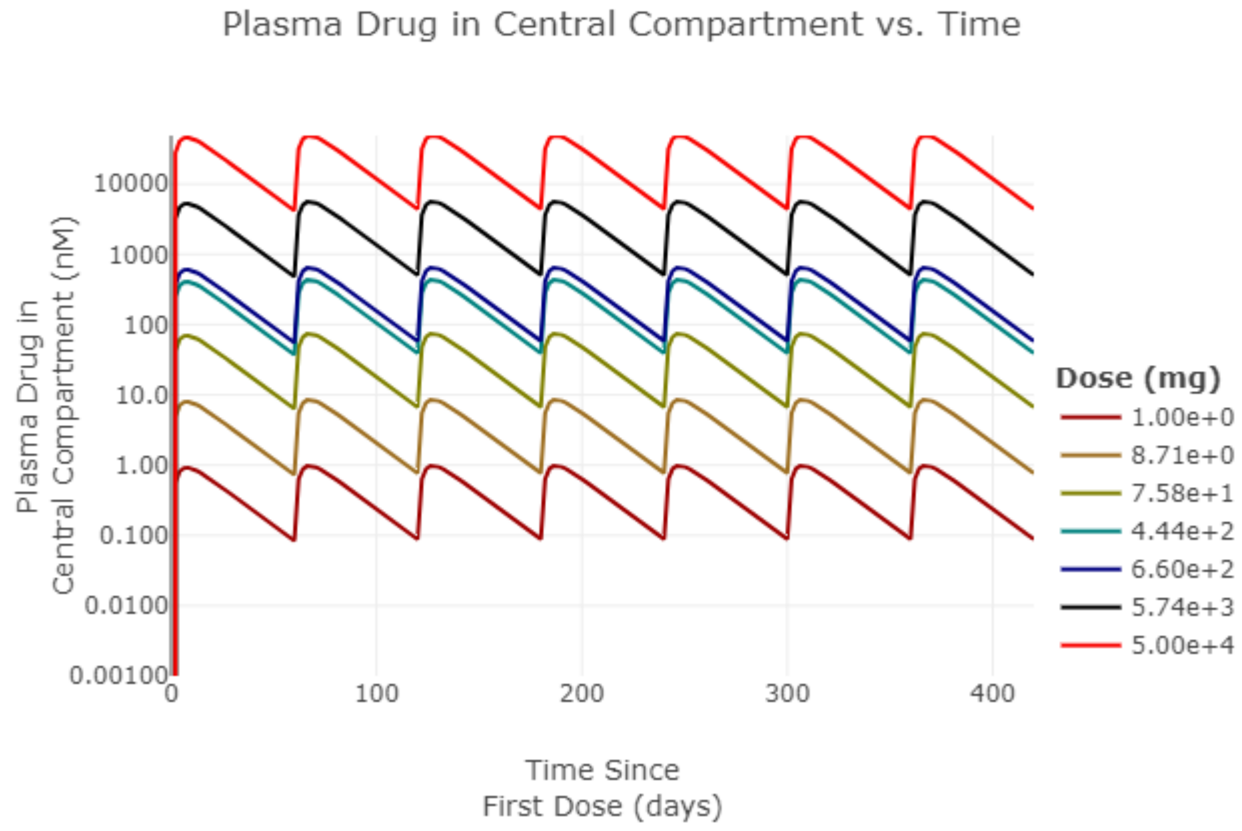


## 2.1) Scenario: abs_half_high

One dimensional scan over parameter, Dose, between 1 and 50000 using log spacing.

### 2.1.1) Model Parameters

Table 2.1.1

| Symbol | Parameter ID | Value | Unit |
| --- | --- | --- | --- |
| $\tau$ | interval | 60 | - |
| $D$ | dose | 100 | mg |
| $K_{D,\mathrm{L}}$ | mab_kd_1 | 0.0042 | nM |
| $N_{\mathrm{doses}}$ | dose_count | 7 | - |
| $MW$ | mw_1 | 149000 | Da |
| $t_{1/2}$ | el_half_1 | 14 | days |
| $t_{1/2,\mathrm{a}}$ | abs_half | 0.83 | days |
| $BW$ | BW | 70 | kg |
| $V$ | volume_central | 5 | L |
| Valency | drug_valency_1 | 1 | - |
| $t_{1/2,\mathrm{L}}$ | lig_half_1 | 30 | min |
| $t_{1/2,\mathrm{R}}$ | rec_half_1 | 540 | min |

| | | | |
|---|---|---|---|
| $t_{1/2,\mathrm{sR}}$ | shed_half_1 | 0.5 | hr |
| $K_{D,\mathrm{L:R}}$ | lig_rec_kd_1 | 0.019 | nM |
| $C_{SS,\mathrm{L}}$ | lig_css_1_central | 0.0000575 | nM |
| $C_{SS,\mathrm{R}}$ | rec_css_1_central | 0.23 | nM |
| $C_{SS,\mathrm{sR}}$ | shed_css_1_central | 0 | nM |
| $\mathrm{Density}_{\mathrm{cells}}$ | cell_density_mL_central | 1000000 | #/mL |

| Parameter ID | Value |
|---|---|
| Scan Parameter 1 | Dose |
| Lower Limit 1 | 1 |
| Upper Limit 1 | 50000 |
| N1 | 6 |
| Scale 1 | log |

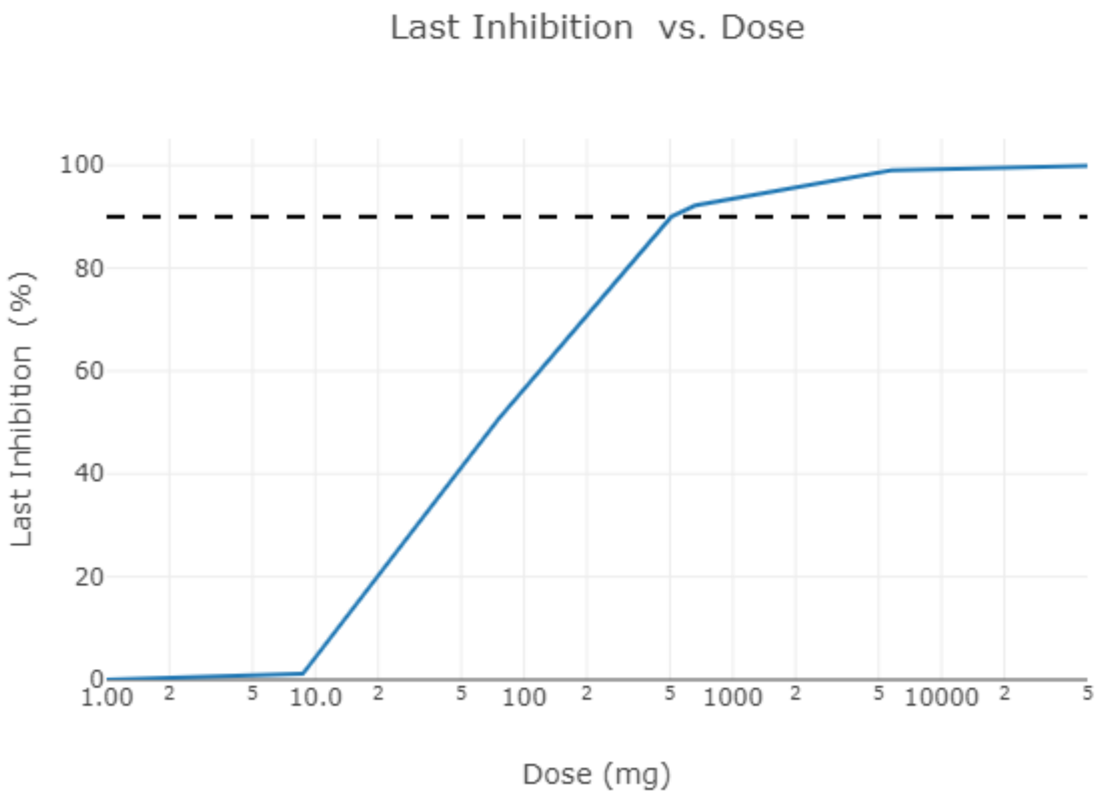


 © Applied BioMath LLC 2017-2021 

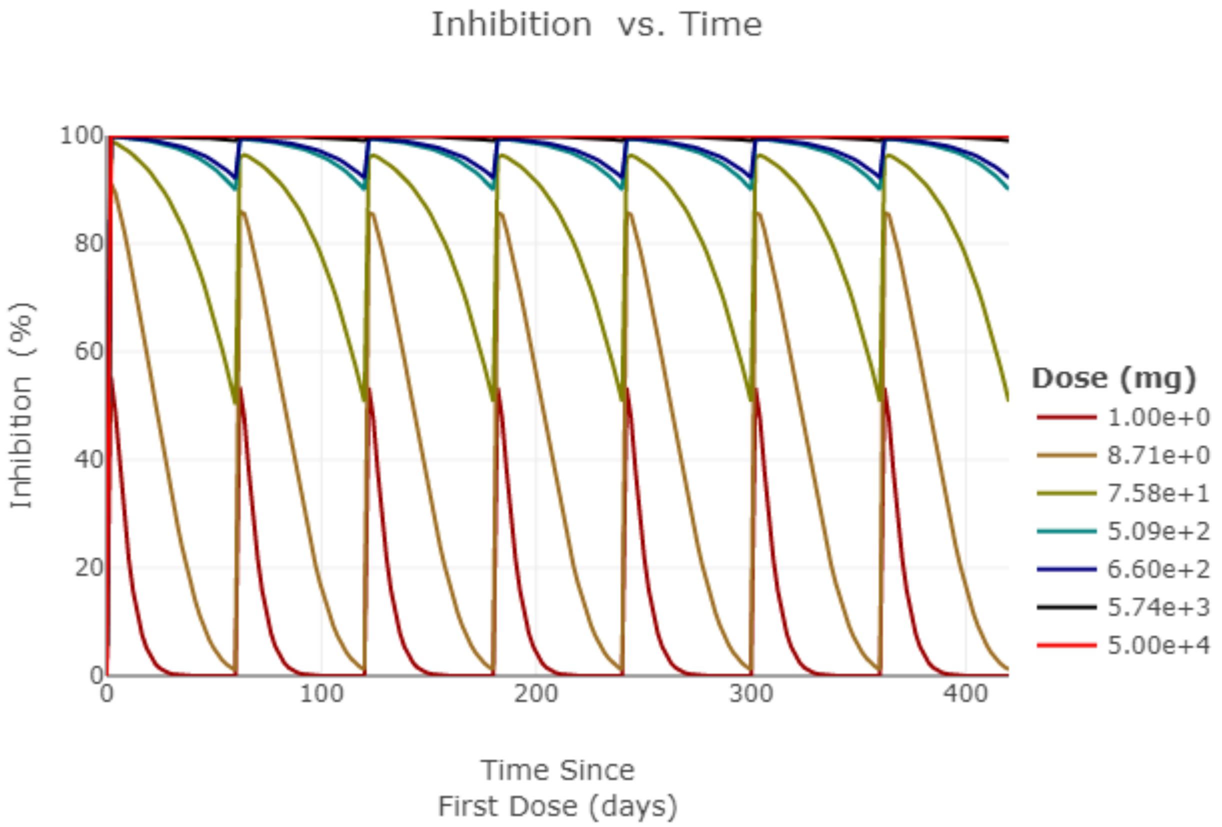

Inhibition vs. Time
Inhibition (%)
100
80
60
40
20
0
0
100
200
300
400
Time Since
First Dose (days)
Dose (mg)
1.00e+0
8.71e+0
7.58e+1
5.09e+2
6.60e+2
5.74e+3
5.00e+4


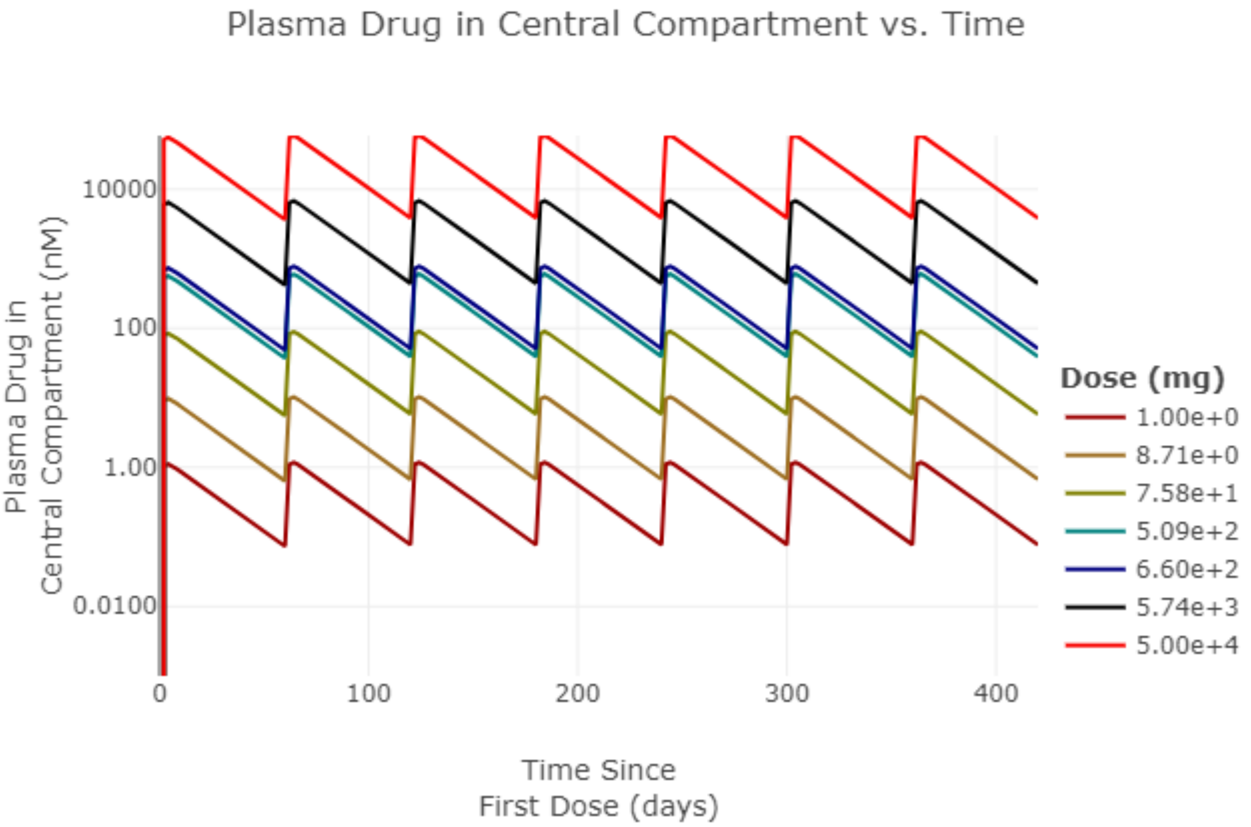

Plasma Drug in Central Compartment vs. Time
Plasma Drug in
Central Compartment (nM)
10000
100
1.00
0.0100
0
100
200
300
400
Time Since
First Dose (days)
Dose (mg)
1.00e+0
8.71e+0
7.58e+1
5.09e+2
6.60e+2
5.74e+3
5.00e+4

## 2.2) Scenario: abs_half_low

One dimensional scan over parameter, Dose, between 1 and 50000 using log spacing.

### 2.2.1) Model Parameters

Table 2.2.1

| Symbol | Parameter ID | Value | Unit |
|---|---|---|---|
| $\tau$ | interval | 60 | - |
| $D$ | dose | 100 | mg |
| $K_{D,\mathrm{L}}$ | mab_kd_1 | 0.0042 | nM |
| $N_{\mathrm{doses}}$ | dose_count | 7 | - |
| $MW$ | mw_1 | 149000 | Da |
| $t_{1/2}$ | el_half_1 | 14 | days |
| $t_{1/2,\mathrm{a}}$ | abs_half | 7.5 | days |
| $BW$ | BW | 70 | kg |
| $V$ | volume_central | 5 | L |
| $\mathrm{Valency}$ | drug_valency_1 | 1 | - |
| $t_{1/2,\mathrm{L}}$ | lig_half_1 | 30 | min |
| $t_{1/2,\mathrm{R}}$ | rec_half_1 | 540 | min |
| $t_{1/2,\mathrm{sR}}$ | shed_half_1 | 0.5 | hr |
| $K_{D,\mathrm{L:R}}$ | lig_rec_kd_1 | 0.019 | nM |
| $C_{ss,\mathrm{L}}$ | lig_css_1_central | 0.0000575 | nM |
| $C_{ss,\mathrm{R}}$ | rec_css_1_central | 0.23 | nM |
| $C_{ss,\mathrm{sR}}$ | shed_css_1_central | 0 | nM |
| $\mathrm{Density}_{\mathrm{cells}}$ | cell_density_mL_central | 1000000 | #/mL |

| Parameter ID | Value |
|---|---|
| Scan Parameter 1 | Dose |
| Lower Limit 1 | 1 |
| Upper Limit 1 | 50000 |
| N1 | 6 |
| Scale 1 | log |

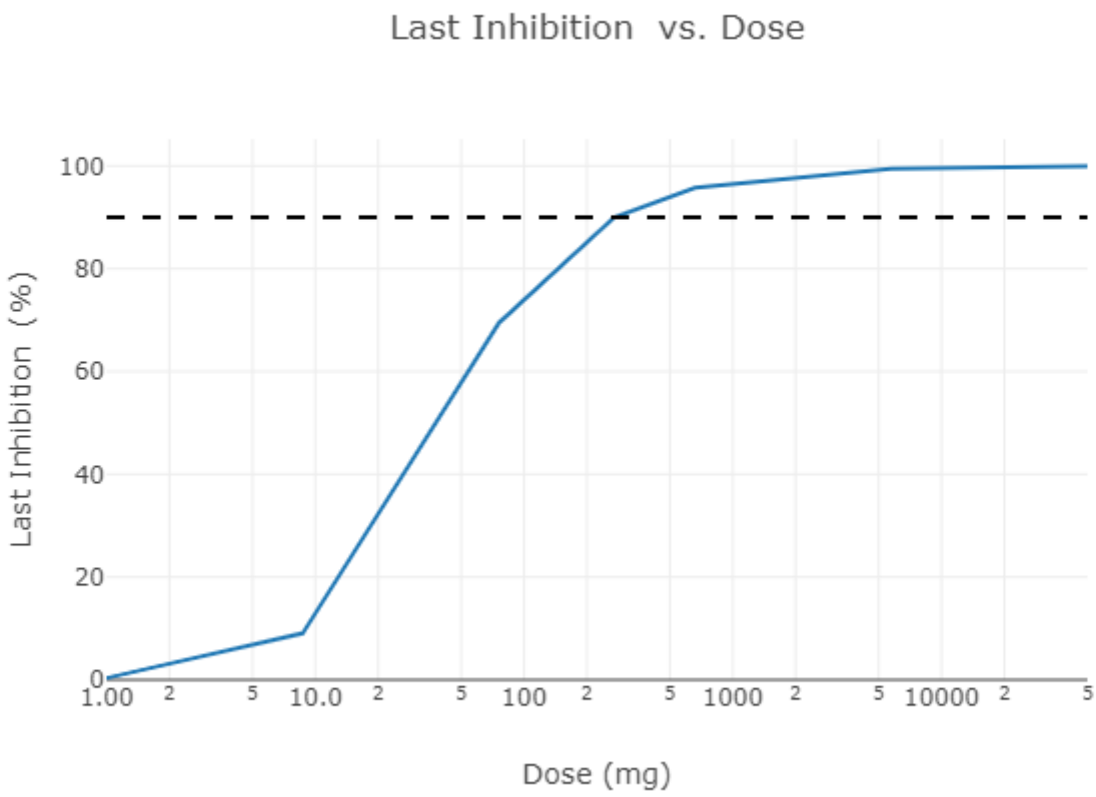
Last Inhibition vs. Dose
Last Inhibition (%)
100
80
60
40
20
0
1.00
2
5
10.0
100
1000
10000
Dose (mg)

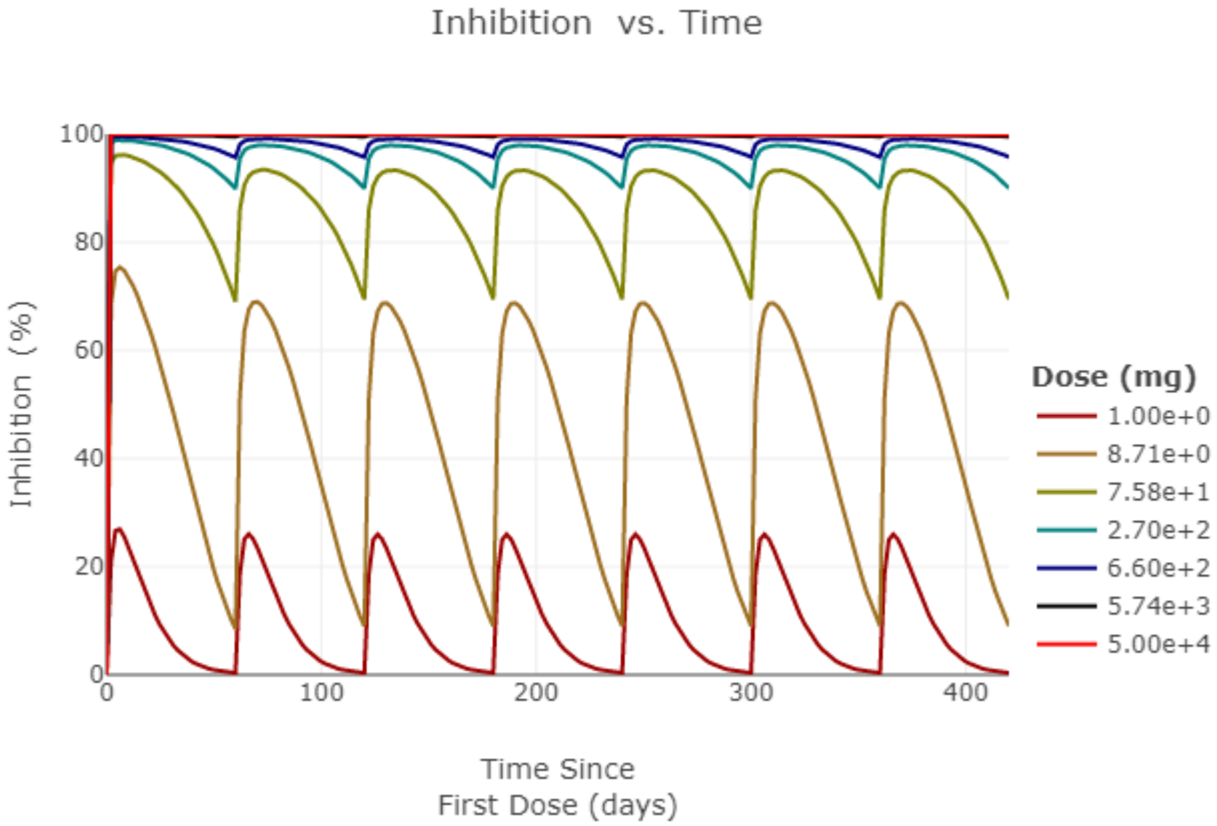
Inhibition vs. Time
Inhibition (%)
100
80
60
40
20
0
0
100
200
300
400
Time Since
First Dose (days)
Dose (mg)
1.00e+0
8.71e+0
7.58e+1
2.70e+2
6.60e+2
5.74e+3
5.00e+4

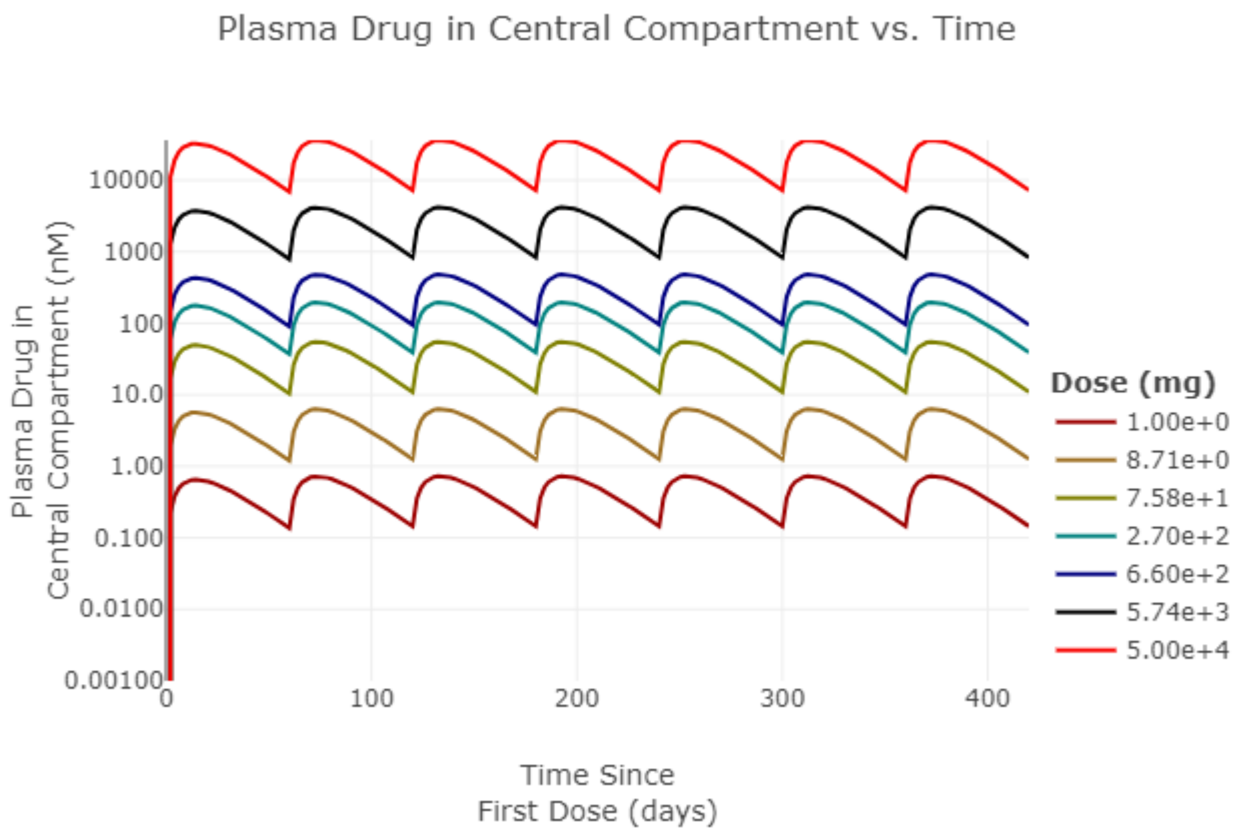


## 2.3) Scenario: valency_high

One dimensional scan over parameter, Dose, between 1 and 50000 using log spacing.

### 2.3.1) Model Parameters

Table 2.3.1

| Symbol | Parameter ID | Value | Unit |
|---|---|---|---|
| $\tau$ | interval | 60 | - |
| $D$ | dose | 100 | mg |
| $K_{D,\mathrm{L}}$ | mab_kd_1 | 0.0042 | nM |
| $N_{\mathrm{doses}}$ | dose_count | 7 | - |
| $MW$ | mw_1 | 149000 | Da |
| $t_{1/2}$ | el_half_1 | 14 | days |
| $t_{1/2,\mathrm{a}}$ | abs_half | 2.5 | days |
| $BW$ | BW | 70 | kg |
| $V$ | volume_central | 5 | L |
| Valency | drug_valency_1 | 2 | - |
| $t_{1/2,\mathrm{L}}$ | lig_half_1 | 30 | min |
| $t_{1/2,\mathrm{R}}$ | rec_half_1 | 540 | min |

 © Applied BioMath LLC 2017-2021 

| | | | |
|---|---|---|---|
| $t_{1/2,\mathrm{sR}}$ | shed_half_1 | 0.5 | hr |
| $K_{D,\mathrm{L:R}}$ | lig_rec_kd_1 | 0.019 | nM |
| $C_{ss,\mathrm{L}}$ | lig_css_1_central | 0.0000575 | nM |
| $C_{ss,\mathrm{R}}$ | rec_css_1_central | 0.23 | nM |
| $C_{ss,\mathrm{sR}}$ | shed_css_1_central | 0 | nM |
| $\mathrm{Density}_{\mathrm{cells}}$ | cell_density_mL_central | 1000000 | #/mL |

| Parameter ID | Value |
|---|---|
| Scan Parameter 1 | Dose |
| Lower Limit 1 | 1 |
| Upper Limit 1 | 50000 |
| N1 | 6 |
| Scale 1 | log |

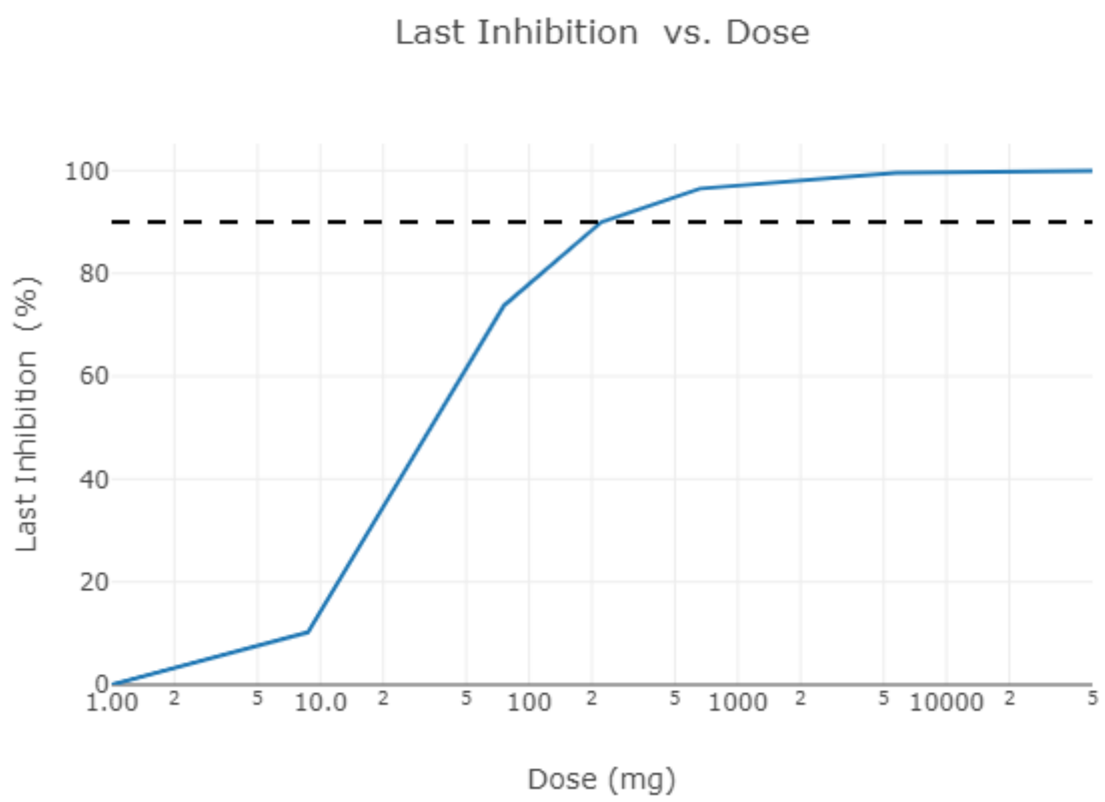

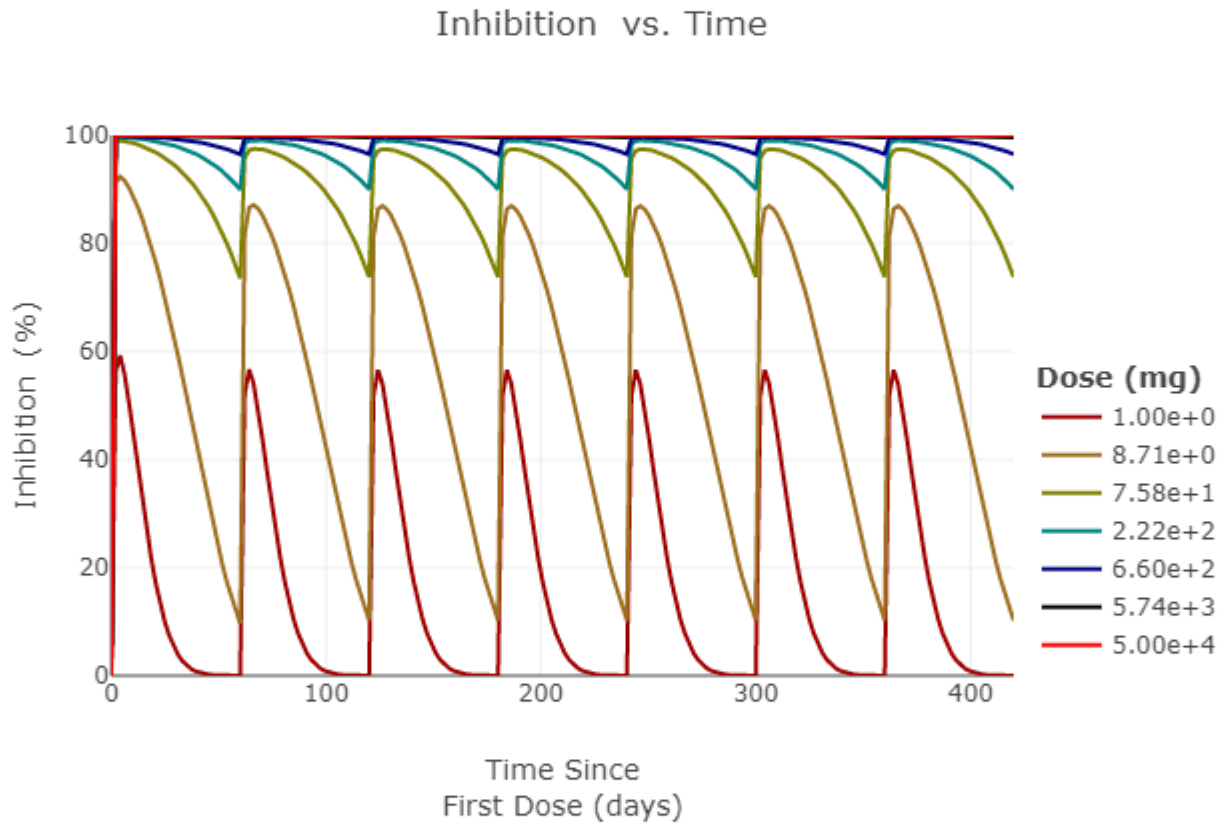


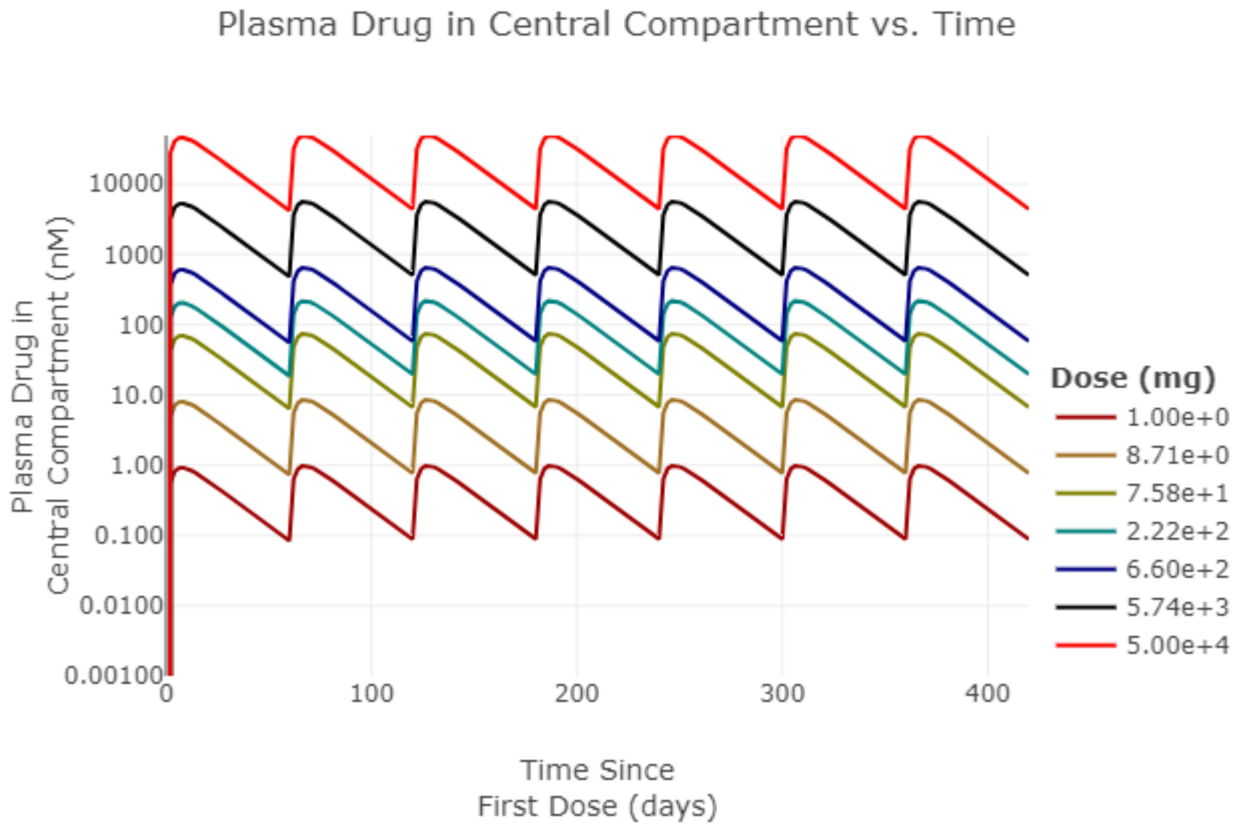


 © Applied BioMath LLC 2017-2021 

## 2.4) Scenario: drug halflife_high

One dimensional scan over parameter, Dose, between 1 and 50000 using log spacing.

### 2.4.1) Model Parameters

Table 2.4.1

| Symbol | Parameter ID | Value | Unit |
|---|---|---|---|
| $\tau$ | interval | 60 | - |
| $D$ | dose | 100 | mg |
| $K_{D,\mathrm{L}}$ | mab_kd_1 | 0.0042 | nM |
| $N_{\mathrm{doses}}$ | dose_count | 7 | - |
| $MW$ | mw_1 | 149000 | Da |
| $t_{1/2}$ | el_half_1 | 42 | days |
| $t_{1/2,\mathrm{a}}$ | abs_half | 2.5 | days |
| $BW$ | BW | 70 | kg |
| $V$ | volume_central | 5 | L |
| $\mathrm{Valency}$ | drug_valency_1 | 1 | - |
| $t_{1/2,\mathrm{L}}$ | lig_half_1 | 30 | min |
| $t_{1/2,\mathrm{R}}$ | rec_half_1 | 540 | min |
| $t_{1/2,\mathrm{sR}}$ | shed_half_1 | 0.5 | hr |
| $K_{D,\mathrm{L:R}}$ | lig_rec_kd_1 | 0.019 | nM |
| $C_{SS,\mathrm{L}}$ | lig_css_1_central | 0.0000575 | nM |
| $C_{SS,\mathrm{R}}$ | rec_css_1_central | 0.23 | nM |
| $C_{SS,\mathrm{sR}}$ | shed_css_1_central | 0 | nM |
| $\mathrm{Density}_{\mathrm{cells}}$ | cell_density_mL_central | 1000000 | #/mL |

| Parameter ID | Value |
|---|---|
| Scan Parameter 1 | Dose |
| Lower Limit 1 | 1 |
| Upper Limit 1 | 50000 |
| N1 | 6 |
| Scale 1 | log |

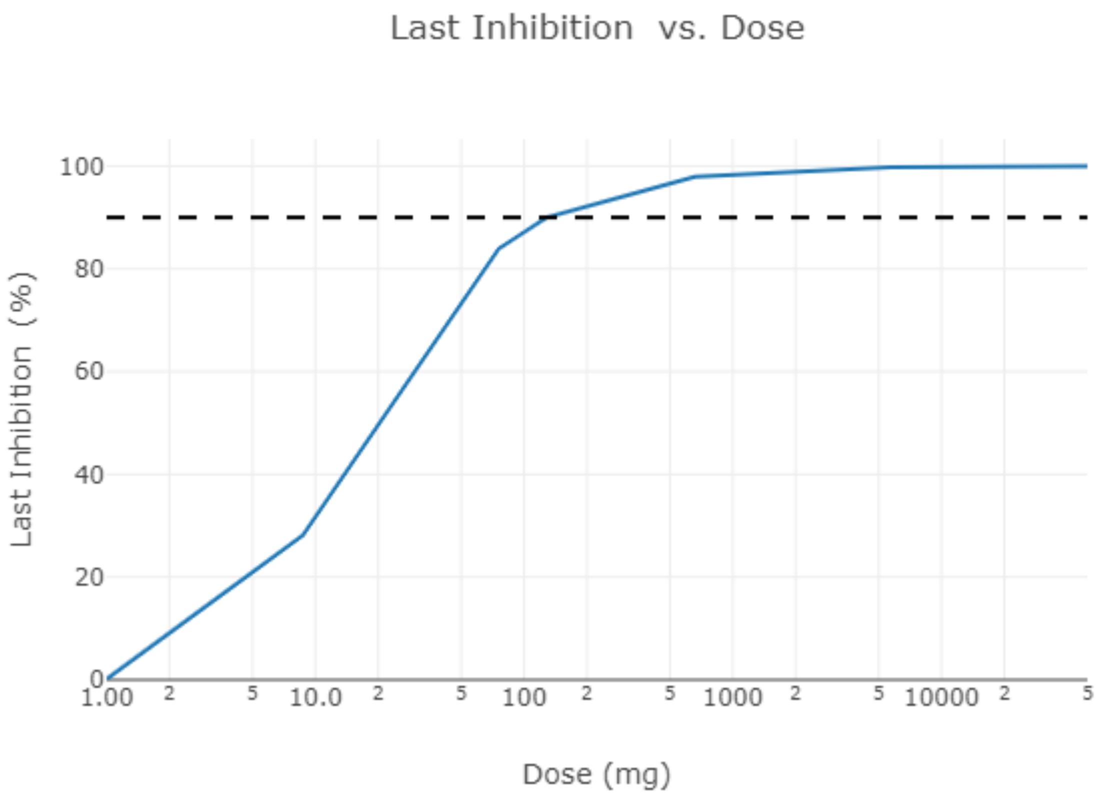
Last Inhibition vs. Dose
Last Inhibition (%)
0
20
40
60
80
100
1.00
10.0
100
1000
10000
Dose (mg)

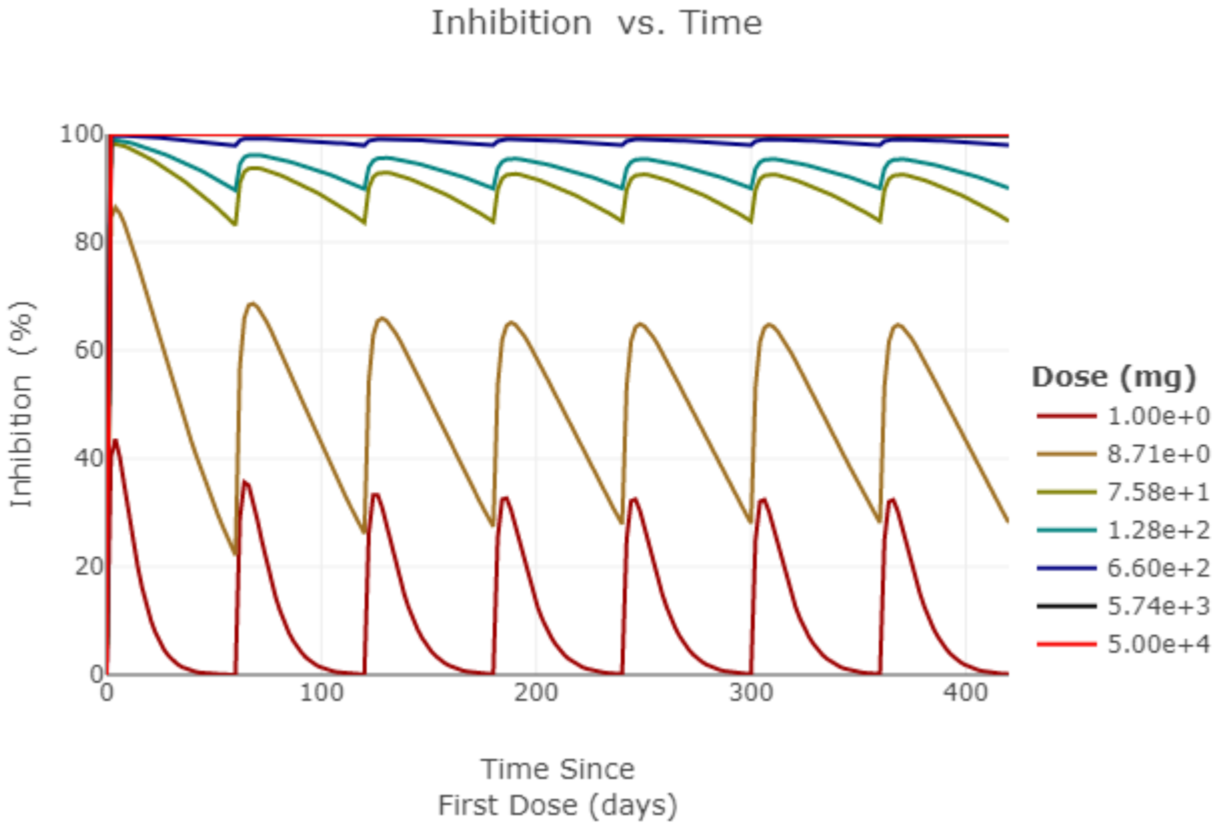
Inhibition vs. Time
Inhibition (%)
0
20
40
60
80
100
0
100
200
300
400
Time Since
First Dose (days)
Dose (mg)
1.00e+0
8.71e+0
7.58e+1
1.28e+2
6.60e+2
5.74e+3
5.00e+4

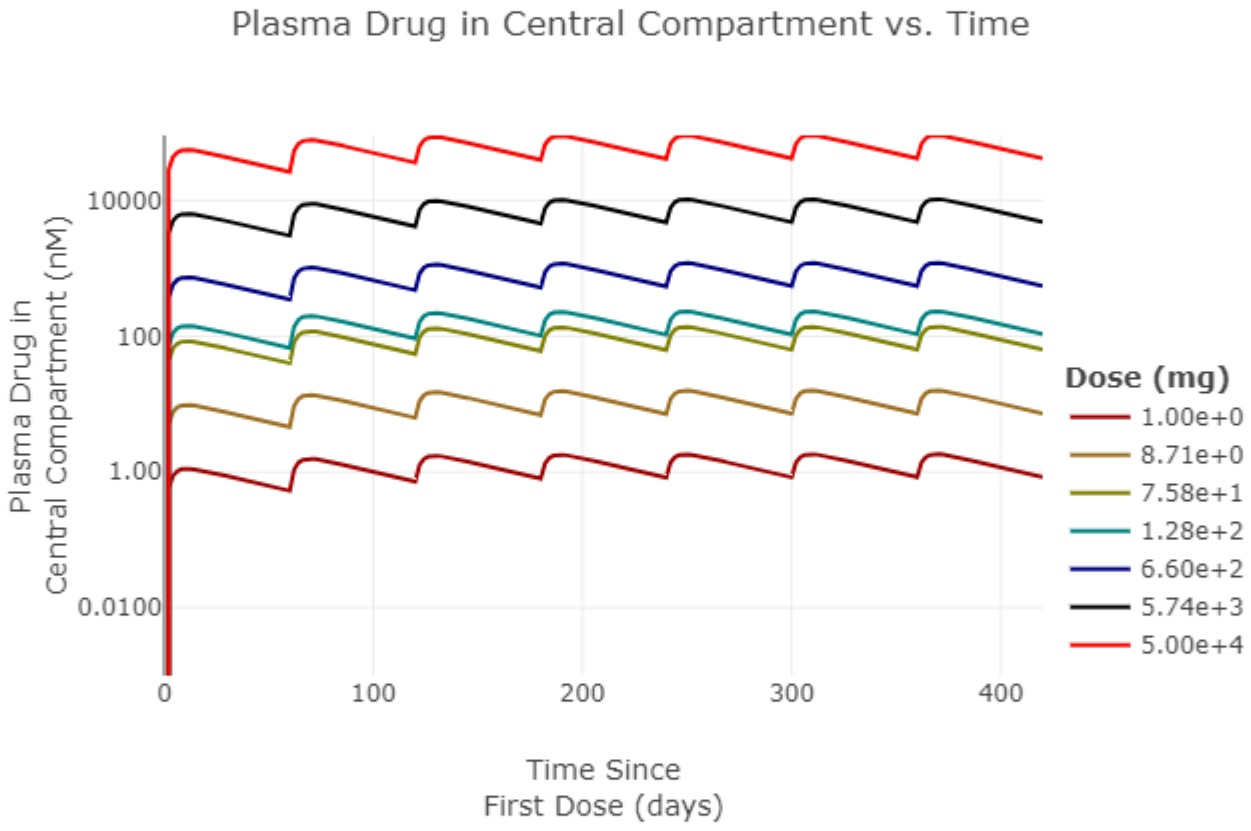


## 2.5) Scenario: drug halflife_low

One dimensional scan over parameter, Dose, between 1 and 50000 using log spacing.

### 2.5.1) Model Parameters

Table 2.5.1

| Symbol | Parameter ID | Value | Unit |
|---|---|---|---|
| $\tau$ | interval | 60 | - |
| $D$ | dose | 100 | mg |
| $K_{D,\mathrm{L}}$ | mab_kd_1 | 0.0042 | nM |
| $N_{\mathrm{doses}}$ | dose_count | 7 | - |
| $MW$ | mw_1 | 149000 | Da |
| $t_{1/2}$ | el_half_1 | 4.67 | days |
| $t_{1/2,\mathrm{a}}$ | abs_half | 2.5 | days |
| $BW$ | BW | 70 | kg |
| $V$ | volume_central | 5 | L |
| Valency | drug_valency_1 | 1 | - |
| $t_{1/2,\mathrm{L}}$ | lig_half_1 | 30 | min |
| $t_{1/2,\mathrm{R}}$ | rec_half_1 | 540 | min |

 © Applied BioMath LLC 2017-2021 

| | | | |
|---|---|---|---|
| $t_{1/2,\mathrm{sR}}$ | shed_half_1 | 0.5 | hr |
| $K_{D,\mathrm{L:R}}$ | lig_rec_kd_1 | 0.019 | nM |
| $C_{SS,\mathrm{L}}$ | lig_css_1_central | 0.0000575 | nM |
| $C_{SS,\mathrm{R}}$ | rec_css_1_central | 0.23 | nM |
| $C_{SS,\mathrm{sR}}$ | shed_css_1_central | 0 | nM |
| $\mathrm{Density}_{\mathrm{cells}}$ | cell_density_mL_central | 1000000 | #/mL |

| Parameter ID | Value |
|---|---|
| Scan Parameter 1 | Dose |
| Lower Limit 1 | 1 |
| Upper Limit 1 | 50000 |
| N1 | 6 |
| Scale 1 | log |

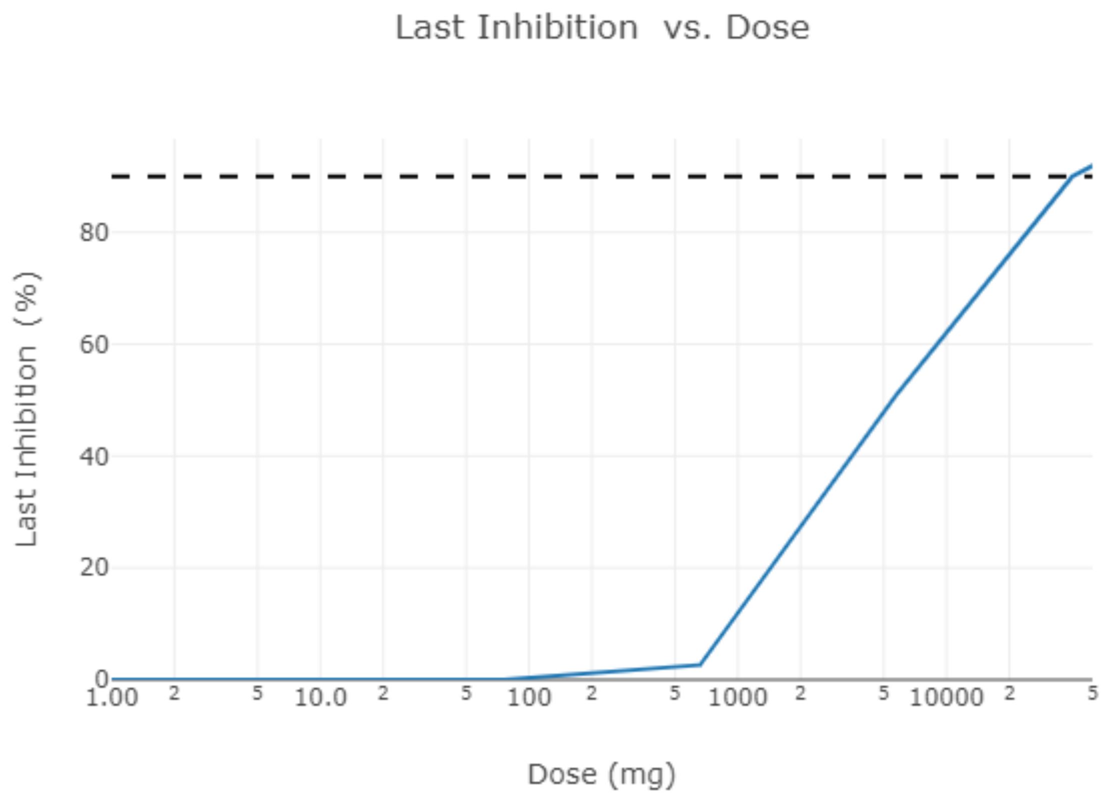

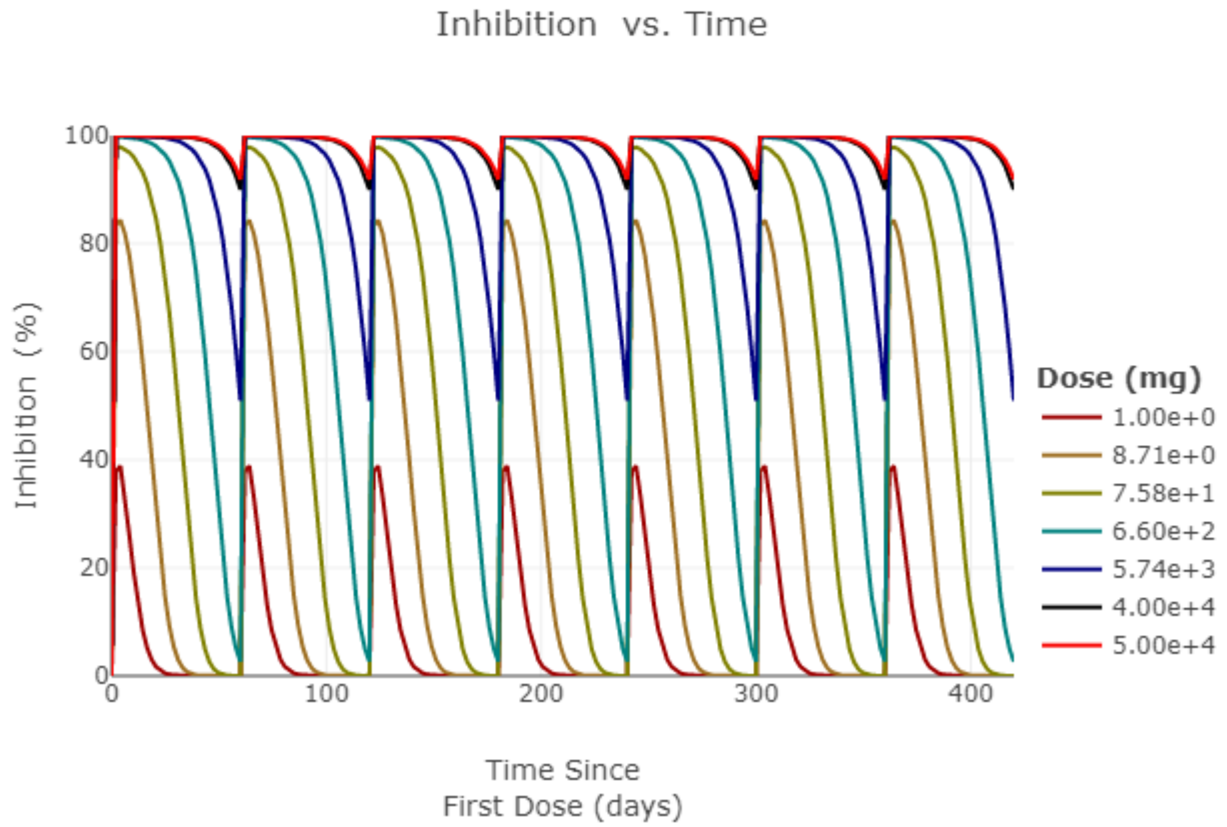


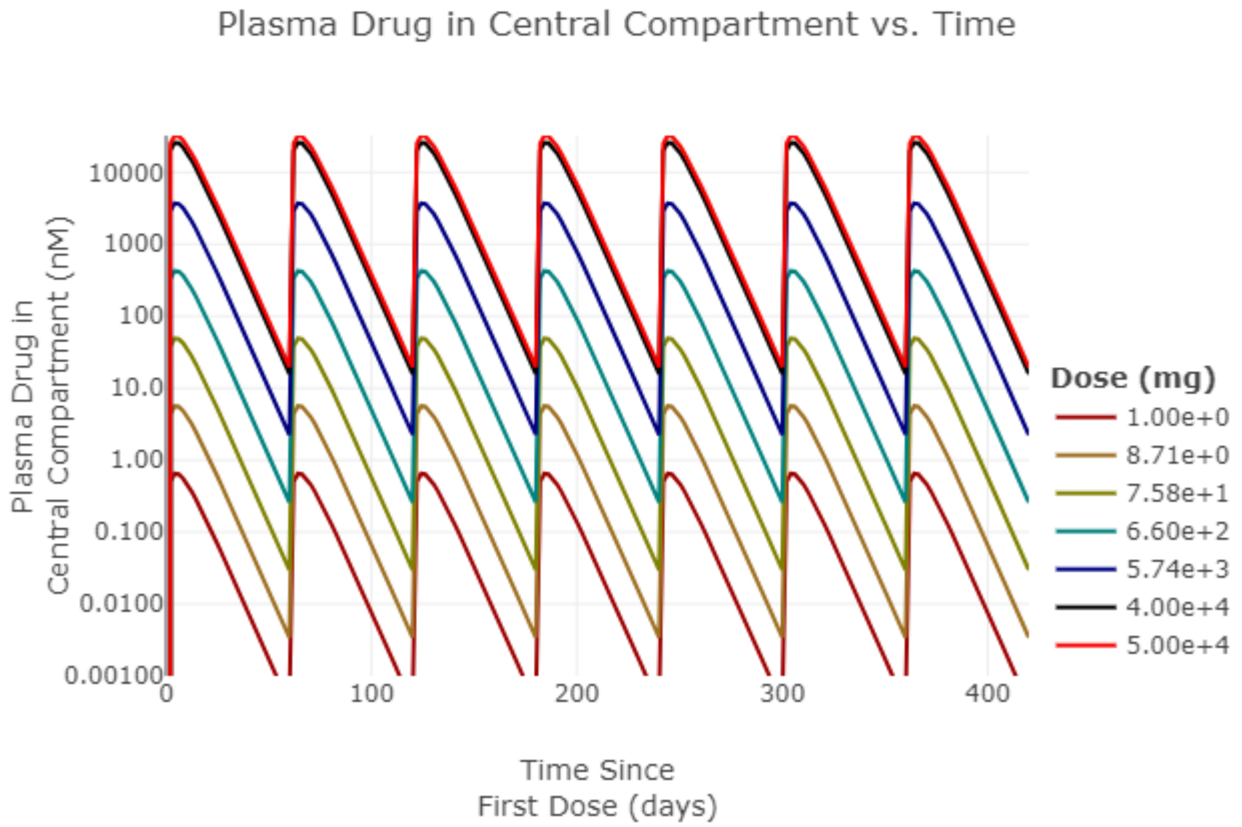


 © Applied BioMath LLC 2017-2021 

# 2.6) Scenario: TNF_conc_high

One dimensional scan over parameter, Dose, between 1 and 50000 using log spacing.

## 2.6.1) Model Parameters

Table 2.6.1

| Symbol | Parameter ID | Value | Unit |
|---|---|---|---|
| $\tau$ | interval | 60 | - |
| $D$ | dose | 100 | mg |
| $K_{D,\mathrm{L}}$ | mab_kd_1 | 0.0042 | nM |
| $N_{\mathrm{doses}}$ | dose_count | 7 | - |
| $MW$ | mw_1 | 149000 | Da |
| $t_{1/2}$ | el_half_1 | 14 | days |
| $t_{1/2,\mathrm{a}}$ | abs_half | 2.5 | days |
| $BW$ | BW | 70 | kg |
| $V$ | volume_central | 5 | L |
| $\mathrm{Valency}$ | drug_valency_1 | 1 | - |
| $t_{1/2,\mathrm{L}}$ | lig_half_1 | 30 | min |
| $t_{1/2,\mathrm{R}}$ | rec_half_1 | 540 | min |
| $t_{1/2,\mathrm{sR}}$ | shed_half_1 | 0.5 | hr |
| $K_{D,\mathrm{L:R}}$ | lig_rec_kd_1 | 0.019 | nM |
| $C_{ss,\mathrm{L}}$ | lig_css_1_central | 0.000173 | nM |
| $C_{ss,\mathrm{R}}$ | rec_css_1_central | 0.23 | nM |
| $C_{ss,\mathrm{sR}}$ | shed_css_1_central | 0 | nM |
| $\mathrm{Density}_{\mathrm{cells}}$ | cell_density_mL_central | 1000000 | #/mL |

| Parameter ID | Value |
|---|---|
| Scan Parameter 1 | Dose |
| Lower Limit 1 | 1 |
| Upper Limit 1 | 50000 |
| N1 | 6 |
| Scale 1 | log |

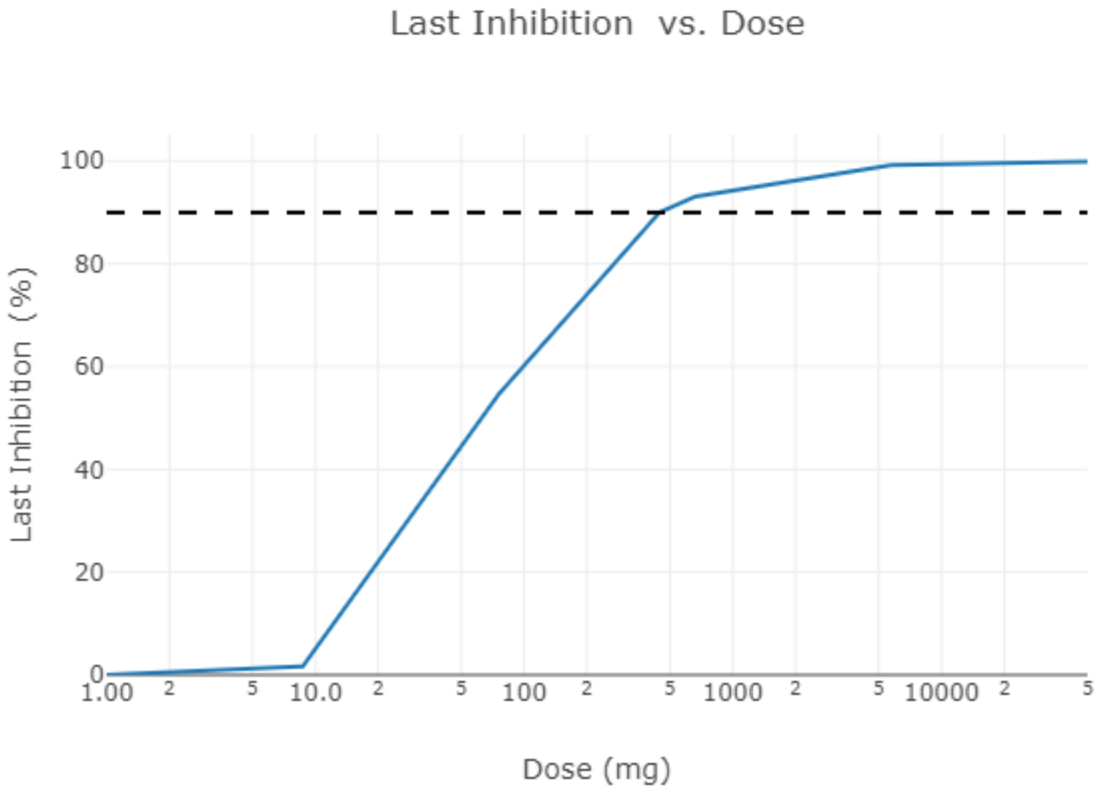

Last Inhibition vs. Dose
100
80
60
40
20
0
Last Inhibition (%)
1.00
2
5
10.0
2
5
100
2
5
1000
2
5
10000
2
5
Dose (mg)


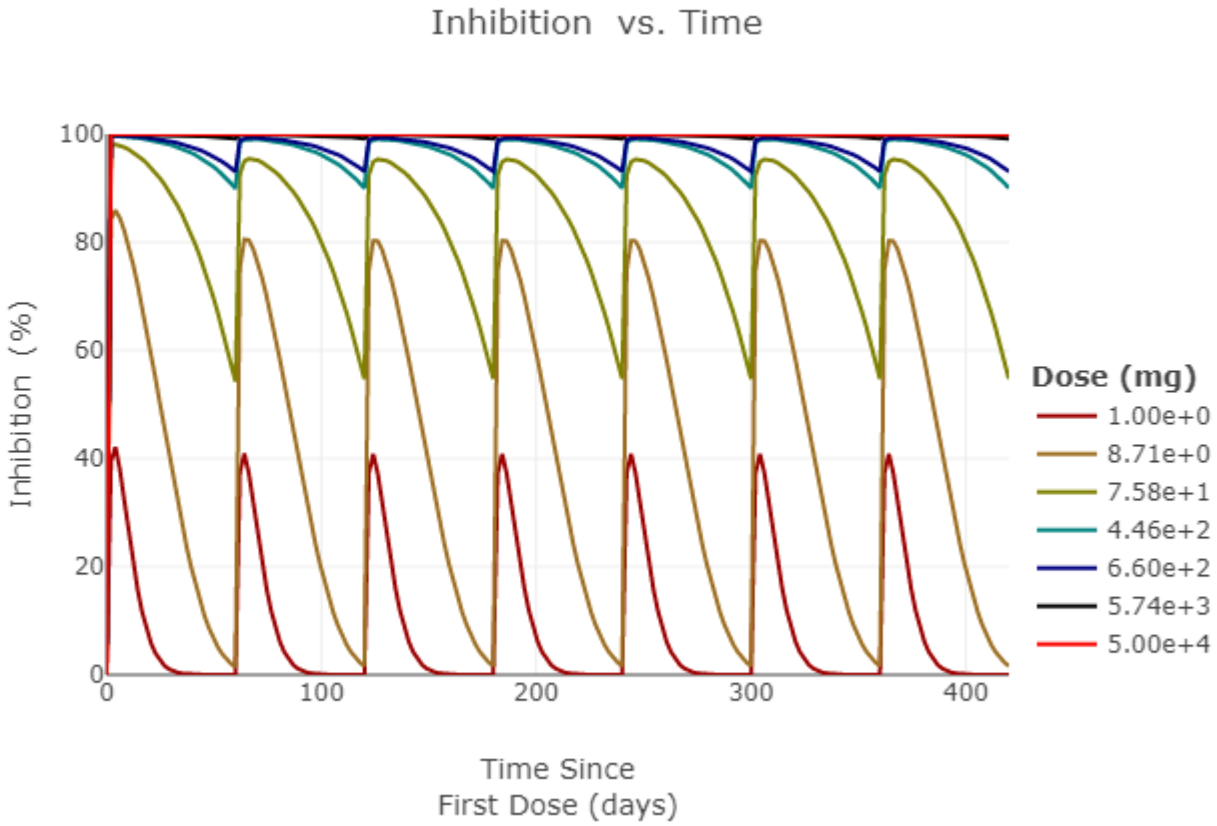

Inhibition vs. Time
100
80
60
40
20
0
Inhibition (%)
0
100
200
300
400
Time Since
First Dose (days)
Dose (mg)
1.00e+0
8.71e+0
7.58e+1
4.46e+2
6.60e+2
5.74e+3
5.00e+4

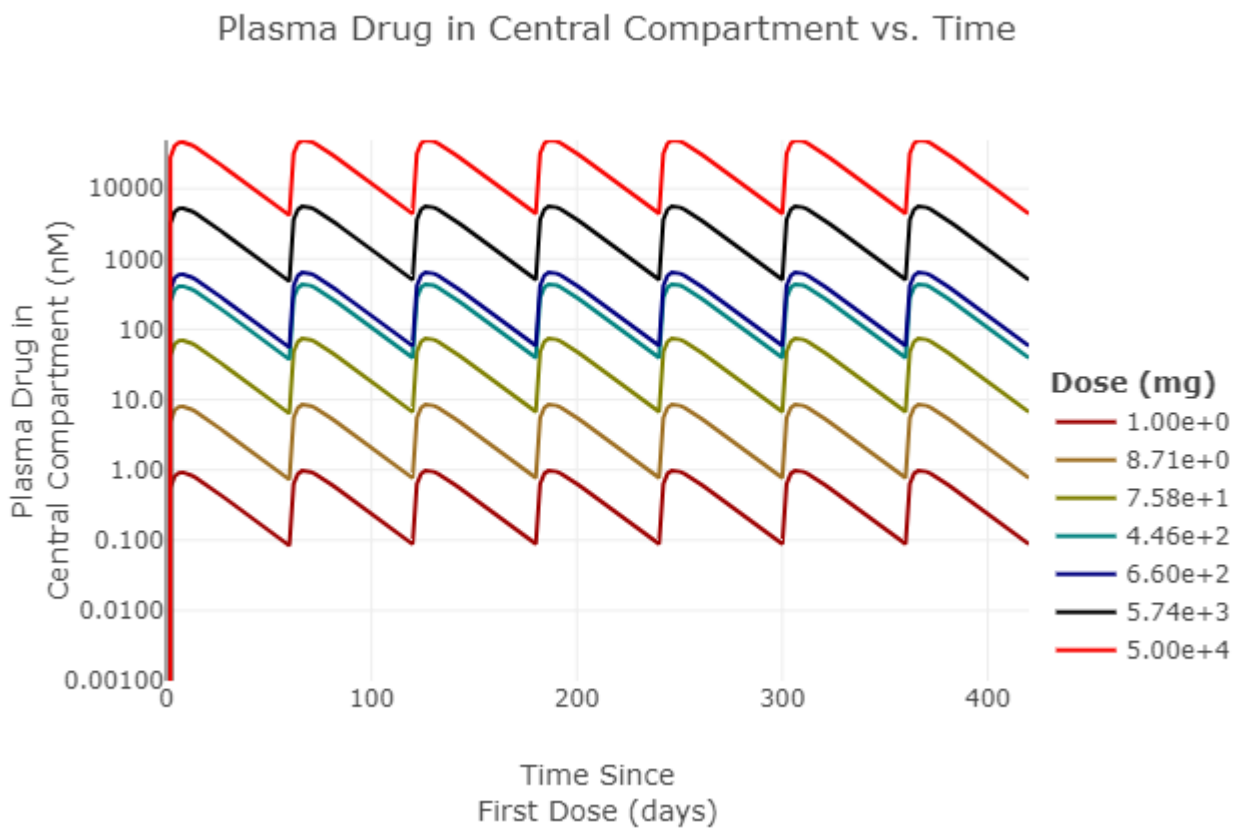


## 2.7) Scenario: TNF_conc_low

One dimensional scan over parameter, Dose, between 1 and 50000 using log spacing.

### 2.7.1) Model Parameters

Table 2.7.1

| Symbol | Parameter ID | Value | Unit |
|---|---|---|---|
| $\tau$ | interval | 60 | - |
| $D$ | dose | 100 | mg |
| $K_{D,\mathrm{L}}$ | mab_kd_1 | 0.0042 | nM |
| $N_{\mathrm{doses}}$ | dose_count | 7 | - |
| $MW$ | mw_1 | 149000 | Da |
| $t_{1/2}$ | el_half_1 | 14 | days |
| $t_{1/2,\mathrm{a}}$ | abs_half | 2.5 | days |
| $BW$ | BW | 70 | kg |
| $V$ | volume_central | 5 | L |
| Valency | drug_valency_1 | 1 | - |
| $t_{1/2,\mathrm{L}}$ | lig_half_1 | 30 | min |
| $t_{1/2,\mathrm{R}}$ | rec_half_1 | 540 | min |

| | | | |
|---|---|---|---|
| $t_{1/2,\mathrm{sR}}$ | shed_half_1 | 0.5 | hr |
| $K_{D,\mathrm{L:R}}$ | lig_rec_kd_1 | 0.019 | nM |
| $C_{SS,\mathrm{L}}$ | lig_css_1_central | 0.0000192 | nM |
| $C_{SS,\mathrm{R}}$ | rec_css_1_central | 0.23 | nM |
| $C_{SS,\mathrm{sR}}$ | shed_css_1_central | 0 | nM |
| $\mathrm{Density}_{\mathrm{cells}}$ | cell_density_mL_central | 1000000 | #/mL |

| Parameter ID | Value |
|---|---|
| Scan Parameter 1 | Dose |
| Lower Limit 1 | 1 |
| Upper Limit 1 | 50000 |
| N1 | 6 |
| Scale 1 | log |

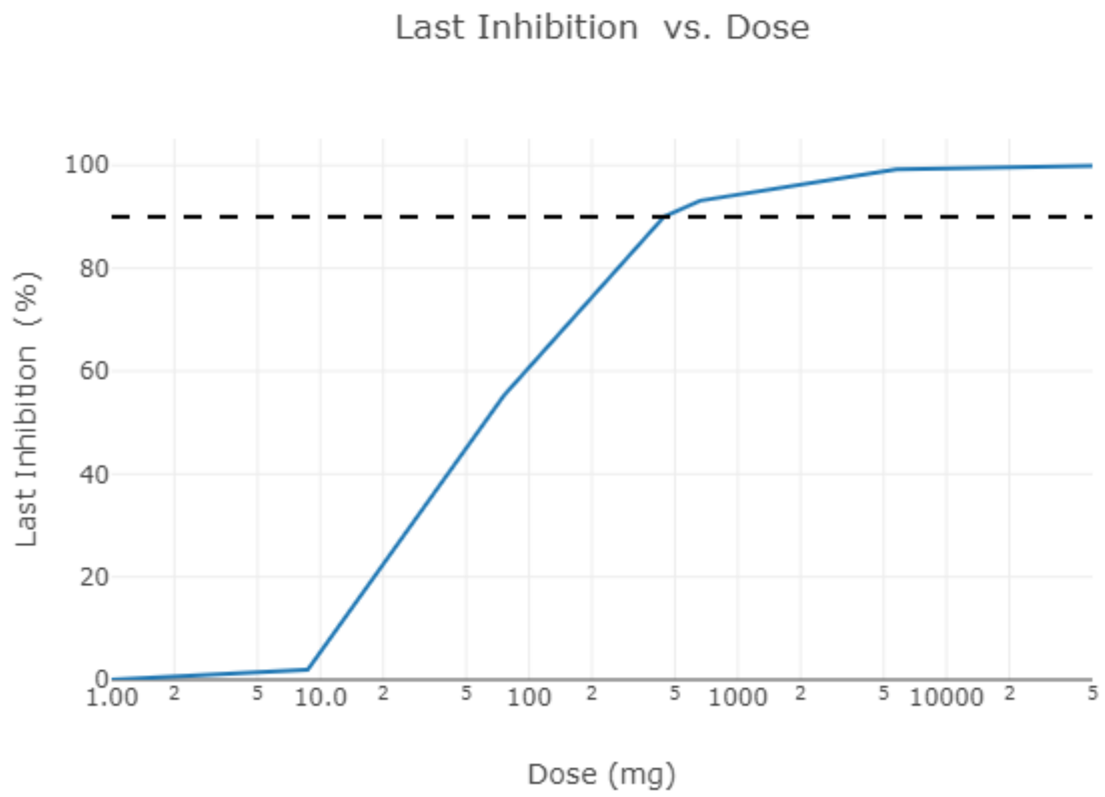

Inhibition vs. Time

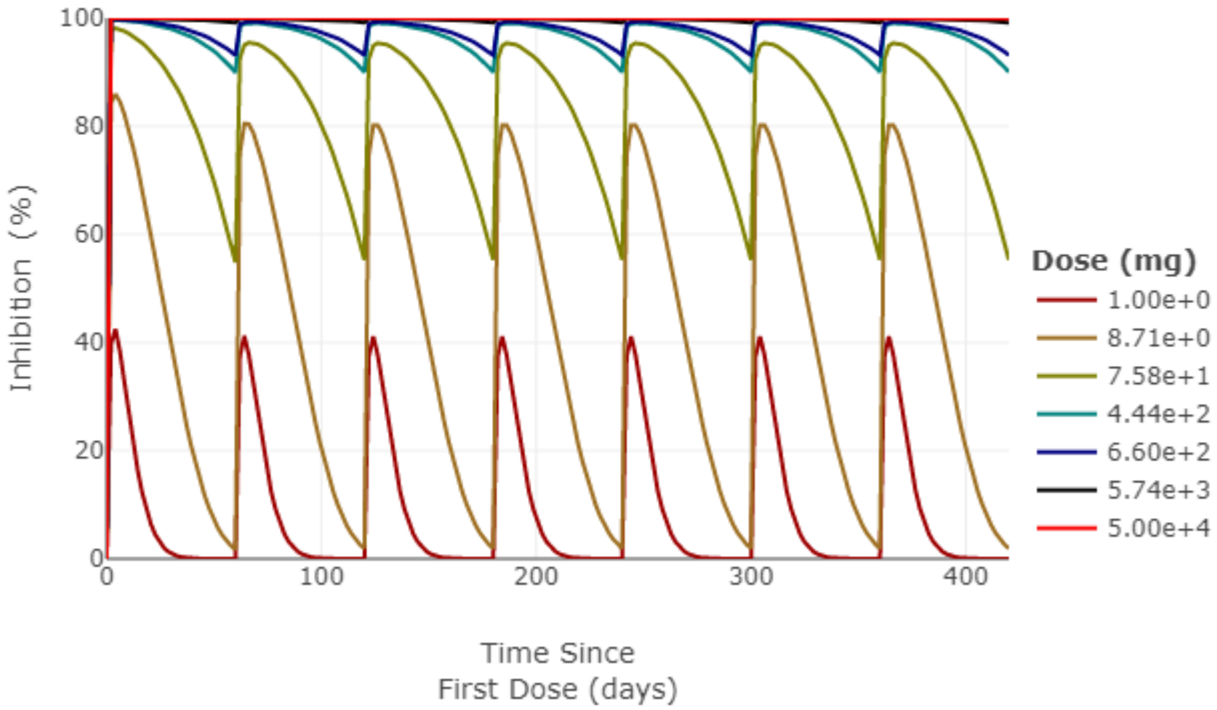


Plasma Drug in Central Compartment vs. Time

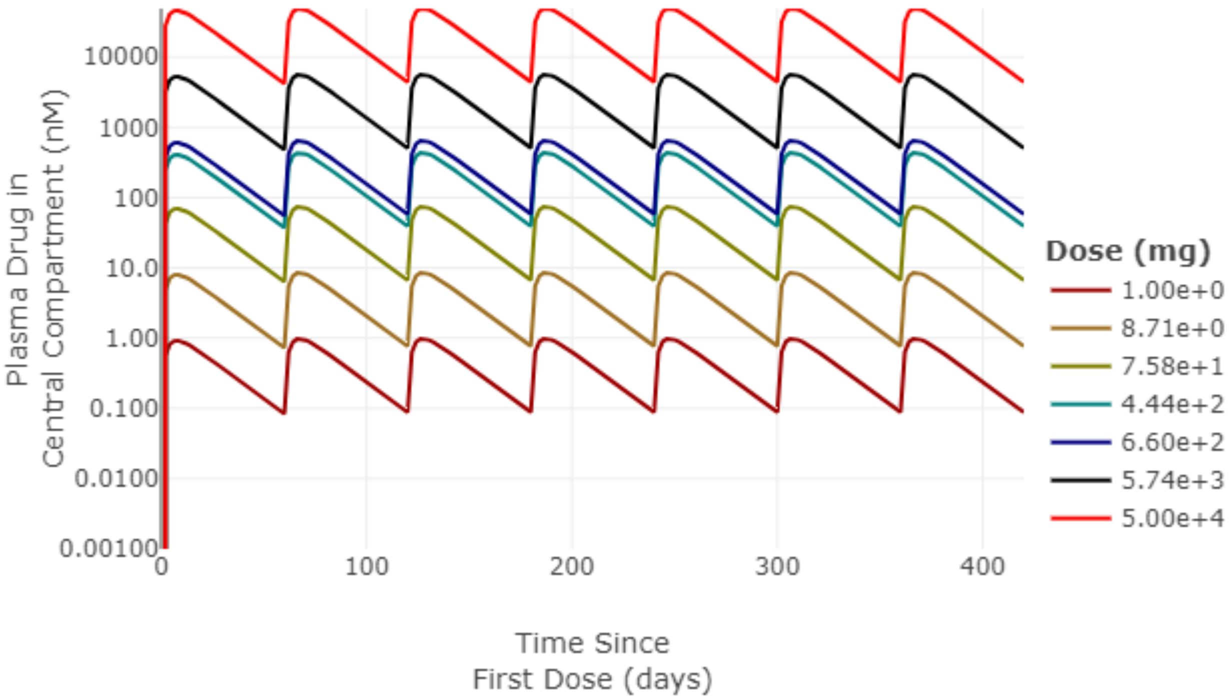


 © Applied BioMath LLC 2017-2021 

# 2.8) Scenario: ligand_halflife_high

One dimensional scan over parameter, Dose, between 1 and 50000 using log spacing.

## 2.8.1) Model Parameters

Table 2.8.1

| Symbol | Parameter ID | Value | Unit |
|---|---|---|---|
| $\tau$ | interval | 60 | - |
| $D$ | dose | 100 | mg |
| $K_{D,\mathrm{L}}$ | mab_kd_1 | 0.0042 | nM |
| $N_{\mathrm{doses}}$ | dose_count | 7 | - |
| $MW$ | mw_1 | 149000 | Da |
| $t_{1/2}$ | el_half_1 | 14 | days |
| $t_{1/2,\mathrm{a}}$ | abs_half | 2.5 | days |
| $BW$ | BW | 70 | kg |
| $V$ | volume_central | 5 | L |
| $\mathrm{Valency}$ | drug_valency_1 | 1 | - |
| $t_{1/2,\mathrm{L}}$ | lig_half_1 | 90 | min |
| $t_{1/2,\mathrm{R}}$ | rec_half_1 | 540 | min |
| $t_{1/2,\mathrm{sR}}$ | shed_half_1 | 0.5 | hr |
| $K_{D,\mathrm{L:R}}$ | lig_rec_kd_1 | 0.019 | nM |
| $C_{ss,\mathrm{L}}$ | lig_css_1_central | 0.0000575 | nM |
| $C_{ss,\mathrm{R}}$ | rec_css_1_central | 0.23 | nM |
| $C_{ss,\mathrm{sR}}$ | shed_css_1_central | 0 | nM |
| $\mathrm{Density}_{\mathrm{cells}}$ | cell_density_mL_central | 1000000 | #/mL |

| Parameter ID | Value |
|---|---|
| Scan Parameter 1 | Dose |
| Lower Limit 1 | 1 |
| Upper Limit 1 | 50000 |
| N1 | 6 |
| Scale 1 | log |

 © Applied BioMath LLC 2017-2021 

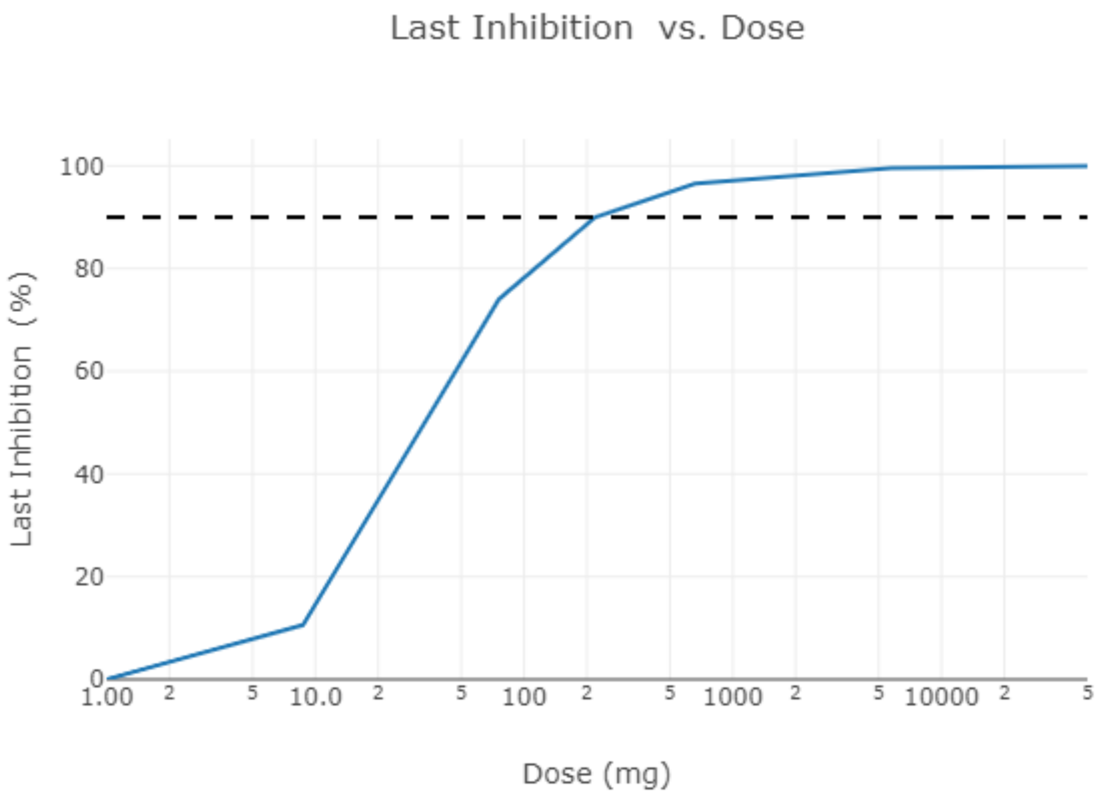
Last Inhibition vs. Dose
Last Inhibition (%)
100
80
60
40
20
0
1.00
2
5
10.0
100
1000
10000
Dose (mg)

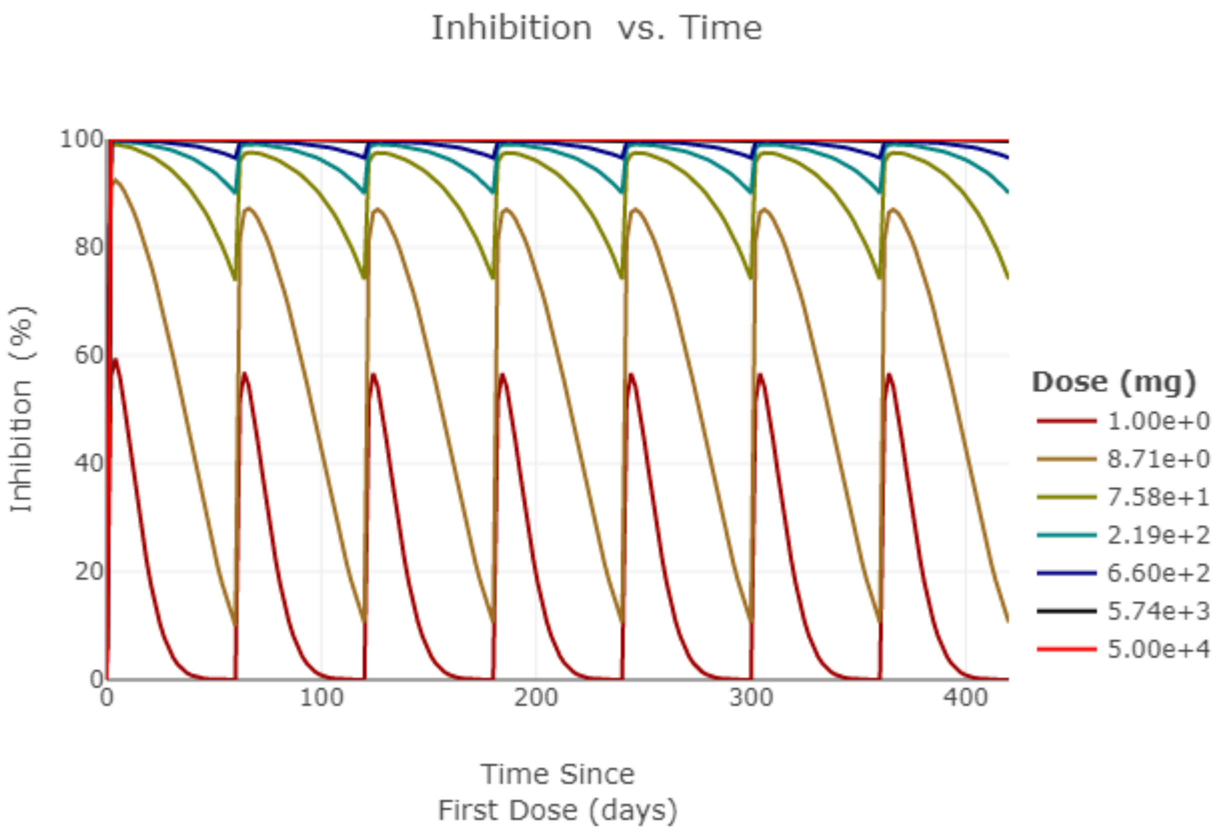
Inhibition vs. Time
Inhibition (%)
100
80
60
40
20
0
100
200
300
400
Time Since
First Dose (days)
Dose (mg)
1.00e+0
8.71e+0
7.58e+1
2.19e+2
6.60e+2
5.74e+3
5.00e+4

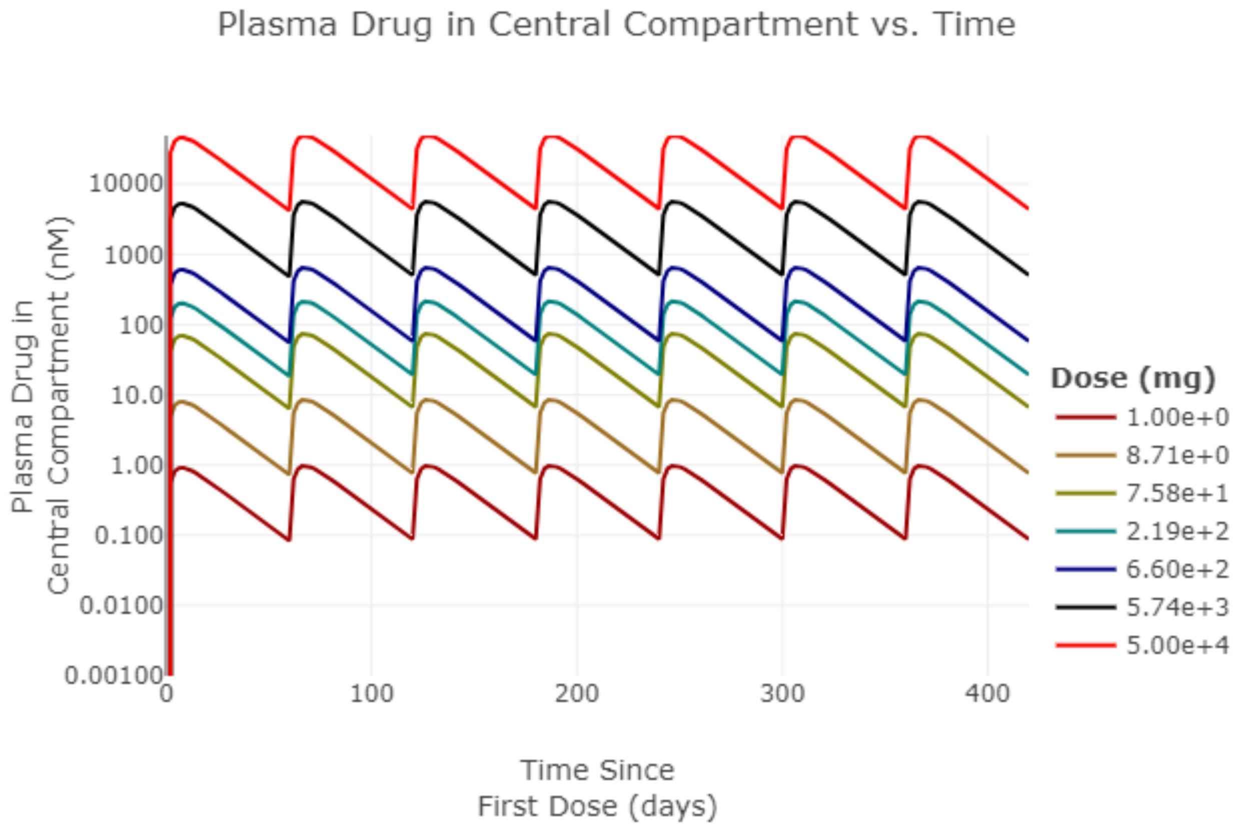


## 2.9) Scenario: ligand_halflife_low

One dimensional scan over parameter, Dose, between 1 and 50000 using log spacing.

### 2.9.1) Model Parameters

Table 2.9.1

| Symbol | Parameter ID | Value | Unit |
| --- | --- | --- | --- |
| $\tau$ | interval | 60 | - |
| $D$ | dose | 100 | mg |
| $K_{D,\mathrm{L}}$ | mab_kd_1 | 0.0042 | nM |
| $N_{\mathrm{doses}}$ | dose_count | 7 | - |
| $MW$ | mw_1 | 149000 | Da |
| $t_{1/2}$ | el_half_1 | 14 | days |
| $t_{1/2,\mathrm{a}}$ | abs_half | 2.5 | days |
| $BW$ | BW | 70 | kg |
| $V$ | volume_central | 5 | L |
| Valency | drug_valency_1 | 1 | - |
| $t_{1/2,\mathrm{L}}$ | lig_half_1 | 10 | min |
| $t_{1/2,\mathrm{R}}$ | rec_half_1 | 540 | min |

 © Applied BioMath LLC 2017-2021 

| | | | |
|---|---|---|---|
| $t_{1/2,\mathrm{sR}}$ | shed_half_1 | 0.5 | hr |
| $K_{D,\mathrm{L:R}}$ | lig_rec_kd_1 | 0.019 | nM |
| $C_{SS,\mathrm{L}}$ | lig_css_1_central | 0.0000575 | nM |
| $C_{SS,\mathrm{R}}$ | rec_css_1_central | 0.23 | nM |
| $C_{SS,\mathrm{sR}}$ | shed_css_1_central | 0 | nM |
| $\mathrm{Density}_{\mathrm{cells}}$ | cell_density_mL_central | 1000000 | #/mL |

| Parameter ID | Value |
|---|---|
| Scan Parameter 1 | Dose |
| Lower Limit 1 | 1 |
| Upper Limit 1 | 50000 |
| N1 | 6 |
| Scale 1 | log |

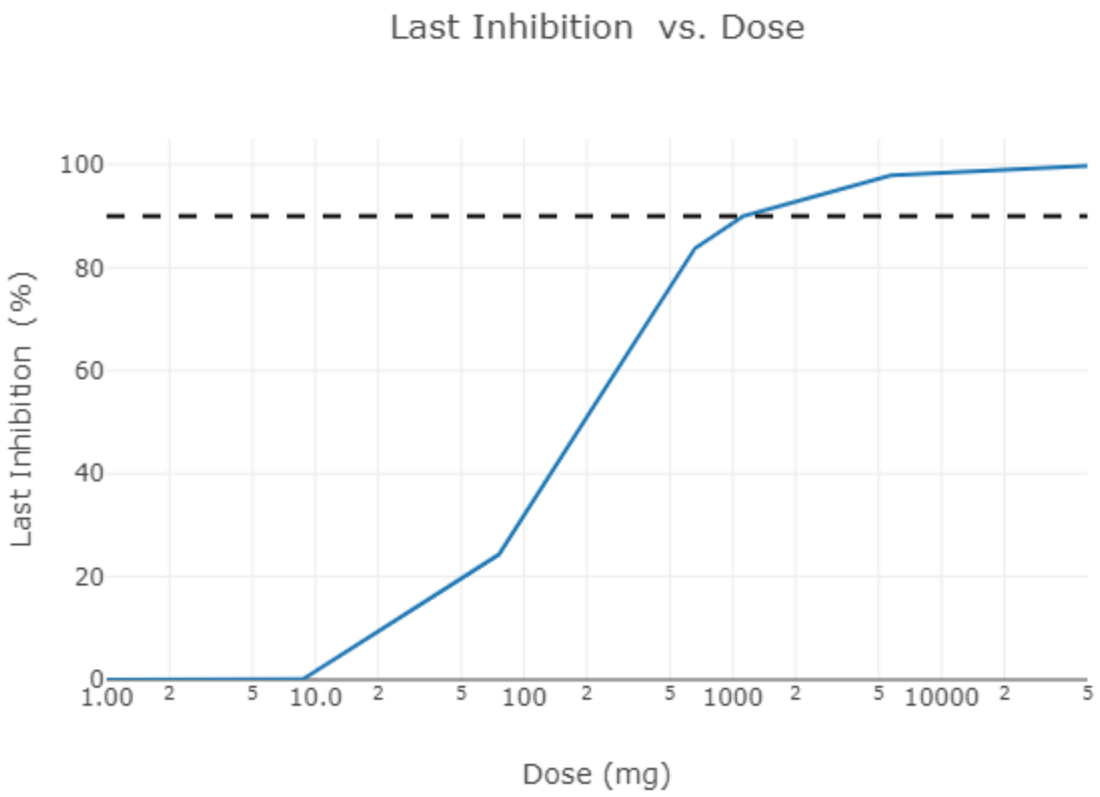

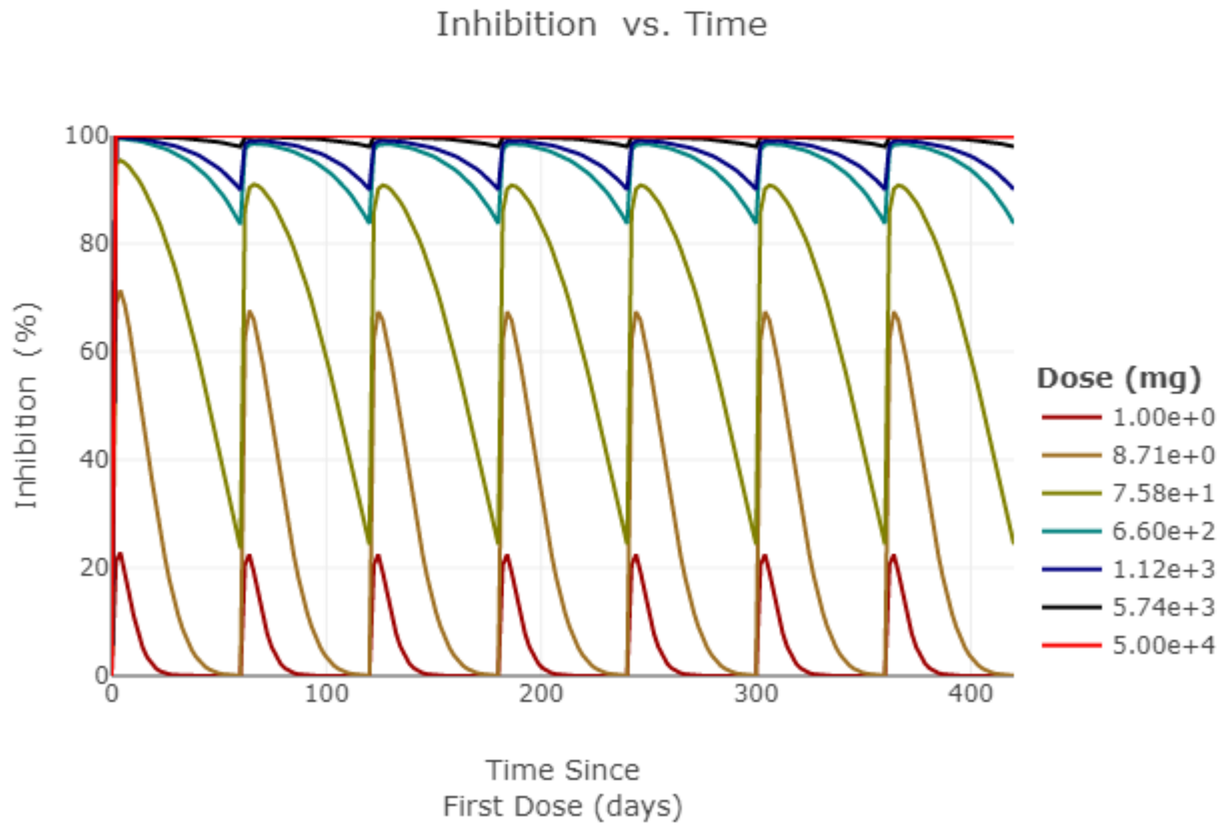


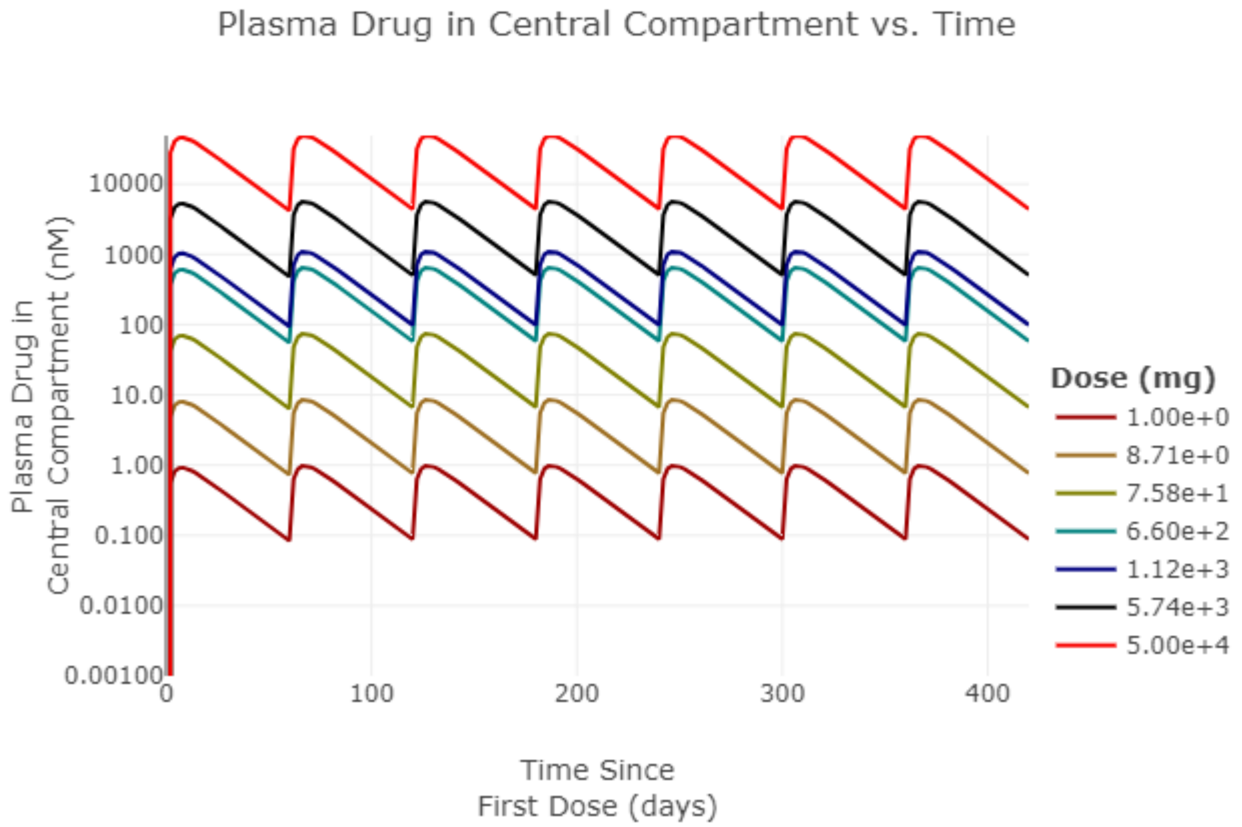


 © Applied BioMath LLC 2017-2021 

## 2.10) Scenario: ligand:rec affinity high

One dimensional scan over parameter, Dose, between 1 and 50000 using log spacing.

### 2.10.1) Model Parameters

Table 2.10.1

| Symbol | Parameter ID | Value | Unit |
|---|---|---|---|
| $\tau$ | interval | 60 | - |
| $D$ | dose | 100 | mg |
| $K_{D,\mathrm{L}}$ | mab_kd_1 | 0.0042 | nM |
| $N_{\mathrm{doses}}$ | dose_count | 7 | - |
| $MW$ | mw_1 | 149000 | Da |
| $t_{1/2}$ | el_half_1 | 14 | days |
| $t_{1/2,\mathrm{a}}$ | abs_half | 2.5 | days |
| $BW$ | BW | 70 | kg |
| $V$ | volume_central | 5 | L |
| $\mathrm{Valency}$ | drug_valency_1 | 1 | - |
| $t_{1/2,\mathrm{L}}$ | lig_half_1 | 30 | min |
| $t_{1/2,\mathrm{R}}$ | rec_half_1 | 540 | min |
| $t_{1/2,\mathrm{sR}}$ | shed_half_1 | 0.5 | hr |
| $K_{D,\mathrm{L:R}}$ | lig_rec_kd_1 | 0.006 | nM |
| $C_{ss,\mathrm{L}}$ | lig_css_1_central | 0.0000575 | nM |
| $C_{ss,\mathrm{R}}$ | rec_css_1_central | 0.23 | nM |
| $C_{ss,\mathrm{sR}}$ | shed_css_1_central | 0 | nM |
| $\mathrm{Density}_{\mathrm{cells}}$ | cell_density_mL_central | 1000000 | #/mL |

| Parameter ID | Value |
|---|---|
| Scan Parameter 1 | Dose |
| Lower Limit 1 | 1 |
| Upper Limit 1 | 50000 |
| N1 | 6 |
| Scale 1 | log |

 © Applied BioMath LLC 2017-2021 

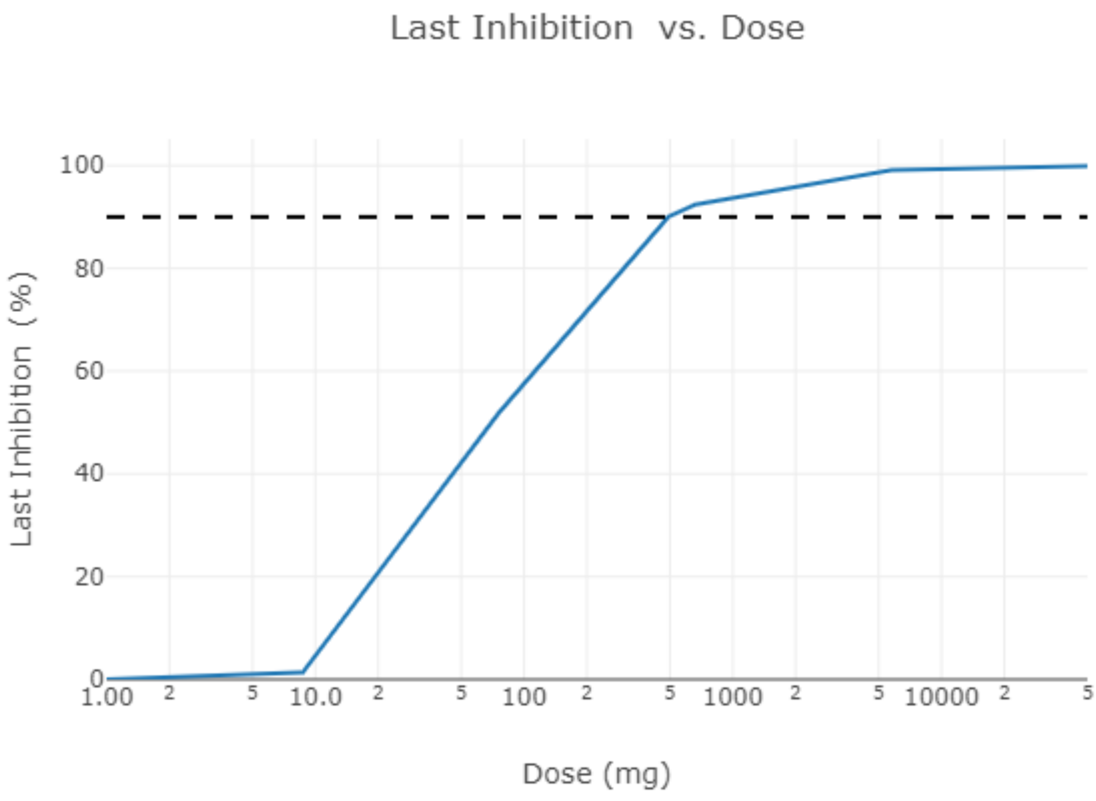

Last Inhibition vs. Dose
100
80
60
40
20
0
Last Inhibition (%)
1.00
2
5
10.0
2
5
100
2
5
1000
2
5
10000
2
5
Dose (mg)


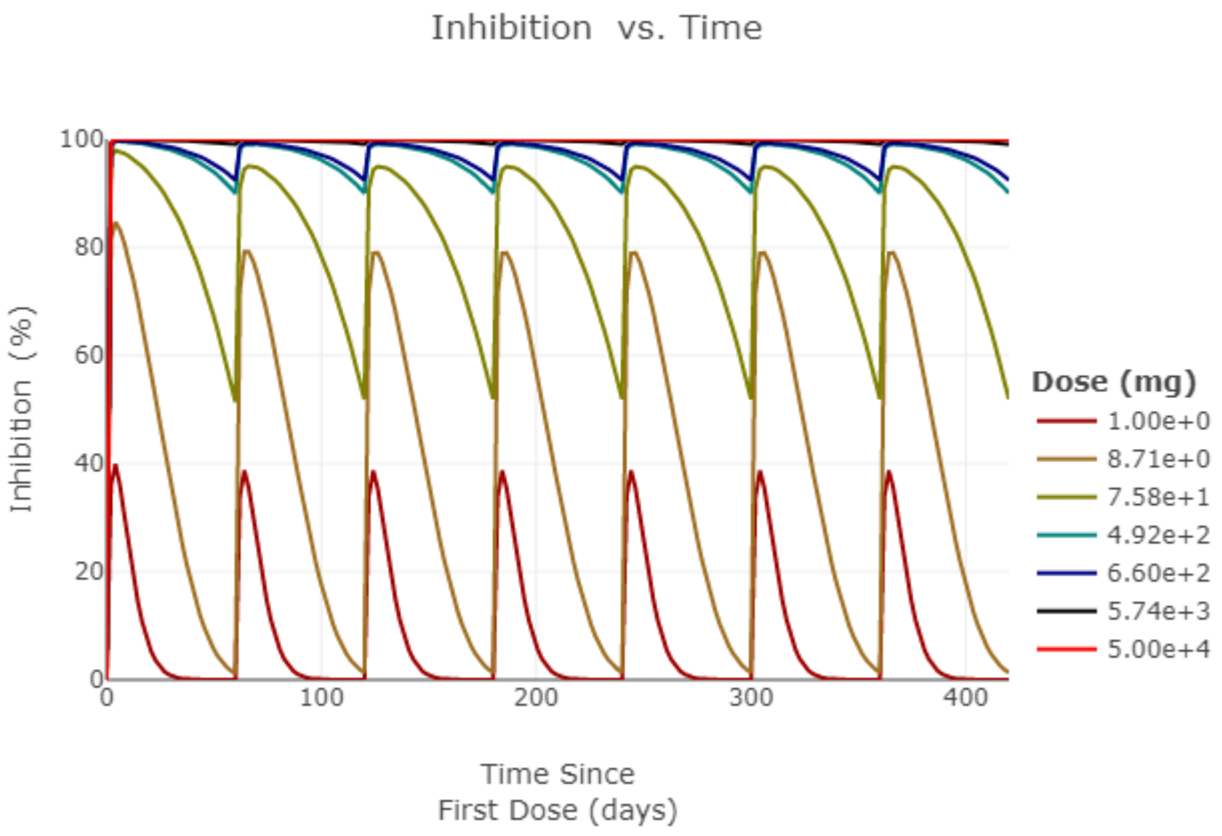

Inhibition vs. Time
100
80
60
40
20
0
Inhibition (%)
0
100
200
300
400
Time Since
First Dose (days)
Dose (mg)
1.00e+0
8.71e+0
7.58e+1
4.92e+2
6.60e+2
5.74e+3
5.00e+4

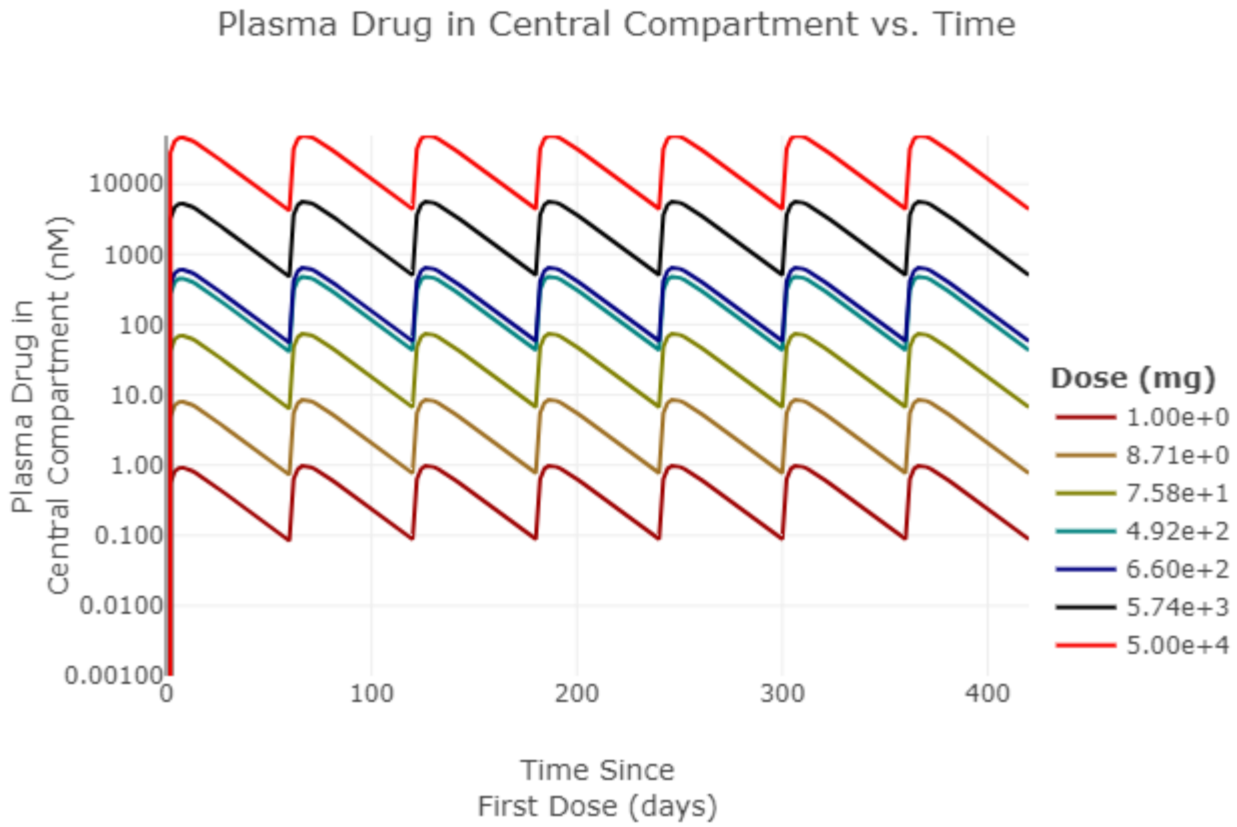


## 2.11) Scenario: ligand:rec affinity low

One dimensional scan over parameter, Dose, between 1 and 50000 using log spacing.

### 2.11.1) Model Parameters

Table 2.11.1

| Symbol | Parameter ID | Value | Unit |
|---|---|---|---|
| $\tau$ | interval | 60 | - |
| $D$ | dose | 100 | mg |
| $K_{D,\mathrm{L}}$ | mab_kd_1 | 0.0042 | nM |
| $N_{\mathrm{doses}}$ | dose_count | 7 | - |
| $MW$ | mw_1 | 149000 | Da |
| $t_{1/2}$ | el_half_1 | 14 | days |
| $t_{1/2,\mathrm{a}}$ | abs_half | 2.5 | days |
| $BW$ | BW | 70 | kg |
| $V$ | volume_central | 5 | L |
| Valency | drug_valency_1 | 1 | - |
| $t_{1/2,\mathrm{L}}$ | lig_half_1 | 30 | min |
| $t_{1/2,\mathrm{R}}$ | rec_half_1 | 540 | min |

 © Applied BioMath LLC 2017-2021 

| | | | |
|---|---|---|---|
| $t_{1/2,\mathrm{sR}}$ | shed_half_1 | 0.5 | hr |
| $K_{D,\mathrm{L:R}}$ | lig_rec_kd_1 | 0.057 | nM |
| $C_{SS,\mathrm{L}}$ | lig_css_1_central | 0.0000575 | nM |
| $C_{SS,\mathrm{R}}$ | rec_css_1_central | 0.23 | nM |
| $C_{SS,\mathrm{sR}}$ | shed_css_1_central | 0 | nM |
| $\mathrm{Density}_{\mathrm{cells}}$ | cell_density_mL_central | 1000000 | #/mL |

| Parameter ID | Value |
|---|---|
| Scan Parameter 1 | Dose |
| Lower Limit 1 | 1 |
| Upper Limit 1 | 50000 |
| N1 | 6 |
| Scale 1 | log |

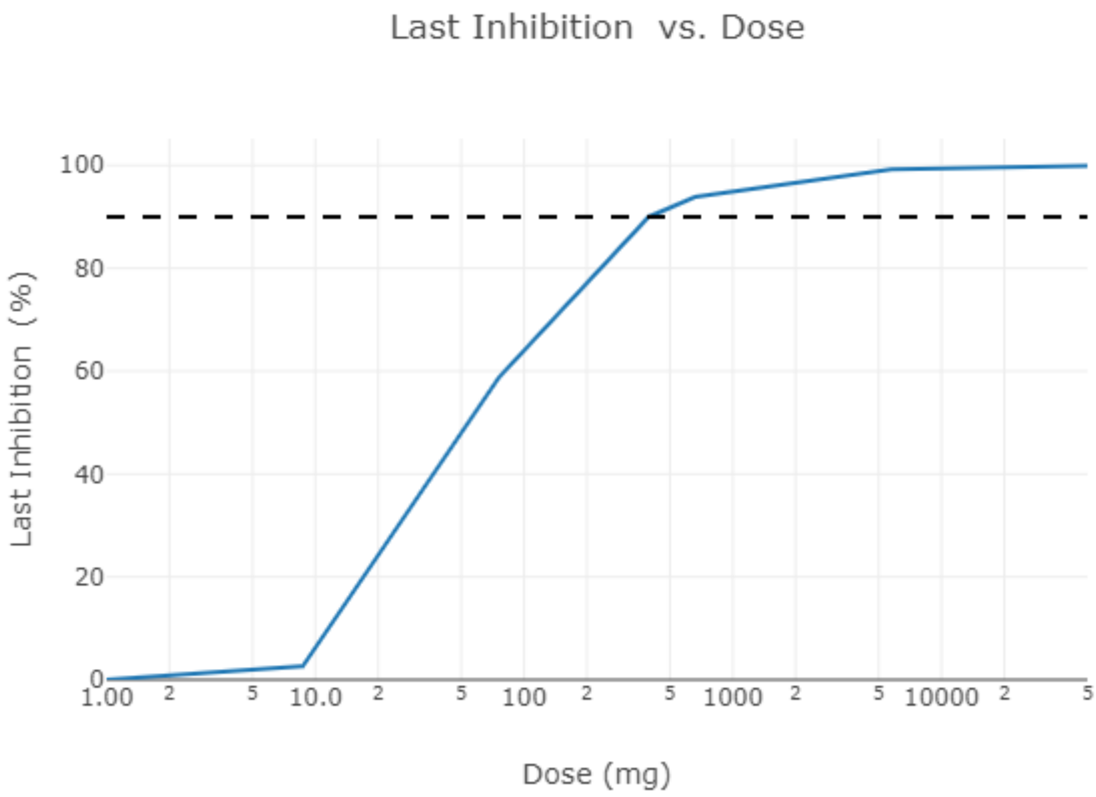


 © Applied BioMath LLC 2017-2021 

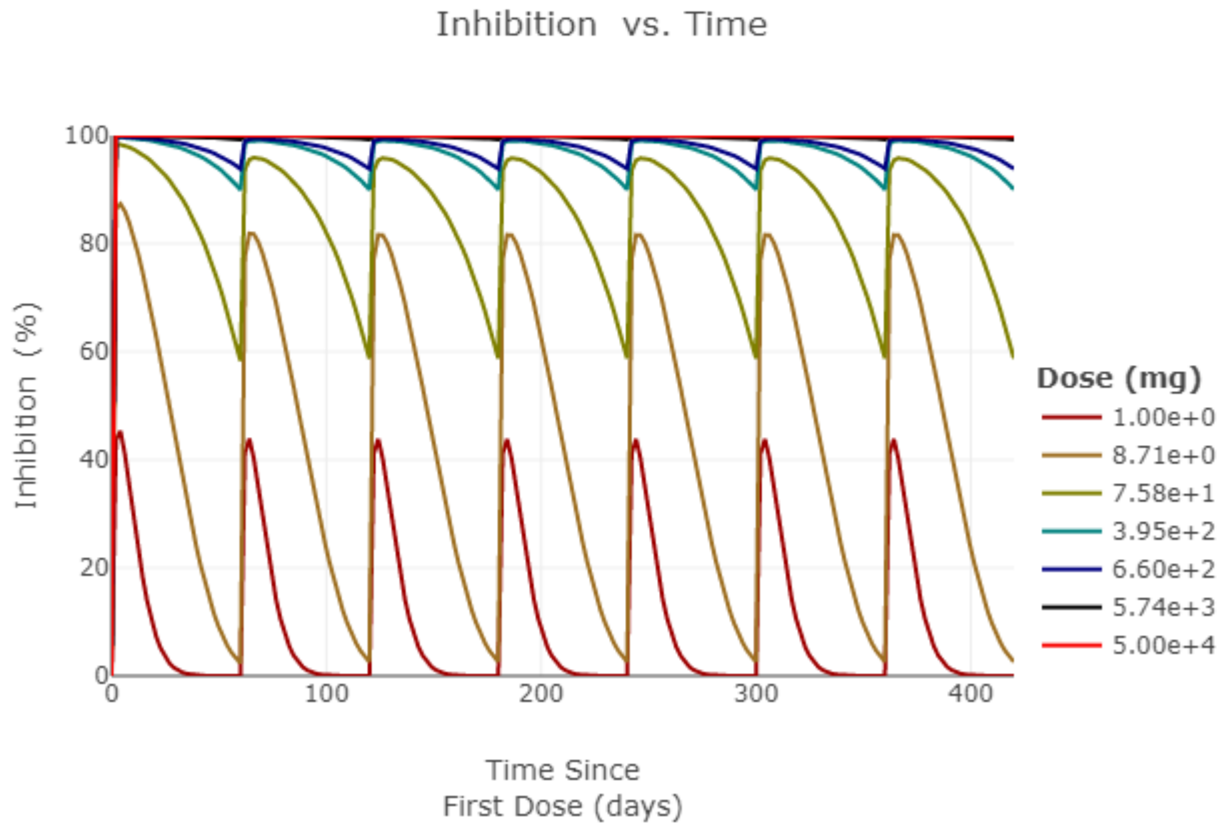
Inhibition vs. Time
Inhibition (%)
100
80
60
40
20
0
0
100
200
300
400
Time Since
First Dose (days)
Dose (mg)
1.00e+0
8.71e+0
7.58e+1
3.95e+2
6.60e+2
5.74e+3
5.00e+4

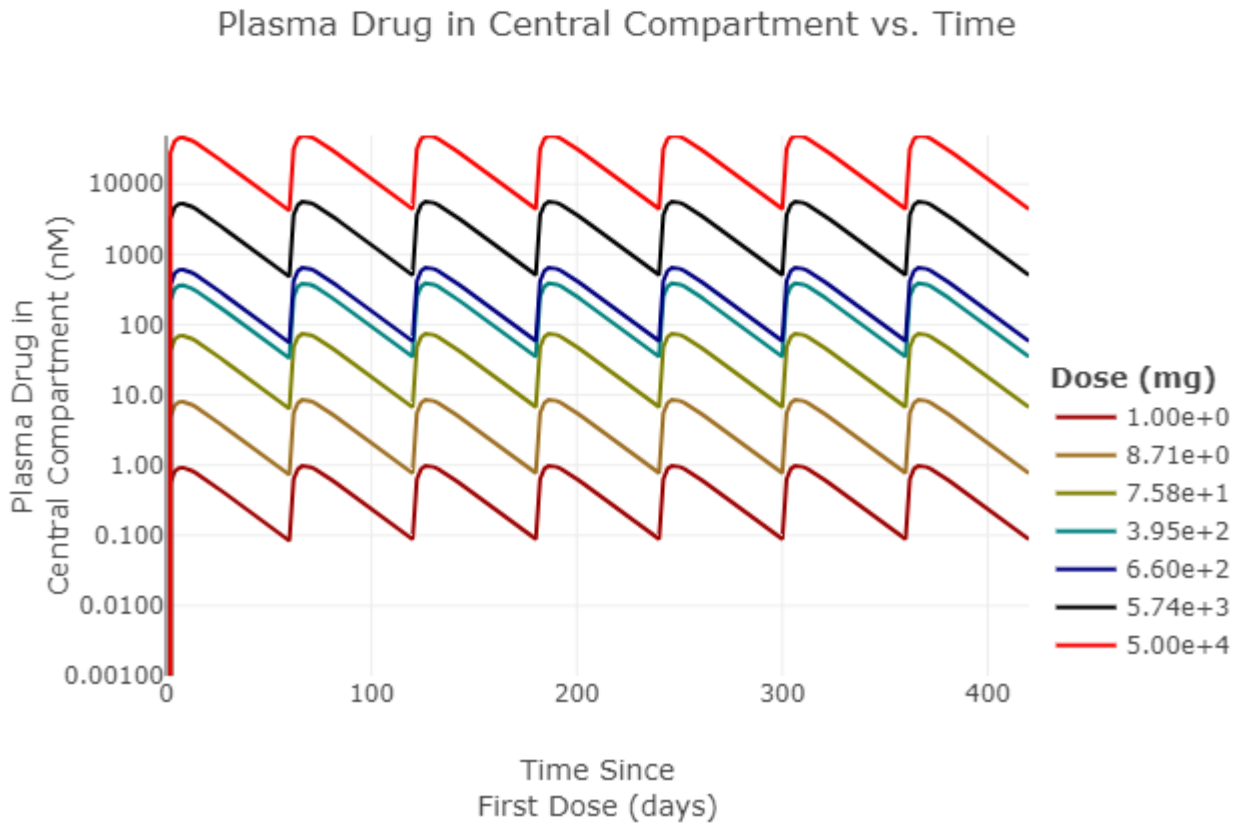
Plasma Drug in Central Compartment vs. Time
Plasma Drug in
Central Compartment (nM)
10000
1000
100
10.0
1.00
0.100
0.0100
0.00100
0
100
200
300
400
Time Since
First Dose (days)
Dose (mg)
1.00e+0
8.71e+0
7.58e+1
3.95e+2
6.60e+2
5.74e+3
5.00e+4

# 2.12) Scenario: drug_affinity_high

One dimensional scan over parameter, Dose, between 1 and 50000 using log spacing.

## 2.12.1) Model Parameters

Table 2.12.1

| Symbol | Parameter ID | Value | Unit |
|---|---|---|---|
| $\tau$ | interval | 60 | - |
| $D$ | dose | 100 | mg |
| $K_{D,\mathrm{L}}$ | mab_kd_1 | 0.0014 | nM |
| $N_{\mathrm{doses}}$ | dose_count | 7 | - |
| $MW$ | mw_1 | 149000 | Da |
| $t_{1/2}$ | el_half_1 | 14 | days |
| $t_{1/2,\mathrm{a}}$ | abs_half | 2.5 | days |
| $BW$ | BW | 70 | kg |
| $V$ | volume_central | 5 | L |
| $\mathrm{Valency}$ | drug_valency_1 | 1 | - |
| $t_{1/2,\mathrm{L}}$ | lig_half_1 | 30 | min |
| $t_{1/2,\mathrm{R}}$ | rec_half_1 | 540 | min |
| $t_{1/2,\mathrm{sR}}$ | shed_half_1 | 0.5 | hr |
| $K_{D,\mathrm{L:R}}$ | lig_rec_kd_1 | 0.019 | nM |
| $C_{ss,\mathrm{L}}$ | lig_css_1_central | 0.0000575 | nM |
| $C_{ss,\mathrm{R}}$ | rec_css_1_central | 0.23 | nM |
| $C_{ss,\mathrm{sR}}$ | shed_css_1_central | 0 | nM |
| $\mathrm{Density}_{\mathrm{cells}}$ | cell_density_mL_central | 1000000 | #/mL |

| Parameter ID | Value |
|---|---|
| Scan Parameter 1 | Dose |
| Lower Limit 1 | 1 |
| Upper Limit 1 | 50000 |
| N1 | 6 |
| Scale 1 | log |

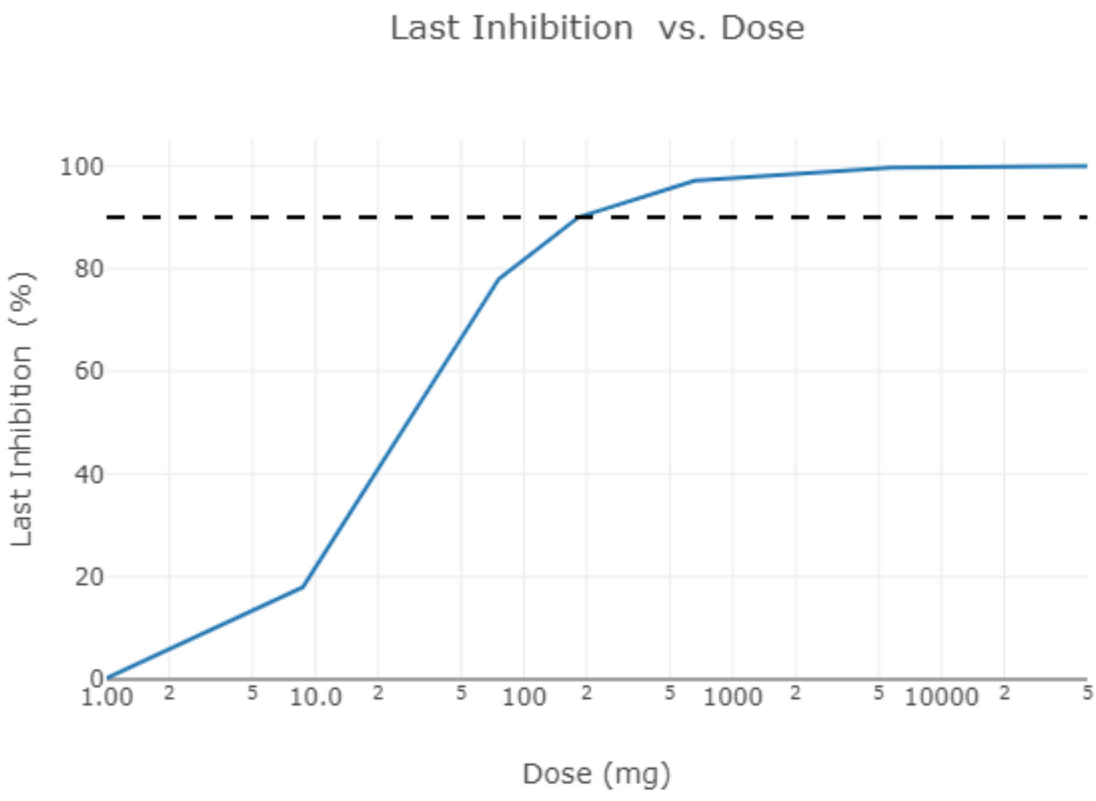
Last Inhibition vs. Dose
Last Inhibition (%)
Dose (mg)
100
80
60
40
20
0
1.00
10.0
100
1000
10000

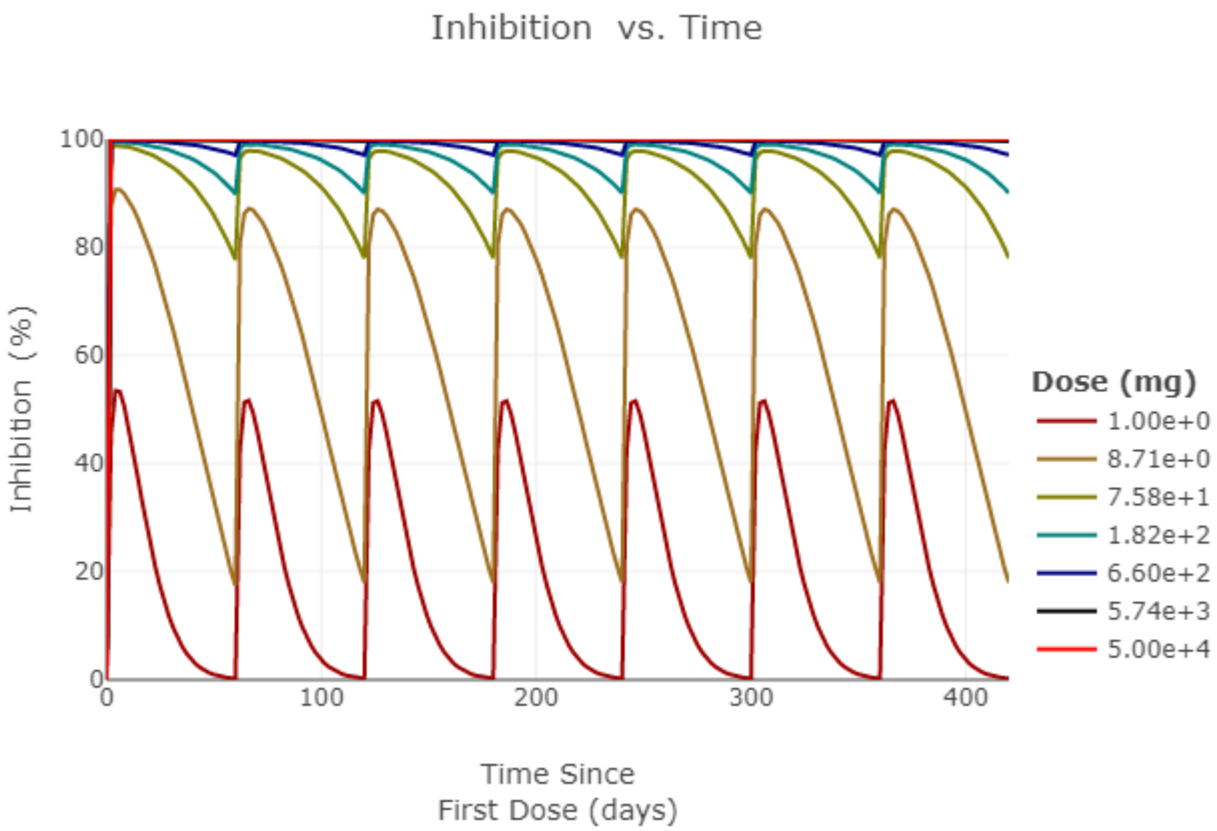
Inhibition vs. Time
Inhibition (%)
Time Since
First Dose (days)
Dose (mg)
1.00e+0
8.71e+0
7.58e+1
1.82e+2
6.60e+2
5.74e+3
5.00e+4

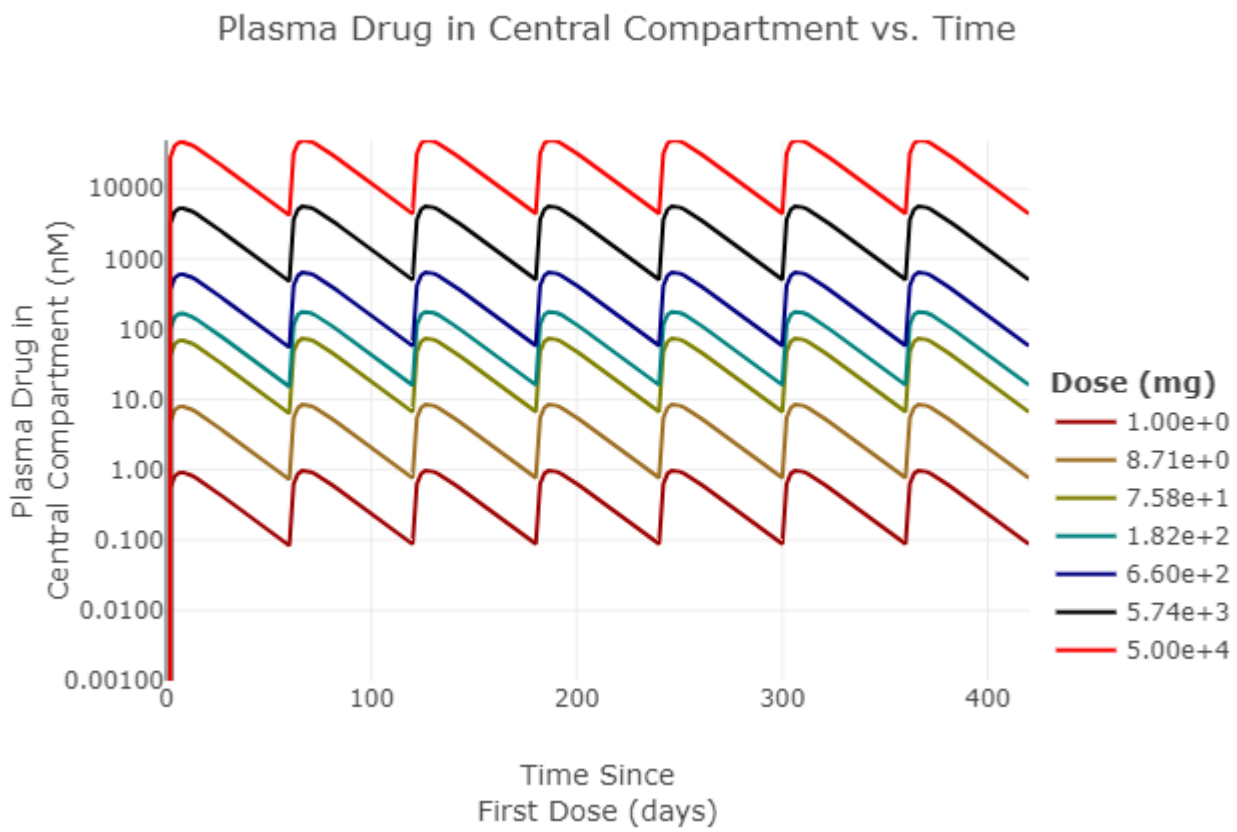


## 2.13) Scenario: drug_affinity_low

One dimensional scan over parameter, Dose, between 1 and 50000 using log spacing.

### 2.13.1) Model Parameters

Table 2.13.1

| Symbol | Parameter ID | Value | Unit |
|---|---|---|---|
| $\tau$ | interval | 60 | - |
| $D$ | dose | 100 | mg |
| $K_{D,\mathrm{L}}$ | mab_kd_1 | 0.0126 | nM |
| $N_{\mathrm{doses}}$ | dose_count | 7 | - |
| $MW$ | mw_1 | 149000 | Da |
| $t_{1/2}$ | el_half_1 | 14 | days |
| $t_{1/2,\mathrm{a}}$ | abs_half | 2.5 | days |
| $BW$ | BW | 70 | kg |
| $V$ | volume_central | 5 | L |
| Valency | drug_valency_1 | 1 | - |
| $t_{1/2,\mathrm{L}}$ | lig_half_1 | 30 | min |
| $t_{1/2,\mathrm{R}}$ | rec_half_1 | 540 | min |

 © Applied BioMath LLC 2017-2021 

| | | | |
|---|---|---|---|
| $t_{1/2,\mathrm{sR}}$ | shed_half_1 | 0.5 | hr |
| $K_{D,\mathrm{L:R}}$ | lig_rec_kd_1 | 0.019 | nM |
| $C_{SS,\mathrm{L}}$ | lig_css_1_central | 0.0000575 | nM |
| $C_{SS,\mathrm{R}}$ | rec_css_1_central | 0.23 | nM |
| $C_{SS,\mathrm{sR}}$ | shed_css_1_central | 0 | nM |
| $\mathrm{Density}_{\mathrm{cells}}$ | cell_density_mL_central | 1000000 | #/mL |

| Parameter ID | Value |
|---|---|
| Scan Parameter 1 | Dose |
| Lower Limit 1 | 1 |
| Upper Limit 1 | 50000 |
| N1 | 6 |
| Scale 1 | log |

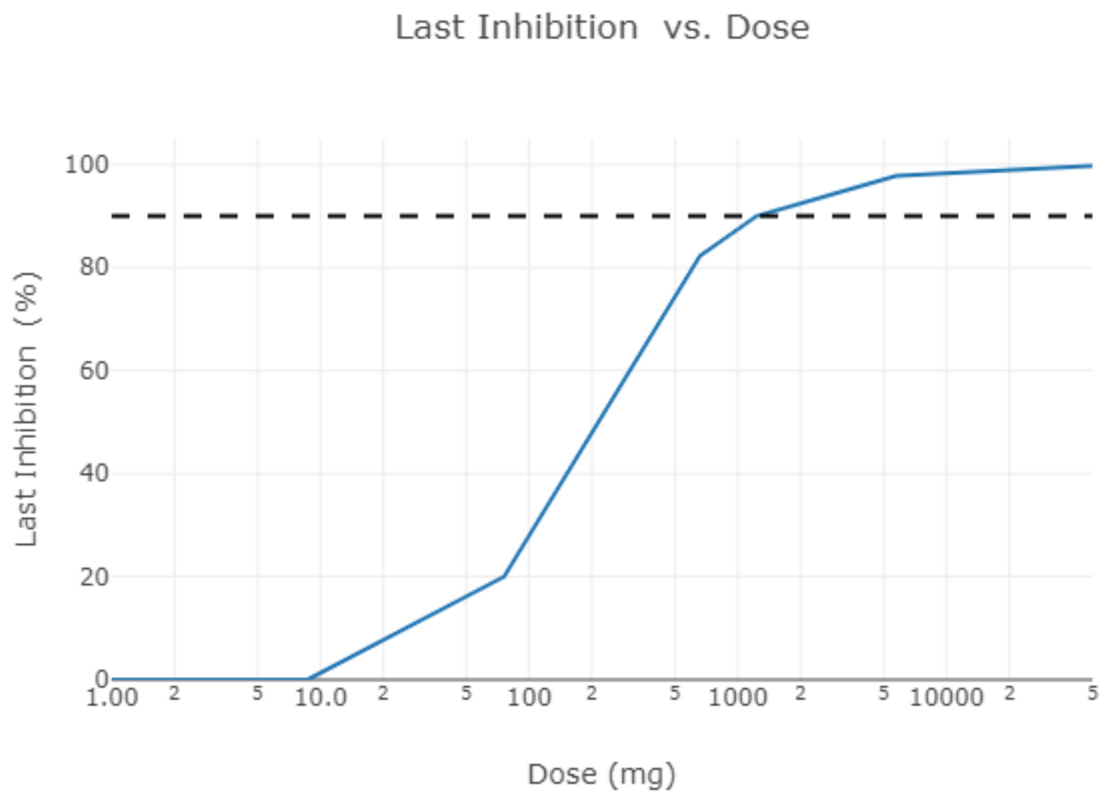


 © Applied BioMath LLC 2017-2021 

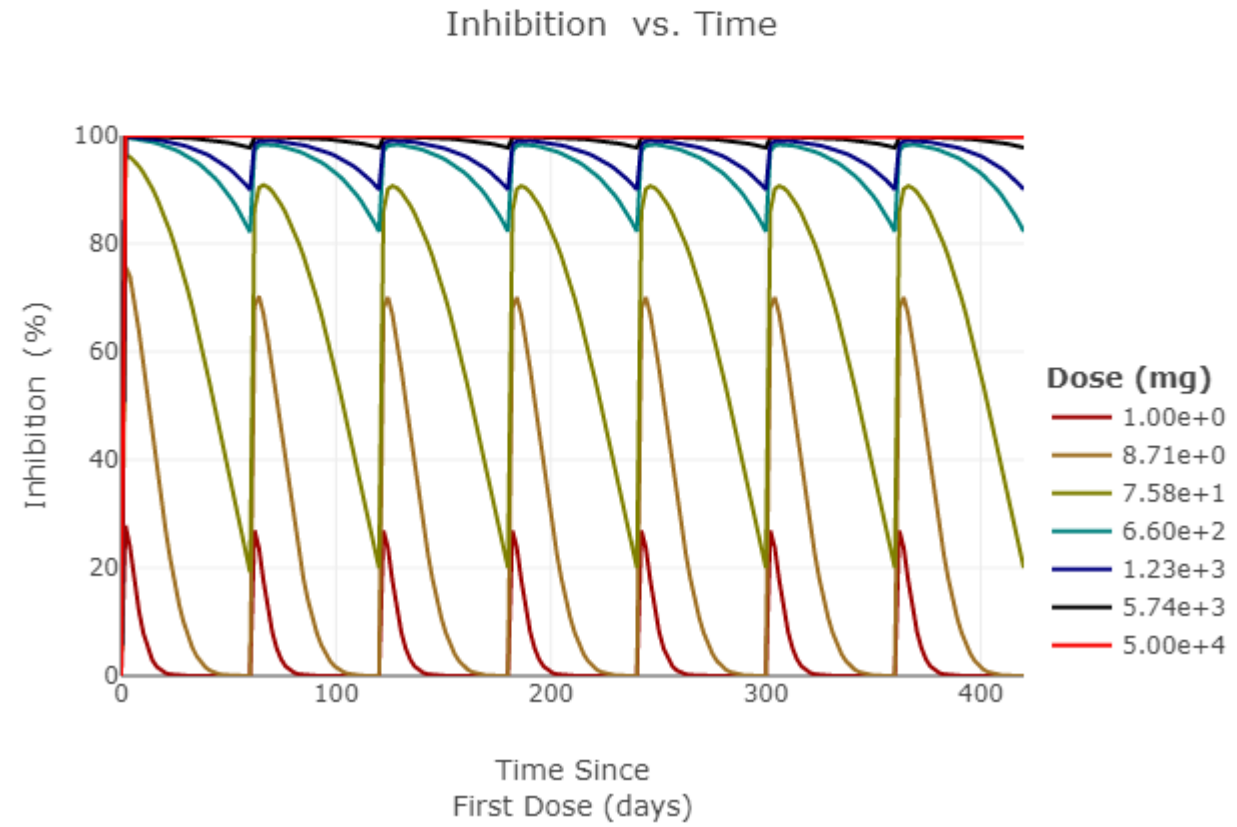


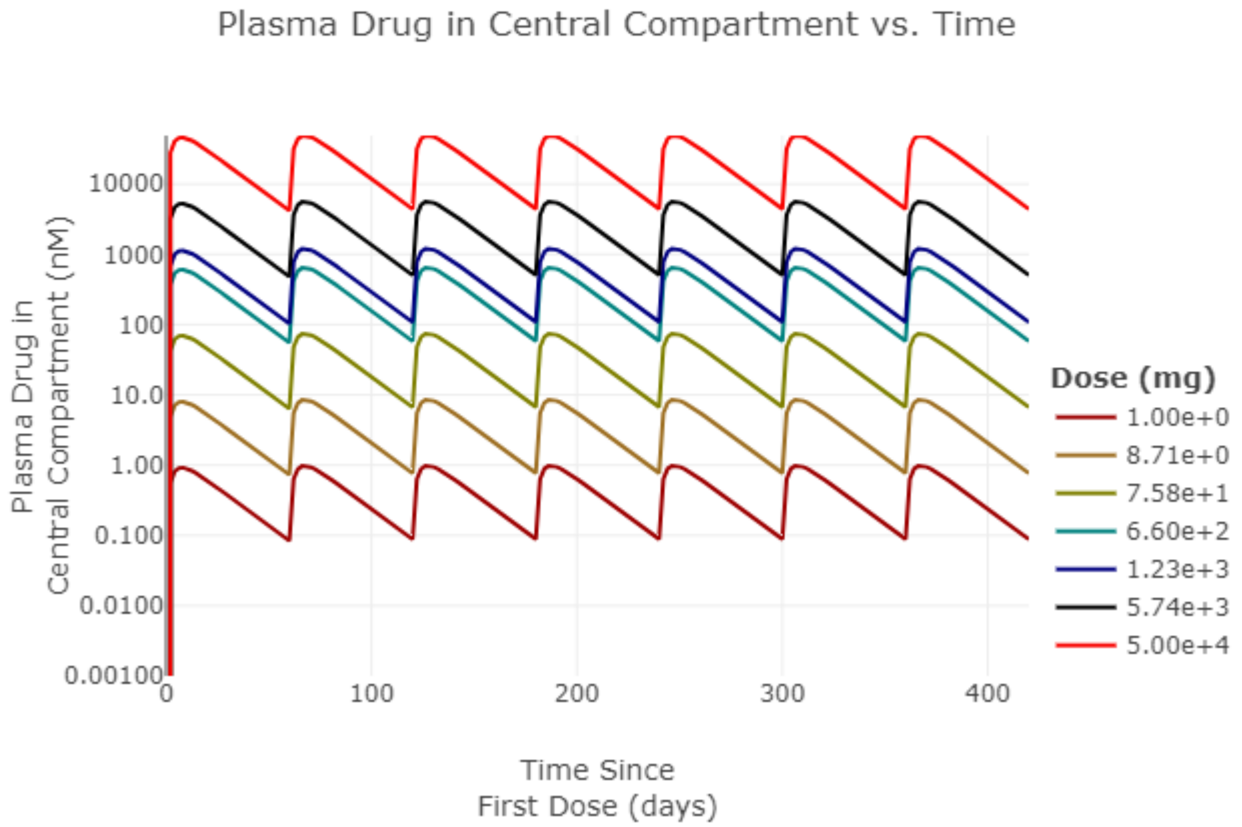


 © Applied BioMath LLC 2017-2021 

# 2.14) Scenario: receptor_conc_high

One dimensional scan over parameter, Dose, between 1 and 50000 using log spacing.

## 2.14.1) Model Parameters

Table 2.14.1

| Symbol | Parameter ID | Value | Unit |
|---|---|---|---|
| $\tau$ | interval | 60 | - |
| $D$ | dose | 100 | mg |
| $K_{D,\mathrm{L}}$ | mab_kd_1 | 0.0042 | nM |
| $N_{\mathrm{doses}}$ | dose_count | 7 | - |
| $MW$ | mw_1 | 149000 | Da |
| $t_{1/2}$ | el_half_1 | 14 | days |
| $t_{1/2,\mathrm{a}}$ | abs_half | 2.5 | days |
| $BW$ | BW | 70 | kg |
| $V$ | volume_central | 5 | L |
| $\mathrm{Valency}$ | drug_valency_1 | 1 | - |
| $t_{1/2,\mathrm{L}}$ | lig_half_1 | 30 | min |
| $t_{1/2,\mathrm{R}}$ | rec_half_1 | 540 | min |
| $t_{1/2,\mathrm{sR}}$ | shed_half_1 | 0.5 | hr |
| $K_{D,\mathrm{L:R}}$ | lig_rec_kd_1 | 0.019 | nM |
| $C_{ss,\mathrm{L}}$ | lig_css_1_central | 0.0000575 | nM |
| $C_{ss,\mathrm{R}}$ | rec_css_1_central | 0.69 | nM |
| $C_{ss,\mathrm{sR}}$ | shed_css_1_central | 0 | nM |
| $\mathrm{Density}_{\mathrm{cells}}$ | cell_density_mL_central | 1000000 | #/mL |

| Parameter ID | Value |
|---|---|
| Scan Parameter 1 | Dose |
| Lower Limit 1 | 1 |
| Upper Limit 1 | 50000 |
| N1 | 6 |
| Scale 1 | log |


© Applied BioMath LLC 2017-2021

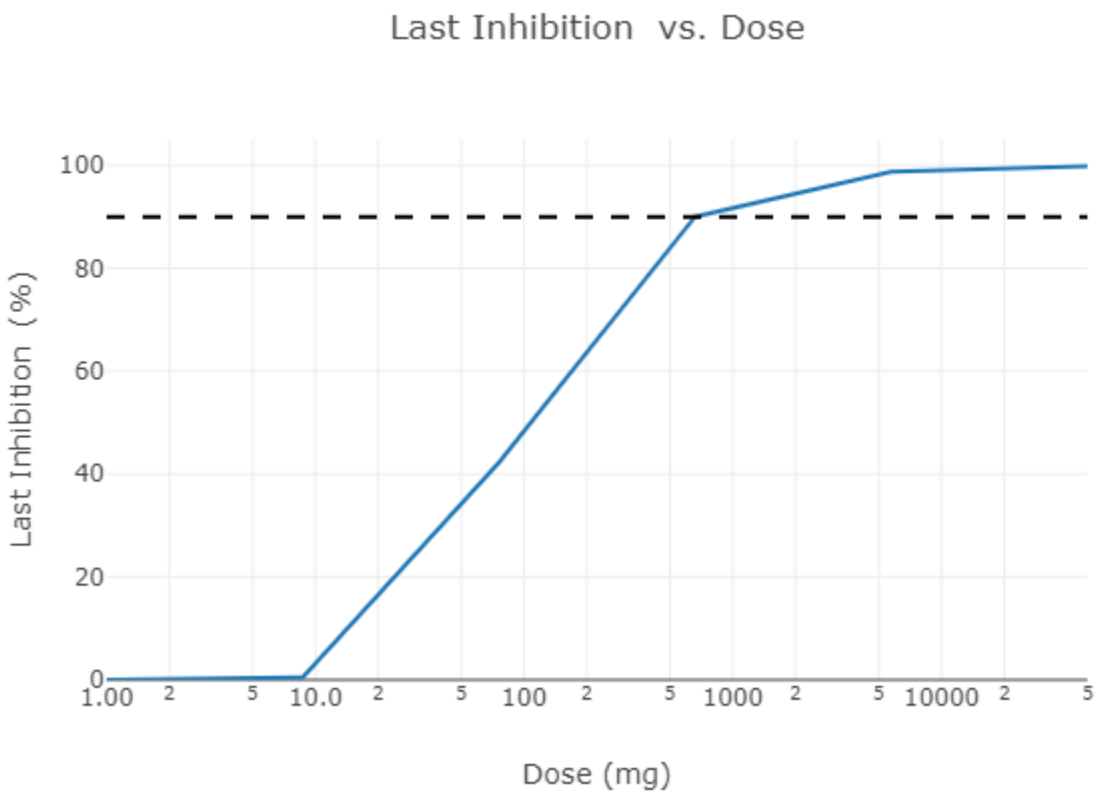
Last Inhibition vs. Dose
Last Inhibition (%)
100
80
60
40
20
0
1.00
2
5
10.0
2
5
100
2
5
1000
2
5
10000
2
5
Dose (mg)

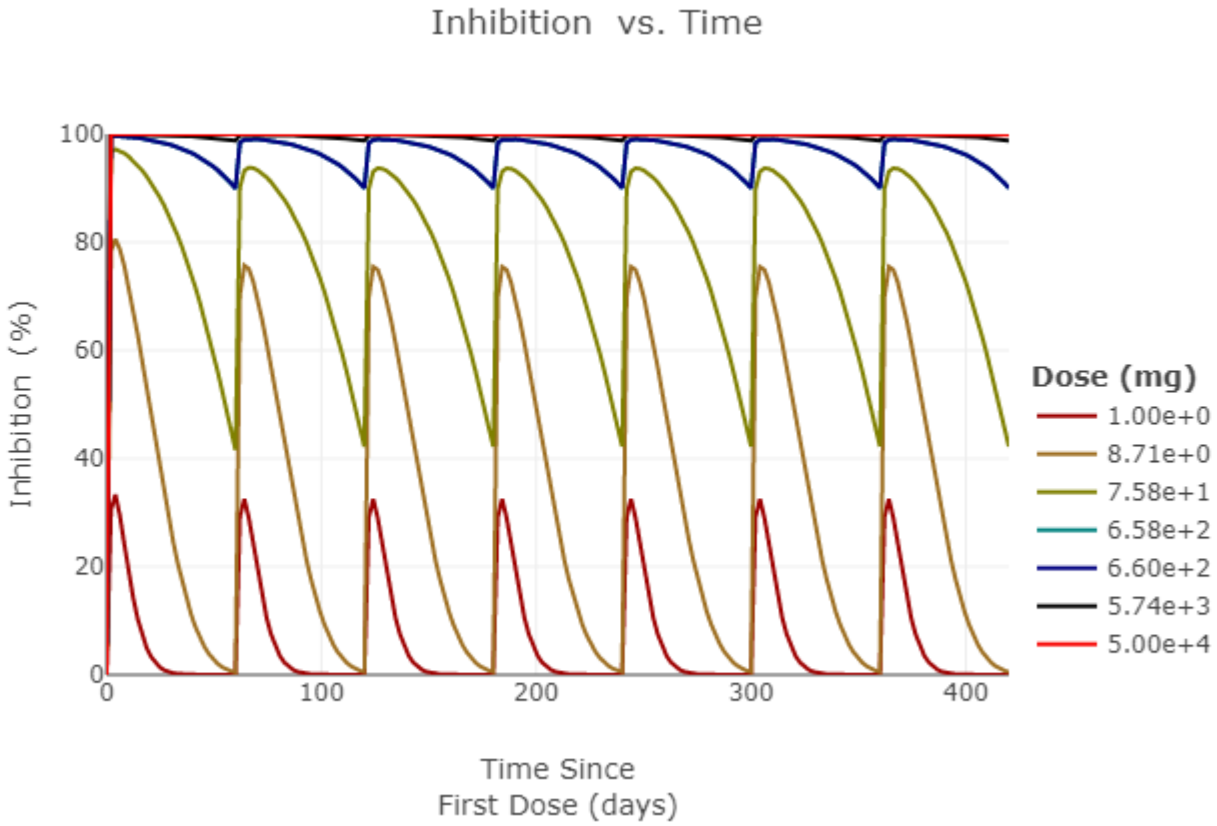
Inhibition vs. Time
Inhibition (%)
100
80
60
40
20
0
0
100
200
300
400
Time Since
First Dose (days)
Dose (mg)
1.00e+0
8.71e+0
7.58e+1
6.58e+2
6.60e+2
5.74e+3
5.00e+4

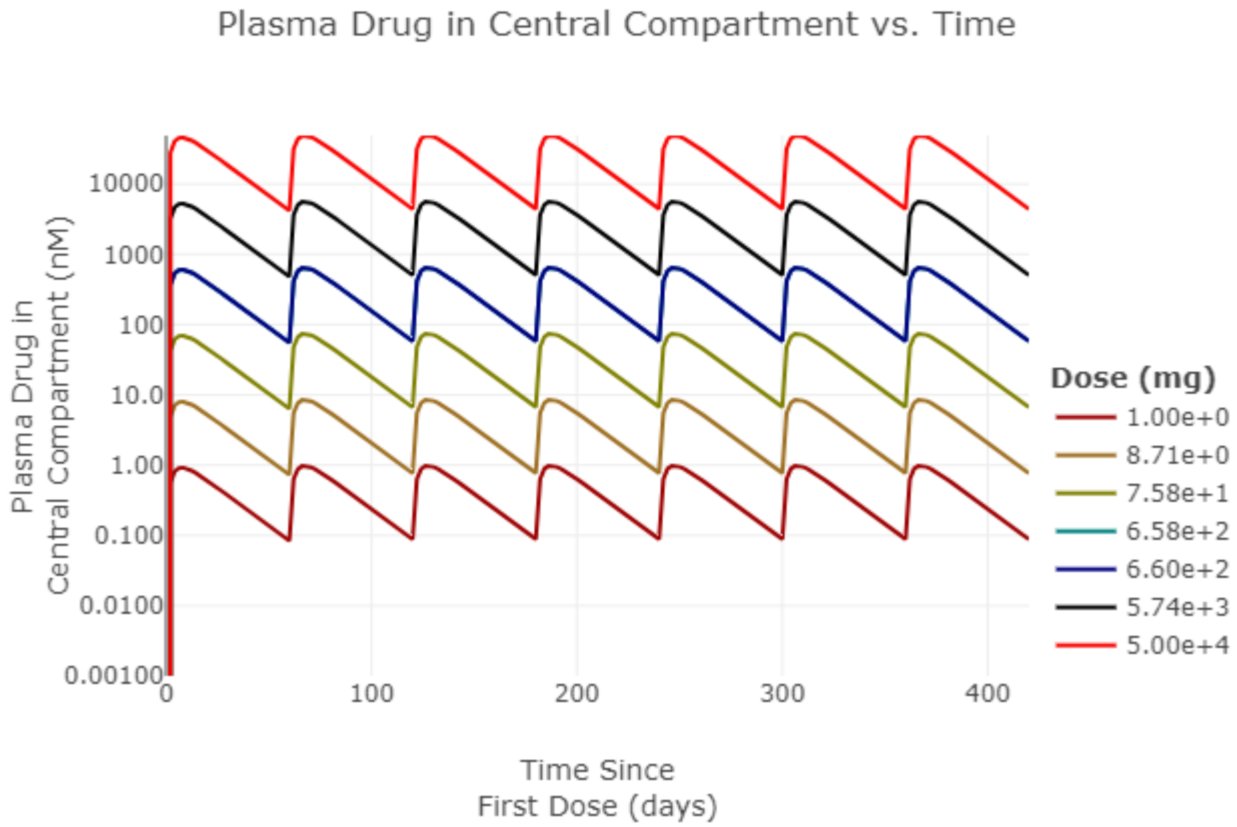


## 2.15) Scenario: receptor_conc_low

One dimensional scan over parameter, Dose, between 1 and 50000 using log spacing.

### 2.15.1) Model Parameters

Table 2.15.1

| Symbol | Parameter ID | Value | Unit |
|---|---|---|---|
| $\tau$ | interval | 60 | - |
| $D$ | dose | 100 | mg |
| $K_{D,\mathrm{L}}$ | mab_kd_1 | 0.0042 | nM |
| $N_{\mathrm{doses}}$ | dose_count | 7 | - |
| $MW$ | mw_1 | 149000 | Da |
| $t_{1/2}$ | el_half_1 | 14 | days |
| $t_{1/2,\mathrm{a}}$ | abs_half | 2.5 | days |
| $BW$ | BW | 70 | kg |
| $V$ | volume_central | 5 | L |
| Valency | drug_valency_1 | 1 | - |
| $t_{1/2,\mathrm{L}}$ | lig_half_1 | 30 | min |
| $t_{1/2,\mathrm{R}}$ | rec_half_1 | 540 | min |

| | | | |
|---|---|---|---|
| $t_{1/2,\mathrm{sR}}$ | shed_half_1 | 0.5 | hr |
| $K_{D,\mathrm{L:R}}$ | lig_rec_kd_1 | 0.019 | nM |
| $C_{SS,\mathrm{L}}$ | lig_css_1_central | 0.0000575 | nM |
| $C_{SS,\mathrm{R}}$ | rec_css_1_central | 0.23 | nM |
| $C_{SS,\mathrm{sR}}$ | shed_css_1_central | 0 | nM |
| $\mathrm{Density}_{\mathrm{cells}}$ | cell_density_mL_central | 1000000 | #/mL |

| Parameter ID | Value |
|---|---|
| Scan Parameter 1 | Dose |
| Lower Limit 1 | 1 |
| Upper Limit 1 | 50000 |
| N1 | 6 |
| Scale 1 | log |

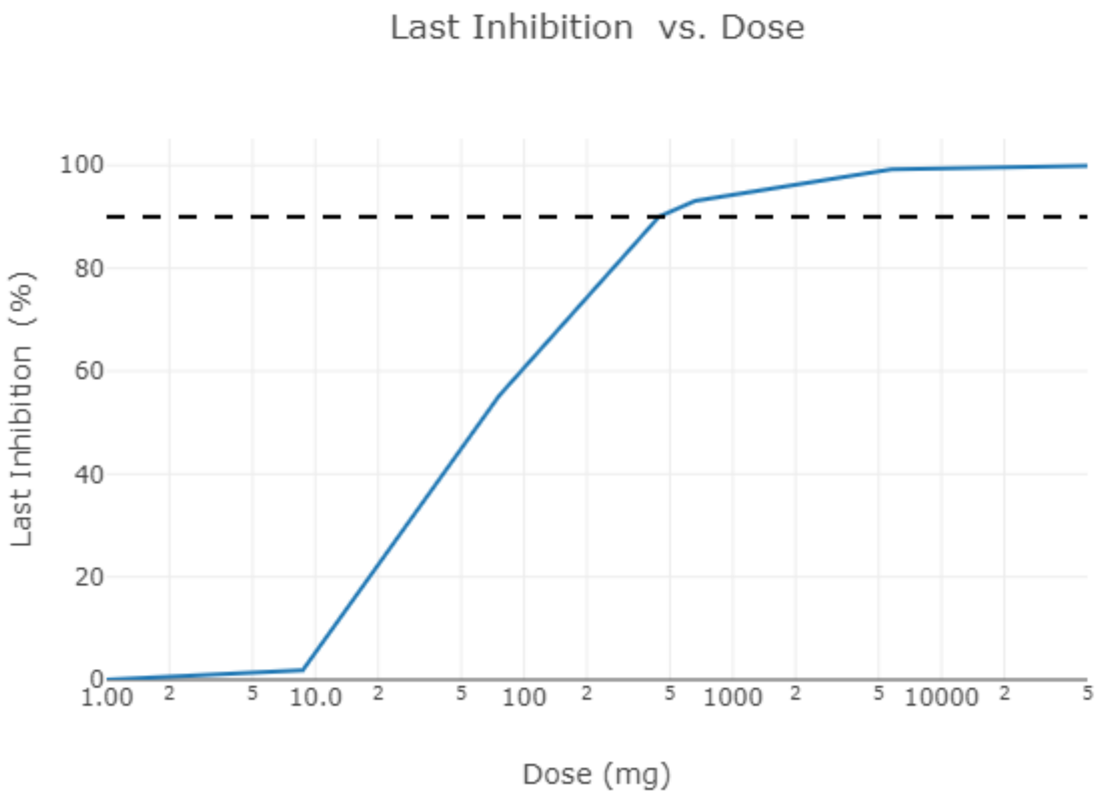


 © Applied BioMath LLC 2017-2021 

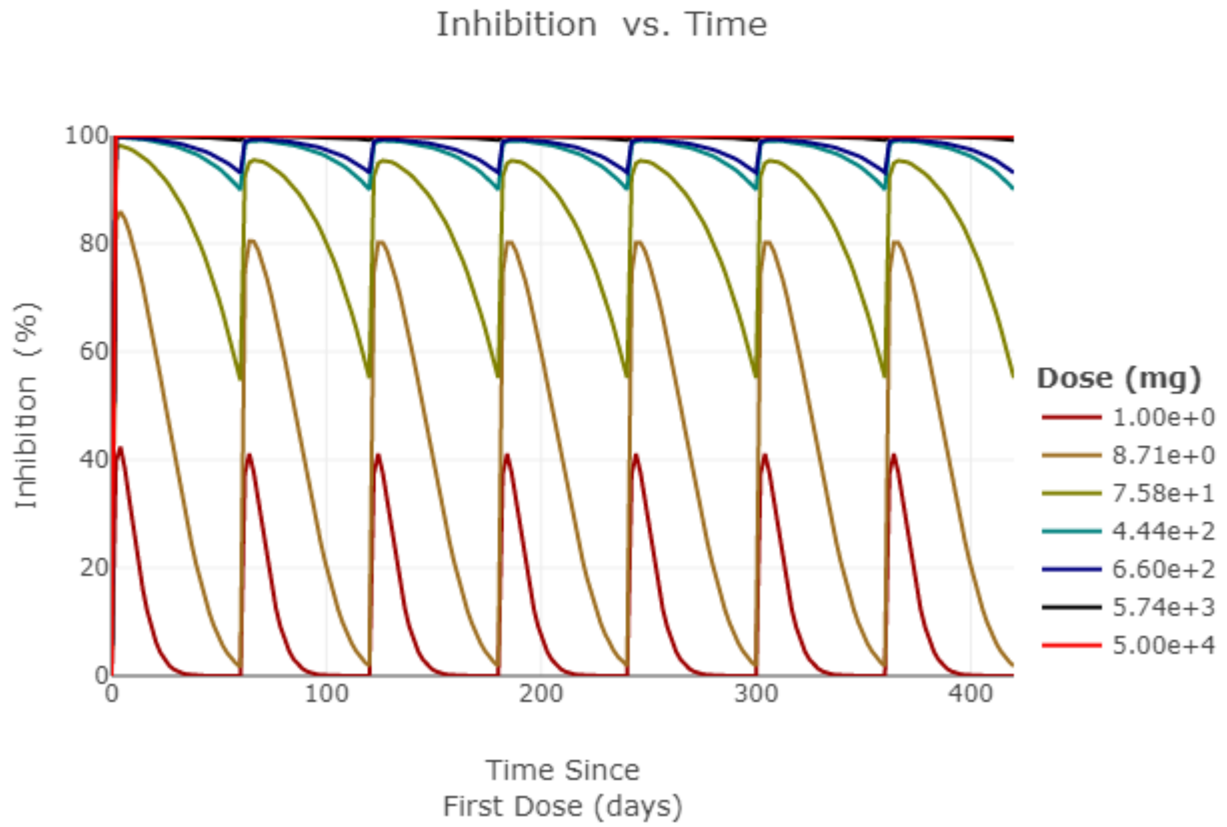


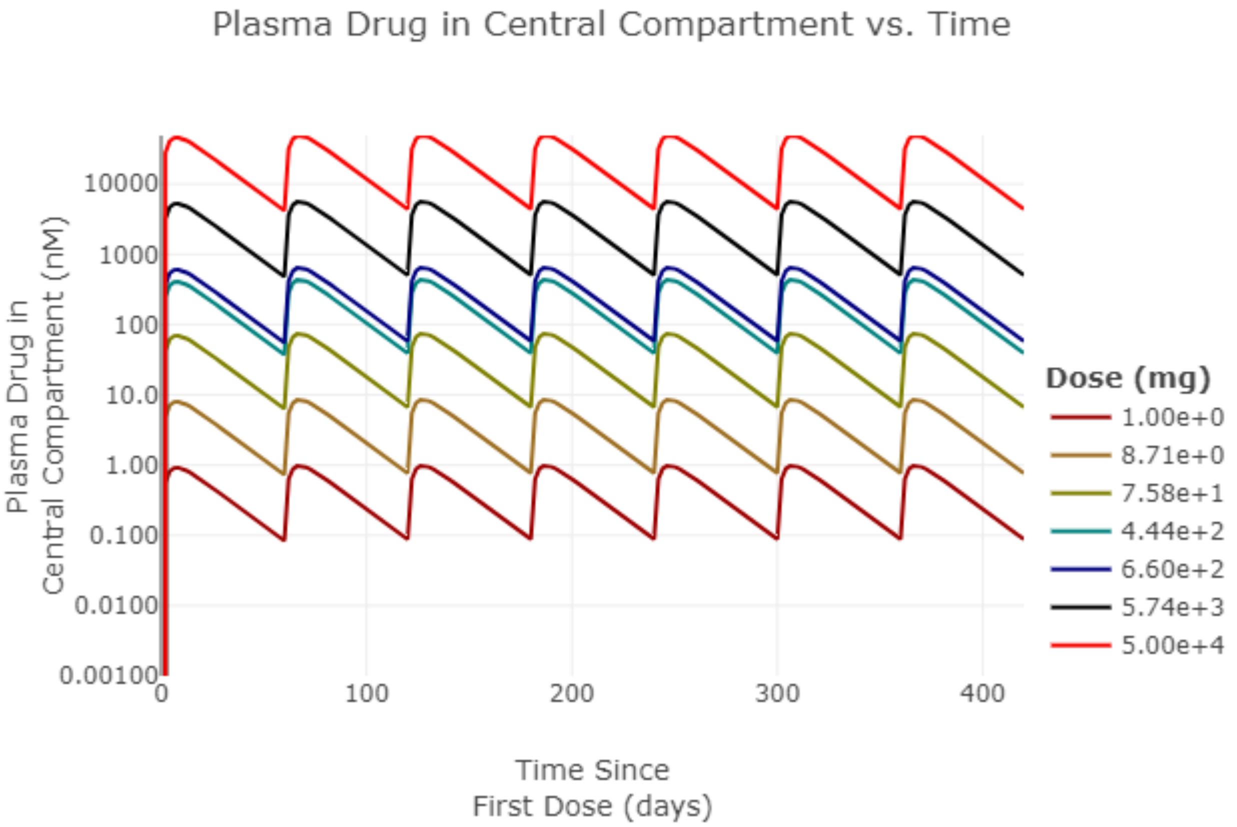


 © Applied BioMath LLC 2017-2021 

# 2.16) Scenario: rec_halflife_high

One dimensional scan over parameter, Dose, between 1 and 50000 using log spacing.

## 2.16.1) Model Parameters

Table 2.16.1

| Symbol | Parameter ID | Value | Unit |
|---|---|---|---|
| $\tau$ | interval | 60 | - |
| $D$ | dose | 100 | mg |
| $K_{D,\mathrm{L}}$ | mab_kd_1 | 0.0042 | nM |
| $N_{\mathrm{doses}}$ | dose_count | 7 | - |
| $MW$ | mw_1 | 149000 | Da |
| $t_{1/2}$ | el_half_1 | 14 | days |
| $t_{1/2,\mathrm{a}}$ | abs_half | 2.5 | days |
| $BW$ | BW | 70 | kg |
| $V$ | volume_central | 5 | L |
| $\mathrm{Valency}$ | drug_valency_1 | 1 | - |
| $t_{1/2,\mathrm{L}}$ | lig_half_1 | 30 | min |
| $t_{1/2,\mathrm{R}}$ | rec_half_1 | 1620 | min |
| $t_{1/2,\mathrm{sR}}$ | shed_half_1 | 0.5 | hr |
| $K_{D,\mathrm{L:R}}$ | lig_rec_kd_1 | 0.019 | nM |
| $C_{ss,\mathrm{L}}$ | lig_css_1_central | 0.0000575 | nM |
| $C_{ss,\mathrm{R}}$ | rec_css_1_central | 0.23 | nM |
| $C_{ss,\mathrm{sR}}$ | shed_css_1_central | 0 | nM |
| $\mathrm{Density}_{\mathrm{cells}}$ | cell_density_mL_central | 1000000 | #/mL |

| Parameter ID | Value |
|---|---|
| Scan Parameter 1 | Dose |
| Lower Limit 1 | 1 |
| Upper Limit 1 | 50000 |
| N1 | 6 |
| Scale 1 | log |

 © Applied BioMath LLC 2017-2021 

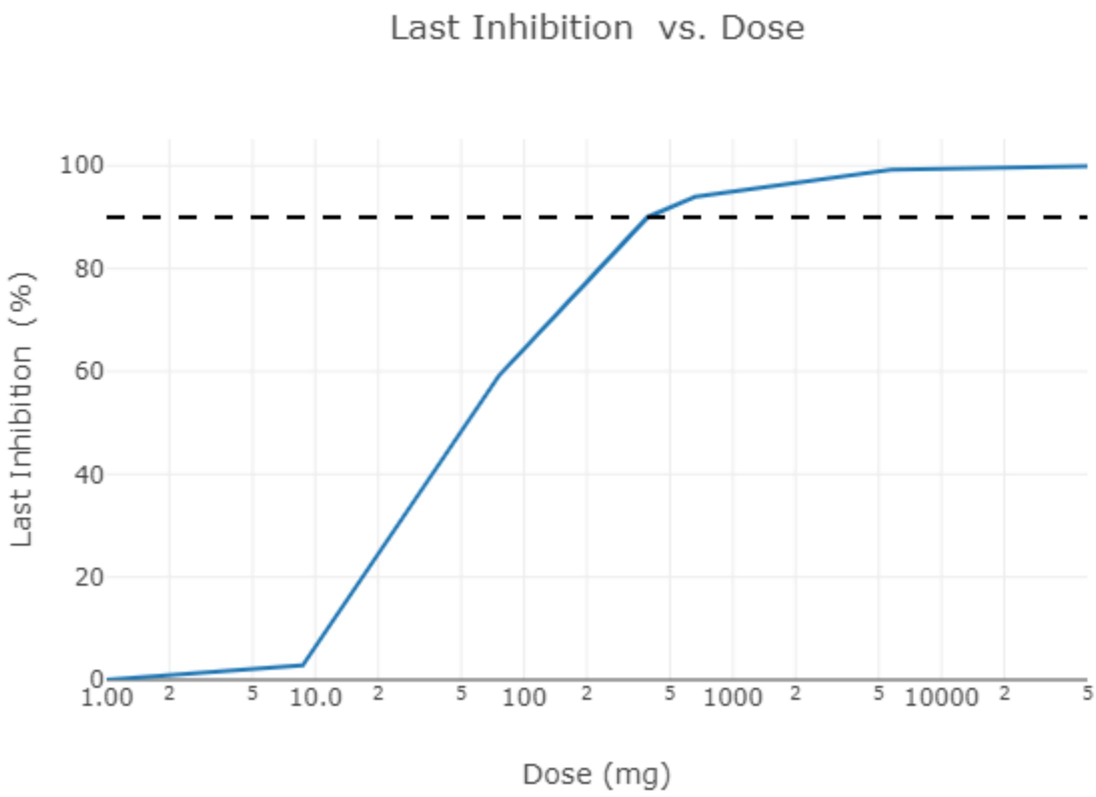


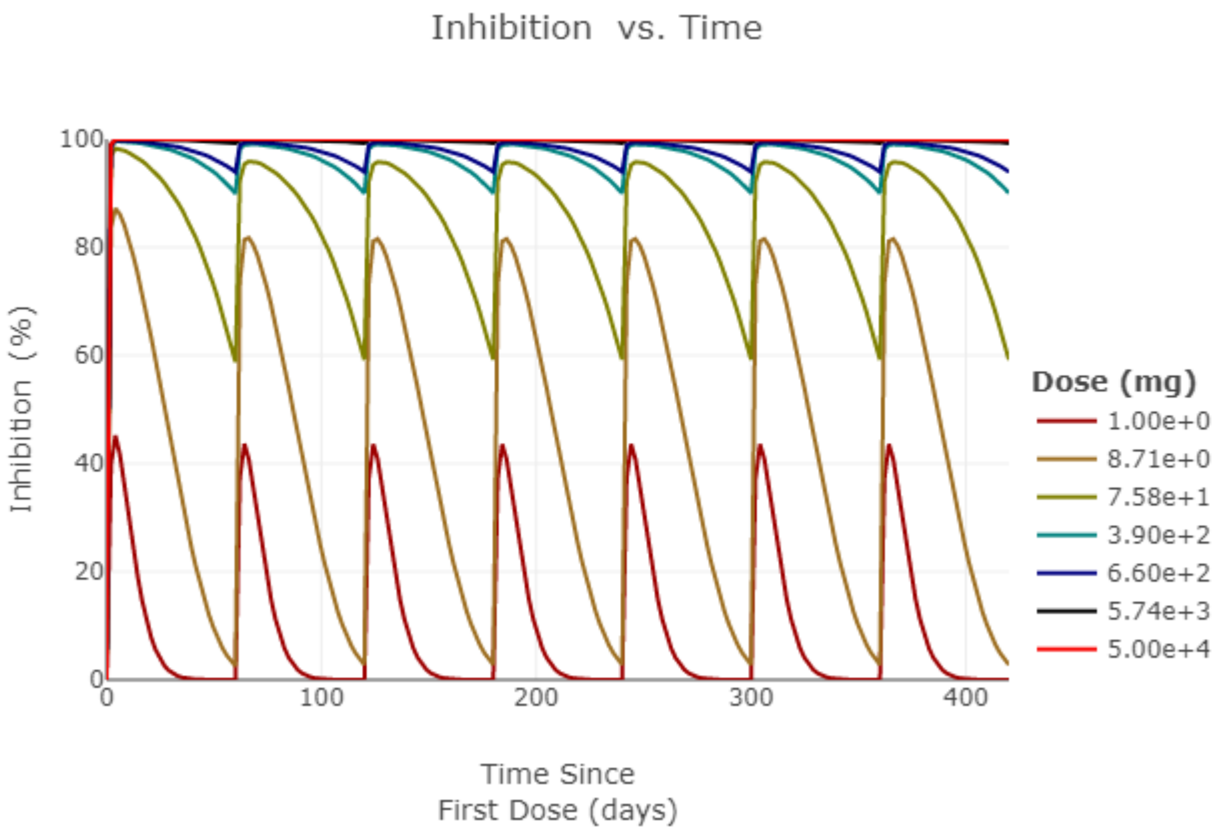


 © Applied BioMath LLC 2017-2021 

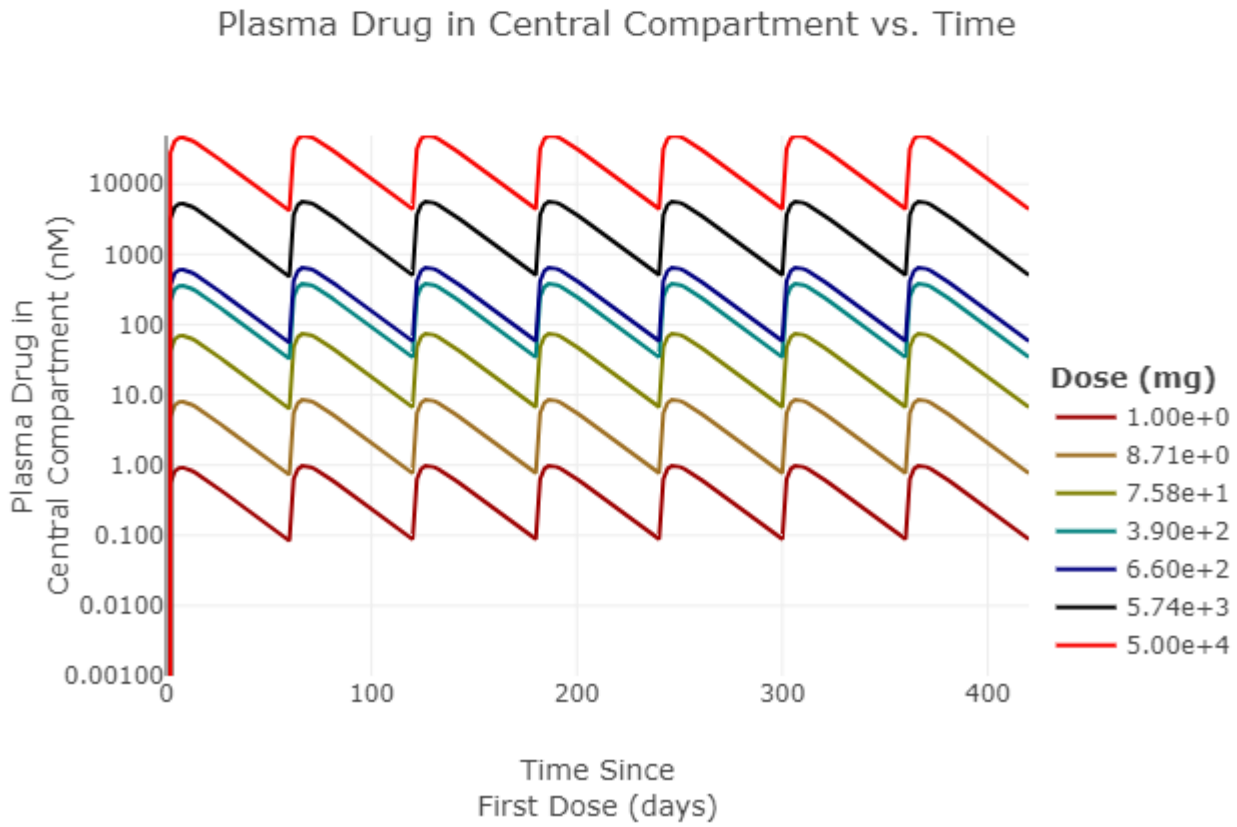


# 2.17) Scenario: rec_halflife_low

One dimensional scan over parameter, Dose, between 1 and 50000 using log spacing.

## 2.17.1) Model Parameters

Table 2.17.1

| Symbol | Parameter ID | Value | Unit |
|---|---|---|---|
| $\tau$ | interval | 60 | - |
| $D$ | dose | 100 | mg |
| $K_{D,\mathrm{L}}$ | mab_kd_1 | 0.0042 | nM |
| $N_{\mathrm{doses}}$ | dose_count | 7 | - |
| $MW$ | mw_1 | 149000 | Da |
| $t_{1/2}$ | el_half_1 | 14 | days |
| $t_{1/2,\mathrm{a}}$ | abs_half | 2.5 | days |
| $BW$ | BW | 70 | kg |
| $V$ | volume_central | 5 | L |
| Valency | drug_valency_1 | 1 | - |
| $t_{1/2,\mathrm{L}}$ | lig_half_1 | 30 | min |
| $t_{1/2,\mathrm{R}}$ | rec_half_1 | 180 | min |

| | | | |
|---|---|---|---|
| $t_{1/2,\mathrm{sR}}$ | shed_half_1 | 0.5 | hr |
| $K_{D,\mathrm{L:R}}$ | lig_rec_kd_1 | 0.019 | nM |
| $C_{SS,\mathrm{L}}$ | lig_css_1_central | 0.0000575 | nM |
| $C_{SS,\mathrm{R}}$ | rec_css_1_central | 0.23 | nM |
| $C_{SS,\mathrm{sR}}$ | shed_css_1_central | 0 | nM |
| $\mathrm{Density}_{\mathrm{cells}}$ | cell_density_mL_central | 1000000 | #/mL |

| Parameter ID | Value |
|---|---|
| Scan Parameter 1 | Dose |
| Lower Limit 1 | 1 |
| Upper Limit 1 | 50000 |
| N1 | 6 |
| Scale 1 | log |

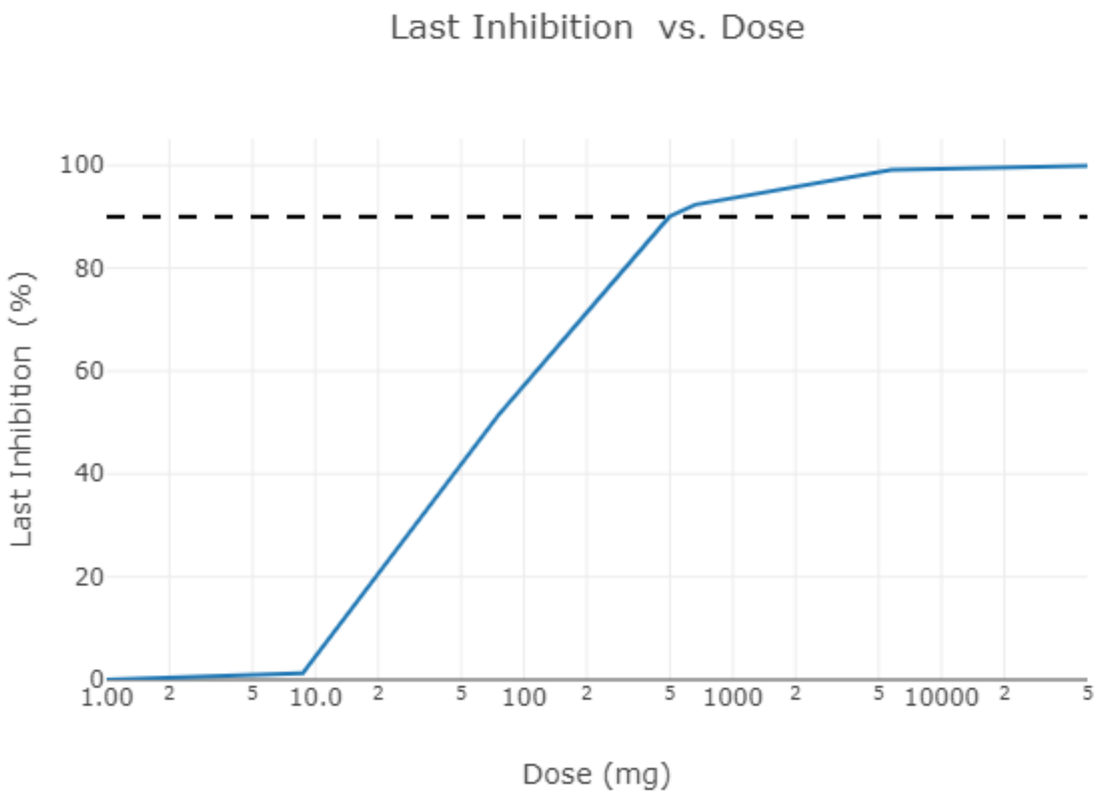

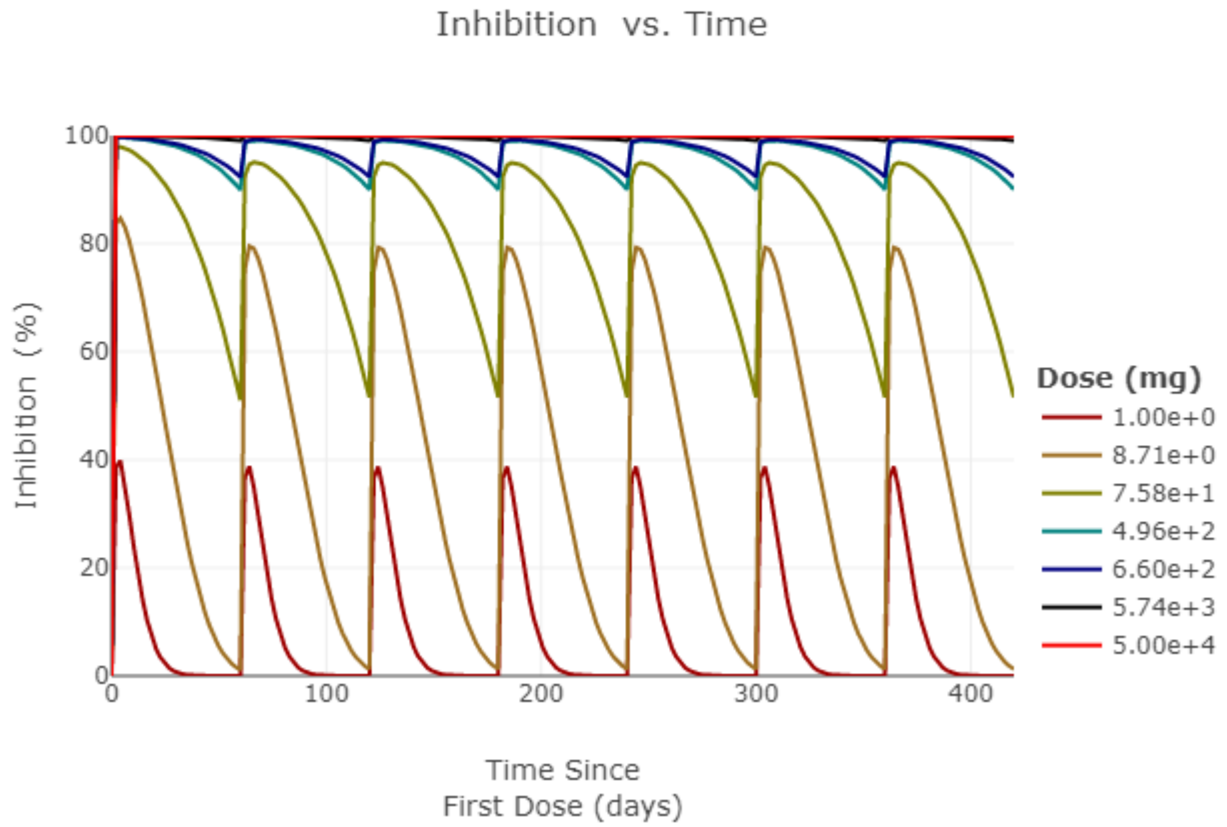
Inhibition vs. Time
Inhibition (%)
100
80
60
40
20
0
0
100
200
300
400
Time Since
First Dose (days)
Dose (mg)
1.00e+0
8.71e+0
7.58e+1
4.96e+2
6.60e+2
5.74e+3
5.00e+4

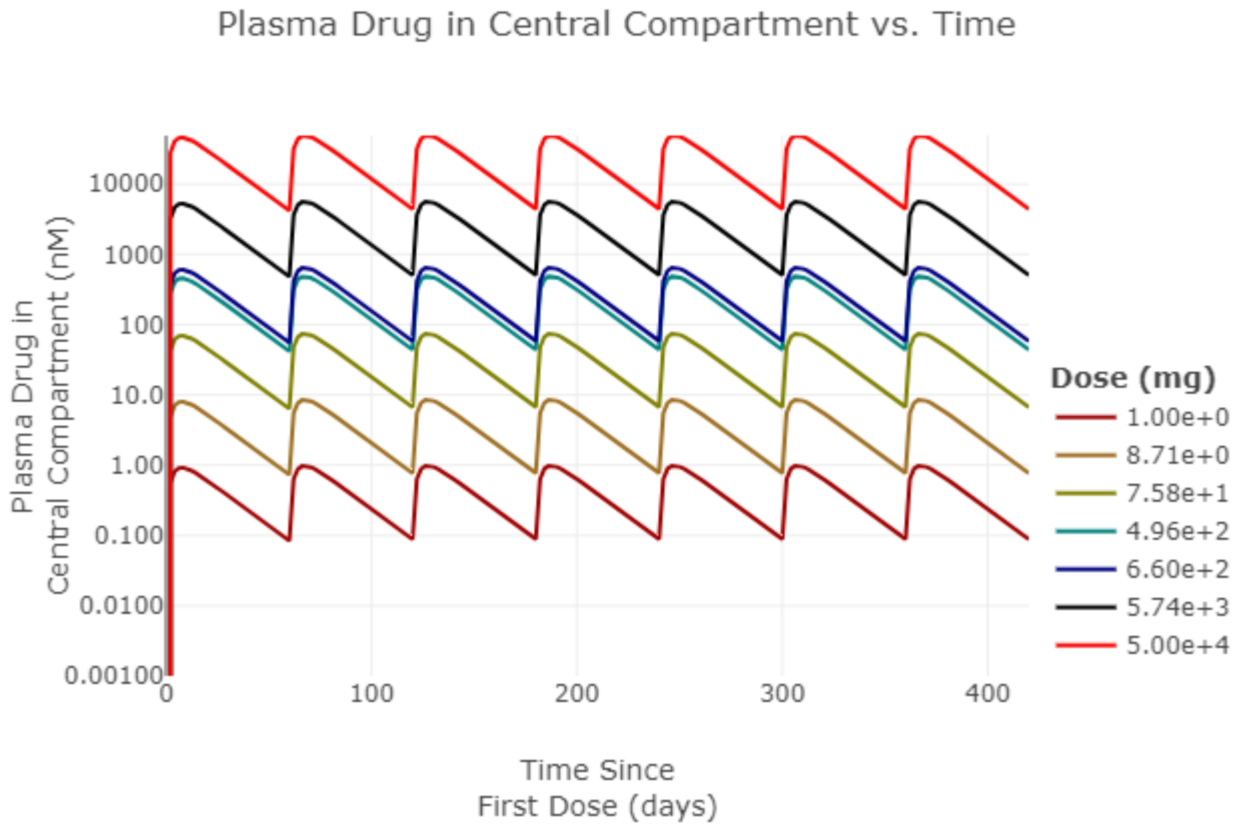
Plasma Drug in Central Compartment vs. Time
Plasma Drug in
Central Compartment (nM)
10000
1000
100
10.0
1.00
0.100
0.0100
0.00100
0
100
200
300
400
Time Since
First Dose (days)
Dose (mg)
1.00e+0
8.71e+0
7.58e+1
4.96e+2
6.60e+2
5.74e+3
5.00e+4

# 2.18) Scenario: Volume_high

One dimensional scan over parameter, Dose, between 1 and 50000 using log spacing.

## 2.18.1) Model Parameters

Table 2.18.1

| Symbol | Parameter ID | Value | Unit |
|---|---|---|---|
| $\tau$ | interval | 60 | - |
| $D$ | dose | 100 | mg |
| $K_{D,\mathrm{L}}$ | mab_kd_1 | 0.0042 | nM |
| $N_{\mathrm{doses}}$ | dose_count | 7 | - |
| $MW$ | mw_1 | 149000 | Da |
| $t_{1/2}$ | el_half_1 | 14 | days |
| $t_{1/2,\mathrm{a}}$ | abs_half | 2.5 | days |
| $BW$ | BW | 70 | kg |
| $V$ | volume_central | 15 | L |
| $\mathrm{Valency}$ | drug_valency_1 | 1 | - |
| $t_{1/2,\mathrm{L}}$ | lig_half_1 | 30 | min |
| $t_{1/2,\mathrm{R}}$ | rec_half_1 | 540 | min |
| $t_{1/2,\mathrm{sR}}$ | shed_half_1 | 0.5 | hr |
| $K_{D,\mathrm{L:R}}$ | lig_rec_kd_1 | 0.019 | nM |
| $C_{ss,\mathrm{L}}$ | lig_css_1_central | 0.0000575 | nM |
| $C_{ss,\mathrm{R}}$ | rec_css_1_central | 0.23 | nM |
| $C_{ss,\mathrm{sR}}$ | shed_css_1_central | 0 | nM |
| $\mathrm{Density}_{\mathrm{cells}}$ | cell_density_mL_central | 1000000 | #/mL |

| Parameter ID | Value |
|---|---|
| Scan Parameter 1 | Dose |
| Lower Limit 1 | 1 |
| Upper Limit 1 | 50000 |
| N1 | 6 |
| Scale 1 | log |

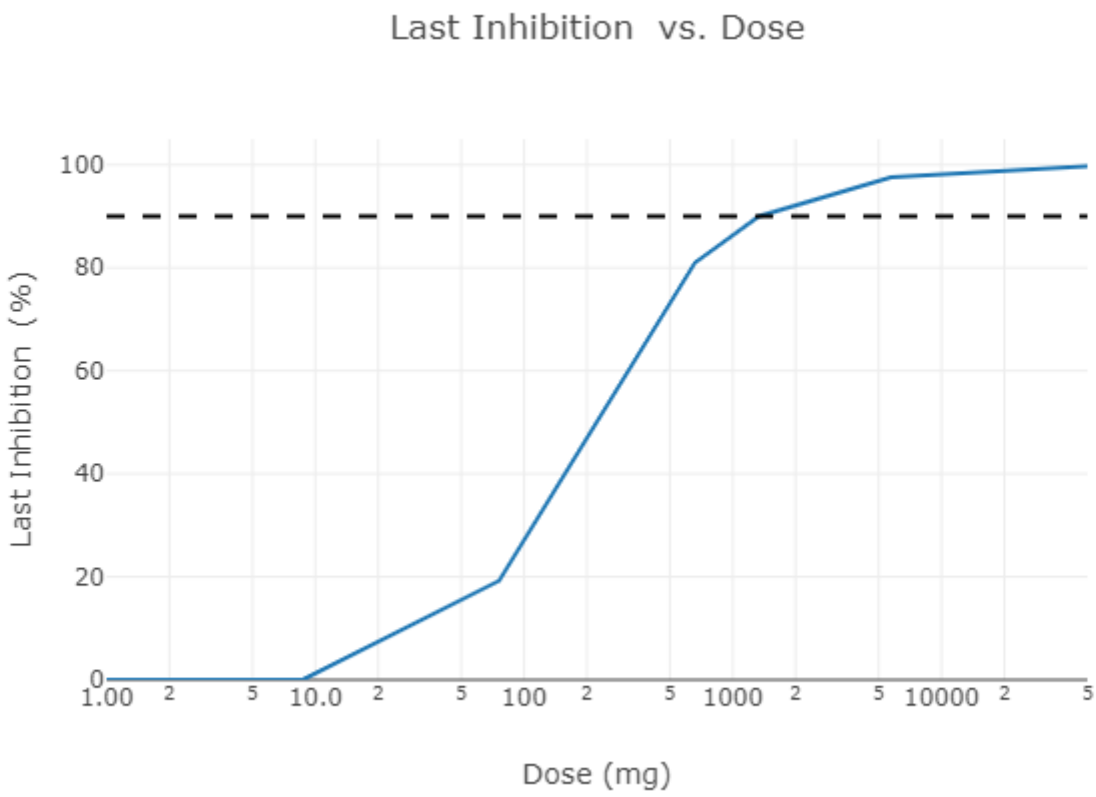
Last Inhibition vs. Dose
Last Inhibition (%)
100
80
60
40
20
0
1.00
2
5
10.0
2
5
100
2
5
1000
2
5
10000
2
5
Dose (mg)

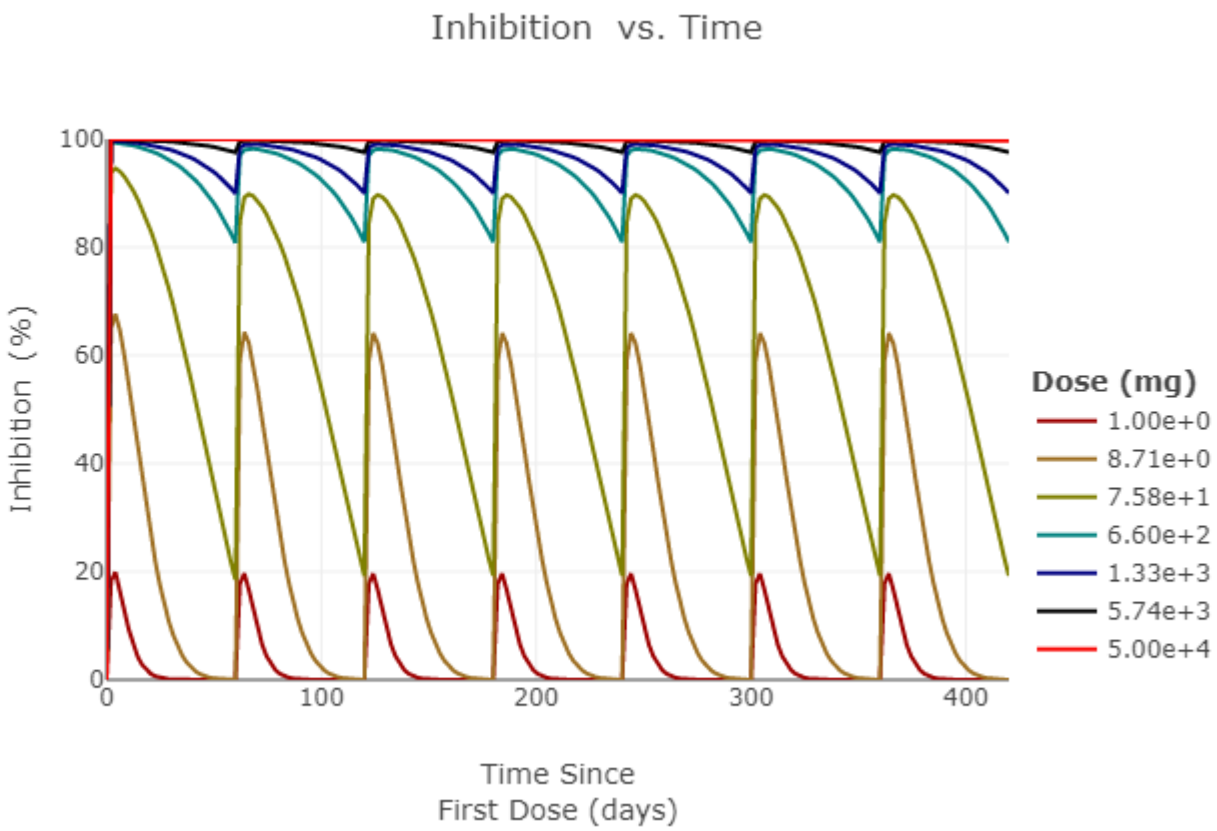
Inhibition vs. Time
Inhibition (%)
100
80
60
40
20
0
0
100
200
300
400
Time Since
First Dose (days)
Dose (mg)
1.00e+0
8.71e+0
7.58e+1
6.60e+2
1.33e+3
5.74e+3
5.00e+4

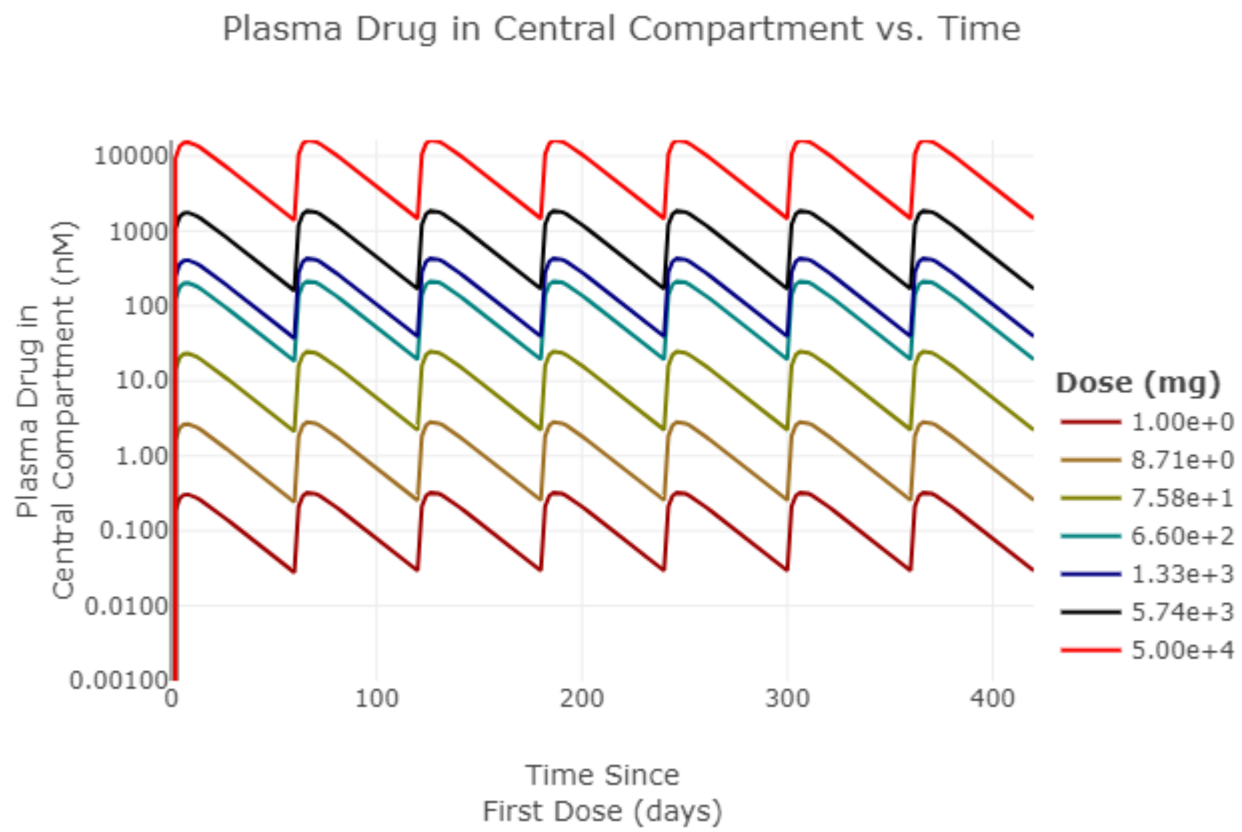


## 2.19) Scenario: Volume_low

One dimensional scan over parameter, Dose, between 1 and 50000 using log spacing.

### 2.19.1) Model Parameters

Table 2.19.1

| Symbol | Parameter ID | Value | Unit |
|---|---|---|---|
| $\tau$ | interval | 60 | - |
| $D$ | dose | 100 | mg |
| $K_{D,\mathrm{L}}$ | mab_kd_1 | 0.0042 | nM |
| $N_{\mathrm{doses}}$ | dose_count | 7 | - |
| $MW$ | mw_1 | 149000 | Da |
| $t_{1/2}$ | el_half_1 | 14 | days |
| $t_{1/2,\mathrm{a}}$ | abs_half | 2.5 | days |
| $BW$ | BW | 70 | kg |
| $V$ | volume_central | 1.67 | L |
| Valency | drug_valency_1 | 1 | - |
| $t_{1/2,\mathrm{L}}$ | lig_half_1 | 30 | min |
| $t_{1/2,\mathrm{R}}$ | rec_half_1 | 540 | min |

| | | | |
|---|---|---|---|
| $t_{1/2,\mathrm{sR}}$ | shed_half_1 | 0.5 | hr |
| $K_{D,\mathrm{L:R}}$ | lig_rec_kd_1 | 0.019 | nM |
| $C_{SS,\mathrm{L}}$ | lig_css_1_central | 0.0000575 | nM |
| $C_{SS,\mathrm{R}}$ | rec_css_1_central | 0.23 | nM |
| $C_{SS,\mathrm{sR}}$ | shed_css_1_central | 0 | nM |
| $\mathrm{Density}_{\mathrm{cells}}$ | cell_density_mL_central | 1000000 | #/mL |

| Parameter ID | Value |
|---|---|
| Scan Parameter 1 | Dose |
| Lower Limit 1 | 1 |
| Upper Limit 1 | 50000 |
| N1 | 6 |
| Scale 1 | log |

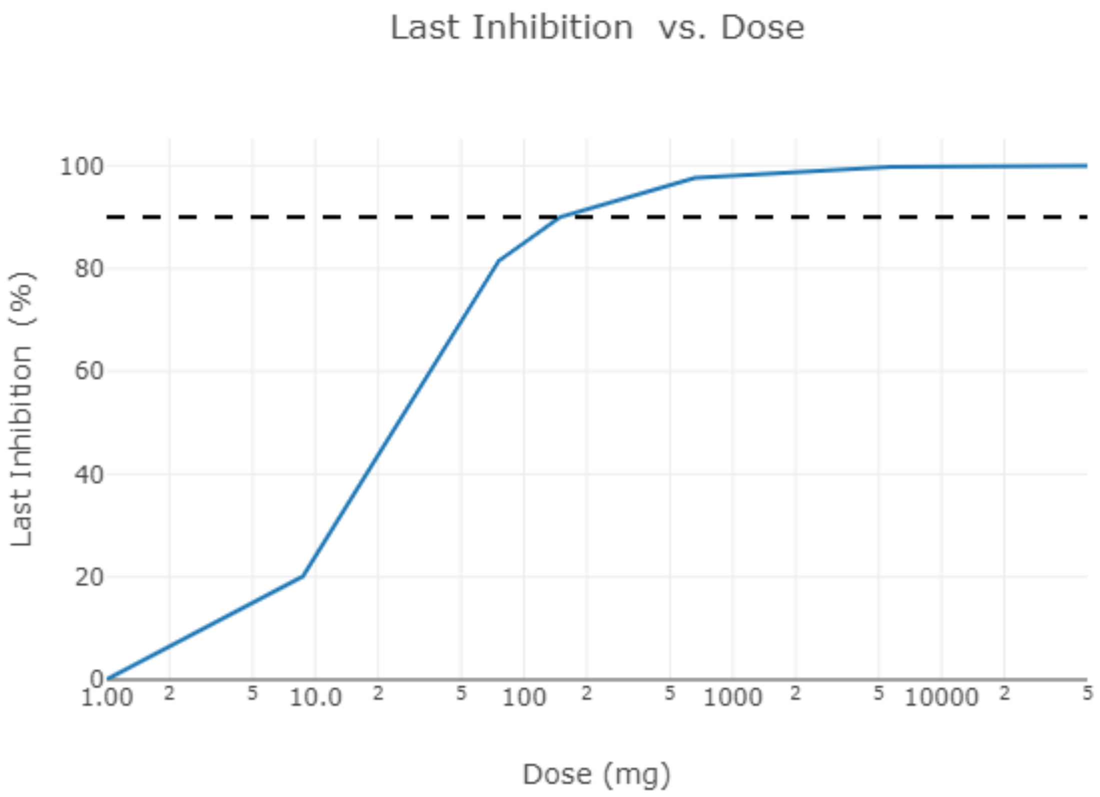

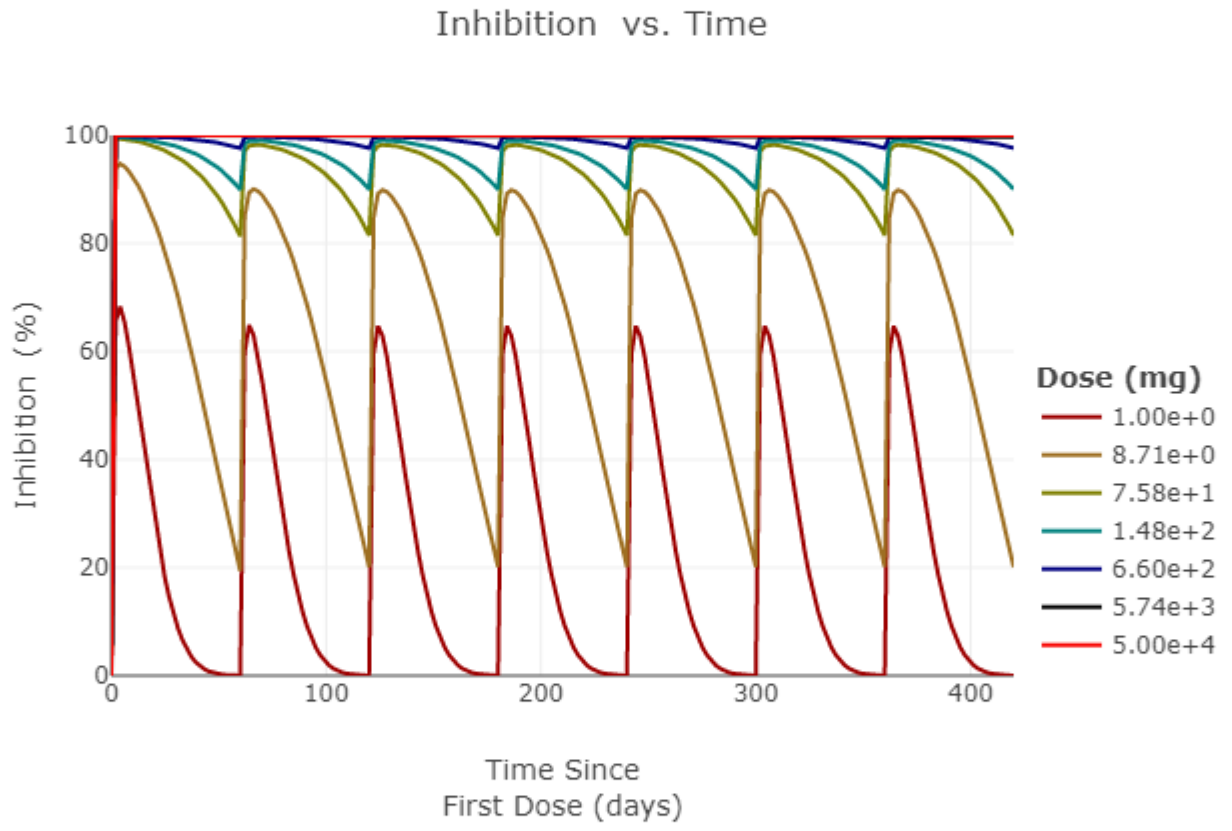


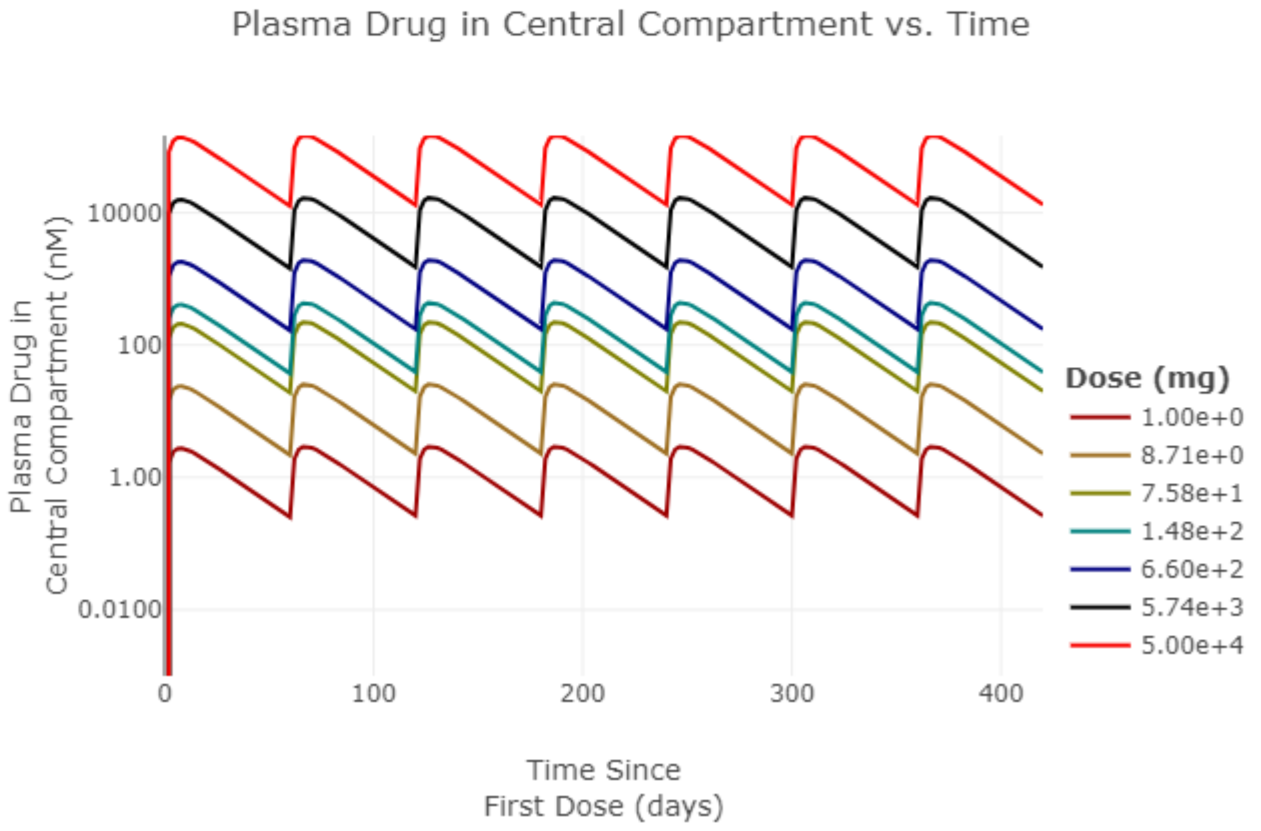


 © Applied BioMath LLC 2017-2021 

# 2.20) Scenario: MW_high

One dimensional scan over parameter, Dose, between 1 and 50000 using log spacing.

## 2.20.1) Model Parameters

Table 2.20.1

| Symbol | Parameter ID | Value | Unit |
|---|---|---|---|
| $\tau$ | interval | 60 | - |
| $D$ | dose | 100 | mg |
| $K_{D,\mathrm{L}}$ | mab_kd_1 | 0.0042 | nM |
| $N_{\mathrm{doses}}$ | dose_count | 7 | - |
| $MW$ | mw_1 | 447000 | Da |
| $t_{1/2}$ | el_half_1 | 14 | days |
| $t_{1/2,\mathrm{a}}$ | abs_half | 2.5 | days |
| $BW$ | BW | 70 | kg |
| $V$ | volume_central | 5 | L |
| $\mathrm{Valency}$ | drug_valency_1 | 1 | - |
| $t_{1/2,\mathrm{L}}$ | lig_half_1 | 30 | min |
| $t_{1/2,\mathrm{R}}$ | rec_half_1 | 540 | min |
| $t_{1/2,\mathrm{sR}}$ | shed_half_1 | 0.5 | hr |
| $K_{D,\mathrm{L:R}}$ | lig_rec_kd_1 | 0.019 | nM |
| $C_{ss,\mathrm{L}}$ | lig_css_1_central | 0.0000575 | nM |
| $C_{ss,\mathrm{R}}$ | rec_css_1_central | 0.23 | nM |
| $C_{ss,\mathrm{sR}}$ | shed_css_1_central | 0 | nM |
| $\mathrm{Density}_{\mathrm{cells}}$ | cell_density_mL_central | 1000000 | #/mL |

| Parameter ID | Value |
|---|---|
| Scan Parameter 1 | Dose |
| Lower Limit 1 | 1 |
| Upper Limit 1 | 50000 |
| N1 | 6 |
| Scale 1 | log |

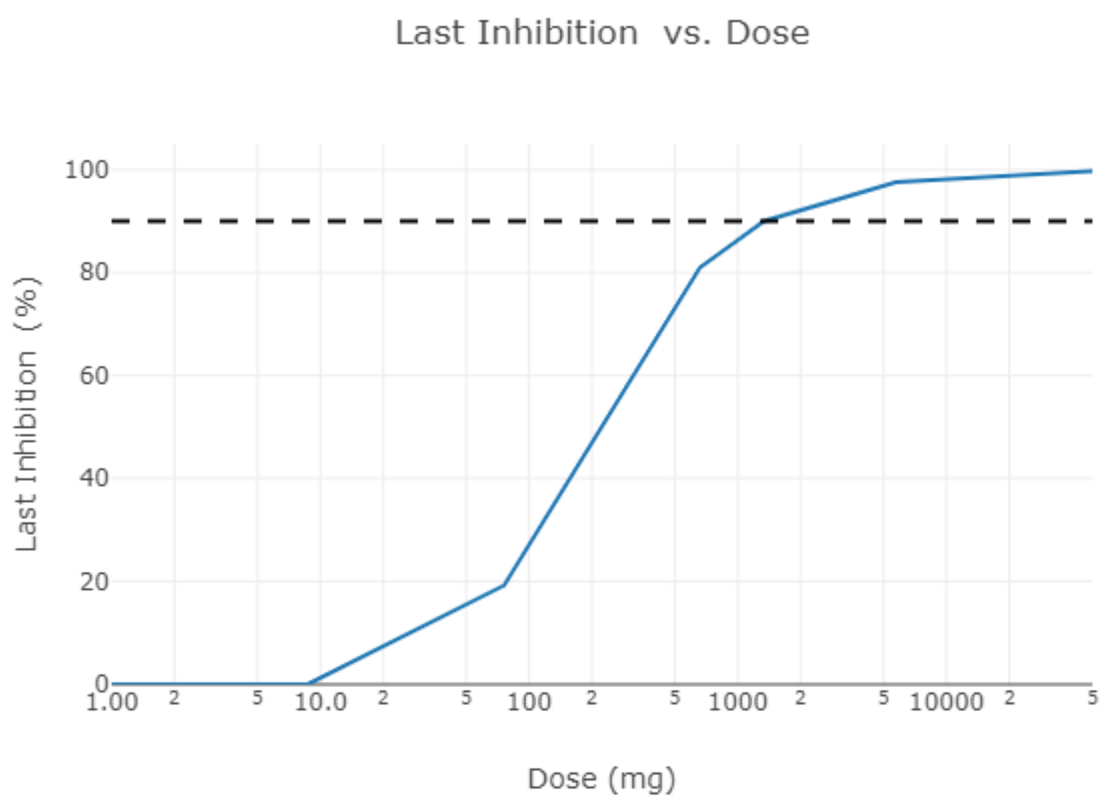
Last Inhibition vs. Dose
Last Inhibition (%)
100
80
60
40
20
0
1.00
2
5
10.0
2
5
100
2
5
1000
2
5
10000
2
5
Dose (mg)

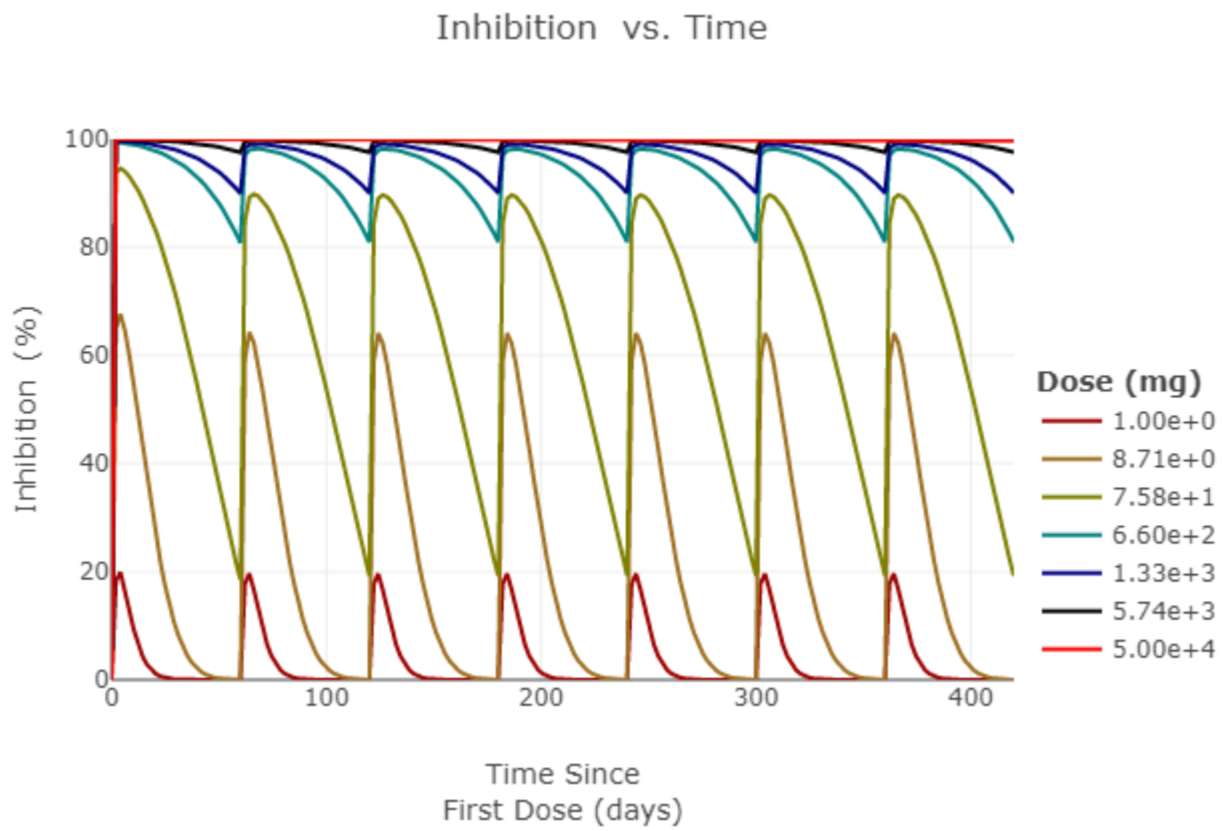
Inhibition vs. Time
Inhibition (%)
100
80
60
40
20
0
0
100
200
300
400
Time Since
First Dose (days)
Dose (mg)
1.00e+0
8.71e+0
7.58e+1
6.60e+2
1.33e+3
5.74e+3
5.00e+4

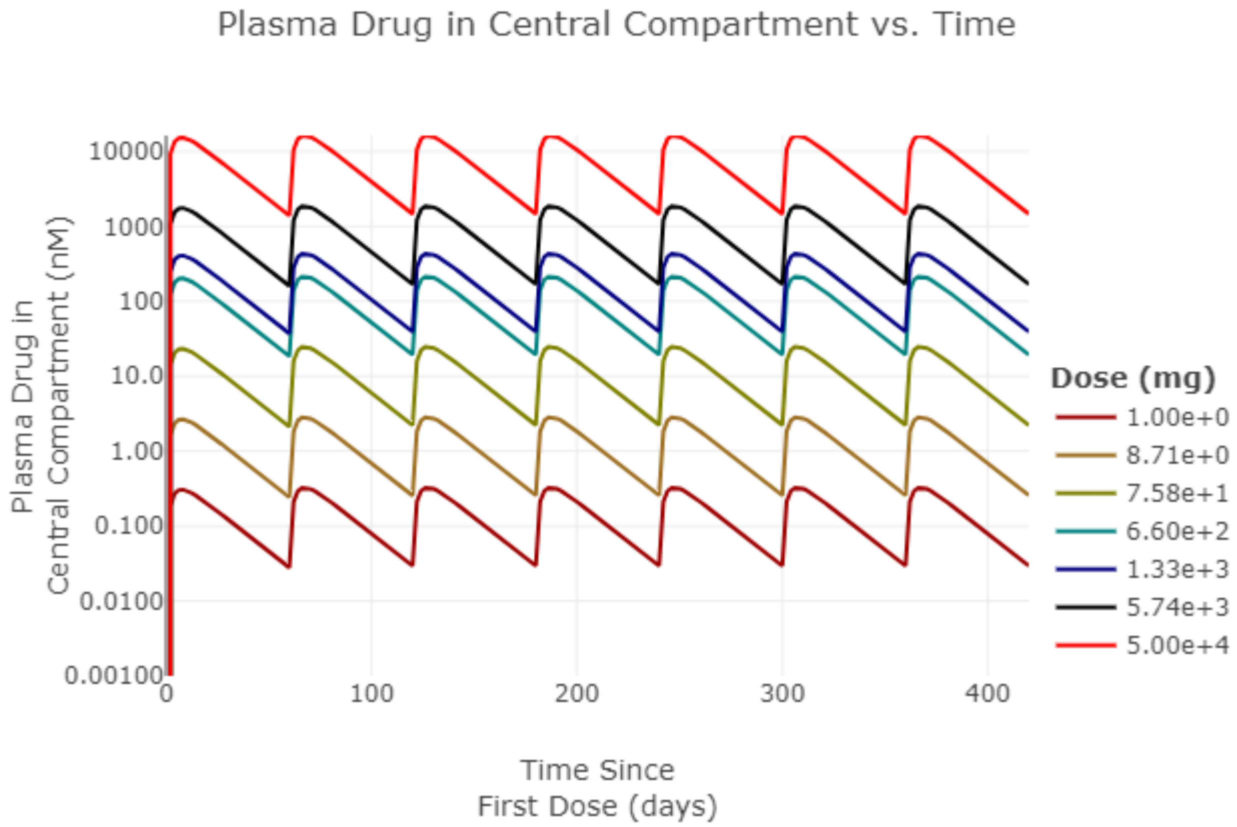


## 2.21) Scenario: MW_low

One dimensional scan over parameter, Dose, between 1 and 50000 using log spacing.

### 2.21.1) Model Parameters

Table 2.21.1

| Symbol | Parameter ID | Value | Unit |
|---|---|---|---|
| $\tau$ | interval | 60 | - |
| $D$ | dose | 100 | mg |
| $K_{D,\mathrm{L}}$ | mab_kd_1 | 0.0042 | nM |
| $N_{\mathrm{doses}}$ | dose_count | 7 | - |
| $MW$ | mw_1 | 49666.67 | Da |
| $t_{1/2}$ | el_half_1 | 14 | days |
| $t_{1/2,\mathrm{a}}$ | abs_half | 2.5 | days |
| $BW$ | BW | 70 | kg |
| $V$ | volume_central | 5 | L |
| Valency | drug_valency_1 | 1 | - |
| $t_{1/2,\mathrm{L}}$ | lig_half_1 | 30 | min |
| $t_{1/2,\mathrm{R}}$ | rec_half_1 | 540 | min |

 © Applied BioMath LLC 2017-2021 

| | | | |
|---|---|---|---|
| $t_{1/2,\mathrm{sR}}$ | shed_half_1 | 0.5 | hr |
| $K_{D,\mathrm{L:R}}$ | lig_rec_kd_1 | 0.019 | nM |
| $C_{SS,\mathrm{L}}$ | lig_css_1_central | 0.0000575 | nM |
| $C_{SS,\mathrm{R}}$ | rec_css_1_central | 0.23 | nM |
| $C_{SS,\mathrm{sR}}$ | shed_css_1_central | 0 | nM |
| $\mathrm{Density}_{\mathrm{cells}}$ | cell_density_mL_central | 1000000 | #/mL |

| Parameter ID | Value |
|---|---|
| Scan Parameter 1 | Dose |
| Lower Limit 1 | 1 |
| Upper Limit 1 | 50000 |
| N1 | 6 |
| Scale 1 | log |

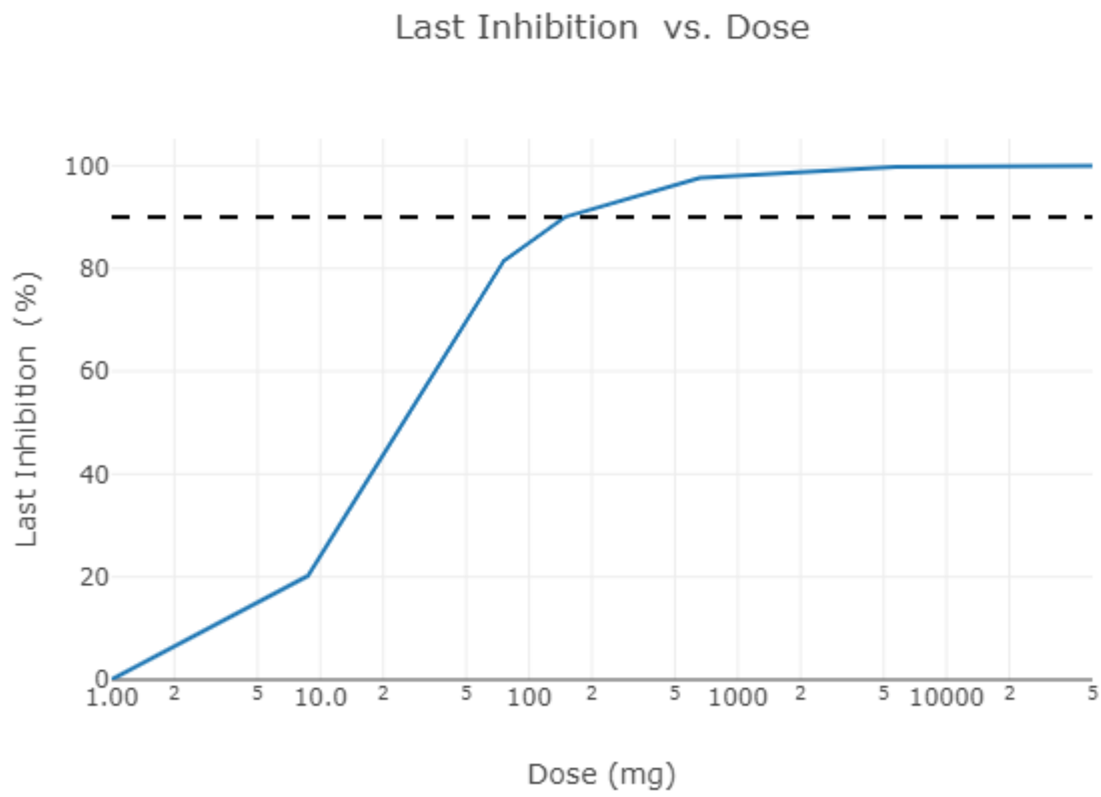

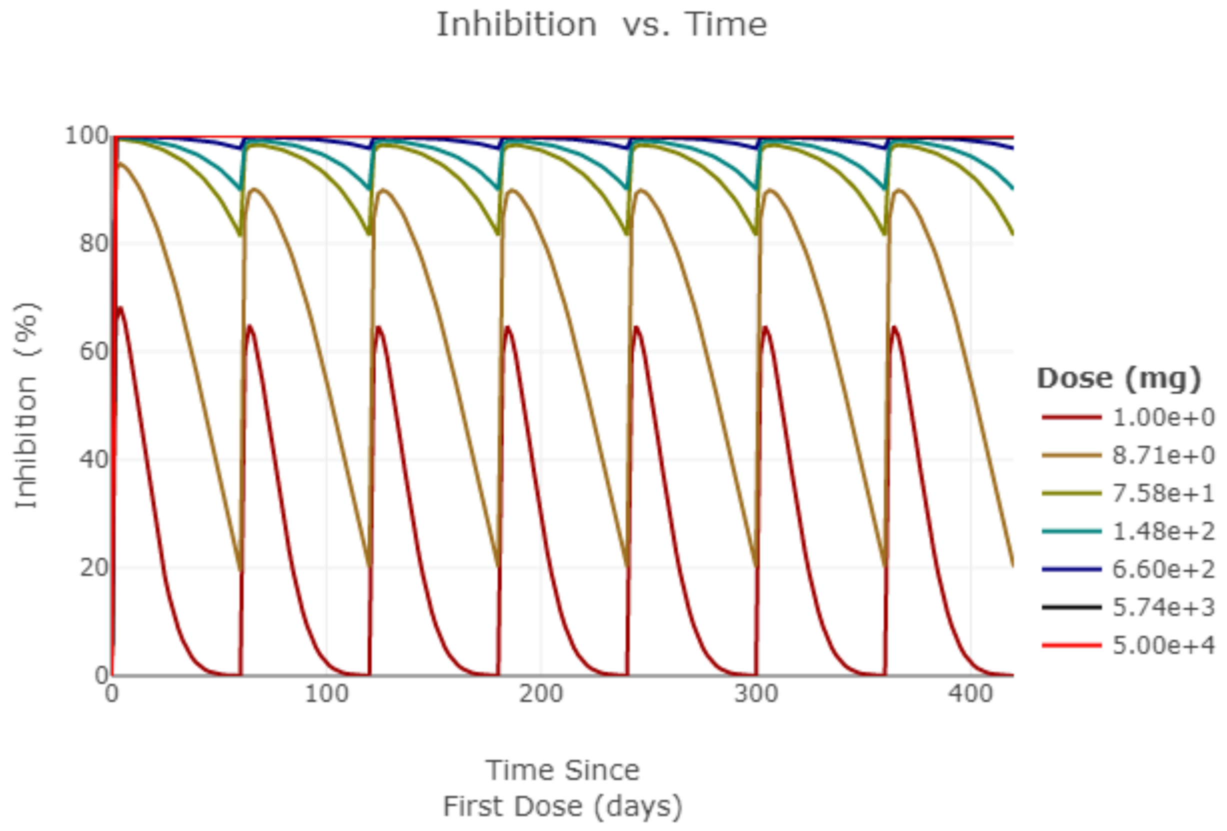


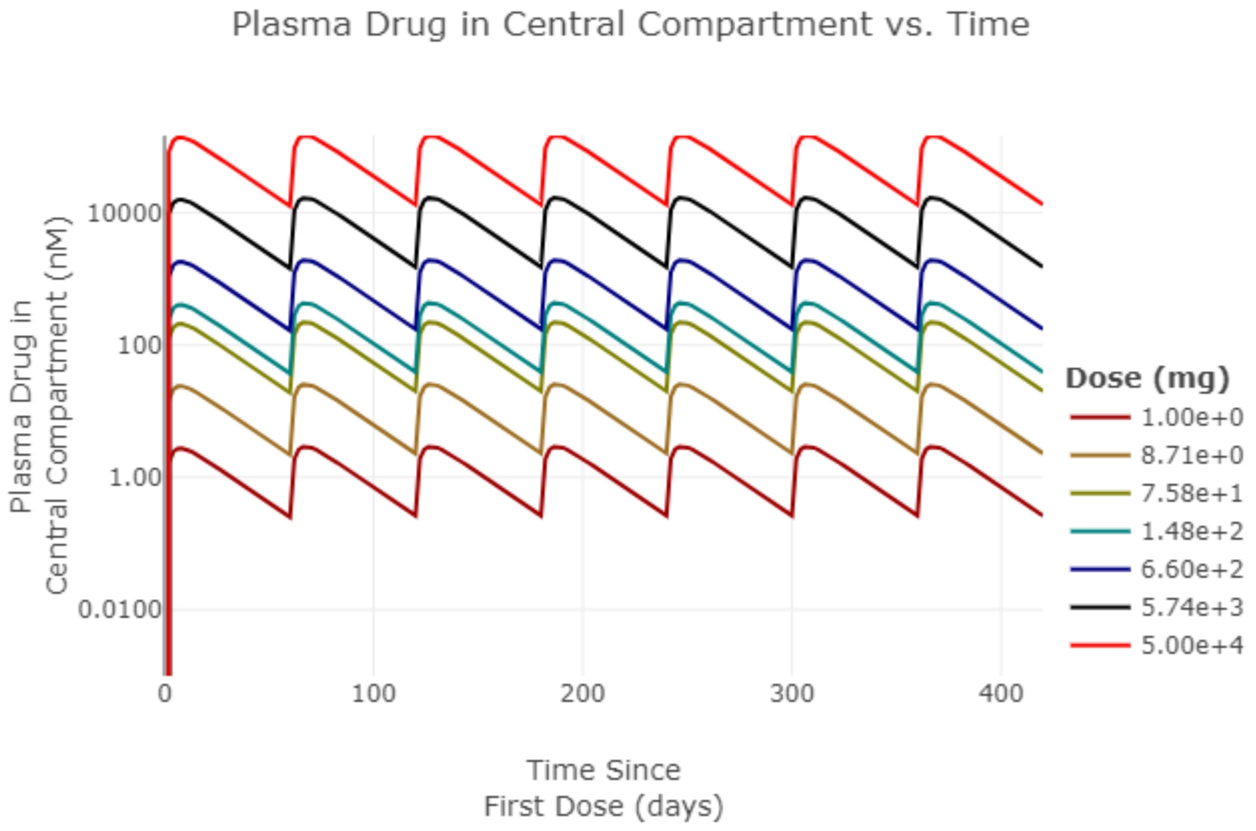


 © Applied BioMath LLC 2017-2021
