# Supplementary material for: Early Feasibility Assessment: A Method for Accurately Predicting Biotherapeutic Dosing to Inform Early Drug Discovery Decisions
Source: Front Pharmacol. 2022 Jun 8;13:864768. doi: 10.3389/fphar.2022.864768 (PMC9214263; doi:10.3389/fphar.2022.864768)
Supplement: Supplementary file 3 [file DataSheet2.ZIP › Model run files_json and reports/Adalimumab_Sensitivity_Analysis.pdf]

Adalimumab Parameter Scans
generated with
Applied BioMath Assess™

Model: Monospecific Anti-Ligand

Date: 2022-03-10T21:59:26.029Z

Software Version: 2022.2.2

This report does not fall within the scope of the United States Food and Drug Administration Good Laboratory Practice or Good Clinical Practice Regulations.

1) Summary

The goal of this study is to determine the feasibility of a drug concept for a target by varying parameters of a Quantitative Systems Pharmacology (QSP) model. By performing simulations over a range of drug and target parameters, this study supports comparison of the effects of dose amount, dose frequency, route of administration, drug design criteria, and target characteristics. This assessment can aid in the prediction of difficulty of discovering and formulation a New Biological Entity that satisfies a Target Product Profile.

The analyses explore the model conditions under which one or more criteria are satisfied. Depending on the kind of analysis, the feasibility of a scenario is defined to be either:

- the dose that exactly satisfies the criteria targets,
- a range of a parameter that satisfies the criteria targets,
- or a region of a parameter grid that satisfies the criteria targets.

The model used in these analyses is: Monospecific Anti-Ligand - A biologic that binds to a soluble ligand target and prevents the ligand from binding to its cognate-receptor. The molecule can be mono- or bivalent. This is a one-compartment model and there is an option for +/- soluble receptor.

Scenario Table

Description	Route	Dose (mg)	Drug:Target KD (nM)	Biologic first order T 1/2 (days)	SC absorption T 1/2 (days)	Volume (L)	Effective Valency (undefined)	Ligand T 1/2 (min)	Receptor T 1/2 (min)	L:R Affinity (nM)	Ligand CSS (nM)	Receptor CSS (nM)	Scan Parameter	Criterion	Result
Nominal	SC	-	0.008	20	2.5	5	1	30	540	0.019	0.0000575	0.23	: Dose (mg) [1 - 300]	Last Inhibition ≥ 90 %	Dose ≥ 39.4 mg
abs_half_high	SC	-	0.008	20	0.83	5	1	30	540	0.019	0.0000575	0.23	: Dose (mg) [1 - 300]	Last Inhibition ≥ 90 %	Dose ≥ 42.8 mg
abs_half_low	SC	-	0.008	20	7.5	5	1	30	540	0.019	0.0000575	0.23	: Dose (mg) [1 - 300]	Last Inhibition ≥ 90 %	Dose ≥ 36.5 mg
valency_high	SC	-	0.008	20	2.5	5	2	30	540	0.019	0.0000575	0.23	: Dose (mg) [1 - 300]	Last Inhibition ≥ 90 %	Dose ≥ 19.7 mg
drug halflife_high	SC	-	0.008	60	2.5	5	1	30	540	0.019	0.0000575	0.23	: Dose (mg) [1 - 300]	Last Inhibition ≥ 90 %	Dose ≥ 35.2 mg
drug halflife_low	SC	-	0.008	6.67	2.5	5	1	30	540	0.019	0.0000575	0.23	: Dose (mg) [1 - 300]	Last Inhibition ≥ 90 %	Dose ≥ 57.0 mg
TNF_conc_high	SC	-	0.008	20	2.5	5	1	30	540	0.019	0.000173	0.23	: Dose (mg) [1 - 300]	Last Inhibition ≥ 90 %	Dose ≥ 39.6 mg
TNF_conc_low	SC	-	0.008	20	2.5	5	1	30	540	0.019	0.0000192	0.23	: Dose (mg) [1 - 300]	Last Inhibition ≥ 90 %	Dose ≥ 39.4 mg
ligand_halflife_high	SC	-	0.008	20	2.5	5	1	90	540	0.019	0.0000575	0.23	: Dose (mg) [1 - 300]	Last Inhibition ≥ 90 %	Dose ≥ 19.5 mg
ligand_halflife_low	SC	-	0.008	20	2.5	5	1	10	540	0.019	0.0000575	0.23	: Dose (mg) [1 - 300]	Last Inhibition ≥ 90 %	Dose ≥ 99.4 mg
ligand:rec affinity high	SC	-	0.008	20	2.5	5	1	30	540	0.006	0.0000575	0.23	: Dose (mg) [1 - 300]	Last Inhibition ≥ 90 %	Dose ≥ 43.7 mg

© Applied BioMath LLC 2017-2021

ligand:rec affinity low	SC	-	0.008	20	2.5	5	1	30	540	0.057	0.0000575	0.23	: Dose (mg) [1 - 300]	Last Inhibition ≥ 90 %	Dose ≥ 35.0 mg
drug_affinity_high	SC	-	0.0027	20	2.5	5	1	30	540	0.019	0.0000575	0.23	: Dose (mg) [1 - 300]	Last Inhibition ≥ 90 %	Dose ≥ 14.6 mg
drug_affinity_low	SC	-	0.024	20	2.5	5	1	30	540	0.019	0.0000575	0.23	: Dose (mg) [1 - 300]	Last Inhibition ≥ 90 %	Dose ≥ 114 mg
receptor_conc_high	SC	-	0.008	20	2.5	5	1	30	540	0.019	0.0000575	0.69	: Dose (mg) [1 - 300]	Last Inhibition ≥ 90 %	Dose ≥ 58.4 mg
receptor_conc_low	SC	-	0.008	20	2.5	5	1	30	540	0.019	0.0000575	0.23	: Dose (mg) [1 - 300]	Last Inhibition ≥ 90 %	Dose ≥ 39.4 mg
rec_halflife_high	SC	-	0.008	20	2.5	5	1	30	1620	0.019	0.0000575	0.23	: Dose (mg) [1 - 300]	Last Inhibition ≥ 90 %	Dose ≥ 34.7 mg
rec_halflife_low	SC	-	0.008	20	2.5	5	1	30	180	0.019	0.0000575	0.23	: Dose (mg) [1 - 300]	Last Inhibition ≥ 90 %	Dose ≥ 44.0 mg
Volume_high	SC	-	0.008	20	2.5	15	1	30	540	0.019	0.0000575	0.23	: Dose (mg) [1 - 300]	Last Inhibition ≥ 90 %	Dose ≥ 118 mg
Volume_low	SC	-	0.008	20	2.5	1.67	1	30	540	0.019	0.0000575	0.23	: Dose (mg) [1 - 300]	Last Inhibition ≥ 90 %	Dose ≥ 13.2 mg

© Applied BioMath LLC 2017-2021

2) Results

2.0) Scenario: Nominal

One dimensional scan over parameter, Dose, between 1 and 300 using log spacing.

2.0.1) Model Parameters

Table 2.0.1

Symbol	Parameter ID	Value	Unit
τ	interval	14	-
D	dose	100	mg
$K_{D,\mathrm{L}}$	mab_kd_1	0.008	nM
N_{doses}	dose_count	7	-
MW	mw_1	148000	Da
$t_{1/2}$	el_half_1	20	days
$t_{1/2,\mathrm{a}}$	abs_half	2.5	days
BW	BW	70	kg
V	volume_central	5	L
$\mathrm{Valency}$	drug_valency_1	1	-
$t_{1/2,\mathrm{L}}$	lig_half_1	30	min
$t_{1/2,\mathrm{R}}$	rec_half_1	540	min
$t_{1/2,\mathrm{sR}}$	shed_half_1	0.5	hr
$K_{D,\mathrm{L:R}}$	lig_rec_kd_1	0.019	nM
$C_{SS,\mathrm{L}}$	lig_css_1_central	0.0000575	nM
$C_{SS,\mathrm{R}}$	rec_css_1_central	0.23	nM
$C_{SS,\mathrm{sR}}$	shed_css_1_central	0	nM
$\mathrm{Density}_{\mathrm{cells}}$	cell_density_mL_central	1000000	#/mL

Parameter ID	Value
Scan Parameter 1	Dose
Lower Limit 1	1
Upper Limit 1	300
N1	11
Scale 1	log

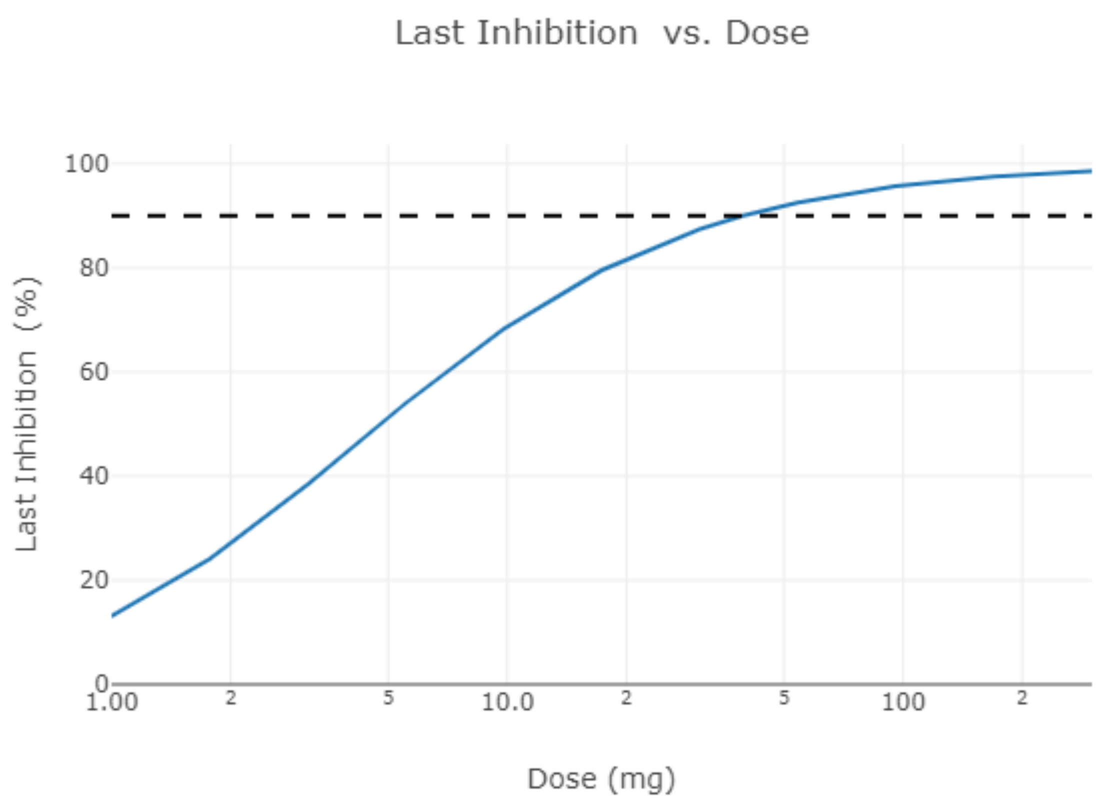
Last Inhibition vs. Dose
Last Inhibition (%)
100
80
60
40
20
0
1.00
2
5
10.0
2
5
100
2
Dose (mg)

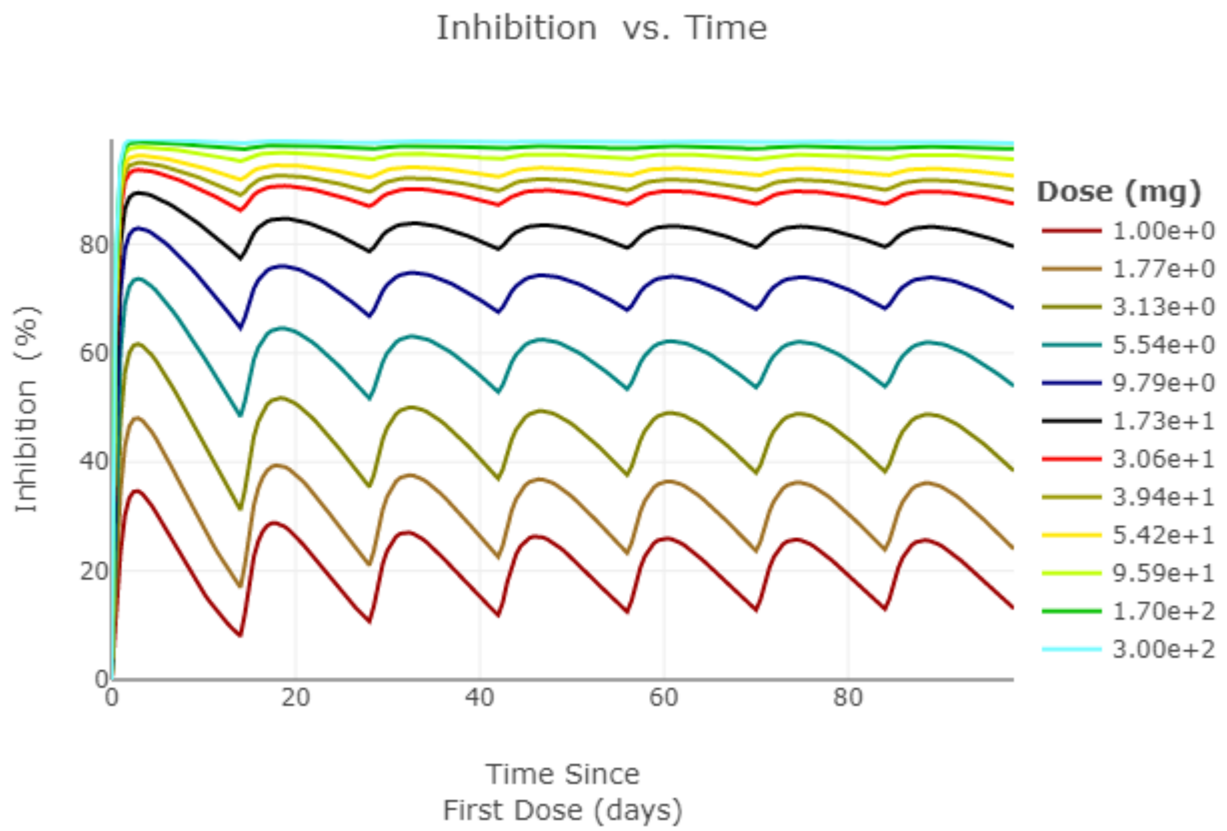
Inhibition vs. Time
Inhibition (%)
80
60
40
20
0
0
20
40
60
80
Time Since
First Dose (days)
Dose (mg)
1.00e+0
1.77e+0
3.13e+0
5.54e+0
9.79e+0
1.73e+1
3.06e+1
3.94e+1
5.42e+1
9.59e+1
1.70e+2
3.00e+2

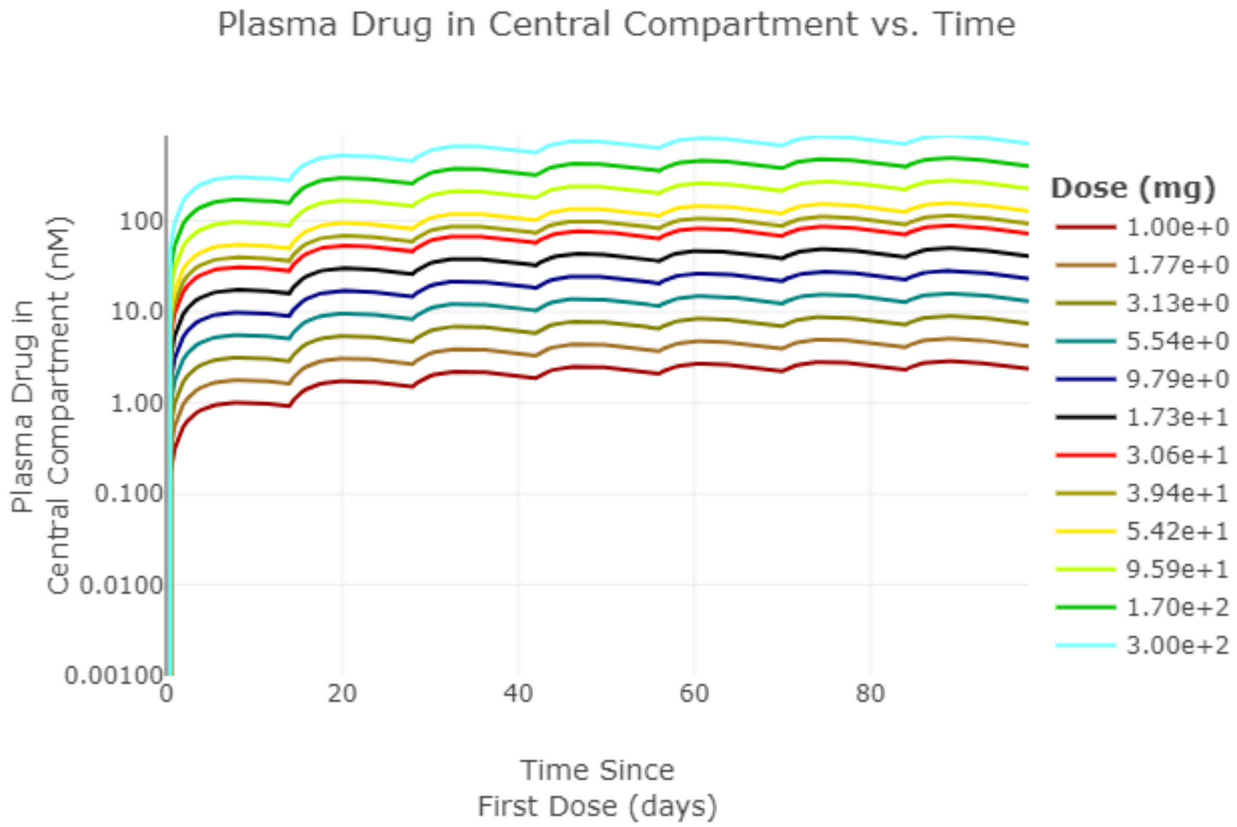

2.1) Scenario: abs_half_high

One dimensional scan over parameter, Dose, between 1 and 300 using log spacing.

2.1.1) Model Parameters

Table 2.1.1

Symbol	Parameter ID	Value	Unit
τ	interval	14	-
D	dose	100	mg
$K_{D,\mathrm{L}}$	mab_kd_1	0.008	nM
N_{doses}	dose_count	7	-
MW	mw_1	148000	Da
$t_{1/2}$	el_half_1	20	days
$t_{1/2,\mathrm{a}}$	abs_half	0.83	days
BW	BW	70	kg
V	volume_central	5	L
Valency	drug_valency_1	1	-
$t_{1/2,\mathrm{L}}$	lig_half_1	30	min
$t_{1/2,\mathrm{R}}$	rec_half_1	540	min

 © Applied BioMath LLC 2017-2021

$t_{1/2,\mathrm{sR}}$	shed_half_1	0.5	hr
$K_{D,\mathrm{L:R}}$	lig_rec_kd_1	0.019	nM
$C_{SS,\mathrm{L}}$	lig_css_1_central	0.0000575	nM
$C_{SS,\mathrm{R}}$	rec_css_1_central	0.23	nM
$C_{SS,\mathrm{sR}}$	shed_css_1_central	0	nM
$\mathrm{Density}_{\mathrm{cells}}$	cell_density_mL_central	1000000	#/mL

Parameter ID	Value
Scan Parameter 1	Dose
Lower Limit 1	1
Upper Limit 1	300
N1	11
Scale 1	log

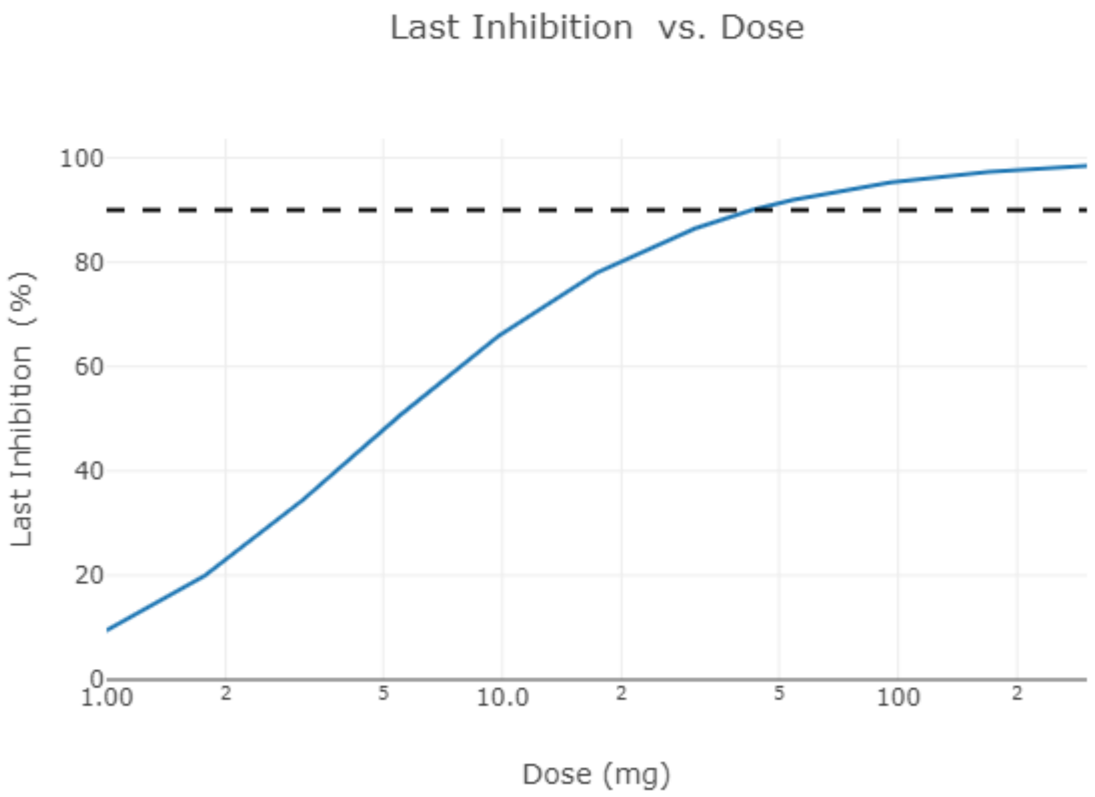

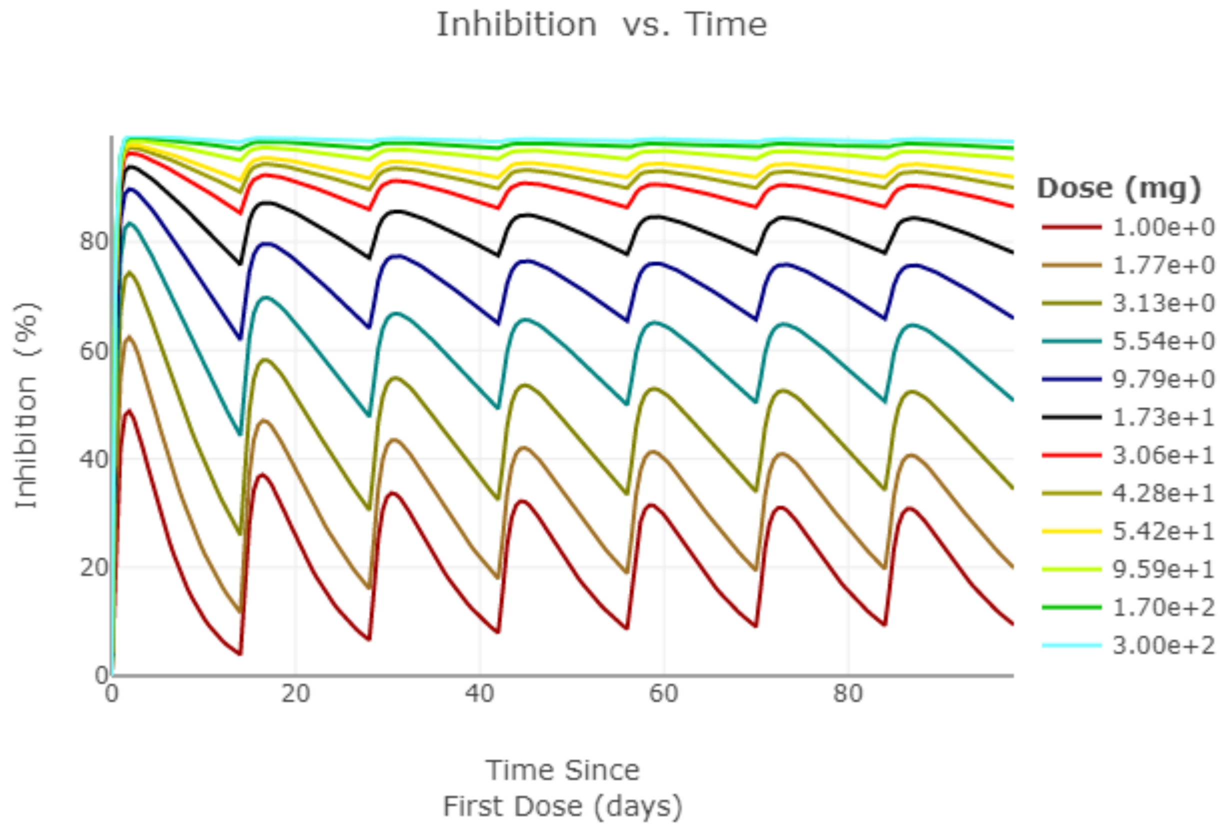

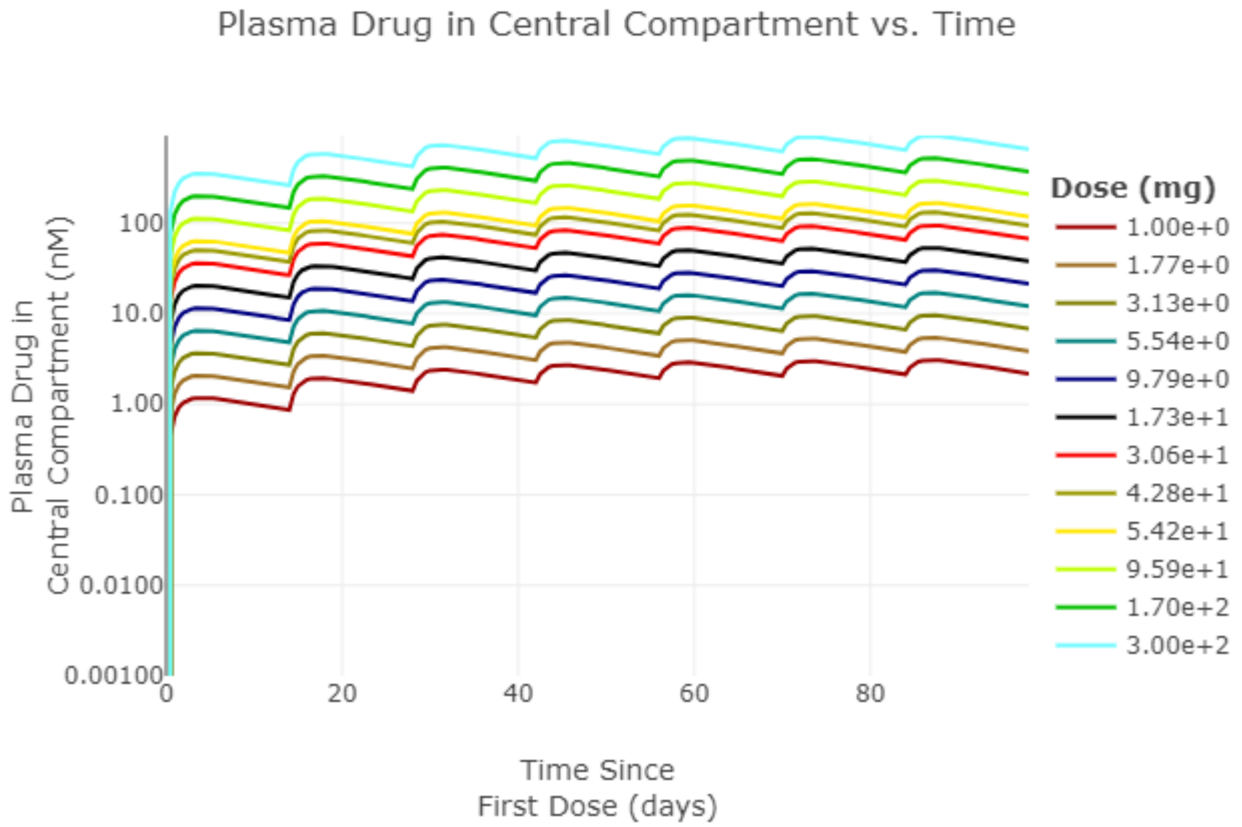

 © Applied BioMath LLC 2017-2021

2.2) Scenario: abs_half_low

One dimensional scan over parameter, Dose, between 1 and 300 using log spacing.

2.2.1) Model Parameters

Table 2.2.1

Symbol	Parameter ID	Value	Unit
τ	interval	14	-
D	dose	100	mg
$K_{D,\mathrm{L}}$	mab_kd_1	0.008	nM
N_{doses}	dose_count	7	-
MW	mw_1	148000	Da
$t_{1/2}$	el_half_1	20	days
$t_{1/2,\mathrm{a}}$	abs_half	7.5	days
BW	BW	70	kg
V	volume_central	5	L
$\mathrm{Valency}$	drug_valency_1	1	-
$t_{1/2,\mathrm{L}}$	lig_half_1	30	min
$t_{1/2,\mathrm{R}}$	rec_half_1	540	min
$t_{1/2,\mathrm{sR}}$	shed_half_1	0.5	hr
$K_{D,\mathrm{L:R}}$	lig_rec_kd_1	0.019	nM
$C_{ss,\mathrm{L}}$	lig_css_1_central	0.0000575	nM
$C_{ss,\mathrm{R}}$	rec_css_1_central	0.23	nM
$C_{ss,\mathrm{sR}}$	shed_css_1_central	0	nM
$\mathrm{Density}_{\mathrm{cells}}$	cell_density_mL_central	1000000	#/mL

Parameter ID	Value
Scan Parameter 1	Dose
Lower Limit 1	1
Upper Limit 1	300
N1	11
Scale 1	log

 © Applied BioMath LLC 2017-2021

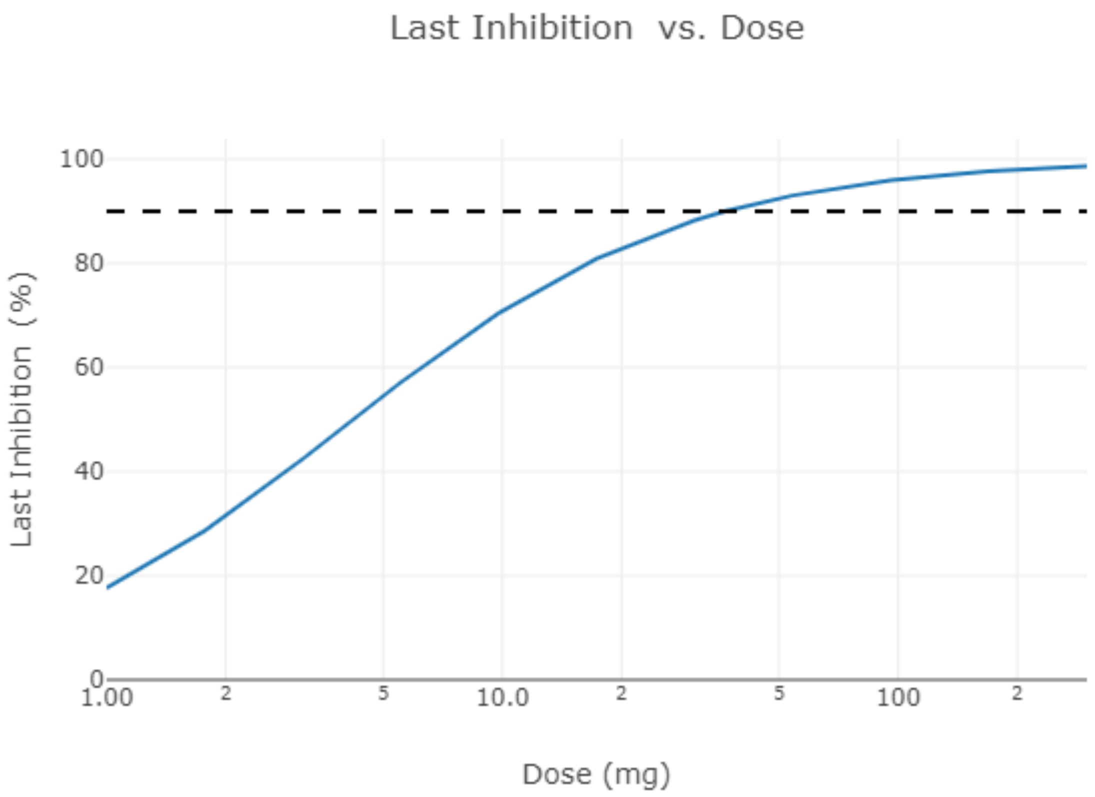
Last Inhibition vs. Dose
Last Inhibition (%)
100
80
60
40
20
0
1.00
2
5
10.0
2
5
100
2
Dose (mg)

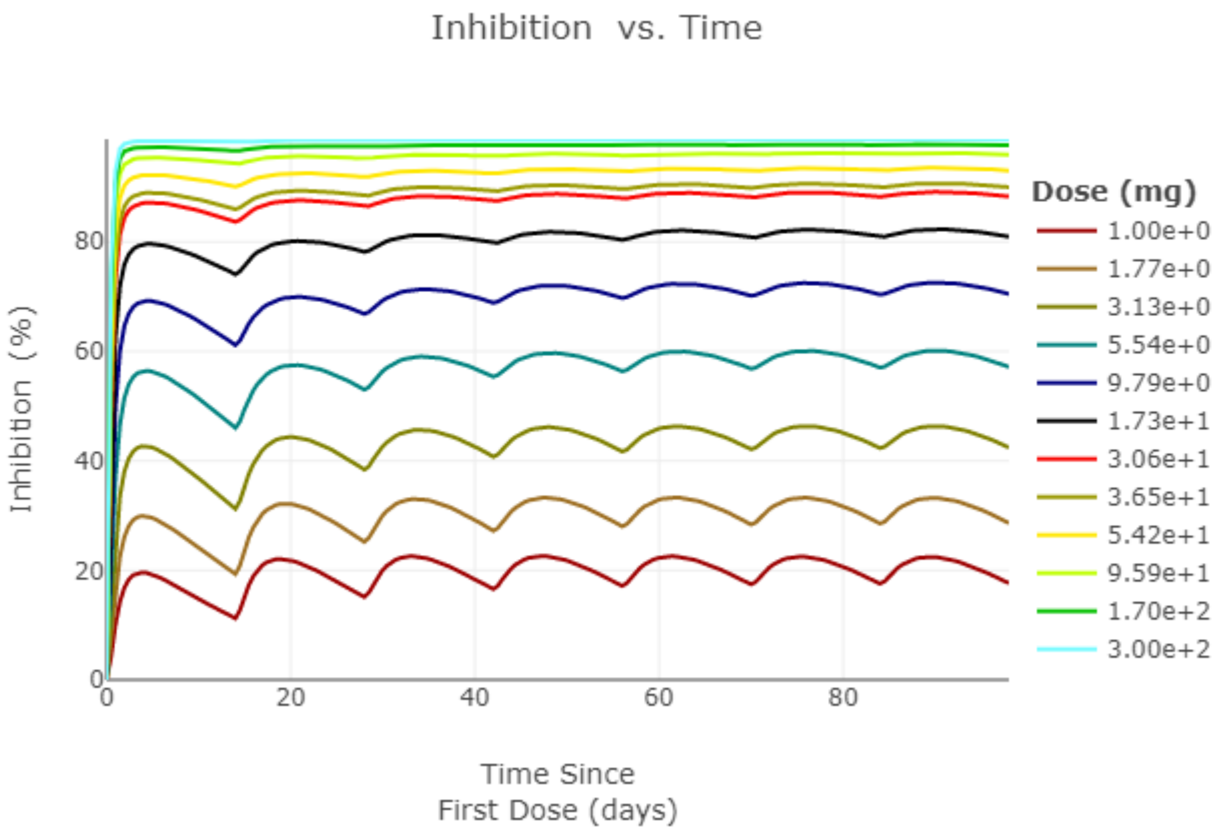
Inhibition vs. Time
Inhibition (%)
80
60
40
20
0
0
20
40
60
80
Time Since
First Dose (days)
Dose (mg)
1.00e+0
1.77e+0
3.13e+0
5.54e+0
9.79e+0
1.73e+1
3.06e+1
3.65e+1
5.42e+1
9.59e+1
1.70e+2
3.00e+2

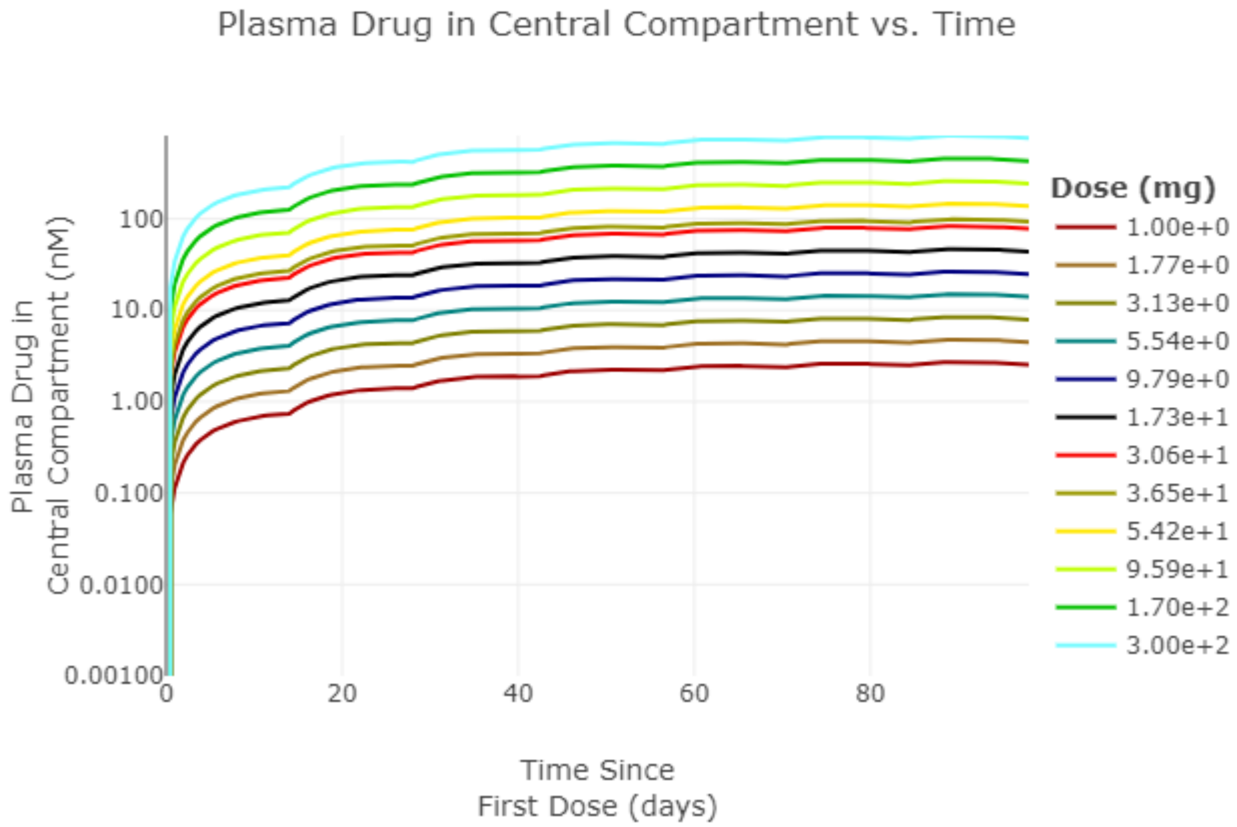

2.3) Scenario: valency_high

One dimensional scan over parameter, Dose, between 1 and 300 using log spacing.

2.3.1) Model Parameters

Table 2.3.1

Symbol	Parameter ID	Value	Unit
τ	interval	14	-
D	dose	100	mg
$K_{D,\mathrm{L}}$	mab_kd_1	0.008	nM
N_{doses}	dose_count	7	-
MW	mw_1	148000	Da
$t_{1/2}$	el_half_1	20	days
$t_{1/2,\mathrm{a}}$	abs_half	2.5	days
BW	BW	70	kg
V	volume_central	5	L
$\mathrm{Valency}$	drug_valency_1	2	-
$t_{1/2,\mathrm{L}}$	lig_half_1	30	min
$t_{1/2,\mathrm{R}}$	rec_half_1	540	min

 © Applied BioMath LLC 2017-2021

$t_{1/2,\mathrm{sR}}$	shed_half_1	0.5	hr
$K_{D,\mathrm{L:R}}$	lig_rec_kd_1	0.019	nM
$C_{SS,\mathrm{L}}$	lig_css_1_central	0.0000575	nM
$C_{SS,\mathrm{R}}$	rec_css_1_central	0.23	nM
$C_{SS,\mathrm{sR}}$	shed_css_1_central	0	nM
$\mathrm{Density}_{\mathrm{cells}}$	cell_density_mL_central	1000000	#/mL

Parameter ID	Value
Scan Parameter 1	Dose
Lower Limit 1	1
Upper Limit 1	300
N1	11
Scale 1	log

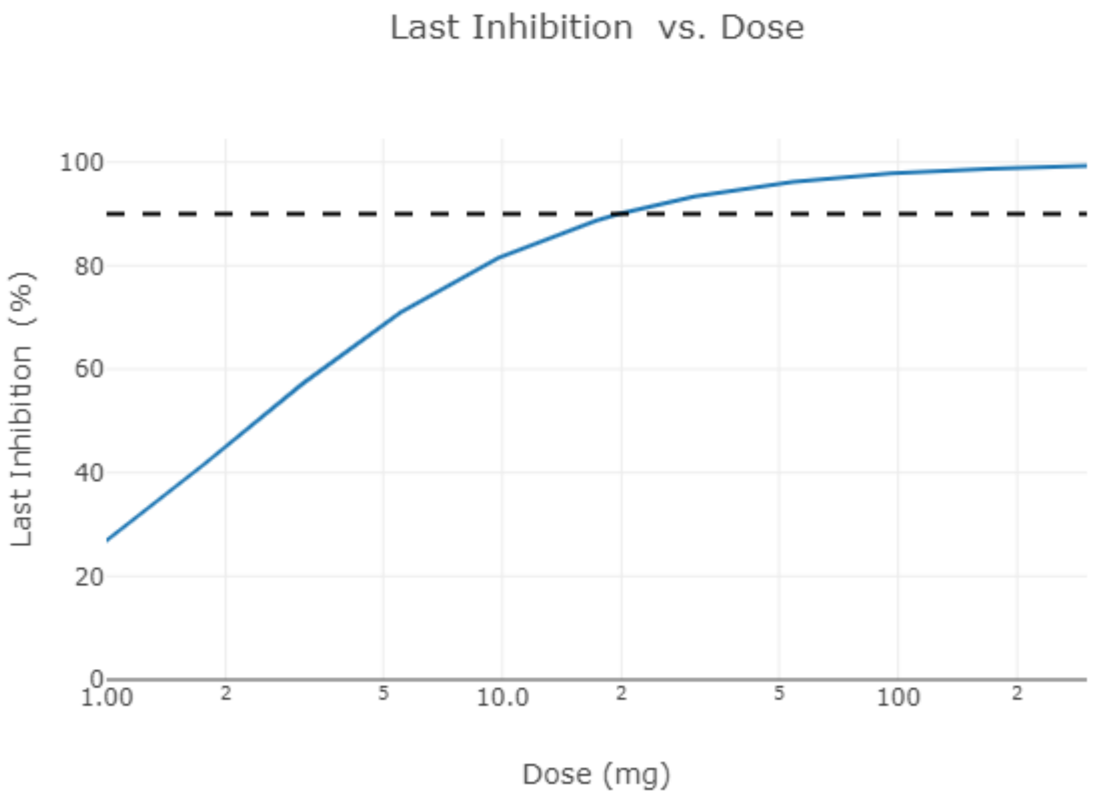

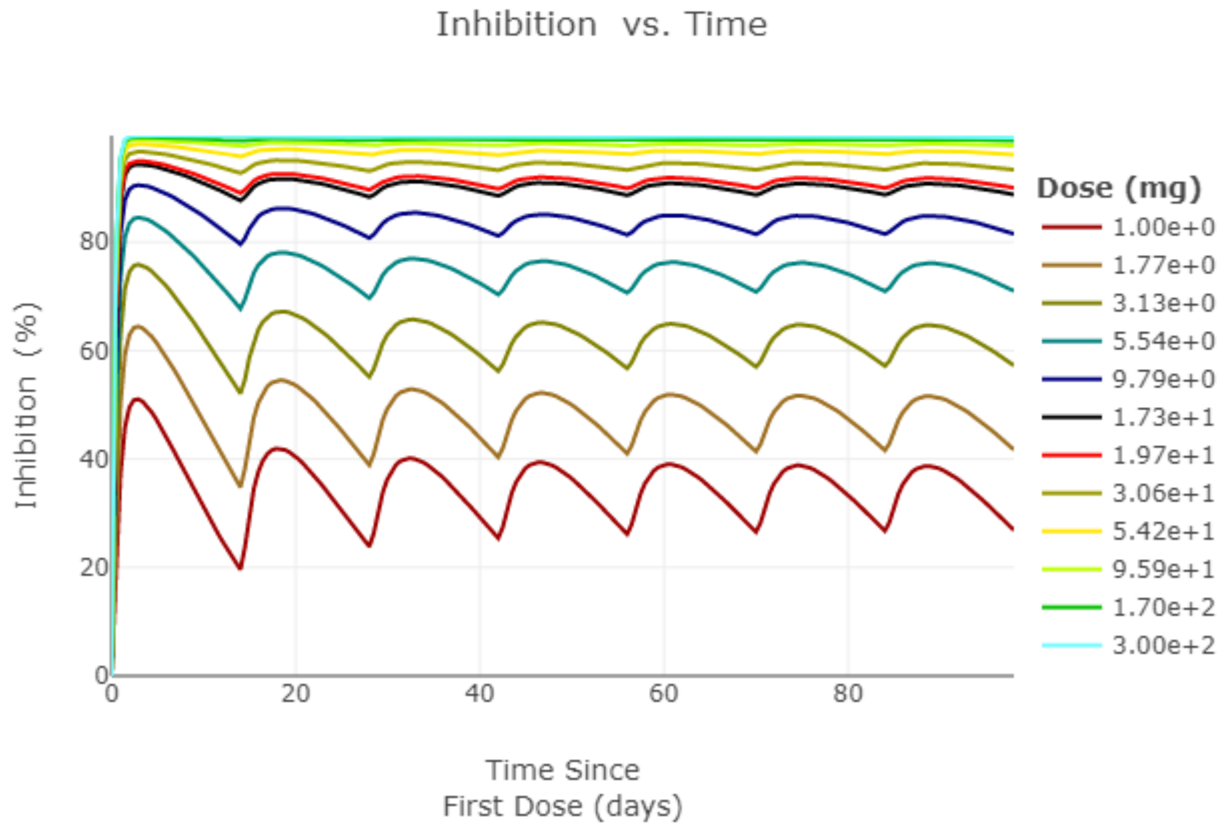

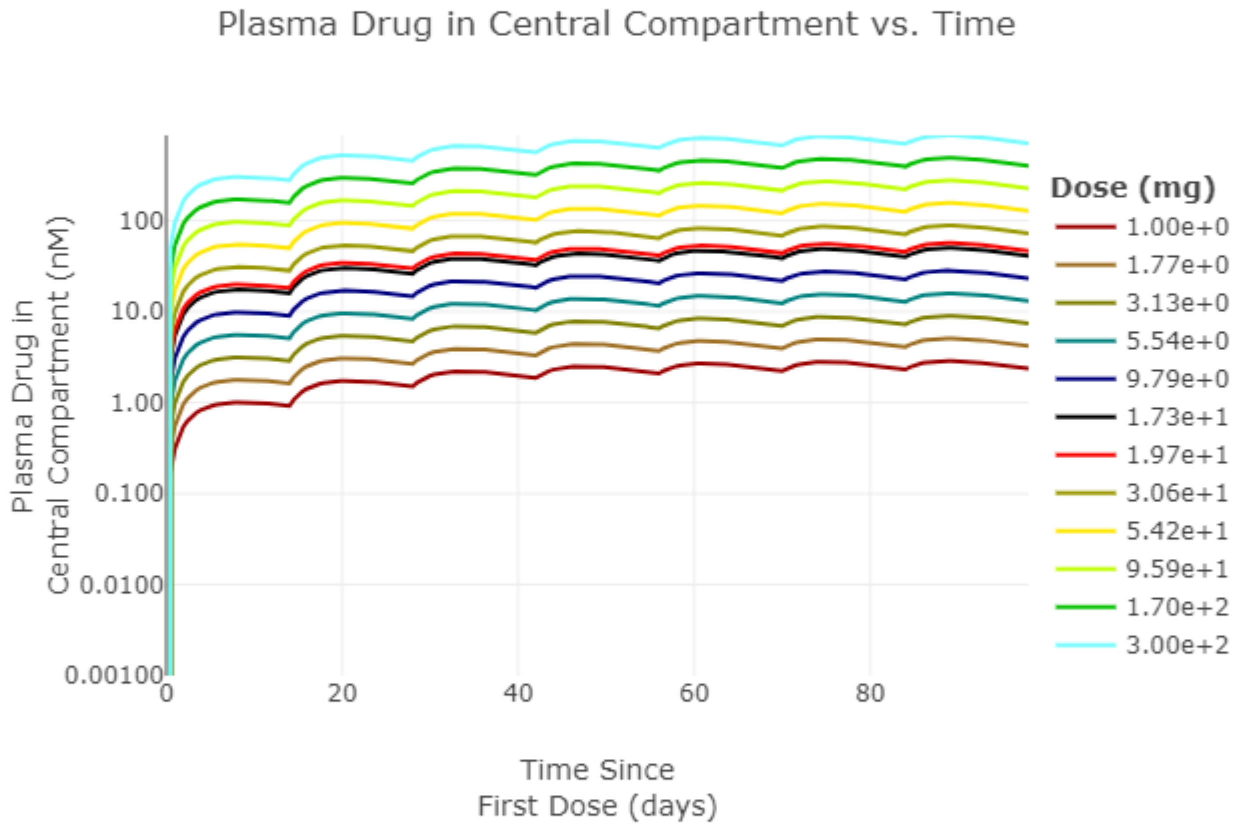

 © Applied BioMath LLC 2017-2021

2.4) Scenario: drug halflife_high

One dimensional scan over parameter, Dose, between 1 and 300 using log spacing.

2.4.1) Model Parameters

Table 2.4.1

Symbol	Parameter ID	Value	Unit
τ	interval	14	-
D	dose	100	mg
$K_{D,\mathrm{L}}$	mab_kd_1	0.008	nM
N_{doses}	dose_count	7	-
MW	mw_1	148000	Da
$t_{1/2}$	el_half_1	60	days
$t_{1/2,\mathrm{a}}$	abs_half	2.5	days
BW	BW	70	kg
V	volume_central	5	L
$\mathrm{Valency}$	drug_valency_1	1	-
$t_{1/2,\mathrm{L}}$	lig_half_1	30	min
$t_{1/2,\mathrm{R}}$	rec_half_1	540	min
$t_{1/2,\mathrm{sR}}$	shed_half_1	0.5	hr
$K_{D,\mathrm{L:R}}$	lig_rec_kd_1	0.019	nM
$C_{ss,\mathrm{L}}$	lig_css_1_central	0.0000575	nM
$C_{ss,\mathrm{R}}$	rec_css_1_central	0.23	nM
$C_{ss,\mathrm{sR}}$	shed_css_1_central	0	nM
$\mathrm{Density}_{\mathrm{cells}}$	cell_density_mL_central	1000000	#/mL

Parameter ID	Value
Scan Parameter 1	Dose
Lower Limit 1	1
Upper Limit 1	300
N1	11
Scale 1	log

 © Applied BioMath LLC 2017-2021

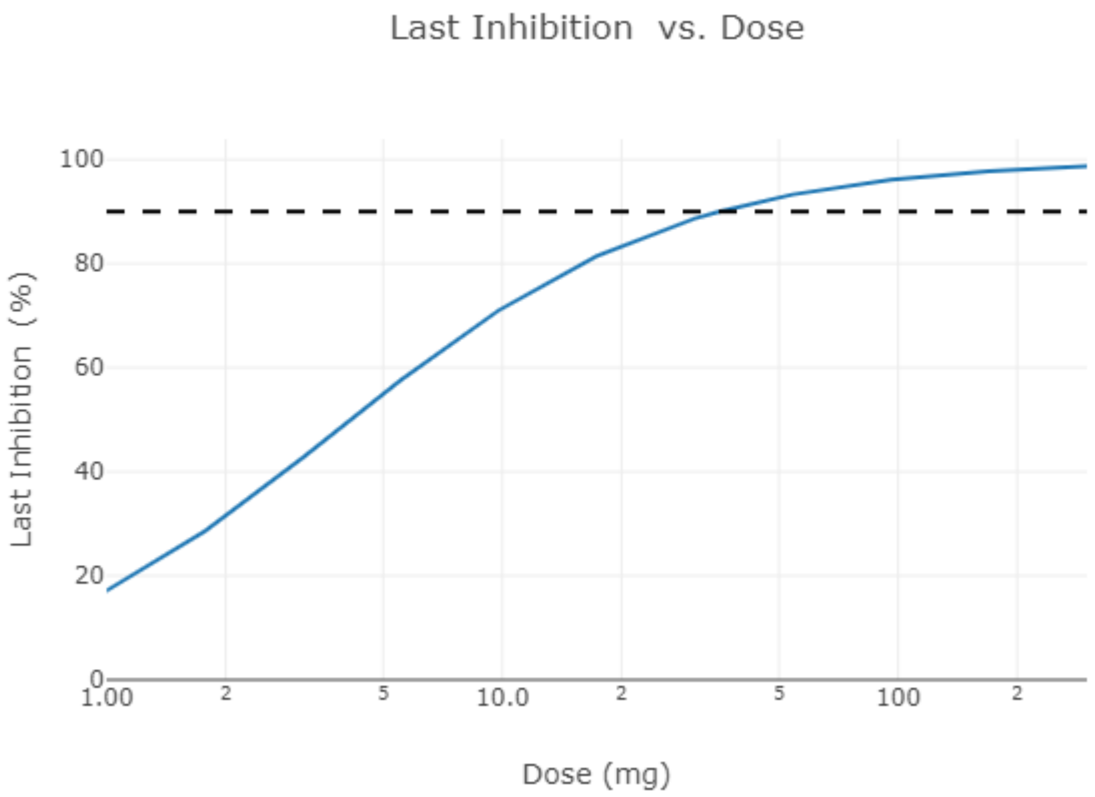
Last Inhibition vs. Dose
Last Inhibition (%)
100
80
60
40
20
0
1.00
2
5
10.0
2
5
100
2
Dose (mg)

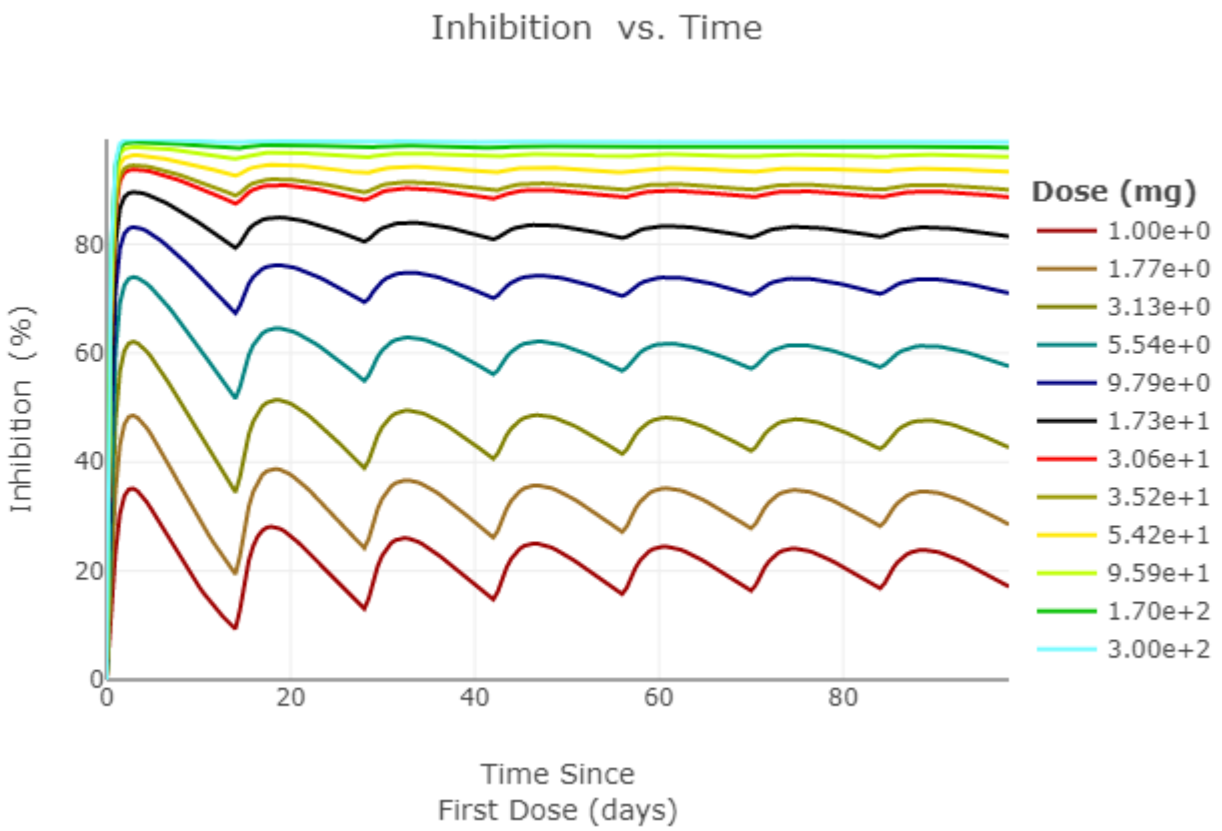
Inhibition vs. Time
Inhibition (%)
80
60
40
20
0
0
20
40
60
80
Time Since
First Dose (days)
Dose (mg)
1.00e+0
1.77e+0
3.13e+0
5.54e+0
9.79e+0
1.73e+1
3.06e+1
3.52e+1
5.42e+1
9.59e+1
1.70e+2
3.00e+2

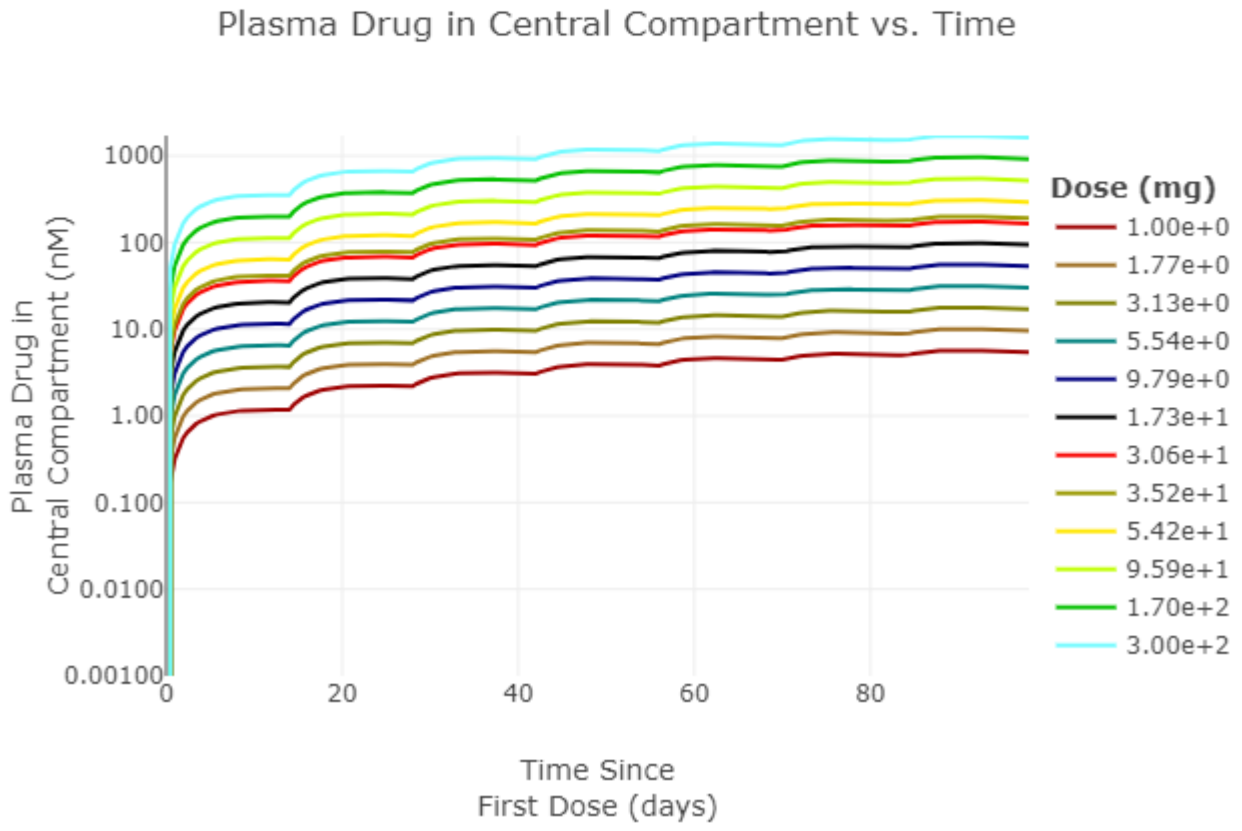

2.5) Scenario: drug halflife_low

One dimensional scan over parameter, Dose, between 1 and 300 using log spacing.

2.5.1) Model Parameters

Table 2.5.1

Symbol	Parameter ID	Value	Unit
τ	interval	14	-
D	dose	100	mg
$K_{D,\mathrm{L}}$	mab_kd_1	0.008	nM
N_{doses}	dose_count	7	-
MW	mw_1	148000	Da
$t_{1/2}$	el_half_1	6.67	days
$t_{1/2,\mathrm{a}}$	abs_half	2.5	days
BW	BW	70	kg
V	volume_central	5	L
Valency	drug_valency_1	1	-
$t_{1/2,\mathrm{L}}$	lig_half_1	30	min
$t_{1/2,\mathrm{R}}$	rec_half_1	540	min

 © Applied BioMath LLC 2017-2021

$t_{1/2,\mathrm{sR}}$	shed_half_1	0.5	hr
$K_{D,\mathrm{L:R}}$	lig_rec_kd_1	0.019	nM
$C_{SS,\mathrm{L}}$	lig_css_1_central	0.0000575	nM
$C_{SS,\mathrm{R}}$	rec_css_1_central	0.23	nM
$C_{SS,\mathrm{sR}}$	shed_css_1_central	0	nM
$\mathrm{Density}_{\mathrm{cells}}$	cell_density_mL_central	1000000	#/mL

Parameter ID	Value
Scan Parameter 1	Dose
Lower Limit 1	1
Upper Limit 1	300
N1	11
Scale 1	log

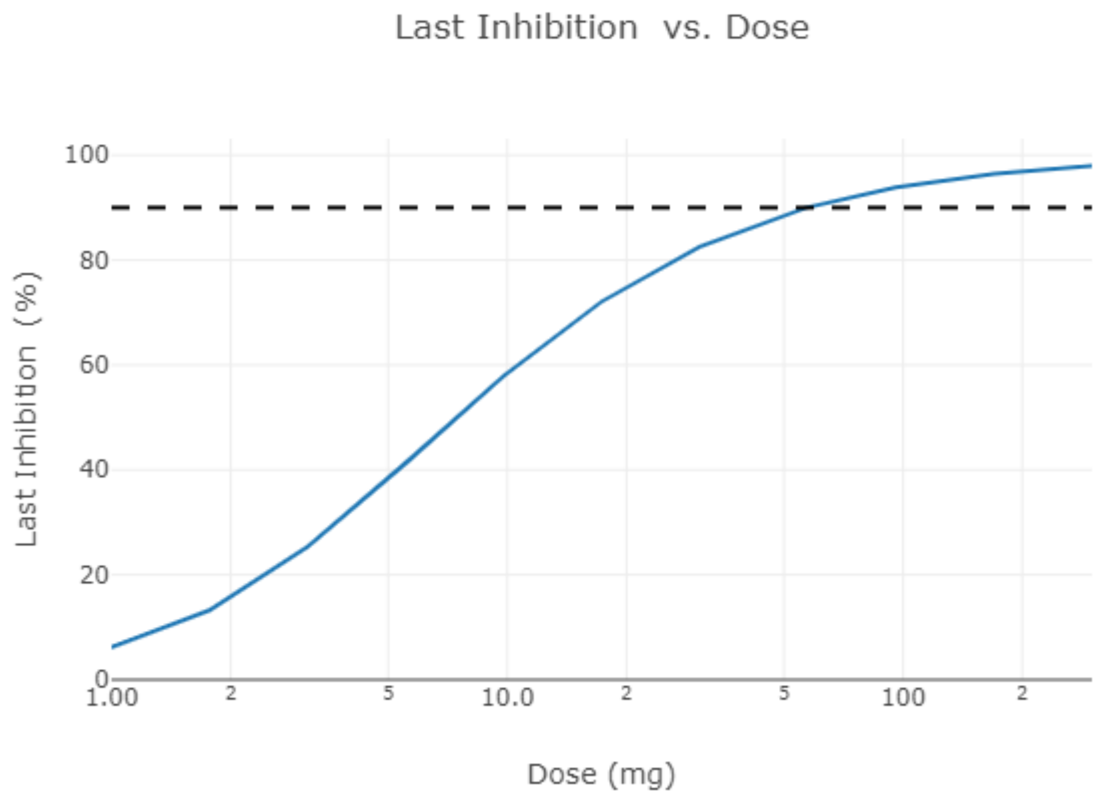

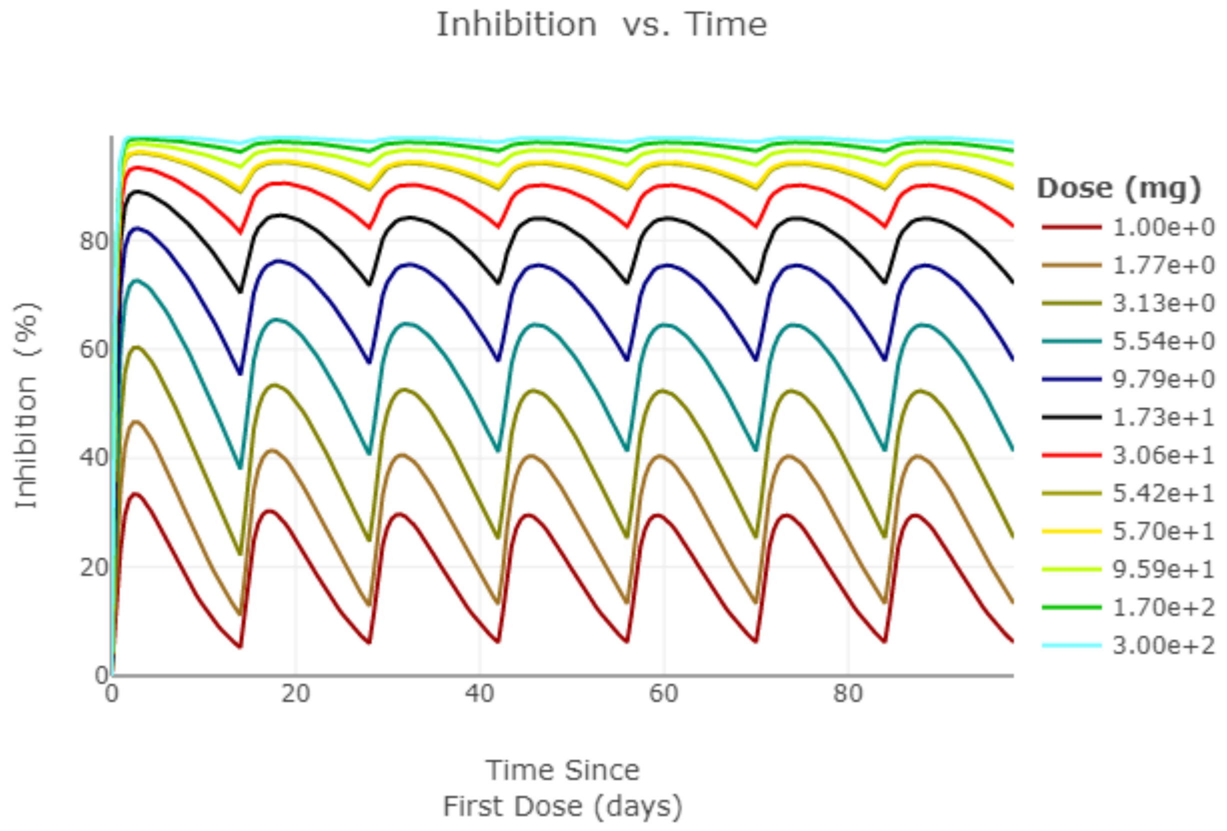

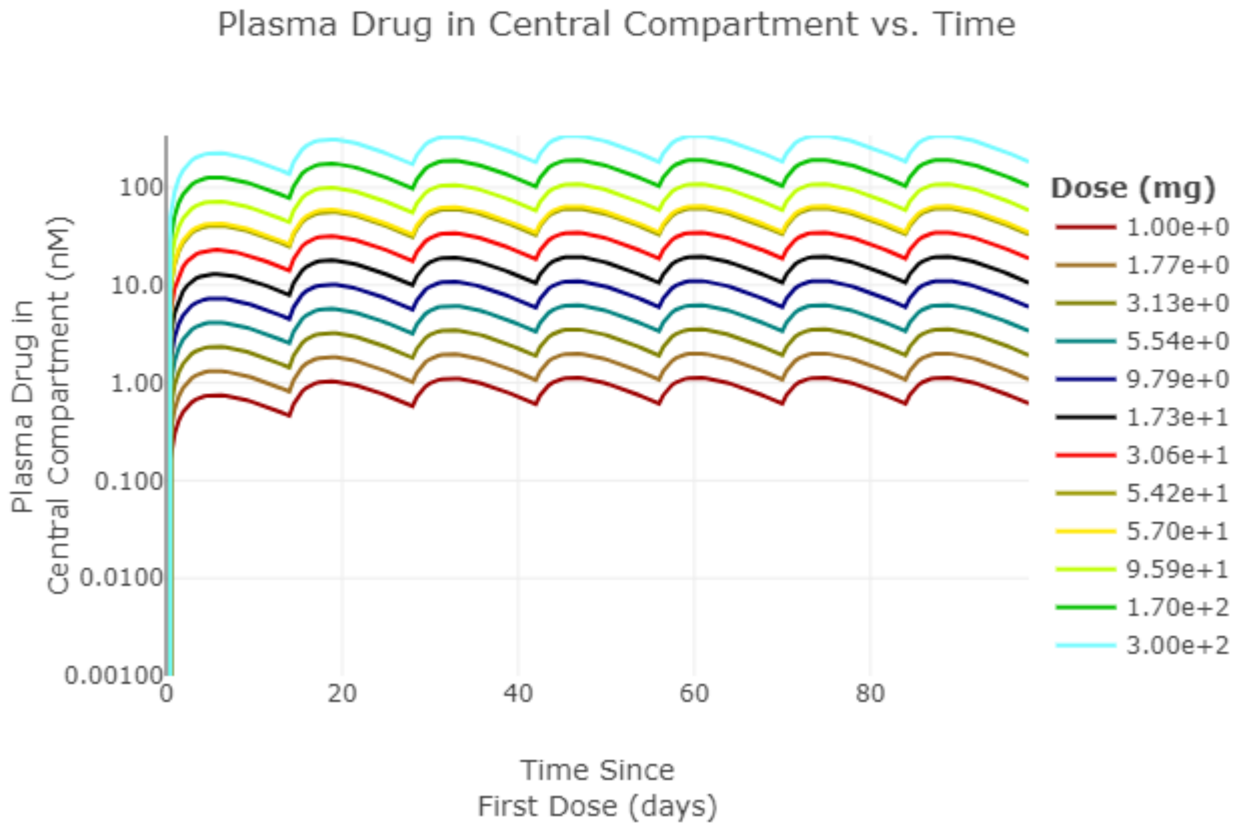

 © Applied BioMath LLC 2017-2021

2.6) Scenario: TNF_conc_high

One dimensional scan over parameter, Dose, between 1 and 300 using log spacing.

2.6.1) Model Parameters

Table 2.6.1

Symbol	Parameter ID	Value	Unit
τ	interval	14	-
D	dose	100	mg
$K_{D,\mathrm{L}}$	mab_kd_1	0.008	nM
N_{doses}	dose_count	7	-
MW	mw_1	148000	Da
$t_{1/2}$	el_half_1	20	days
$t_{1/2,\mathrm{a}}$	abs_half	2.5	days
BW	BW	70	kg
V	volume_central	5	L
$\mathrm{Valency}$	drug_valency_1	1	-
$t_{1/2,\mathrm{L}}$	lig_half_1	30	min
$t_{1/2,\mathrm{R}}$	rec_half_1	540	min
$t_{1/2,\mathrm{sR}}$	shed_half_1	0.5	hr
$K_{D,\mathrm{L:R}}$	lig_rec_kd_1	0.019	nM
$C_{ss,\mathrm{L}}$	lig_css_1_central	0.000173	nM
$C_{ss,\mathrm{R}}$	rec_css_1_central	0.23	nM
$C_{ss,\mathrm{sR}}$	shed_css_1_central	0	nM
$\mathrm{Density}_{\mathrm{cells}}$	cell_density_mL_central	1000000	#/mL

Parameter ID	Value
Scan Parameter 1	Dose
Lower Limit 1	1
Upper Limit 1	300
N1	11
Scale 1	log

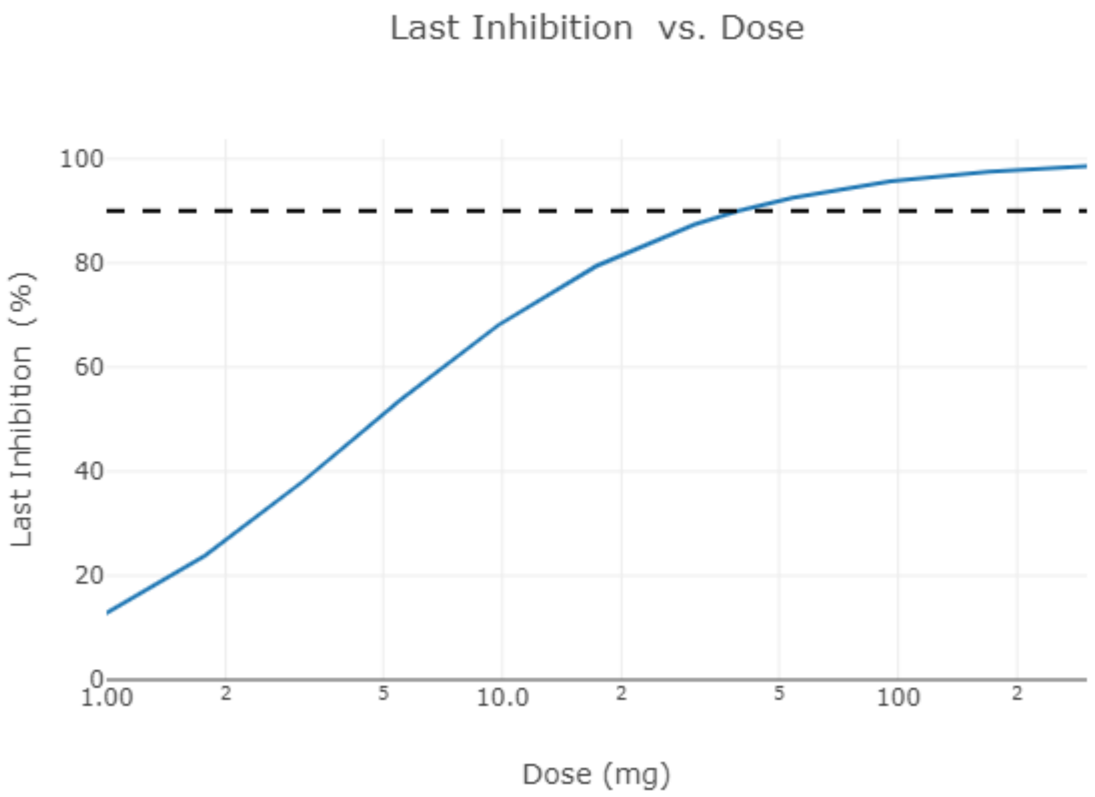
Last Inhibition vs. Dose
Last Inhibition (%)
100
80
60
40
20
0
1.00
2
5
10.0
2
5
100
2
Dose (mg)

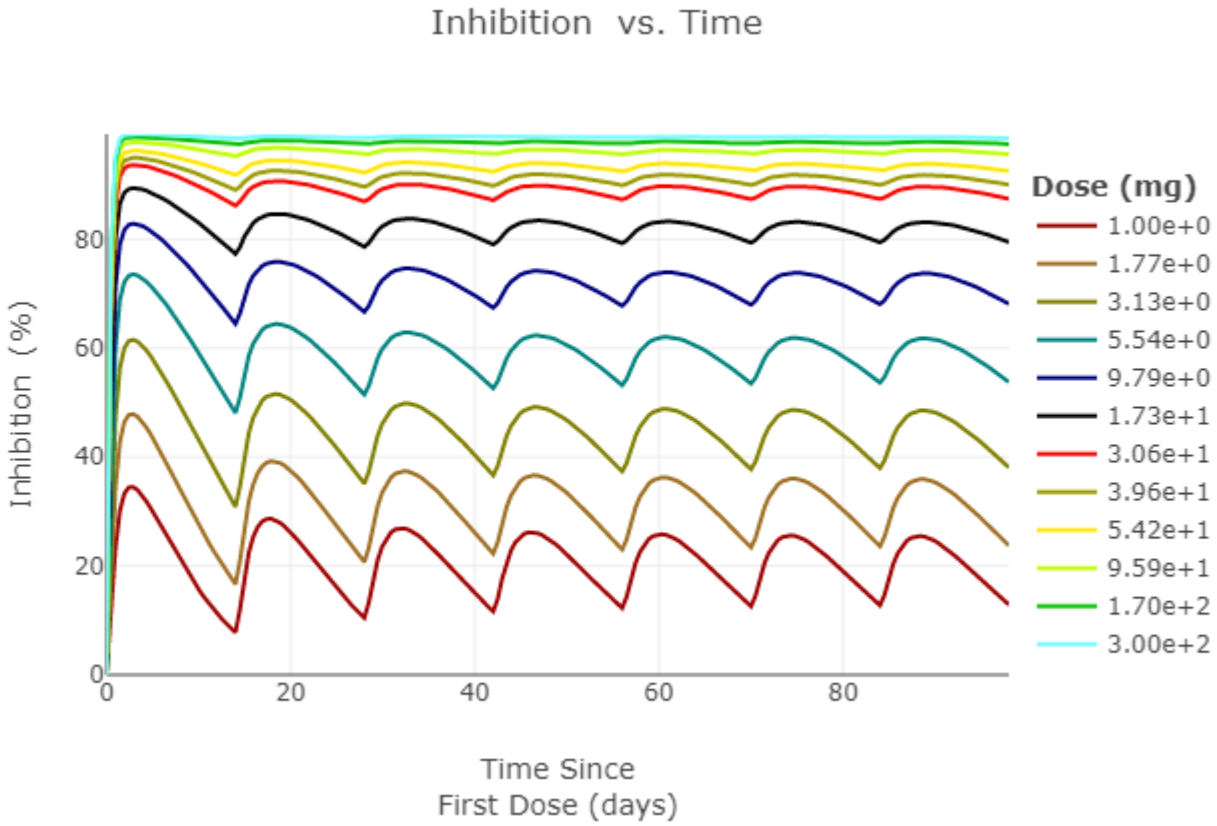
Inhibition vs. Time
Inhibition (%)
80
60
40
20
0
0
20
40
60
80
Time Since
First Dose (days)
Dose (mg)
1.00e+0
1.77e+0
3.13e+0
5.54e+0
9.79e+0
1.73e+1
3.06e+1
3.96e+1
5.42e+1
9.59e+1
1.70e+2
3.00e+2

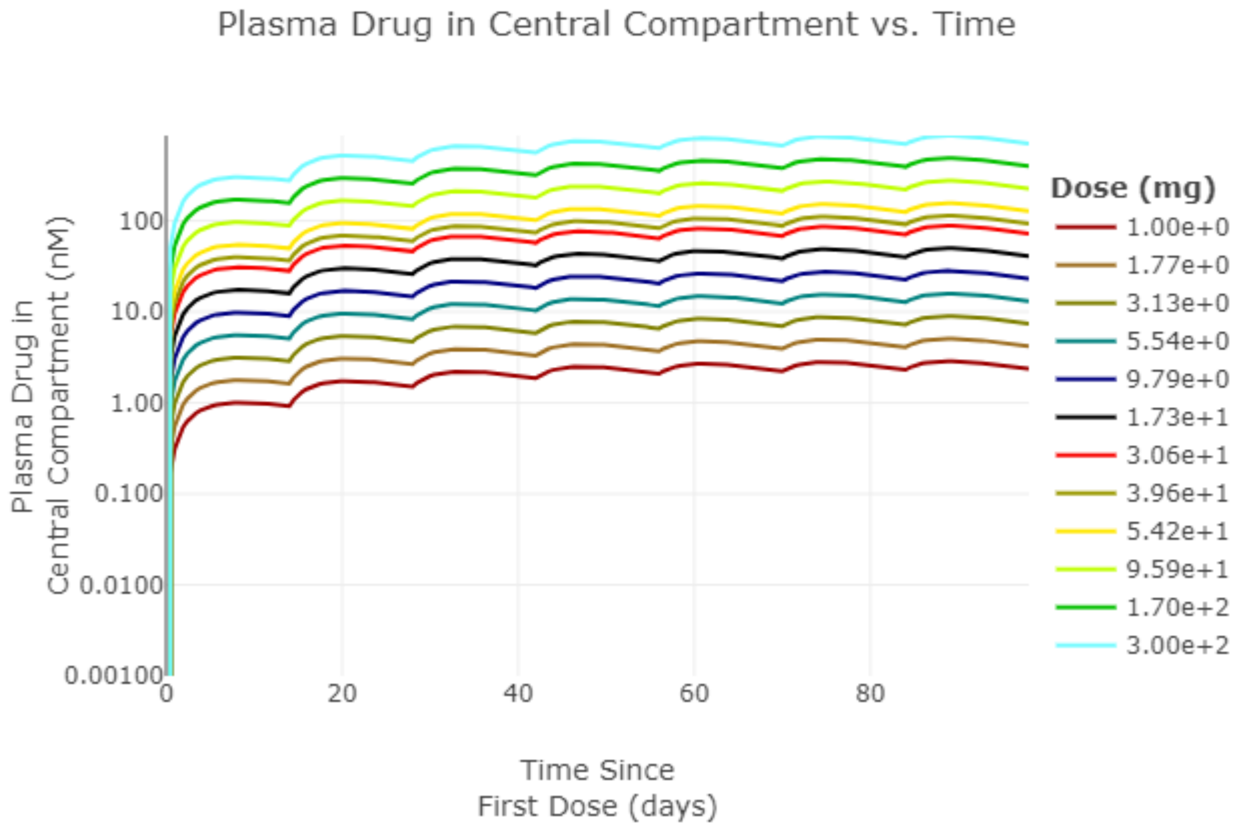

2.7) Scenario: TNF_conc_low

One dimensional scan over parameter, Dose, between 1 and 300 using log spacing.

2.7.1) Model Parameters

Table 2.7.1

Symbol	Parameter ID	Value	Unit
τ	interval	14	-
D	dose	100	mg
$K_{D,\mathrm{L}}$	mab_kd_1	0.008	nM
N_{doses}	dose_count	7	-
MW	mw_1	148000	Da
$t_{1/2}$	el_half_1	20	days
$t_{1/2,\mathrm{a}}$	abs_half	2.5	days
BW	BW	70	kg
V	volume_central	5	L
Valency	drug_valency_1	1	-
$t_{1/2,\mathrm{L}}$	lig_half_1	30	min
$t_{1/2,\mathrm{R}}$	rec_half_1	540	min

 © Applied BioMath LLC 2017-2021

$t_{1/2,\mathrm{sR}}$	shed_half_1	0.5	hr
$K_{D,\mathrm{L:R}}$	lig_rec_kd_1	0.019	nM
$C_{SS,\mathrm{L}}$	lig_css_1_central	0.0000192	nM
$C_{SS,\mathrm{R}}$	rec_css_1_central	0.23	nM
$C_{SS,\mathrm{sR}}$	shed_css_1_central	0	nM
$\mathrm{Density}_{\mathrm{cells}}$	cell_density_mL_central	1000000	#/mL

Parameter ID	Value
Scan Parameter 1	Dose
Lower Limit 1	1
Upper Limit 1	300
N1	11
Scale 1	log

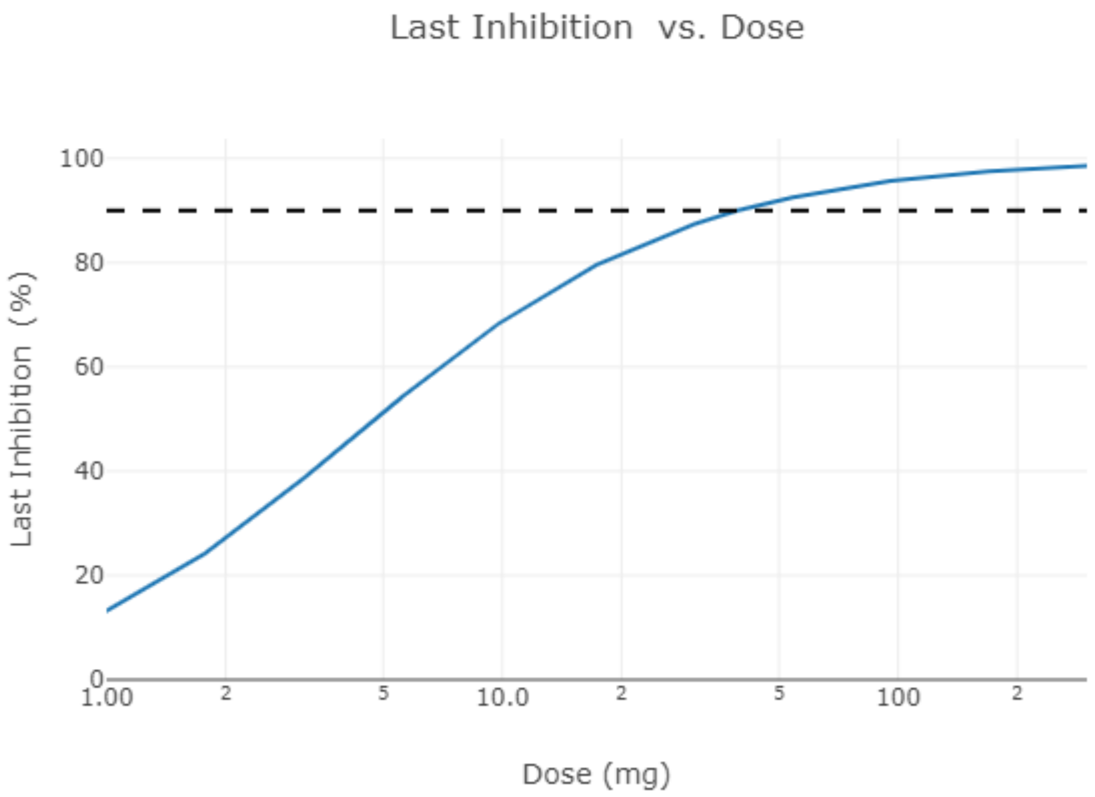

Inhibition vs. Time

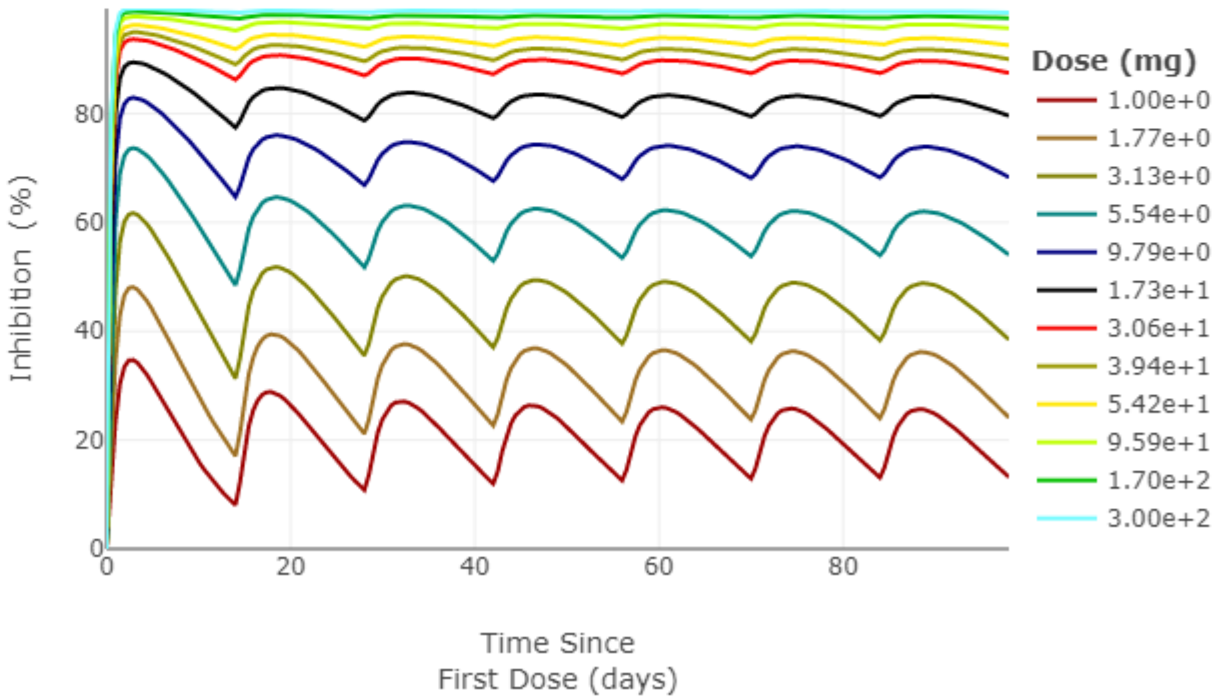

Plasma Drug in Central Compartment vs. Time

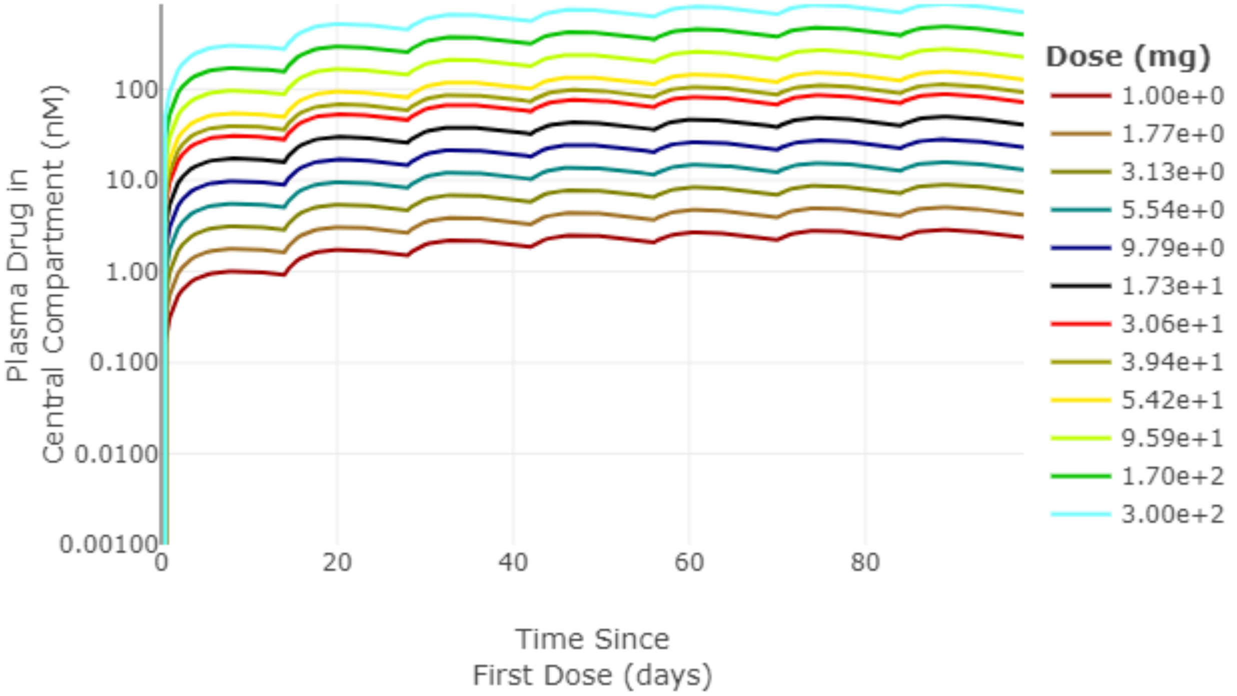

2.8) Scenario: ligand_halflife_high

One dimensional scan over parameter, Dose, between 1 and 300 using log spacing.

2.8.1) Model Parameters

Table 2.8.1

Symbol	Parameter ID	Value	Unit
τ	interval	14	-
D	dose	100	mg
$K_{D,\mathrm{L}}$	mab_kd_1	0.008	nM
N_{doses}	dose_count	7	-
MW	mw_1	148000	Da
$t_{1/2}$	el_half_1	20	days
$t_{1/2,\mathrm{a}}$	abs_half	2.5	days
BW	BW	70	kg
V	volume_central	5	L
$\mathrm{Valency}$	drug_valency_1	1	-
$t_{1/2,\mathrm{L}}$	lig_half_1	90	min
$t_{1/2,\mathrm{R}}$	rec_half_1	540	min
$t_{1/2,\mathrm{sR}}$	shed_half_1	0.5	hr
$K_{D,\mathrm{L:R}}$	lig_rec_kd_1	0.019	nM
$C_{ss,\mathrm{L}}$	lig_css_1_central	0.0000575	nM
$C_{ss,\mathrm{R}}$	rec_css_1_central	0.23	nM
$C_{ss,\mathrm{sR}}$	shed_css_1_central	0	nM
$\mathrm{Density}_{\mathrm{cells}}$	cell_density_mL_central	1000000	#/mL

Parameter ID	Value
Scan Parameter 1	Dose
Lower Limit 1	1
Upper Limit 1	300
N1	11
Scale 1	log

 © Applied BioMath LLC 2017-2021

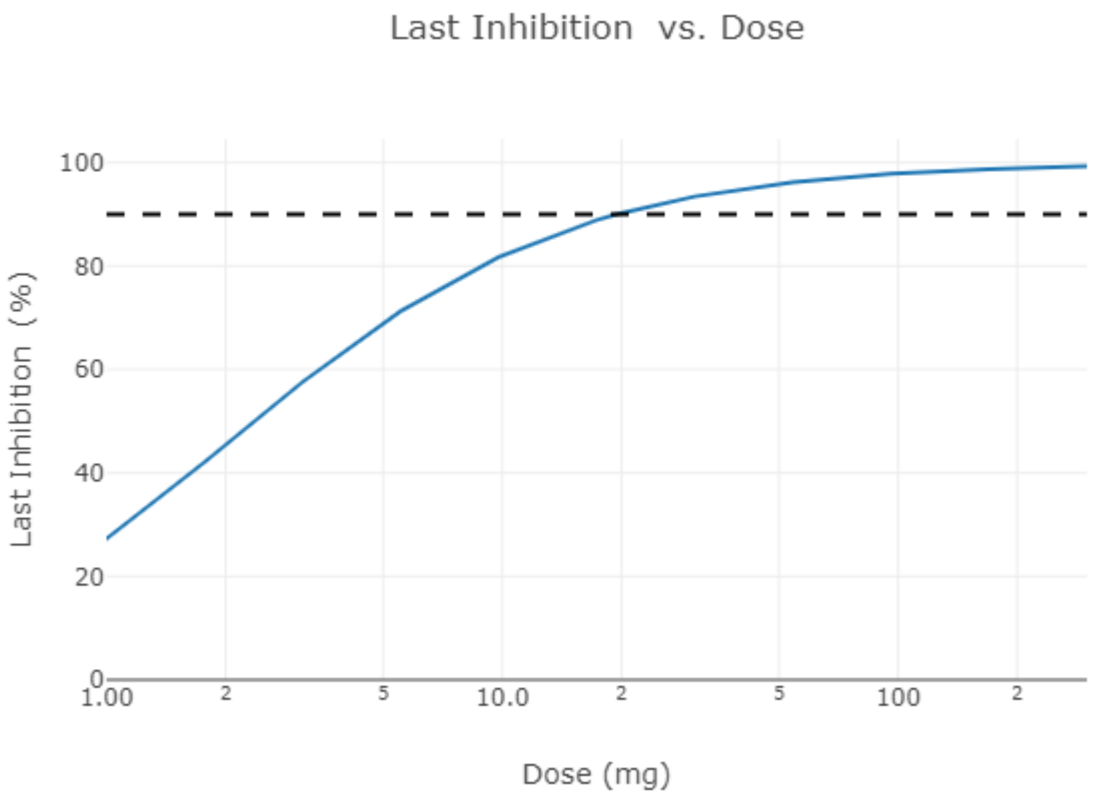
Last Inhibition vs. Dose
100
80
60
40
20
0
Last Inhibition (%)
1.00
2
5
10.0
2
5
100
2
Dose (mg)

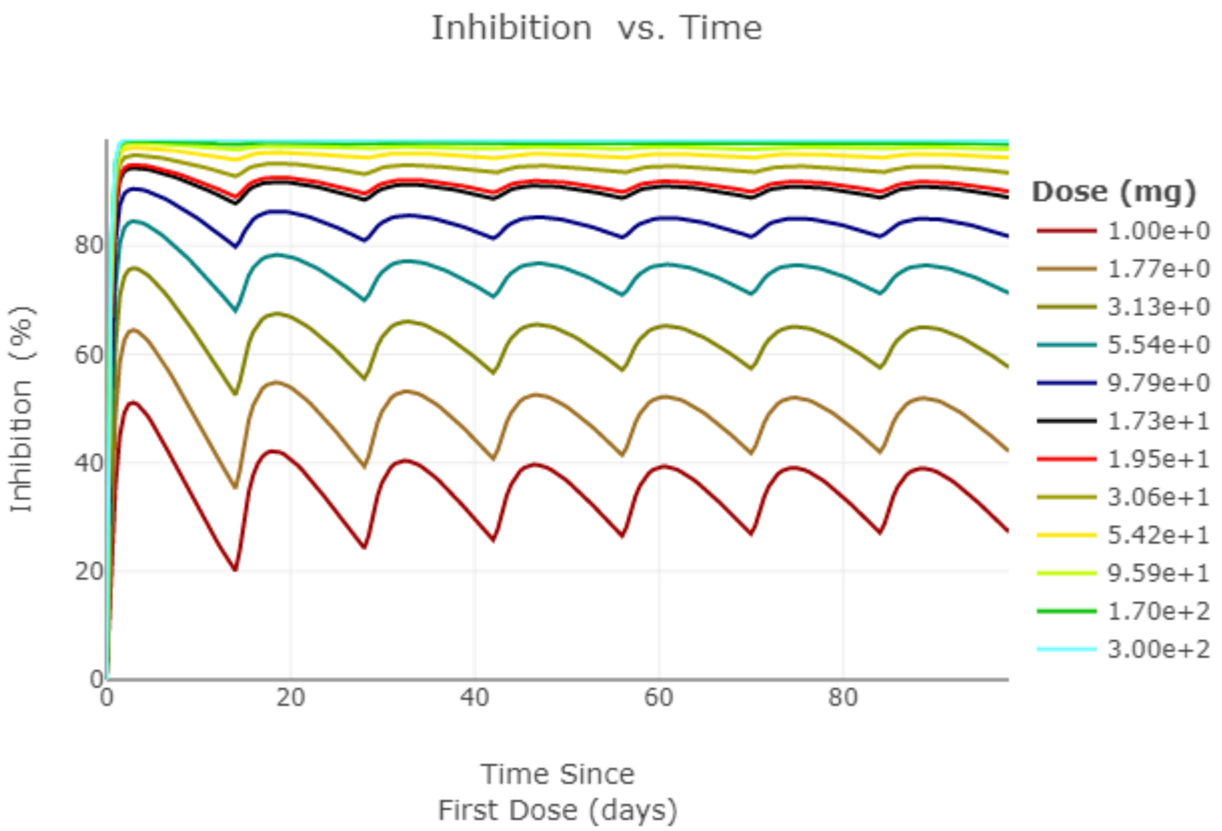
Inhibition vs. Time
80
60
40
20
0
Inhibition (%)
0
20
40
60
80
Time Since
First Dose (days)
Dose (mg)
1.00e+0
1.77e+0
3.13e+0
5.54e+0
9.79e+0
1.73e+1
1.95e+1
3.06e+1
5.42e+1
9.59e+1
1.70e+2
3.00e+2

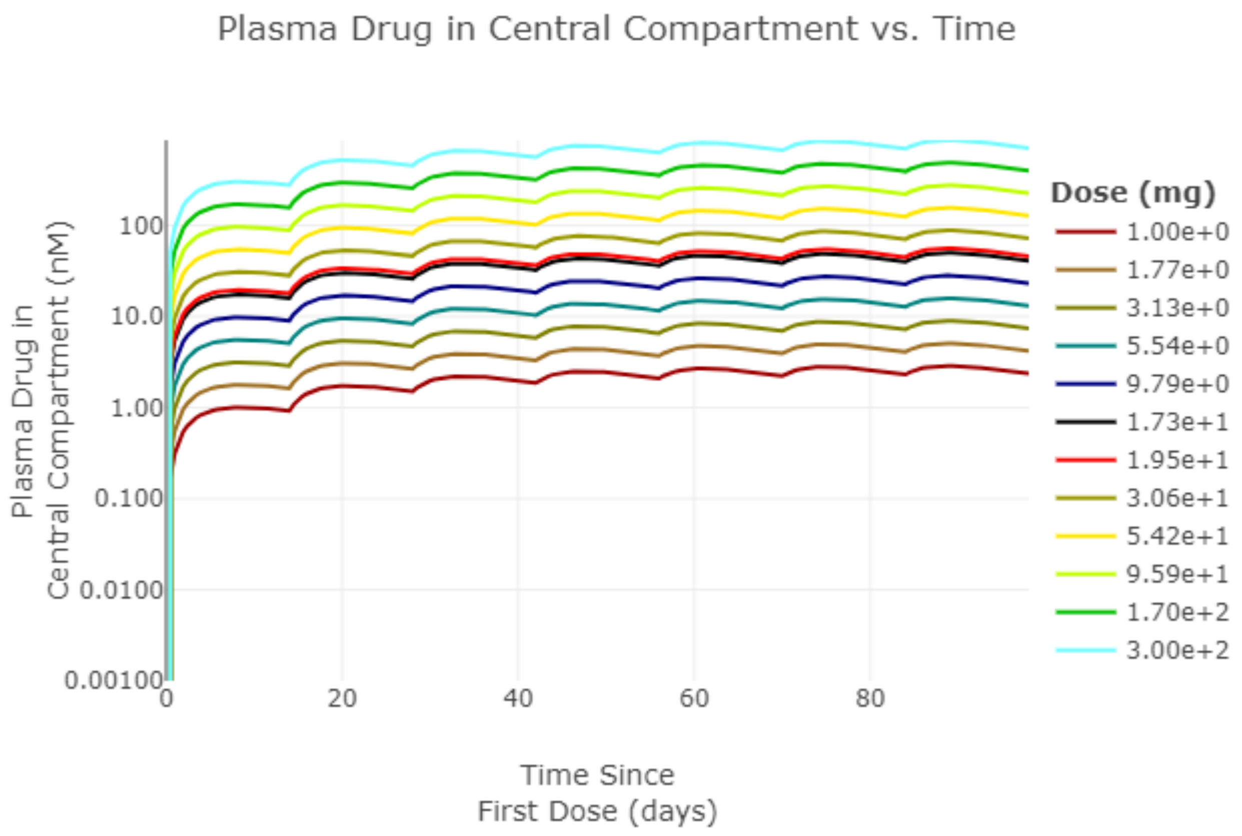

2.9) Scenario: ligand_halflife_low

One dimensional scan over parameter, Dose, between 1 and 300 using log spacing.

2.9.1) Model Parameters

Table 2.9.1

Symbol	Parameter ID	Value	Unit
τ	interval	14	-
D	dose	100	mg
$K_{D,\mathrm{L}}$	mab_kd_1	0.008	nM
N_{doses}	dose_count	7	-
MW	mw_1	148000	Da
$t_{1/2}$	el_half_1	20	days
$t_{1/2,\mathrm{a}}$	abs_half	2.5	days
BW	BW	70	kg
V	volume_central	5	L
Valency	drug_valency_1	1	-
$t_{1/2,\mathrm{L}}$	lig_half_1	10	min
$t_{1/2,\mathrm{R}}$	rec_half_1	540	min

$t_{1/2,\mathrm{sR}}$	shed_half_1	0.5	hr
$K_{D,\mathrm{L:R}}$	lig_rec_kd_1	0.019	nM
$C_{SS,\mathrm{L}}$	lig_css_1_central	0.0000575	nM
$C_{SS,\mathrm{R}}$	rec_css_1_central	0.23	nM
$C_{SS,\mathrm{sR}}$	shed_css_1_central	0	nM
$\mathrm{Density}_{\mathrm{cells}}$	cell_density_mL_central	1000000	#/mL

Parameter ID	Value
Scan Parameter 1	Dose
Lower Limit 1	1
Upper Limit 1	300
N1	11
Scale 1	log

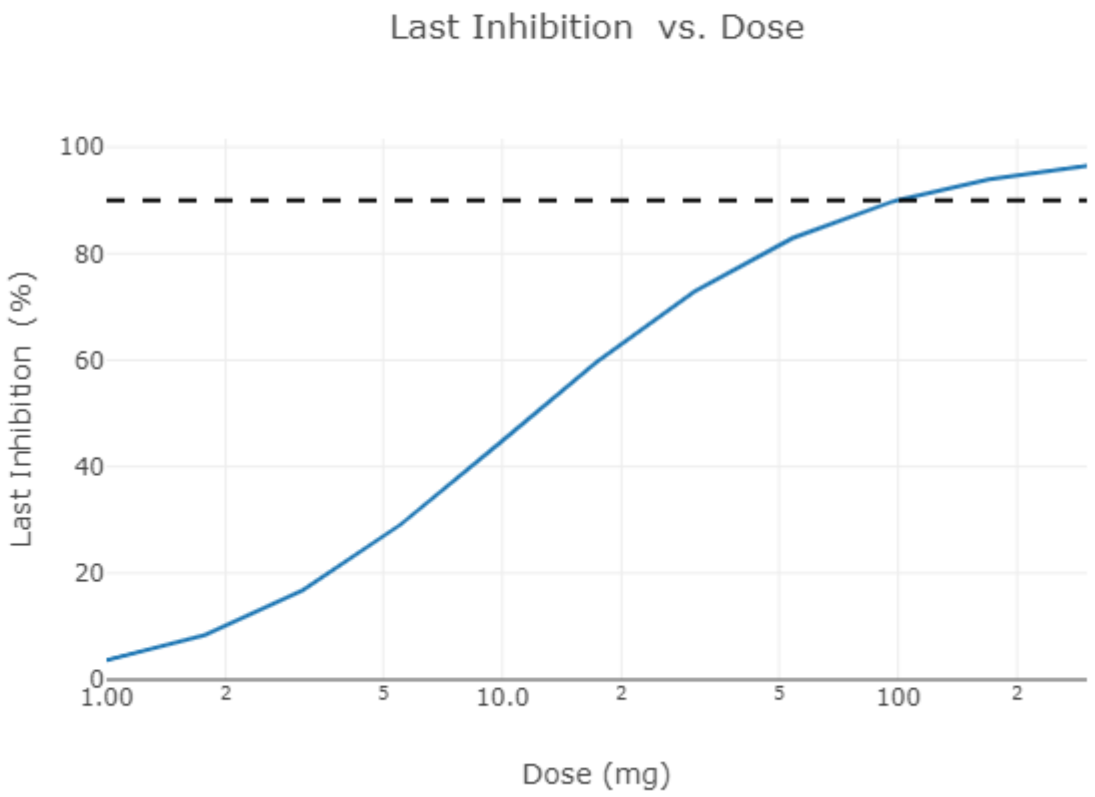

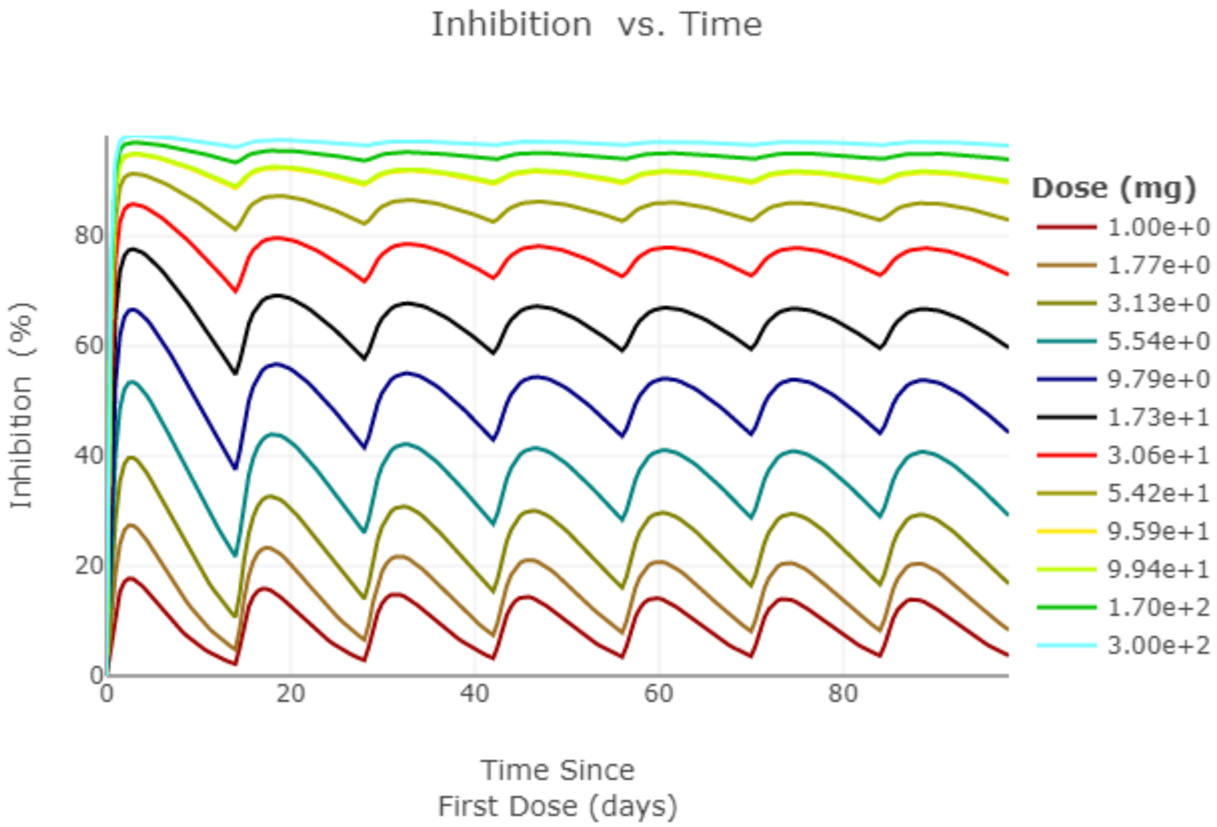
Inhibition vs. Time
Inhibition (%)
0
20
40
60
80
0
20
40
60
80
Time Since
First Dose (days)
Dose (mg)
1.00e+0
1.77e+0
3.13e+0
5.54e+0
9.79e+0
1.73e+1
3.06e+1
5.42e+1
9.59e+1
9.94e+1
1.70e+2
3.00e+2

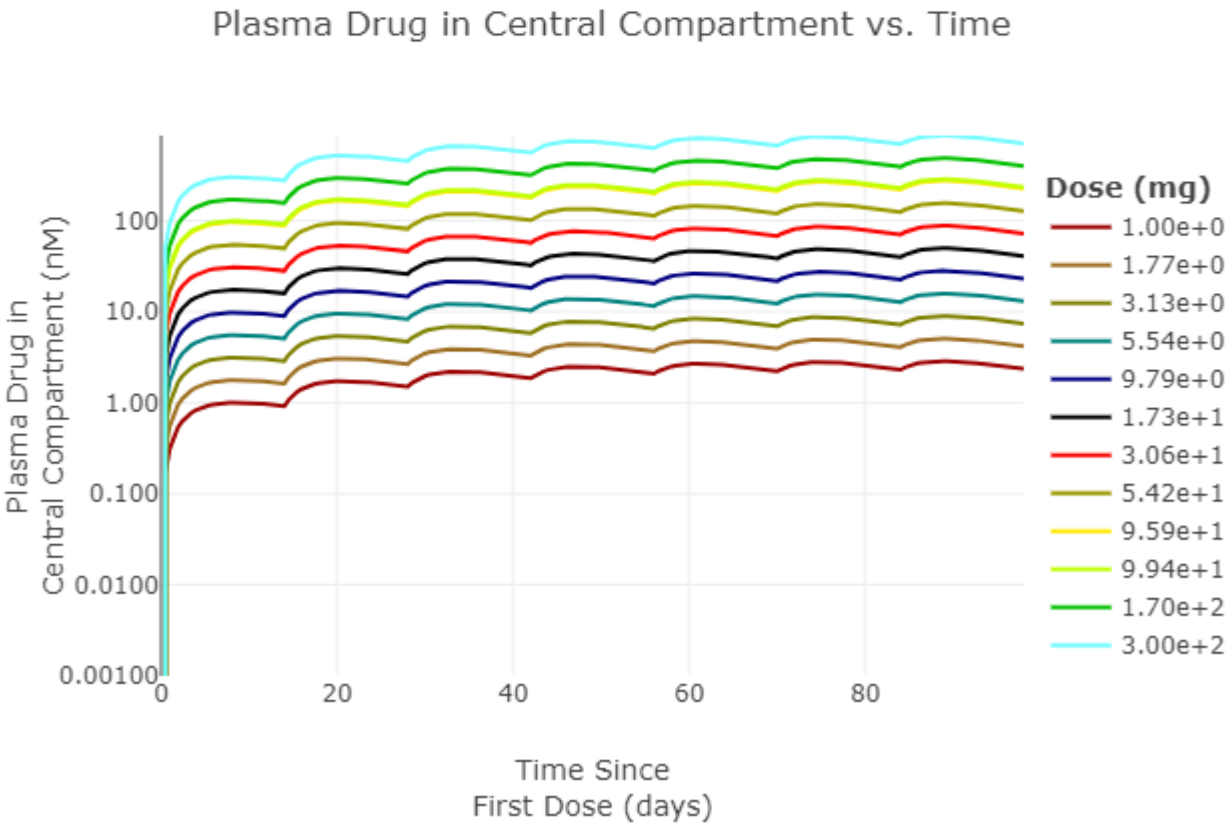
Plasma Drug in Central Compartment vs. Time
Plasma Drug in
Central Compartment (nM)
0.00100
0.0100
0.100
1.00
10.0
100
0
20
40
60
80
Time Since
First Dose (days)
Dose (mg)
1.00e+0
1.77e+0
3.13e+0
5.54e+0
9.79e+0
1.73e+1
3.06e+1
5.42e+1
9.59e+1
9.94e+1
1.70e+2
3.00e+2

2.10) Scenario: ligand:rec affinity high

One dimensional scan over parameter, Dose, between 1 and 300 using log spacing.

2.10.1) Model Parameters

Table 2.10.1

Symbol	Parameter ID	Value	Unit
τ	interval	14	-
D	dose	100	mg
$K_{D,\mathrm{L}}$	mab_kd_1	0.008	nM
N_{doses}	dose_count	7	-
MW	mw_1	148000	Da
$t_{1/2}$	el_half_1	20	days
$t_{1/2,\mathrm{a}}$	abs_half	2.5	days
BW	BW	70	kg
V	volume_central	5	L
$\mathrm{Valency}$	drug_valency_1	1	-
$t_{1/2,\mathrm{L}}$	lig_half_1	30	min
$t_{1/2,\mathrm{R}}$	rec_half_1	540	min
$t_{1/2,\mathrm{sR}}$	shed_half_1	0.5	hr
$K_{D,\mathrm{L:R}}$	lig_rec_kd_1	0.006	nM
$C_{ss,\mathrm{L}}$	lig_css_1_central	0.0000575	nM
$C_{ss,\mathrm{R}}$	rec_css_1_central	0.23	nM
$C_{ss,\mathrm{sR}}$	shed_css_1_central	0	nM
$\mathrm{Density}_{\mathrm{cells}}$	cell_density_mL_central	1000000	#/mL

Parameter ID	Value
Scan Parameter 1	Dose
Lower Limit 1	1
Upper Limit 1	300
N1	11
Scale 1	log

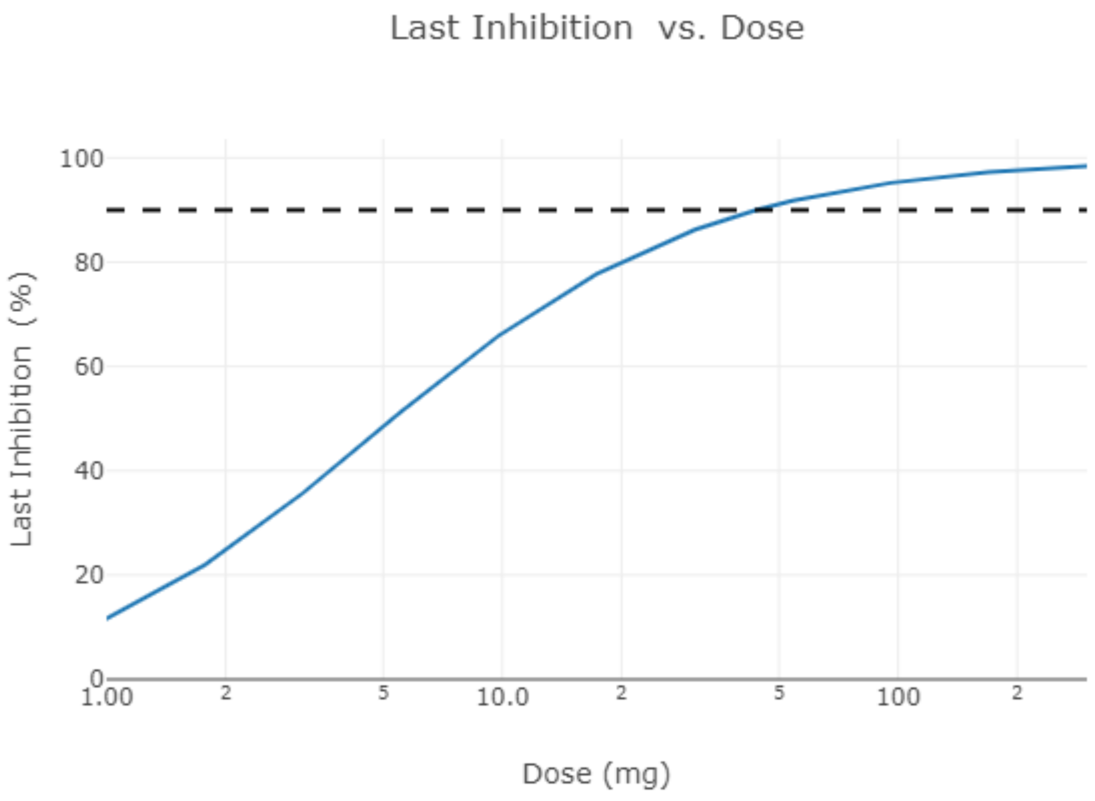

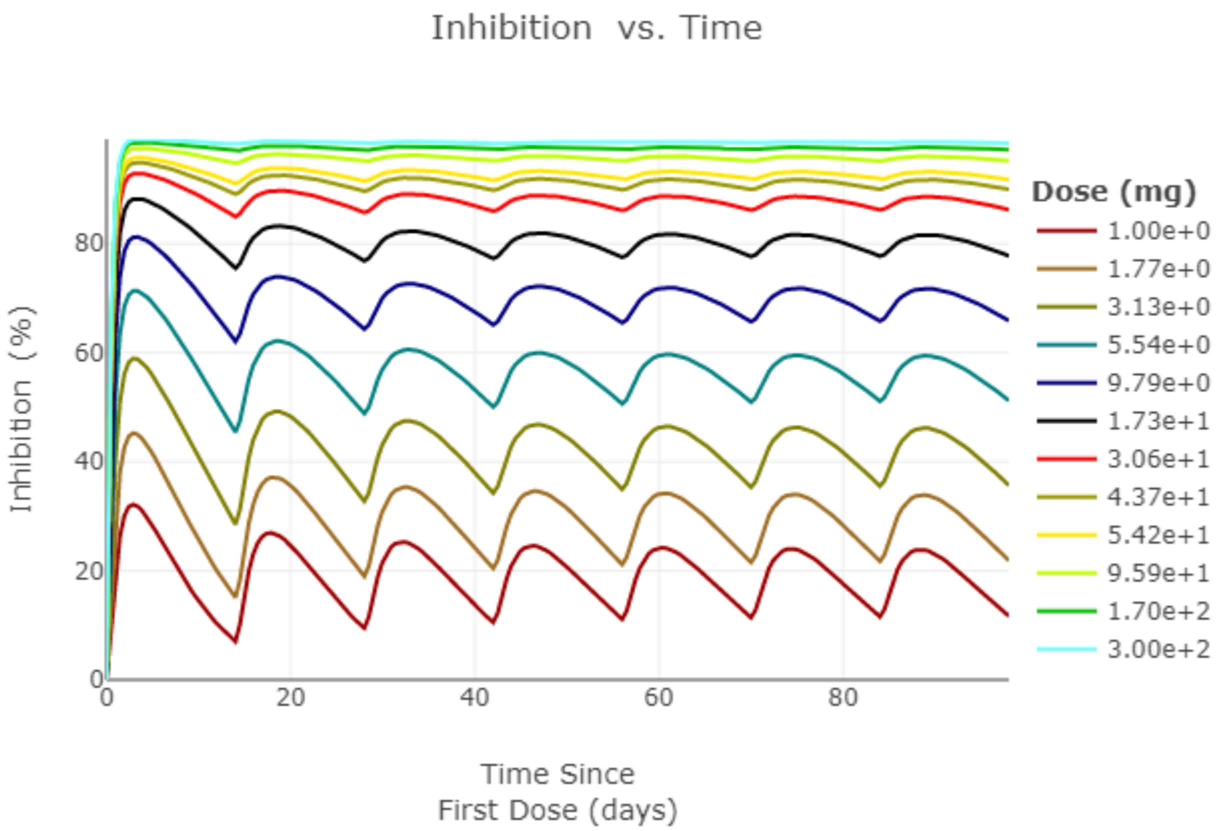

 © Applied BioMath LLC 2017-2021

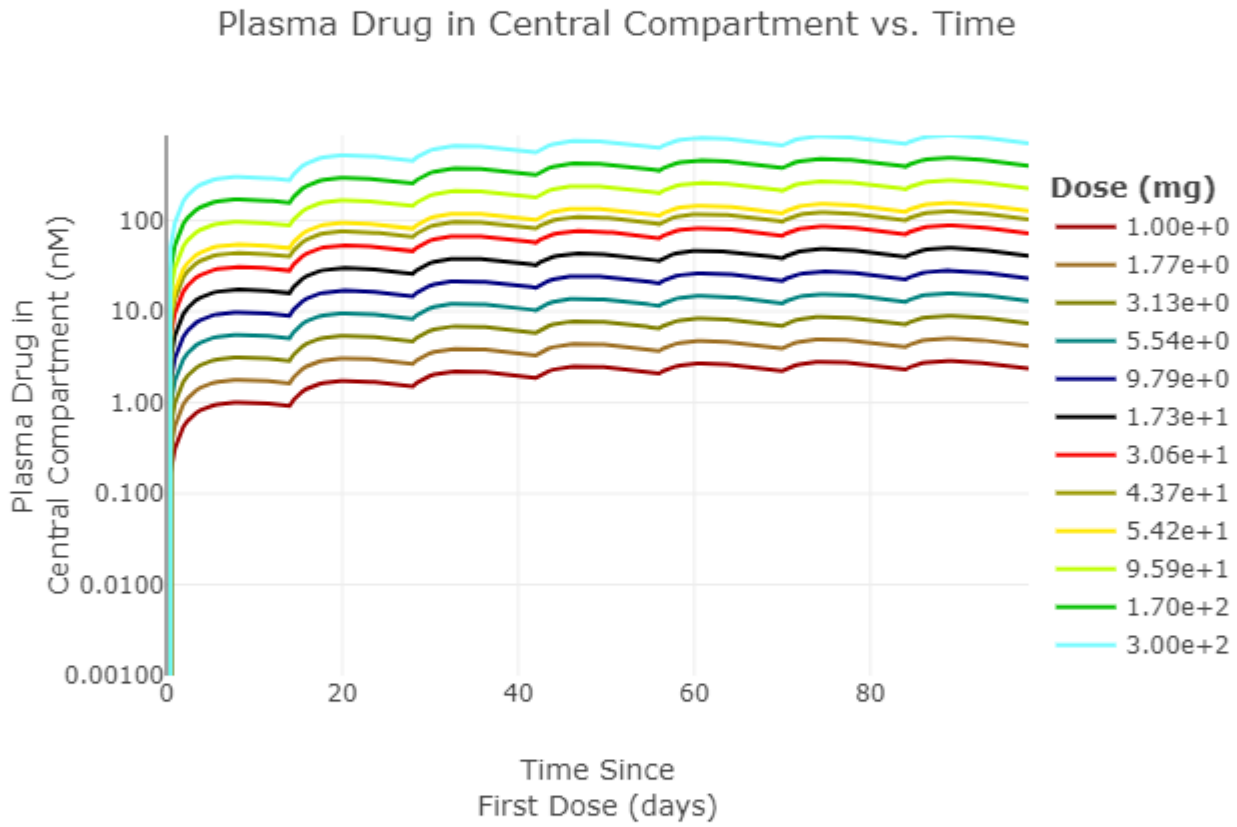

2.11) Scenario: ligand:rec affinity low

One dimensional scan over parameter, Dose, between 1 and 300 using log spacing.

2.11.1) Model Parameters

Table 2.11.1

Symbol	Parameter ID	Value	Unit
τ	interval	14	-
D	dose	100	mg
$K_{D,\mathrm{L}}$	mab_kd_1	0.008	nM
N_{doses}	dose_count	7	-
MW	mw_1	148000	Da
$t_{1/2}$	el_half_1	20	days
$t_{1/2,\mathrm{a}}$	abs_half	2.5	days
BW	BW	70	kg
V	volume_central	5	L
Valency	drug_valency_1	1	-
$t_{1/2,\mathrm{L}}$	lig_half_1	30	min
$t_{1/2,\mathrm{R}}$	rec_half_1	540	min

$t_{1/2,\mathrm{sR}}$	shed_half_1	0.5	hr
$K_{D,\mathrm{L:R}}$	lig_rec_kd_1	0.057	nM
$C_{SS,\mathrm{L}}$	lig_css_1_central	0.0000575	nM
$C_{SS,\mathrm{R}}$	rec_css_1_central	0.23	nM
$C_{SS,\mathrm{sR}}$	shed_css_1_central	0	nM
$\mathrm{Density}_{\mathrm{cells}}$	cell_density_mL_central	1000000	#/mL

Parameter ID	Value
Scan Parameter 1	Dose
Lower Limit 1	1
Upper Limit 1	300
N1	11
Scale 1	log

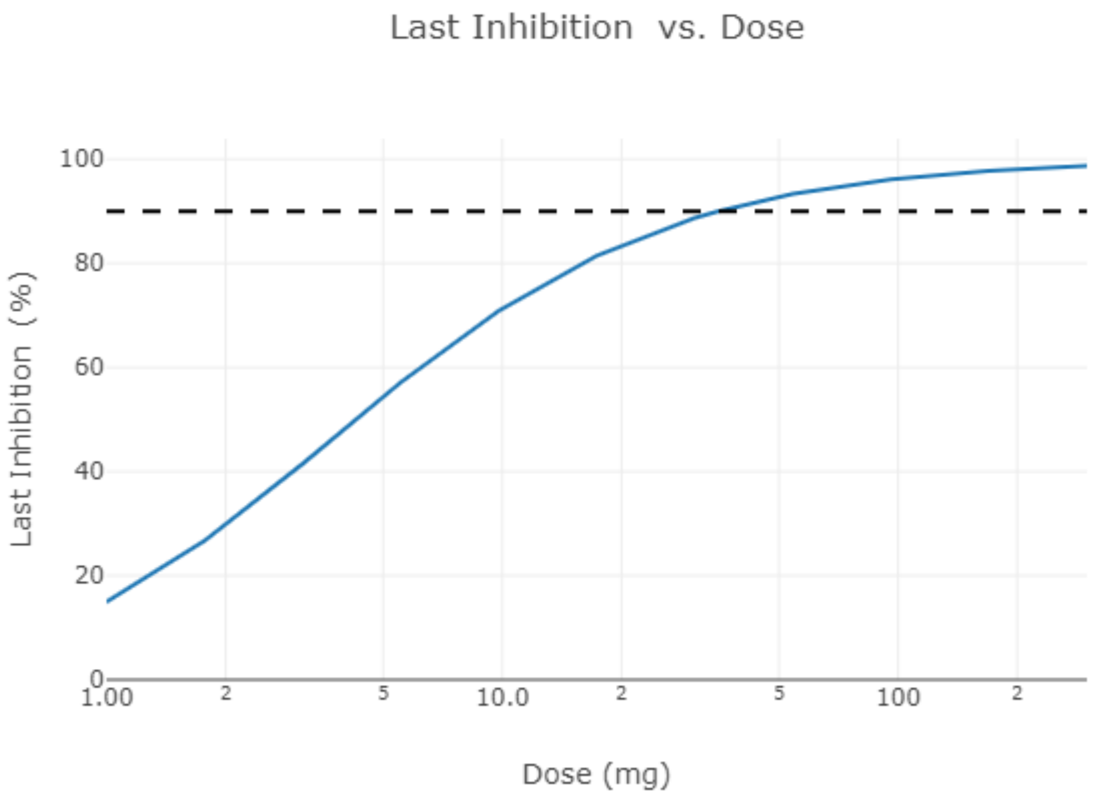

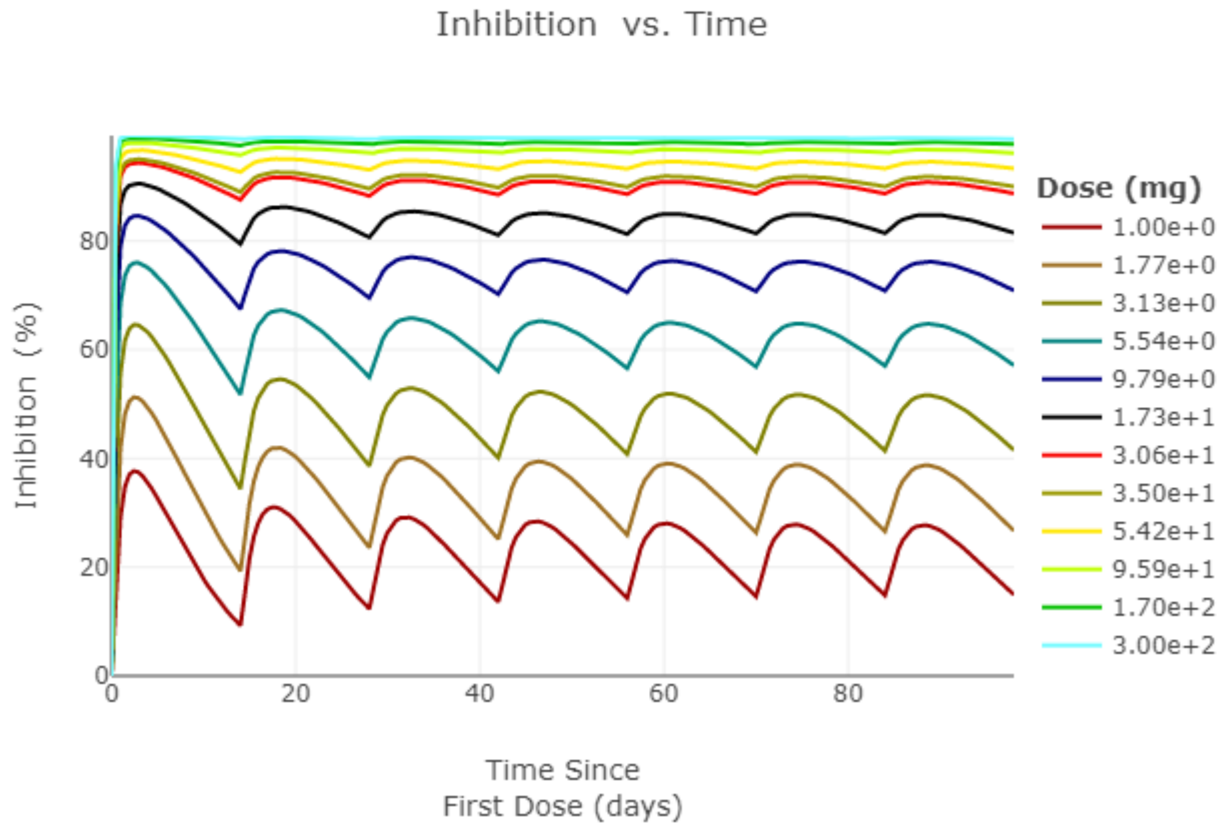

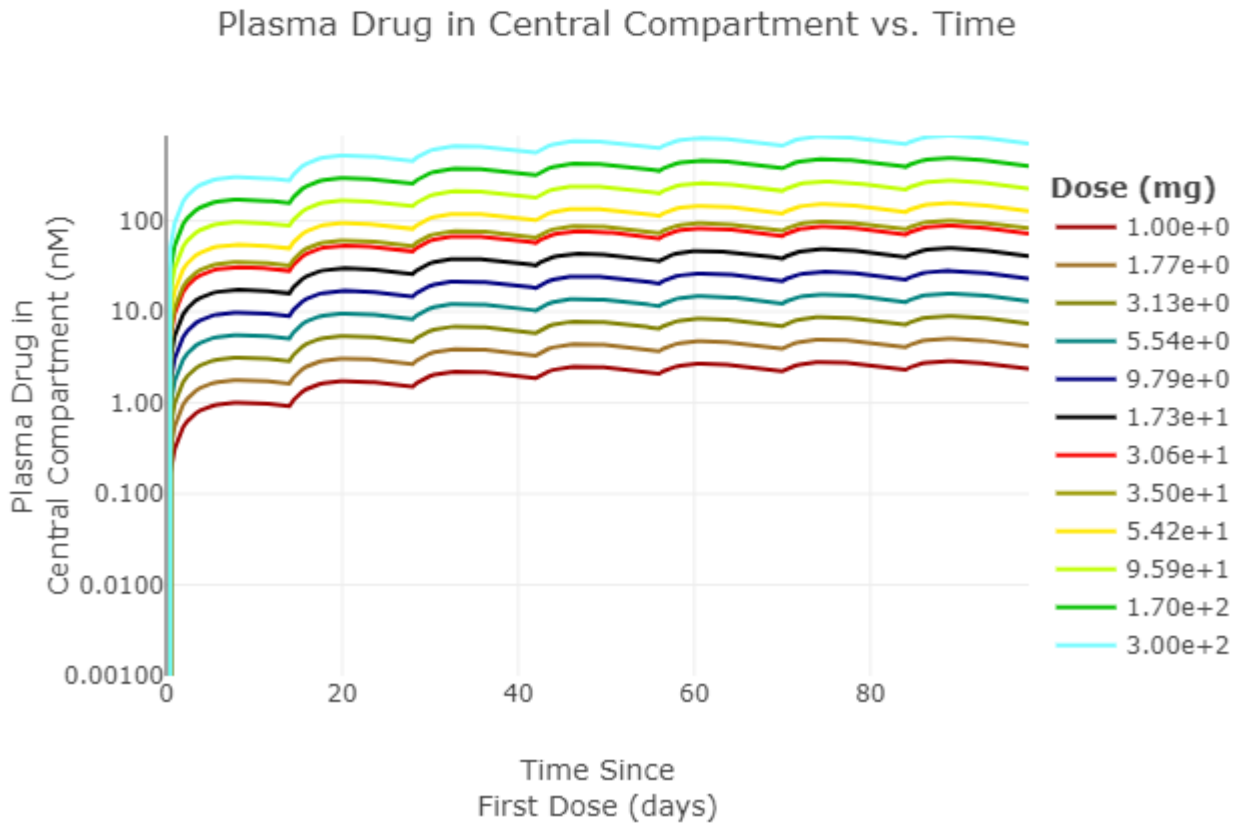

 © Applied BioMath LLC 2017-2021

2.12) Scenario: drug_affinity_high

One dimensional scan over parameter, Dose, between 1 and 300 using log spacing.

2.12.1) Model Parameters

Table 2.12.1

Symbol	Parameter ID	Value	Unit
τ	interval	14	-
D	dose	100	mg
$K_{D,\mathrm{L}}$	mab_kd_1	0.0027	nM
N_{doses}	dose_count	7	-
MW	mw_1	148000	Da
$t_{1/2}$	el_half_1	20	days
$t_{1/2,\mathrm{a}}$	abs_half	2.5	days
BW	BW	70	kg
V	volume_central	5	L
$\mathrm{Valency}$	drug_valency_1	1	-
$t_{1/2,\mathrm{L}}$	lig_half_1	30	min
$t_{1/2,\mathrm{R}}$	rec_half_1	540	min
$t_{1/2,\mathrm{sR}}$	shed_half_1	0.5	hr
$K_{D,\mathrm{L:R}}$	lig_rec_kd_1	0.019	nM
$C_{ss,\mathrm{L}}$	lig_css_1_central	0.0000575	nM
$C_{ss,\mathrm{R}}$	rec_css_1_central	0.23	nM
$C_{ss,\mathrm{sR}}$	shed_css_1_central	0	nM
$\mathrm{Density}_{\mathrm{cells}}$	cell_density_mL_central	1000000	#/mL

Parameter ID	Value
Scan Parameter 1	Dose
Lower Limit 1	1
Upper Limit 1	300
N1	11
Scale 1	log

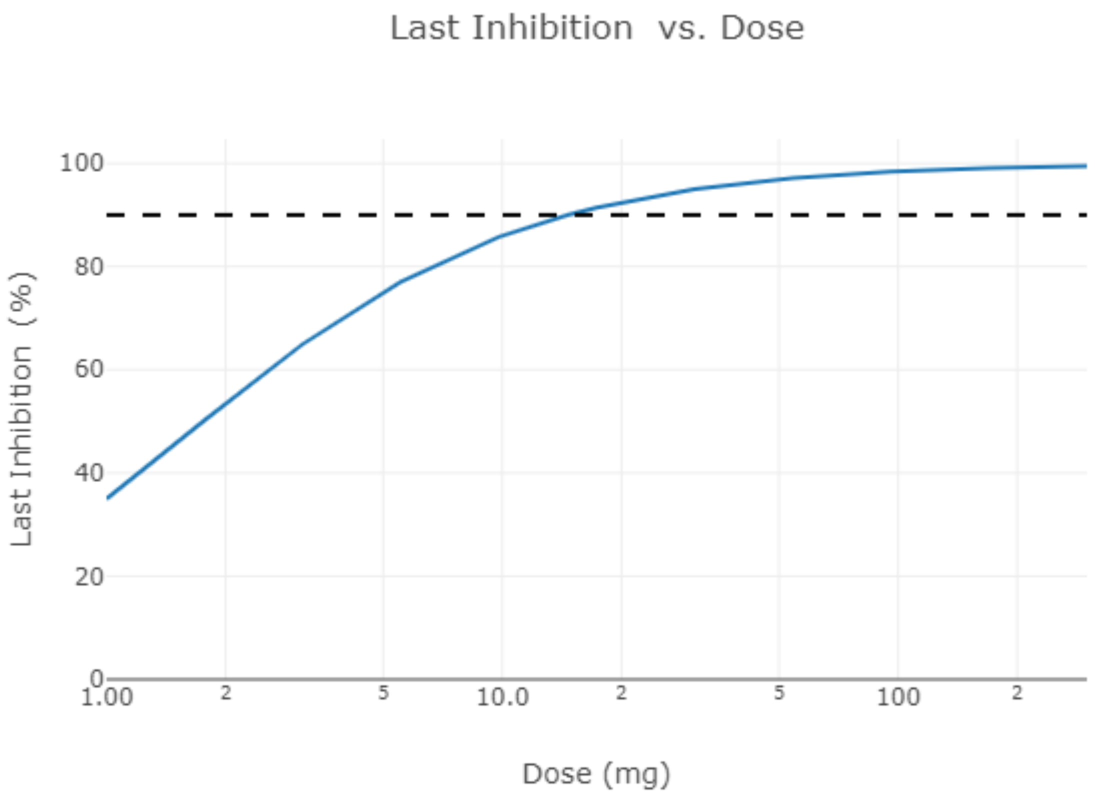
Last Inhibition vs. Dose
Last Inhibition (%)
100
80
60
40
20
0
1.00
2
5
10.0
2
5
100
2
Dose (mg)

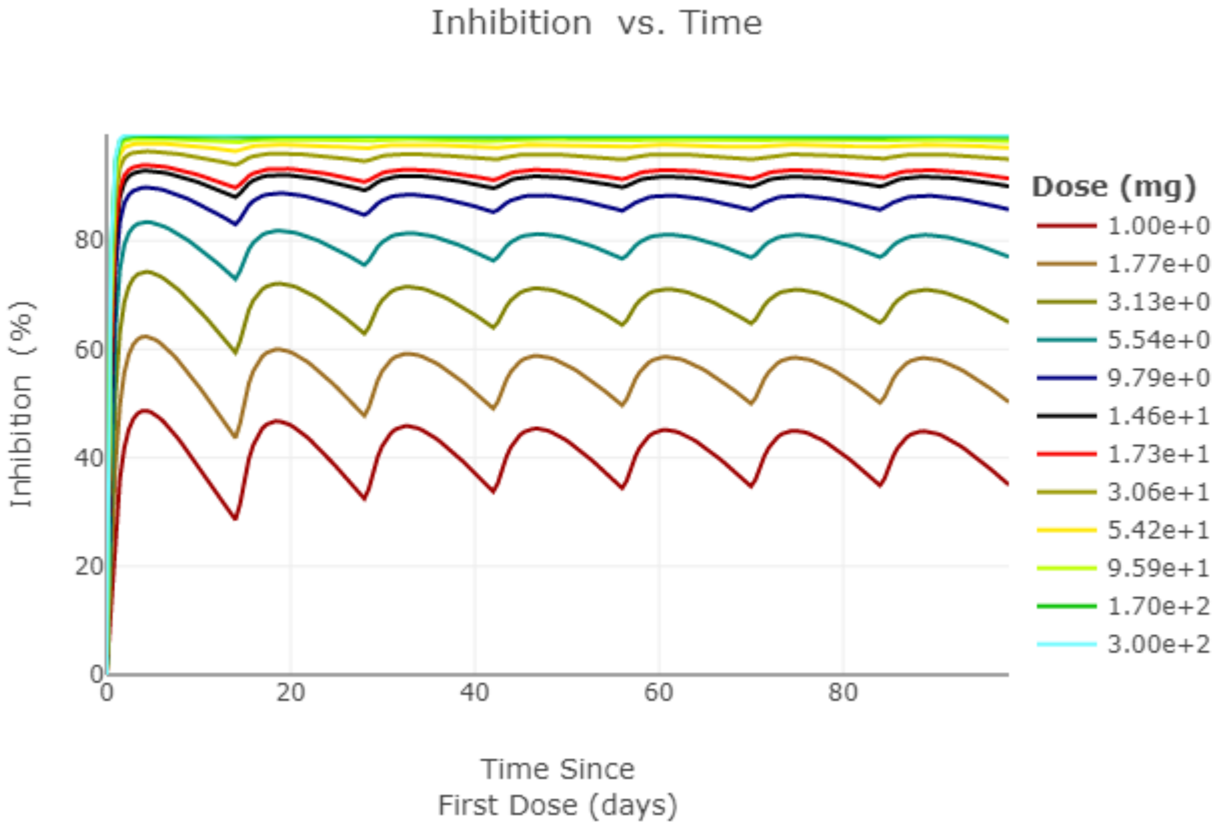
Inhibition vs. Time
Inhibition (%)
80
60
40
20
0
0
20
40
60
80
Time Since
First Dose (days)
Dose (mg)
1.00e+0
1.77e+0
3.13e+0
5.54e+0
9.79e+0
1.46e+1
1.73e+1
3.06e+1
5.42e+1
9.59e+1
1.70e+2
3.00e+2

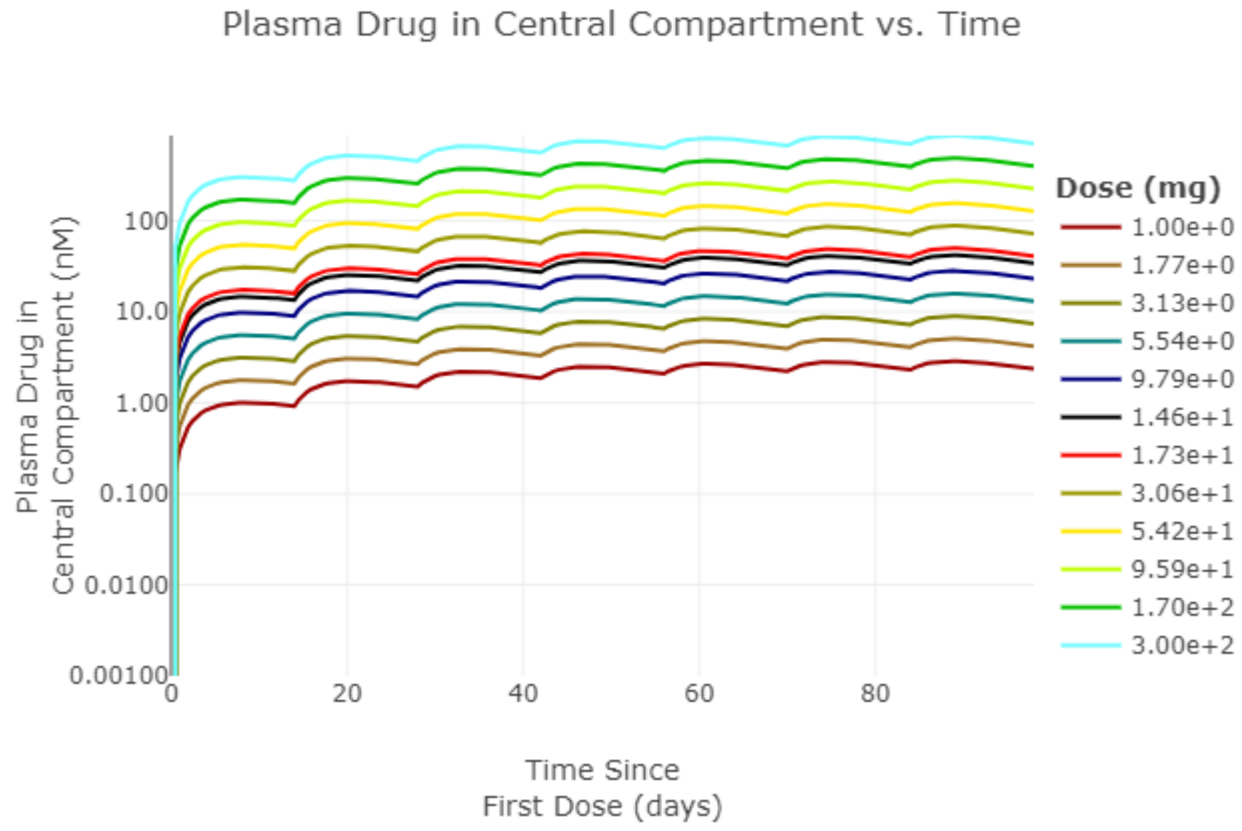

2.13) Scenario: drug_affinity_low

One dimensional scan over parameter, Dose, between 1 and 300 using log spacing.

2.13.1) Model Parameters

Table 2.13.1

Symbol	Parameter ID	Value	Unit
τ	interval	14	-
D	dose	100	mg
$K_{D,\mathrm{L}}$	mab_kd_1	0.024	nM
N_{doses}	dose_count	7	-
MW	mw_1	148000	Da
$t_{1/2}$	el_half_1	20	days
$t_{1/2,\mathrm{a}}$	abs_half	2.5	days
BW	BW	70	kg
V	volume_central	5	L
Valency	drug_valency_1	1	-
$t_{1/2,\mathrm{L}}$	lig_half_1	30	min
$t_{1/2,\mathrm{R}}$	rec_half_1	540	min

 © Applied BioMath LLC 2017-2021

$t_{1/2,\mathrm{sR}}$	shed_half_1	0.5	hr
$K_{D,\mathrm{L:R}}$	lig_rec_kd_1	0.019	nM
$C_{SS,\mathrm{L}}$	lig_css_1_central	0.0000575	nM
$C_{SS,\mathrm{R}}$	rec_css_1_central	0.23	nM
$C_{SS,\mathrm{sR}}$	shed_css_1_central	0	nM
$\mathrm{Density}_{\mathrm{cells}}$	cell_density_mL_central	1000000	#/mL

Parameter ID	Value
Scan Parameter 1	Dose
Lower Limit 1	1
Upper Limit 1	300
N1	11
Scale 1	log

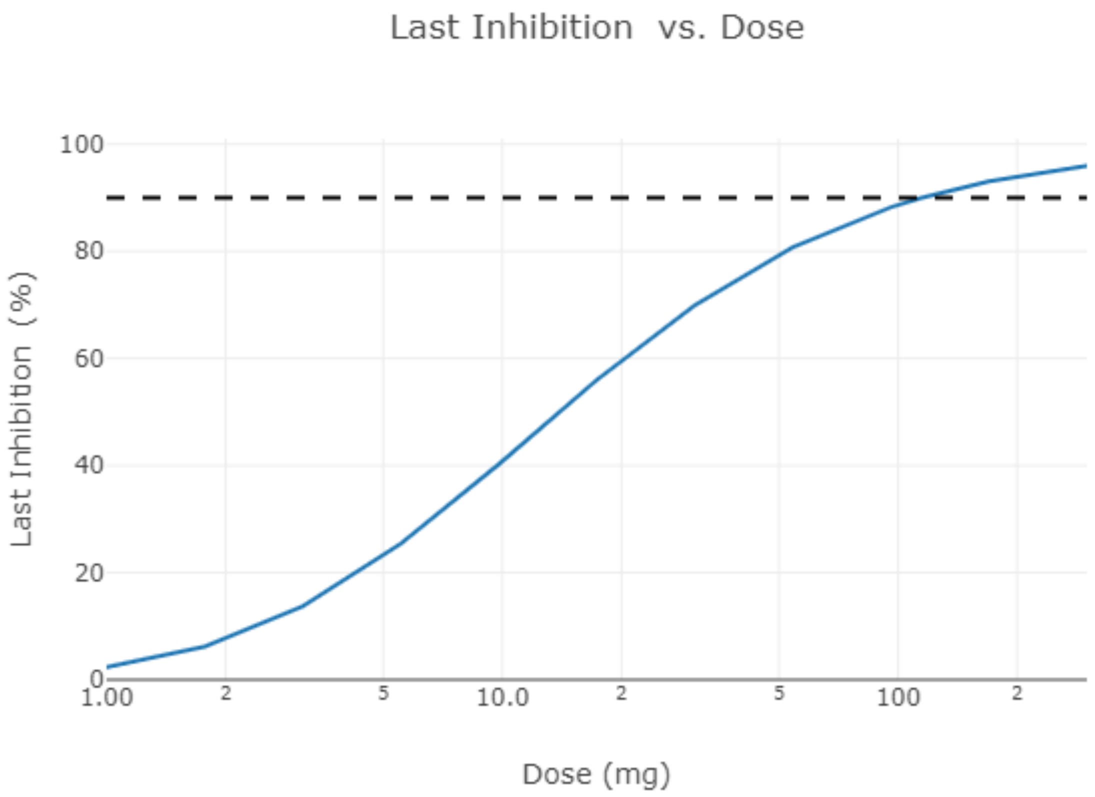

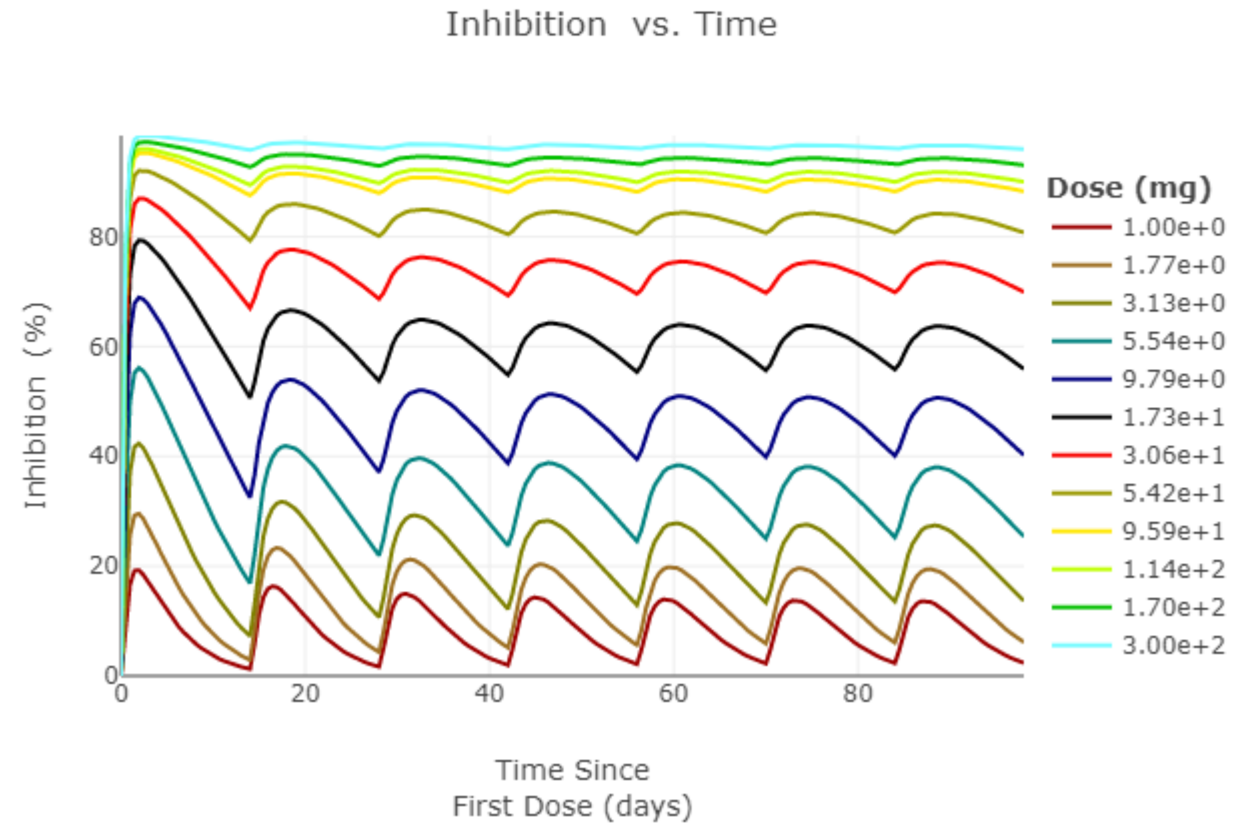
Inhibition vs. Time
Inhibition (%)
0
20
40
60
80
0
20
40
60
80
Time Since
First Dose (days)
Dose (mg)
1.00e+0
1.77e+0
3.13e+0
5.54e+0
9.79e+0
1.73e+1
3.06e+1
5.42e+1
9.59e+1
1.14e+2
1.70e+2
3.00e+2

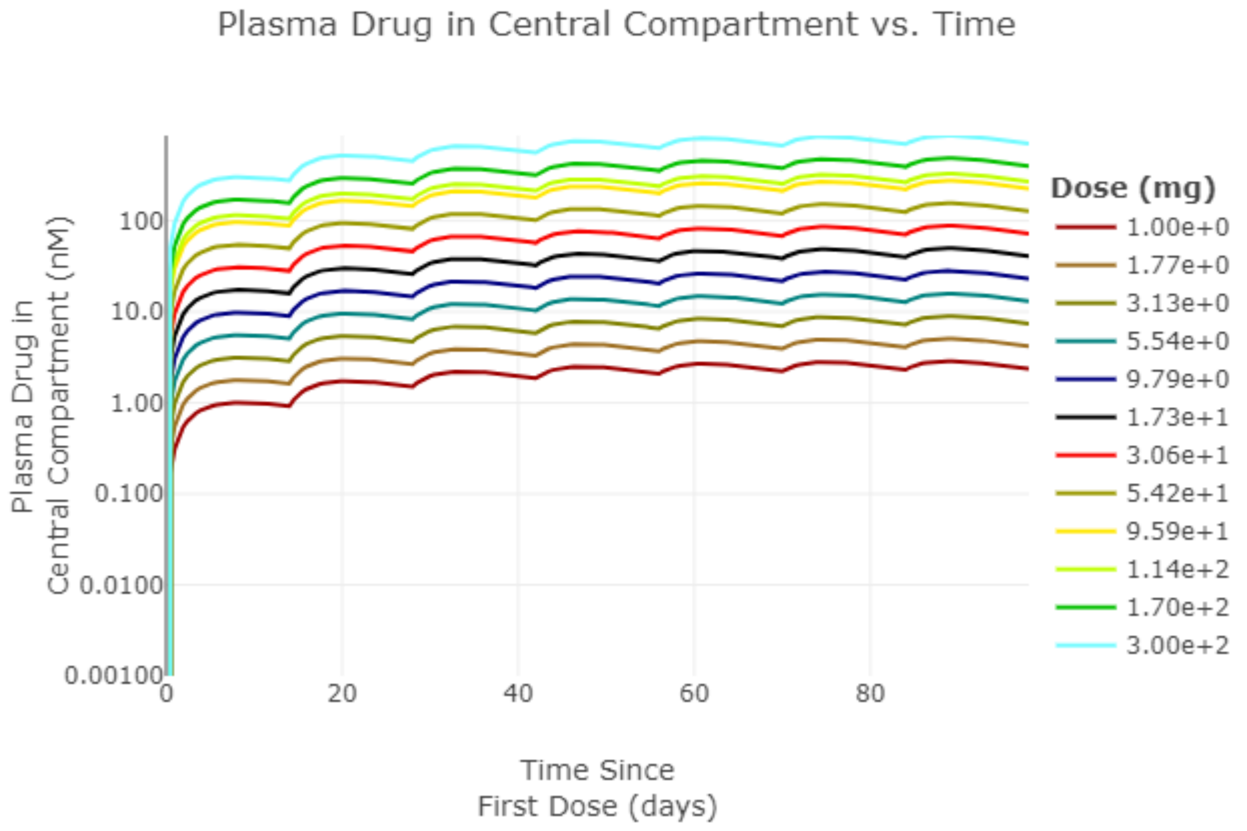
Plasma Drug in Central Compartment vs. Time
Plasma Drug in
Central Compartment (nM)
0.00100
0.0100
0.100
1.00
10.0
100
0
20
40
60
80
Time Since
First Dose (days)
Dose (mg)
1.00e+0
1.77e+0
3.13e+0
5.54e+0
9.79e+0
1.73e+1
3.06e+1
5.42e+1
9.59e+1
1.14e+2
1.70e+2
3.00e+2

2.14) Scenario: receptor_conc_high

One dimensional scan over parameter, Dose, between 1 and 300 using log spacing.

2.14.1) Model Parameters

Table 2.14.1

Symbol	Parameter ID	Value	Unit
τ	interval	14	-
D	dose	100	mg
$K_{D,\mathrm{L}}$	mab_kd_1	0.008	nM
N_{doses}	dose_count	7	-
MW	mw_1	148000	Da
$t_{1/2}$	el_half_1	20	days
$t_{1/2,\mathrm{a}}$	abs_half	2.5	days
BW	BW	70	kg
V	volume_central	5	L
$\mathrm{Valency}$	drug_valency_1	1	-
$t_{1/2,\mathrm{L}}$	lig_half_1	30	min
$t_{1/2,\mathrm{R}}$	rec_half_1	540	min
$t_{1/2,\mathrm{sR}}$	shed_half_1	0.5	hr
$K_{D,\mathrm{L:R}}$	lig_rec_kd_1	0.019	nM
$C_{ss,\mathrm{L}}$	lig_css_1_central	0.0000575	nM
$C_{ss,\mathrm{R}}$	rec_css_1_central	0.69	nM
$C_{ss,\mathrm{sR}}$	shed_css_1_central	0	nM
$\mathrm{Density}_{\mathrm{cells}}$	cell_density_mL_central	1000000	#/mL

Parameter ID	Value
Scan Parameter 1	Dose
Lower Limit 1	1
Upper Limit 1	300
N1	11
Scale 1	log

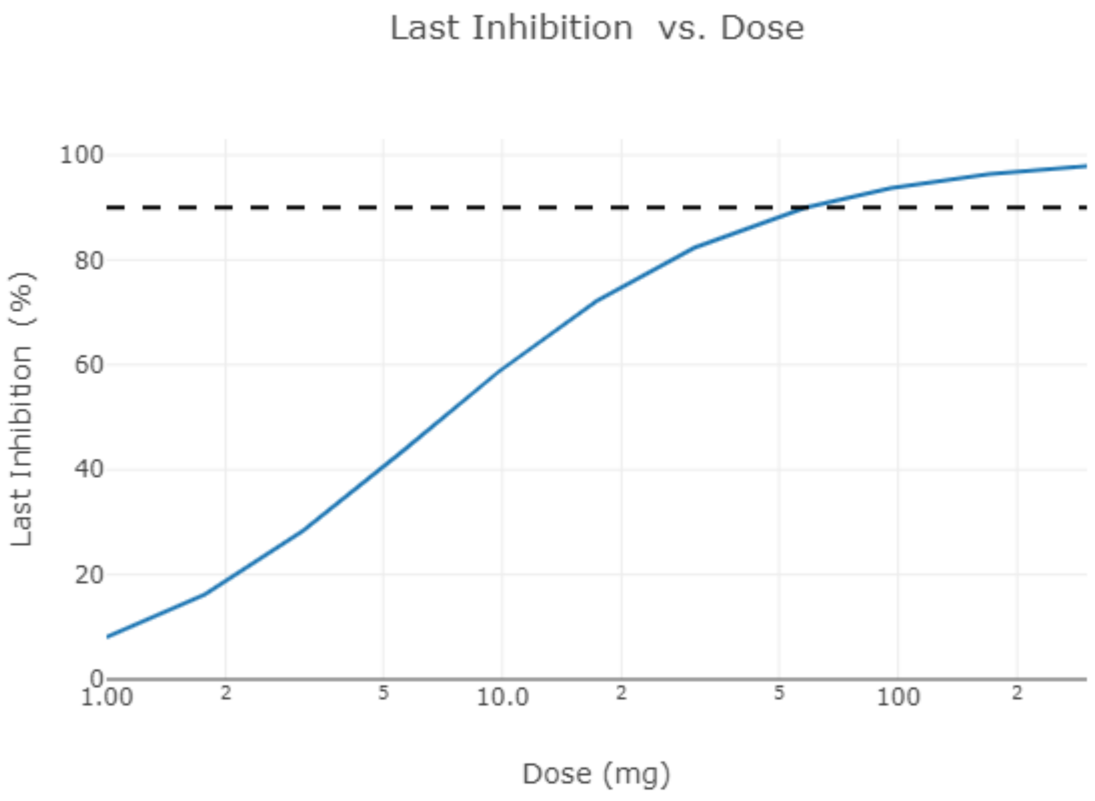
Last Inhibition vs. Dose
Last Inhibition (%)
100
80
60
40
20
0
1.00
2
5
10.0
2
5
100
2
Dose (mg)

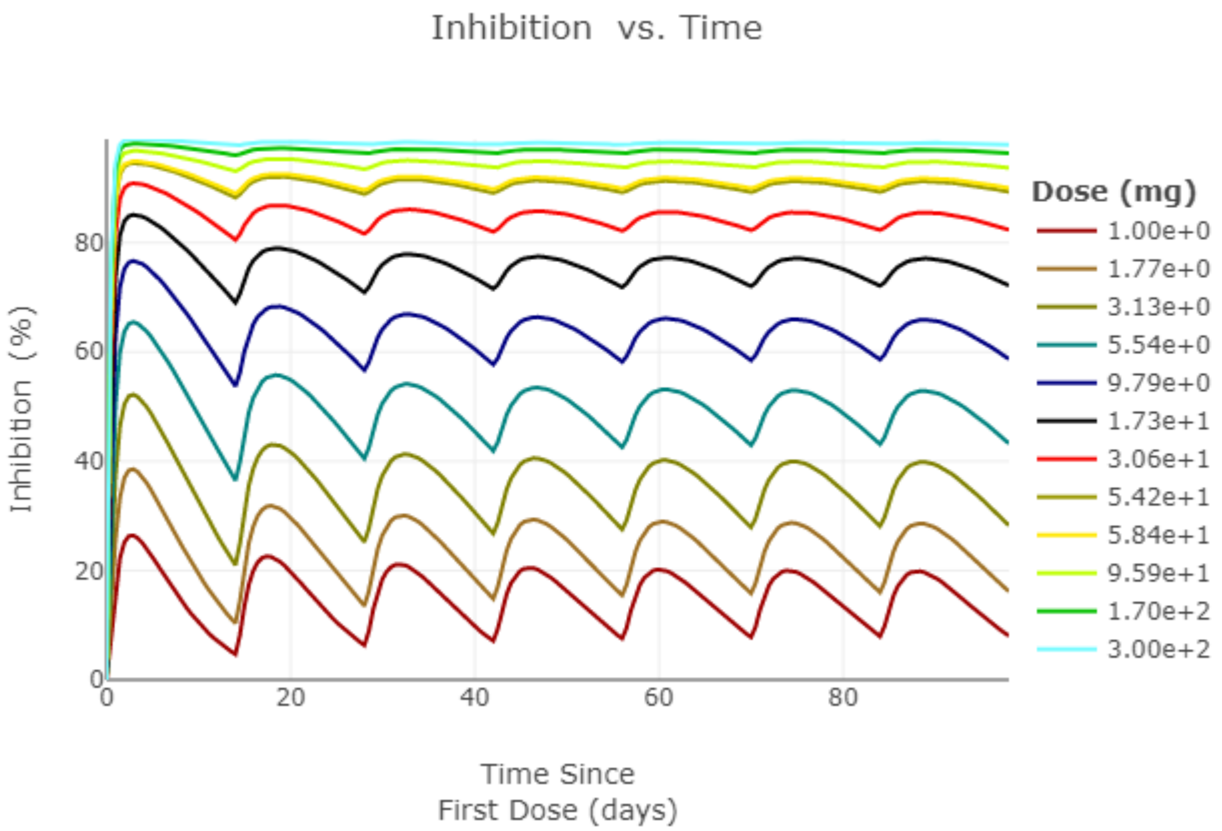
Inhibition vs. Time
Inhibition (%)
80
60
40
20
0
0
20
40
60
80
Time Since
First Dose (days)
Dose (mg)
1.00e+0
1.77e+0
3.13e+0
5.54e+0
9.79e+0
1.73e+1
3.06e+1
5.42e+1
5.84e+1
9.59e+1
1.70e+2
3.00e+2

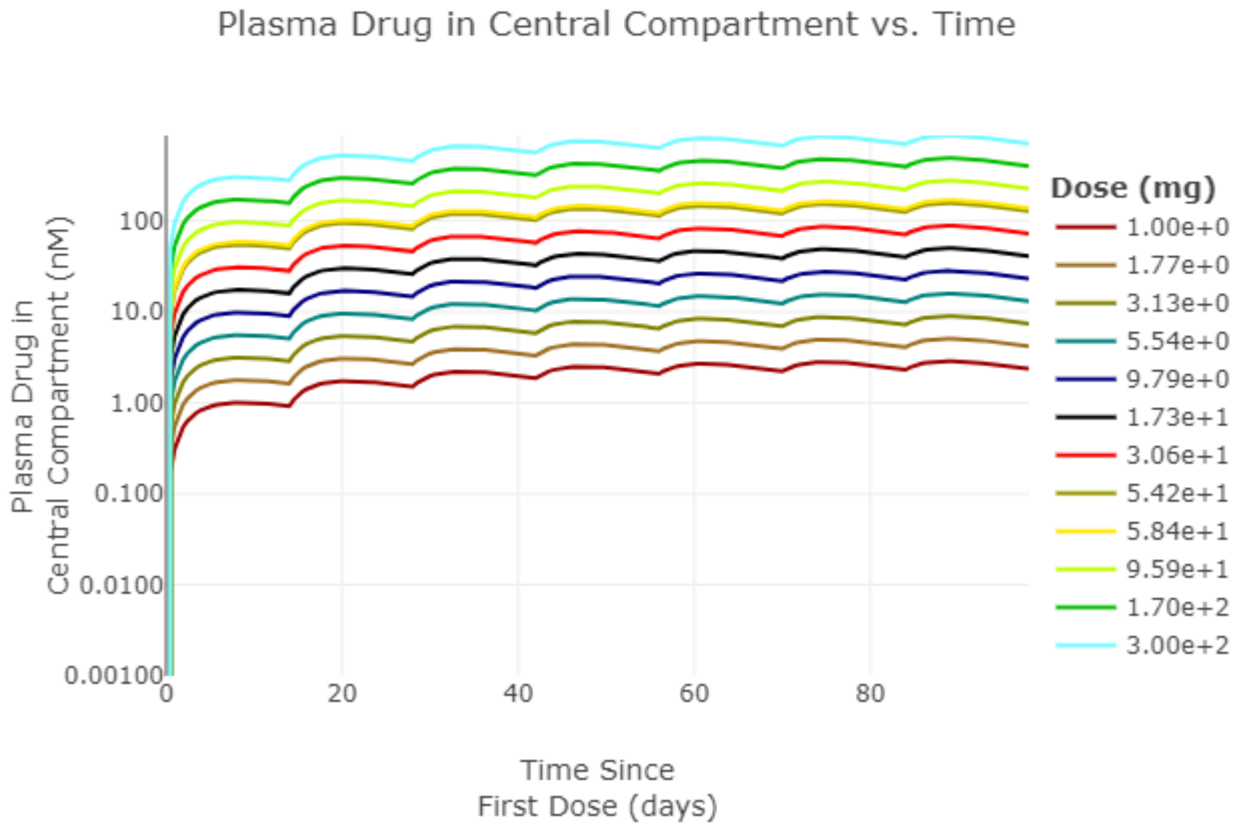

2.15) Scenario: receptor_conc_low

One dimensional scan over parameter, Dose, between 1 and 300 using log spacing.

2.15.1) Model Parameters

Table 2.15.1

Symbol	Parameter ID	Value	Unit
τ	interval	14	-
D	dose	100	mg
$K_{D,\mathrm{L}}$	mab_kd_1	0.008	nM
N_{doses}	dose_count	7	-
MW	mw_1	148000	Da
$t_{1/2}$	el_half_1	20	days
$t_{1/2,\mathrm{a}}$	abs_half	2.5	days
BW	BW	70	kg
V	volume_central	5	L
Valency	drug_valency_1	1	-
$t_{1/2,\mathrm{L}}$	lig_half_1	30	min
$t_{1/2,\mathrm{R}}$	rec_half_1	540	min

$t_{1/2,\mathrm{sR}}$	shed_half_1	0.5	hr
$K_{D,\mathrm{L:R}}$	lig_rec_kd_1	0.019	nM
$C_{SS,\mathrm{L}}$	lig_css_1_central	0.0000575	nM
$C_{SS,\mathrm{R}}$	rec_css_1_central	0.23	nM
$C_{SS,\mathrm{sR}}$	shed_css_1_central	0	nM
$\mathrm{Density}_{\mathrm{cells}}$	cell_density_mL_central	1000000	#/mL

Parameter ID	Value
Scan Parameter 1	Dose
Lower Limit 1	1
Upper Limit 1	300
N1	11
Scale 1	log

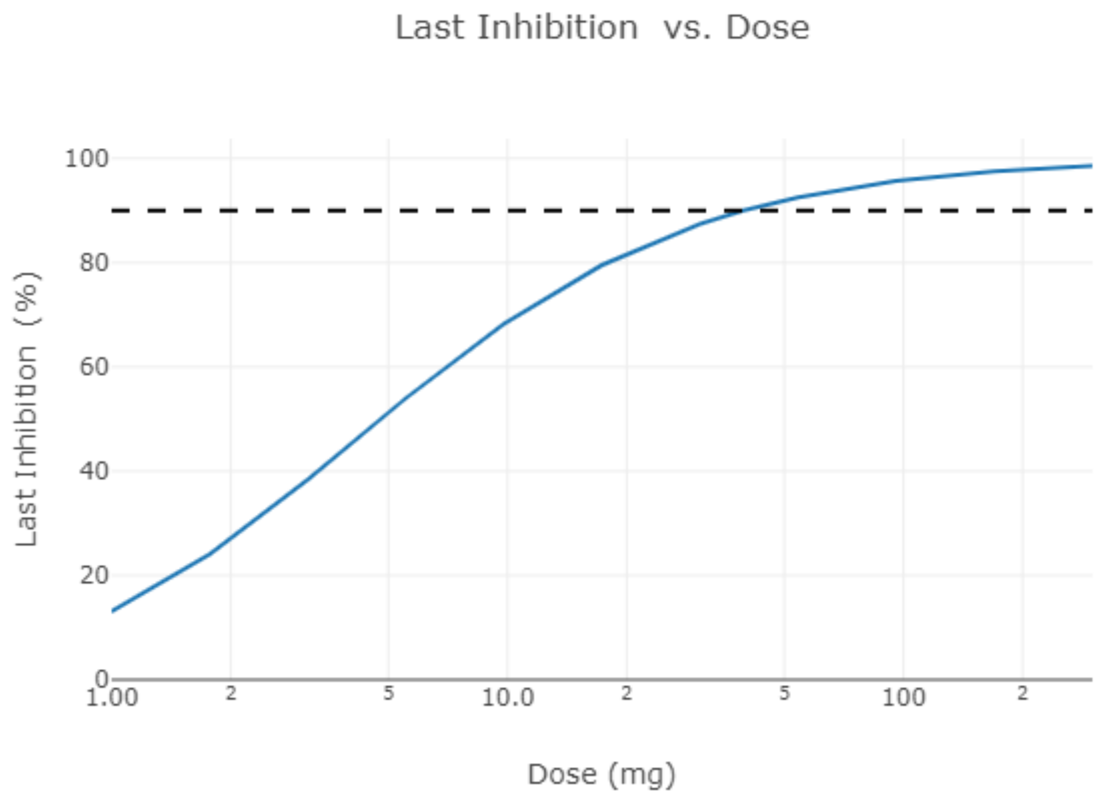

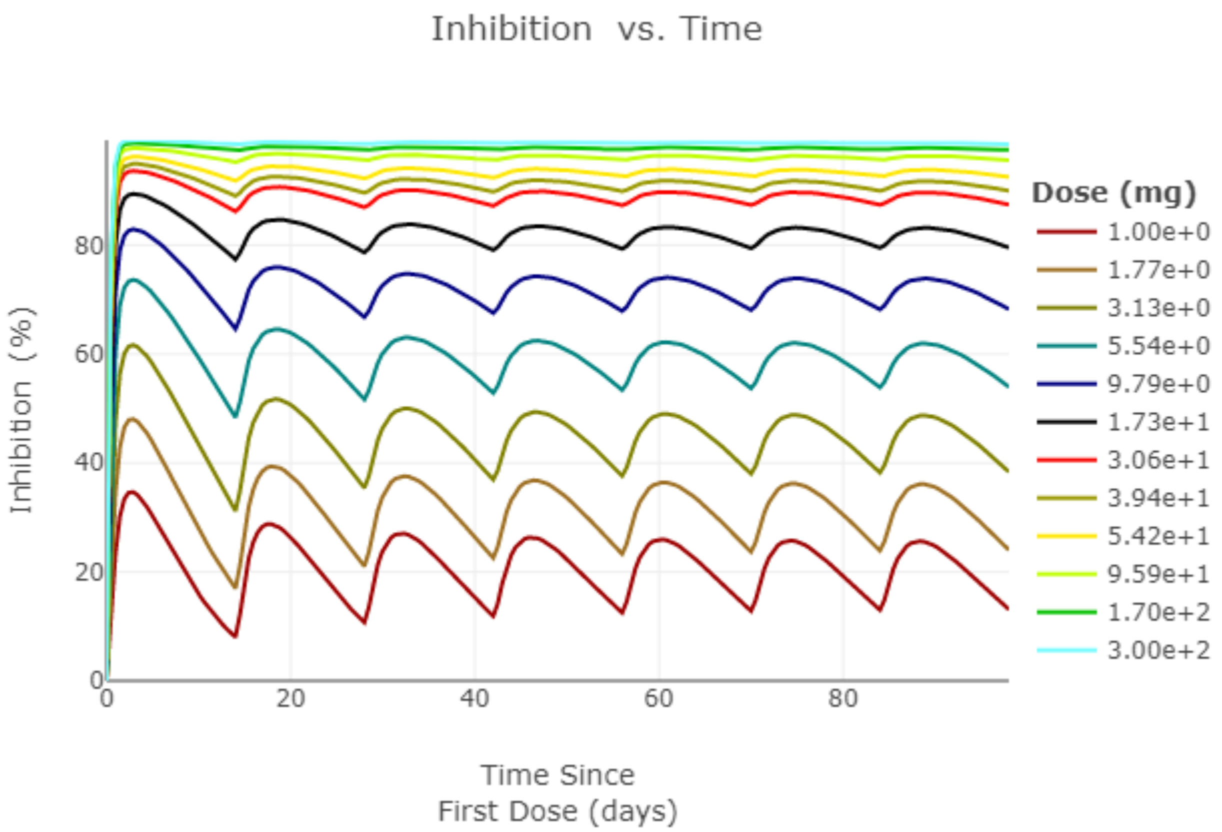
Inhibition vs. Time
Inhibition (%)
0
20
40
60
80
0
20
40
60
80
Time Since
First Dose (days)
Dose (mg)
1.00e+0
1.77e+0
3.13e+0
5.54e+0
9.79e+0
1.73e+1
3.06e+1
3.94e+1
5.42e+1
9.59e+1
1.70e+2
3.00e+2

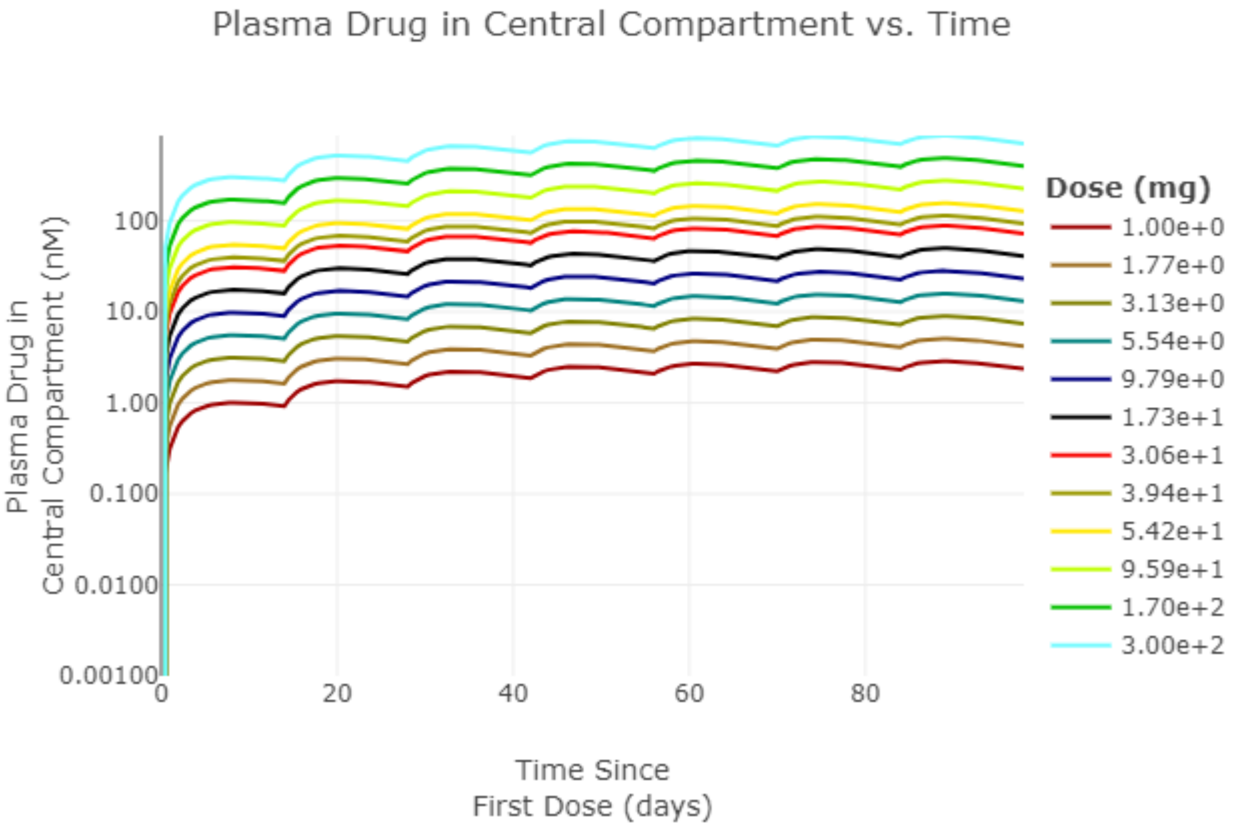
Plasma Drug in Central Compartment vs. Time
Plasma Drug in
Central Compartment (nM)
0.00100
0.0100
0.100
1.00
10.0
100
0
20
40
60
80
Time Since
First Dose (days)
Dose (mg)
1.00e+0
1.77e+0
3.13e+0
5.54e+0
9.79e+0
1.73e+1
3.06e+1
3.94e+1
5.42e+1
9.59e+1
1.70e+2
3.00e+2

2.16) Scenario: rec_halflife_high

One dimensional scan over parameter, Dose, between 1 and 300 using log spacing.

2.16.1) Model Parameters

Table 2.16.1

Symbol	Parameter ID	Value	Unit
τ	interval	14	-
D	dose	100	mg
$K_{D,\mathrm{L}}$	mab_kd_1	0.008	nM
N_{doses}	dose_count	7	-
MW	mw_1	148000	Da
$t_{1/2}$	el_half_1	20	days
$t_{1/2,\mathrm{a}}$	abs_half	2.5	days
BW	BW	70	kg
V	volume_central	5	L
$\mathrm{Valency}$	drug_valency_1	1	-
$t_{1/2,\mathrm{L}}$	lig_half_1	30	min
$t_{1/2,\mathrm{R}}$	rec_half_1	1620	min
$t_{1/2,\mathrm{sR}}$	shed_half_1	0.5	hr
$K_{D,\mathrm{L:R}}$	lig_rec_kd_1	0.019	nM
$C_{ss,\mathrm{L}}$	lig_css_1_central	0.0000575	nM
$C_{ss,\mathrm{R}}$	rec_css_1_central	0.23	nM
$C_{ss,\mathrm{sR}}$	shed_css_1_central	0	nM
$\mathrm{Density}_{\mathrm{cells}}$	cell_density_mL_central	1000000	#/mL

Parameter ID	Value
Scan Parameter 1	Dose
Lower Limit 1	1
Upper Limit 1	300
N1	11
Scale 1	log

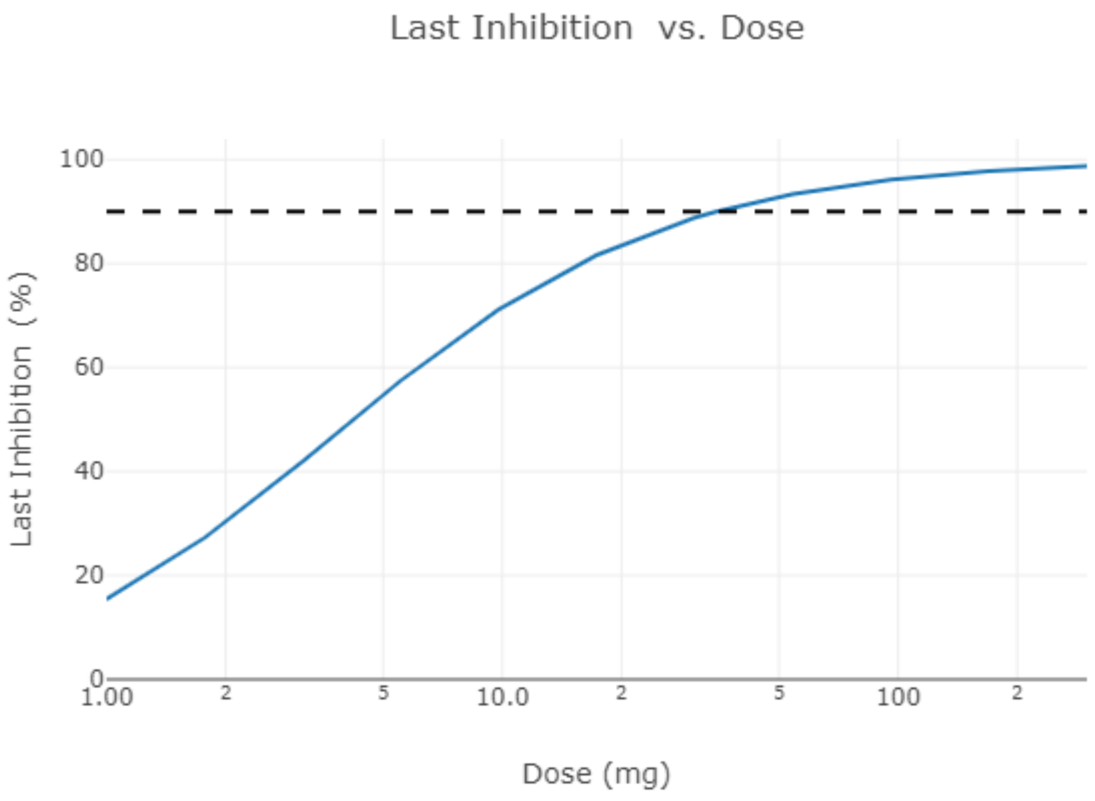
Last Inhibition vs. Dose
Last Inhibition (%)
100
80
60
40
20
0
1.00
2
5
10.0
2
5
100
2
Dose (mg)

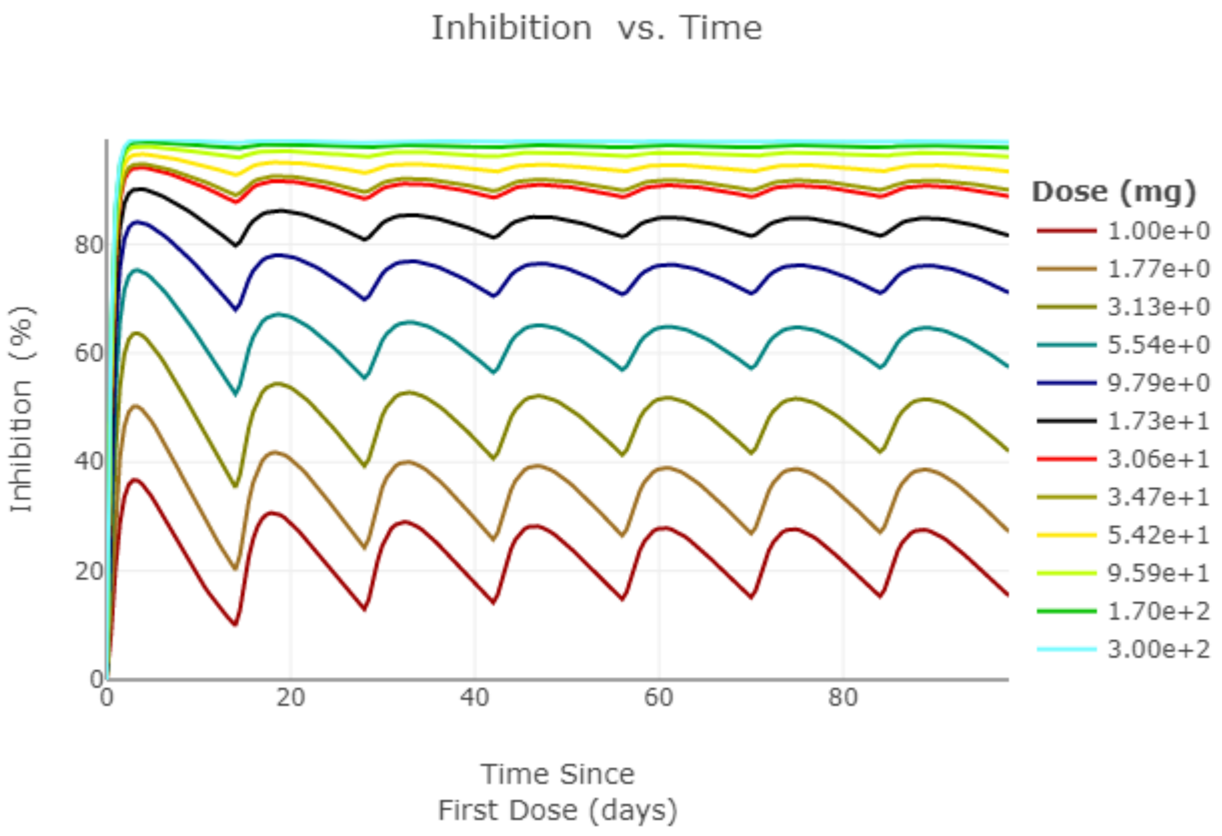
Inhibition vs. Time
Inhibition (%)
80
60
40
20
0
0
20
40
60
80
Time Since
First Dose (days)
Dose (mg)
1.00e+0
1.77e+0
3.13e+0
5.54e+0
9.79e+0
1.73e+1
3.06e+1
3.47e+1
5.42e+1
9.59e+1
1.70e+2
3.00e+2

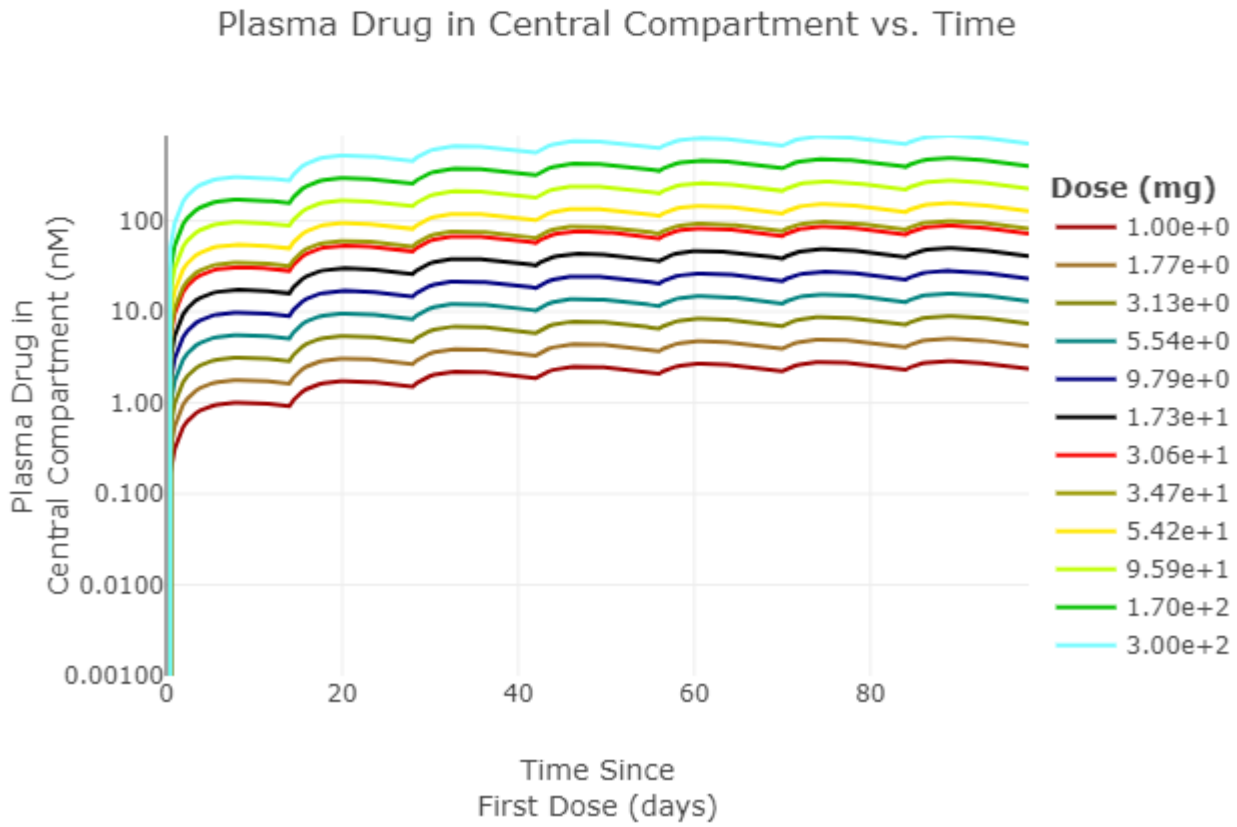

2.17) Scenario: rec_halflife_low

One dimensional scan over parameter, Dose, between 1 and 300 using log spacing.

2.17.1) Model Parameters

Table 2.17.1

Symbol	Parameter ID	Value	Unit
τ	interval	14	-
D	dose	100	mg
$K_{D,\mathrm{L}}$	mab_kd_1	0.008	nM
N_{doses}	dose_count	7	-
MW	mw_1	148000	Da
$t_{1/2}$	el_half_1	20	days
$t_{1/2,\mathrm{a}}$	abs_half	2.5	days
BW	BW	70	kg
V	volume_central	5	L
$\mathrm{Valency}$	drug_valency_1	1	-
$t_{1/2,\mathrm{L}}$	lig_half_1	30	min
$t_{1/2,\mathrm{R}}$	rec_half_1	180	min

 © Applied BioMath LLC 2017-2021

$t_{1/2,\mathrm{sR}}$	shed_half_1	0.5	hr
$K_{D,\mathrm{L:R}}$	lig_rec_kd_1	0.019	nM
$C_{SS,\mathrm{L}}$	lig_css_1_central	0.0000575	nM
$C_{SS,\mathrm{R}}$	rec_css_1_central	0.23	nM
$C_{SS,\mathrm{sR}}$	shed_css_1_central	0	nM
$\mathrm{Density}_{\mathrm{cells}}$	cell_density_mL_central	1000000	#/mL

Parameter ID	Value
Scan Parameter 1	Dose
Lower Limit 1	1
Upper Limit 1	300
N1	11
Scale 1	log

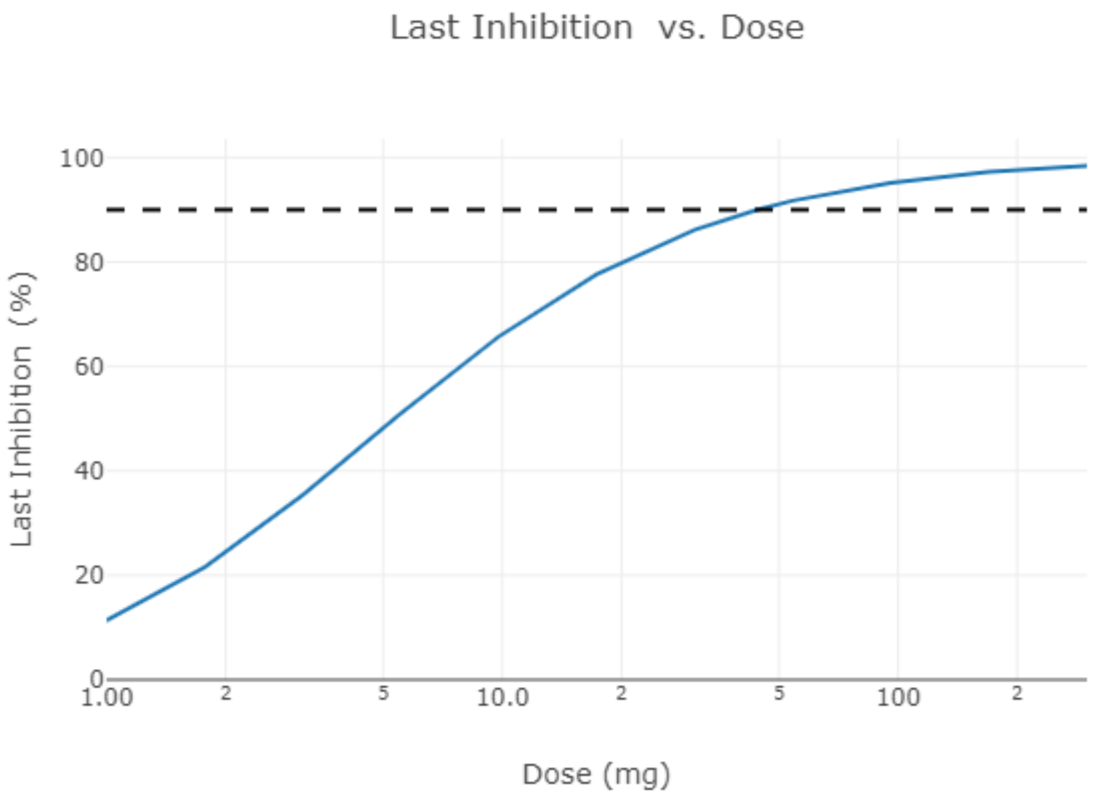

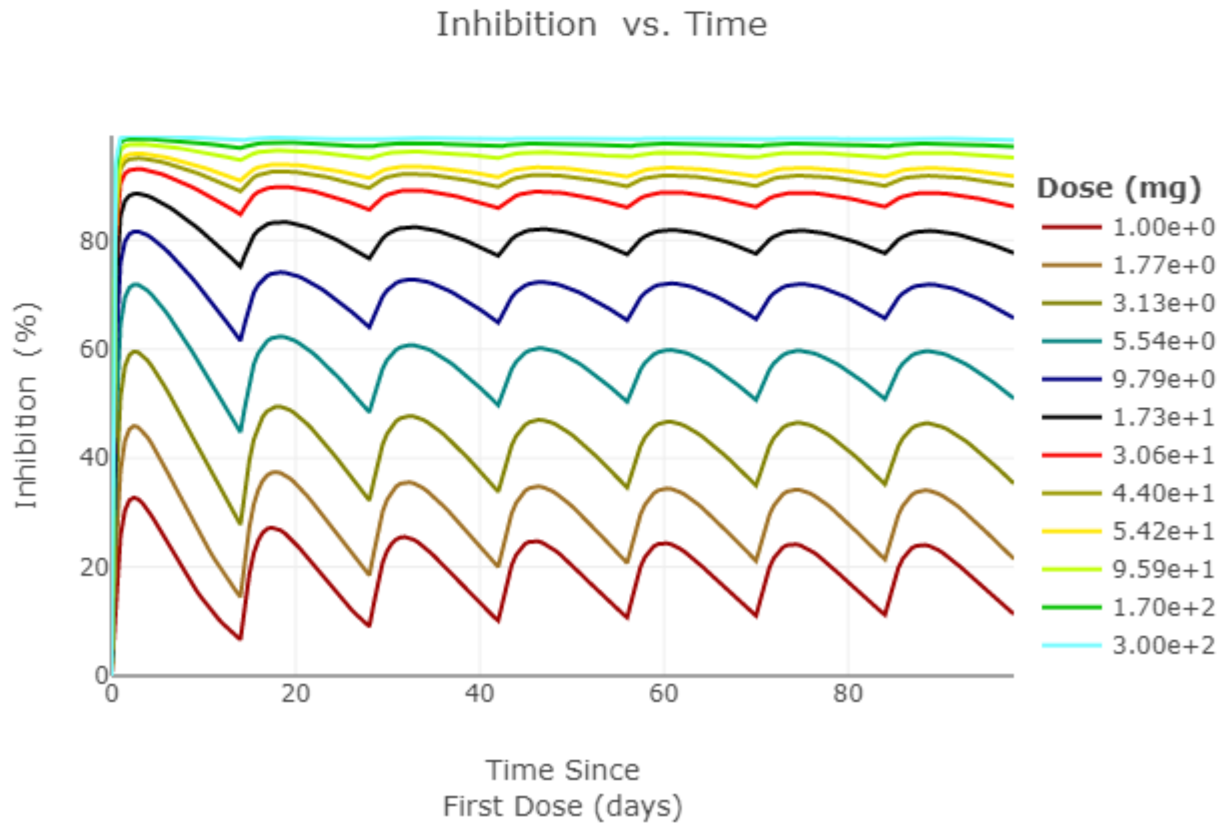

Inhibition vs. Time
Inhibition (%)
0
20
40
60
80
0
20
40
60
80
Time Since
First Dose (days)
Dose (mg)
1.00e+0
1.77e+0
3.13e+0
5.54e+0
9.79e+0
1.73e+1
3.06e+1
4.40e+1
5.42e+1
9.59e+1
1.70e+2
3.00e+2

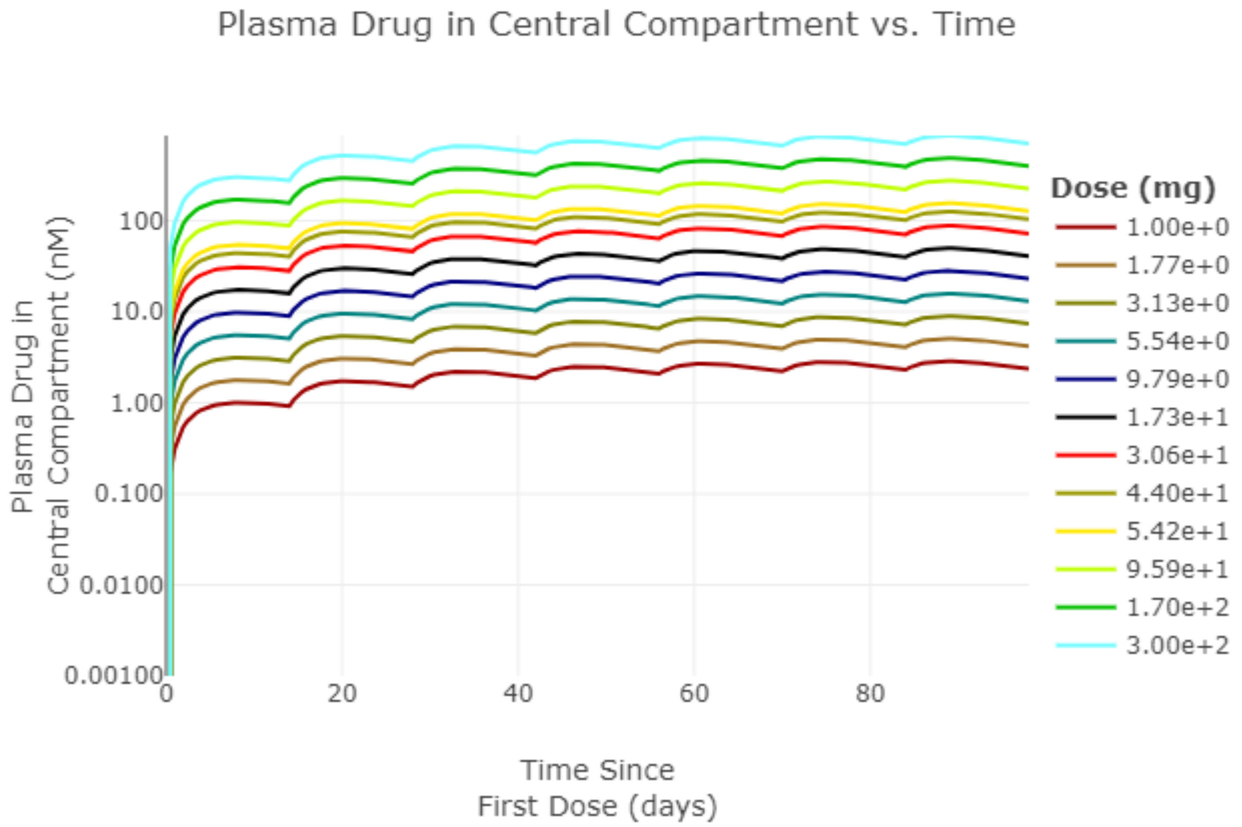

Plasma Drug in Central Compartment vs. Time
Plasma Drug in
Central Compartment (nM)
0.00100
0.0100
0.100
1.00
10.0
100
0
20
40
60
80
Time Since
First Dose (days)
Dose (mg)
1.00e+0
1.77e+0
3.13e+0
5.54e+0
9.79e+0
1.73e+1
3.06e+1
4.40e+1
5.42e+1
9.59e+1
1.70e+2
3.00e+2

2.18) Scenario: Volume_high

One dimensional scan over parameter, Dose, between 1 and 300 using log spacing.

2.18.1) Model Parameters

Table 2.18.1

Symbol	Parameter ID	Value	Unit
τ	interval	14	-
D	dose	100	mg
$K_{D,\mathrm{L}}$	mab_kd_1	0.008	nM
N_{doses}	dose_count	7	-
MW	mw_1	148000	Da
$t_{1/2}$	el_half_1	20	days
$t_{1/2,\mathrm{a}}$	abs_half	2.5	days
BW	BW	70	kg
V	volume_central	15	L
$\mathrm{Valency}$	drug_valency_1	1	-
$t_{1/2,\mathrm{L}}$	lig_half_1	30	min
$t_{1/2,\mathrm{R}}$	rec_half_1	540	min
$t_{1/2,\mathrm{sR}}$	shed_half_1	0.5	hr
$K_{D,\mathrm{L:R}}$	lig_rec_kd_1	0.019	nM
$C_{ss,\mathrm{L}}$	lig_css_1_central	0.0000575	nM
$C_{ss,\mathrm{R}}$	rec_css_1_central	0.23	nM
$C_{ss,\mathrm{sR}}$	shed_css_1_central	0	nM
$\mathrm{Density}_{\mathrm{cells}}$	cell_density_mL_central	1000000	#/mL

Parameter ID	Value
Scan Parameter 1	Dose
Lower Limit 1	1
Upper Limit 1	300
N1	11
Scale 1	log

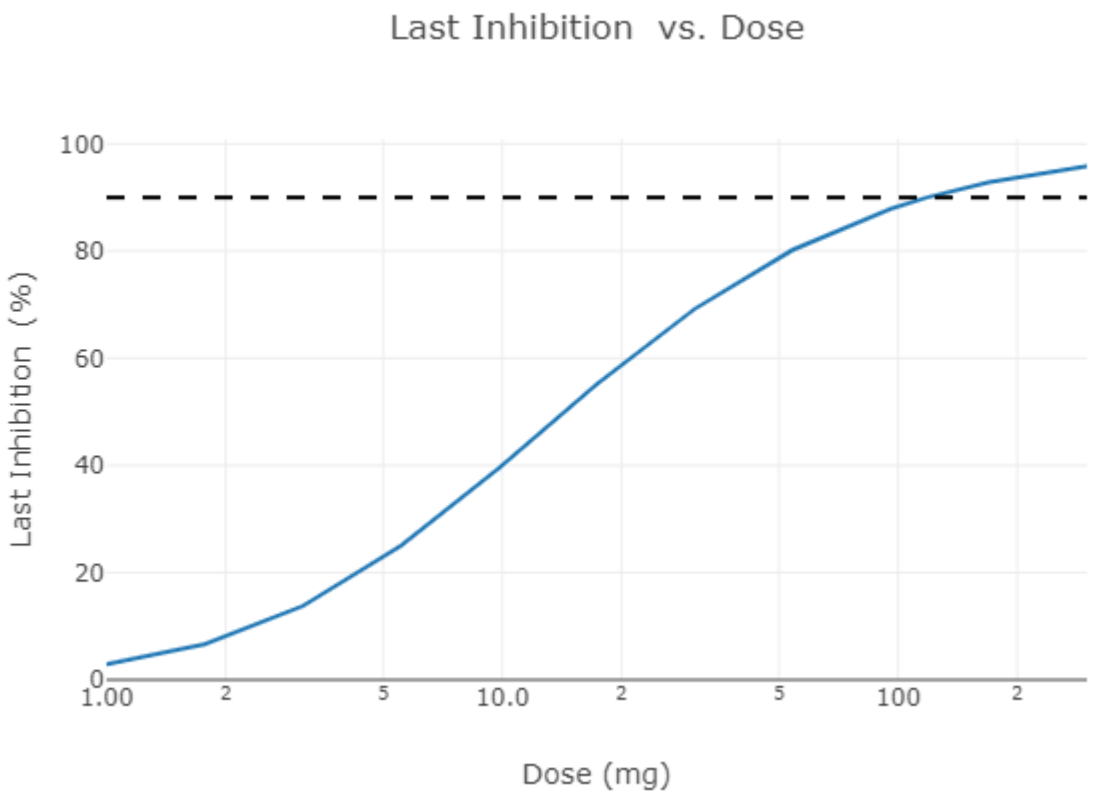

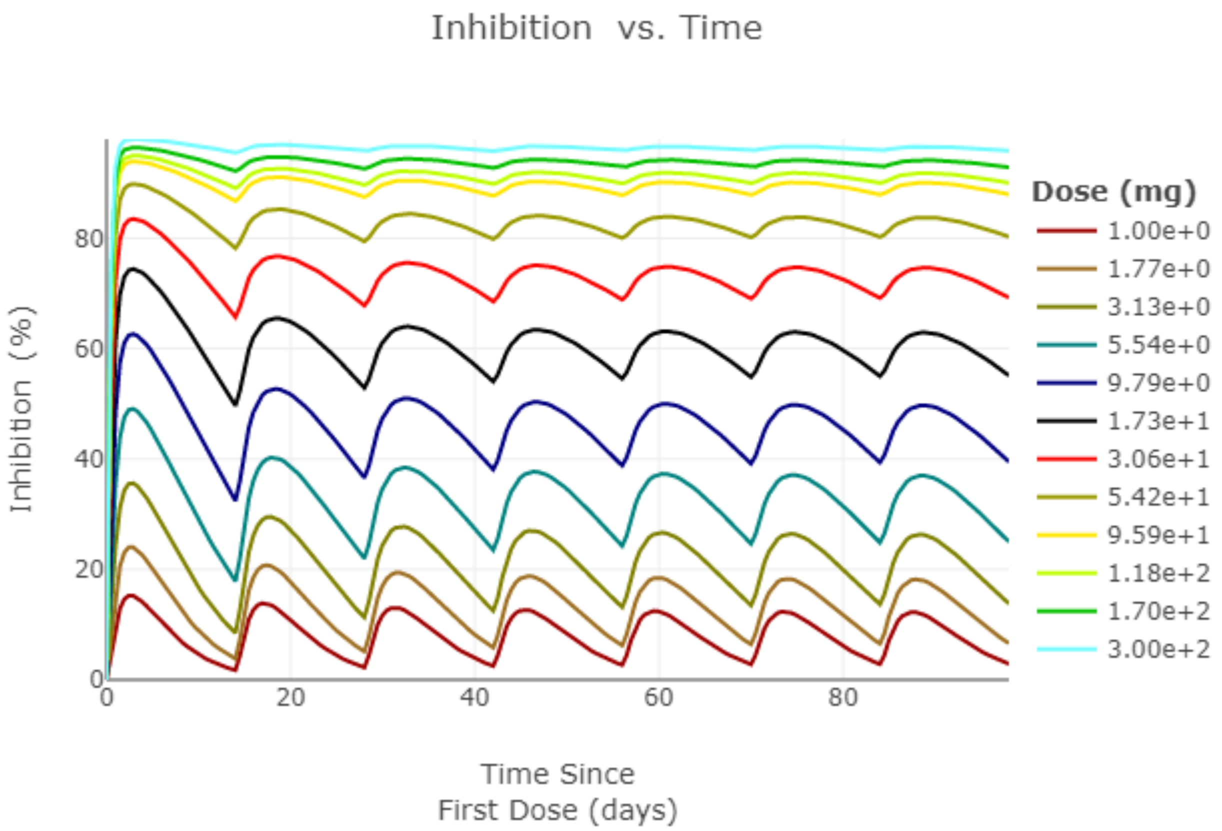

 © Applied BioMath LLC 2017-2021

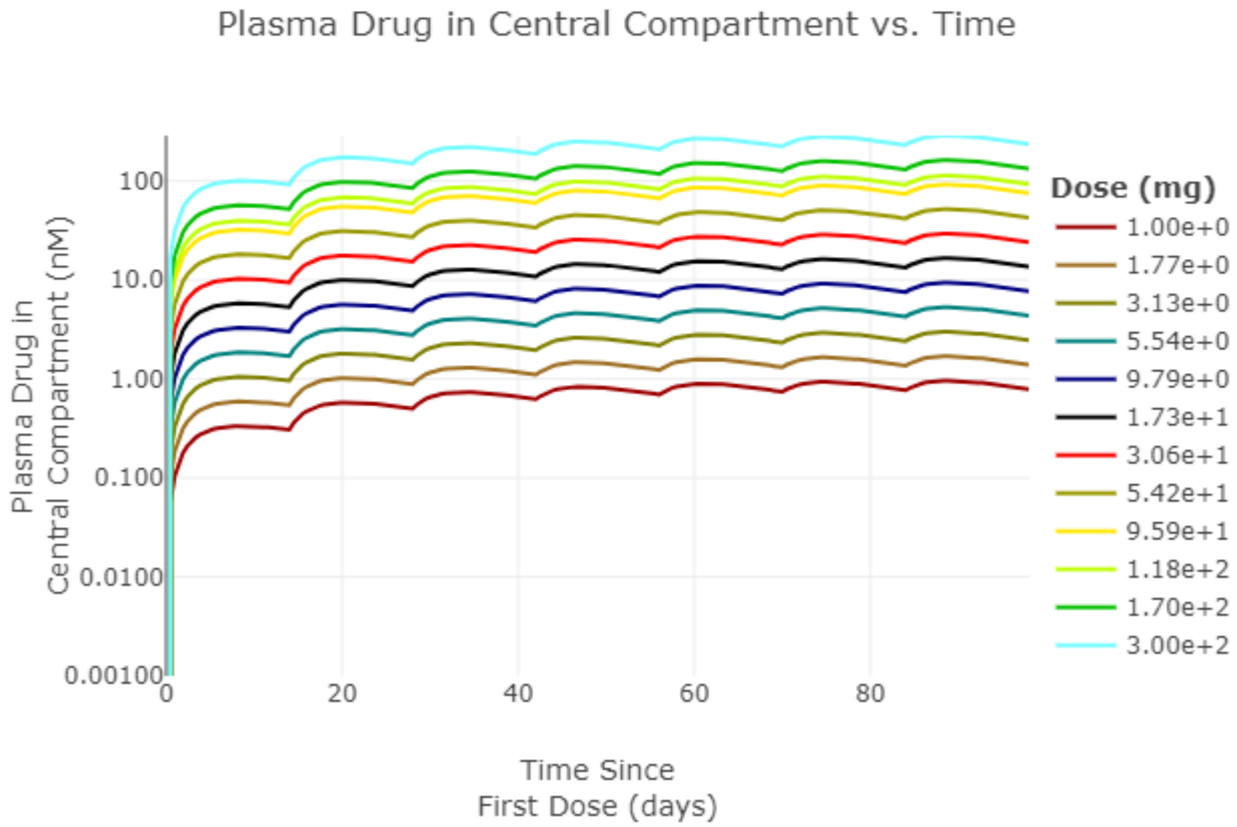

2.19) Scenario: Volume_low

One dimensional scan over parameter, Dose, between 1 and 300 using log spacing.

2.19.1) Model Parameters

Table 2.19.1

Symbol	Parameter ID	Value	Unit
τ	interval	14	-
D	dose	100	mg
$K_{D,\mathrm{L}}$	mab_kd_1	0.008	nM
N_{doses}	dose_count	7	-
MW	mw_1	148000	Da
$t_{1/2}$	el_half_1	20	days
$t_{1/2,\mathrm{a}}$	abs_half	2.5	days
BW	BW	70	kg
V	volume_central	1.67	L
Valency	drug_valency_1	1	-
$t_{1/2,\mathrm{L}}$	lig_half_1	30	min
$t_{1/2,\mathrm{R}}$	rec_half_1	540	min

$t_{1/2,\mathrm{sR}}$	shed_half_1	0.5	hr
$K_{D,\mathrm{L:R}}$	lig_rec_kd_1	0.019	nM
$C_{SS,\mathrm{L}}$	lig_css_1_central	0.0000575	nM
$C_{SS,\mathrm{R}}$	rec_css_1_central	0.23	nM
$C_{SS,\mathrm{sR}}$	shed_css_1_central	0	nM
$\mathrm{Density}_{\mathrm{cells}}$	cell_density_mL_central	1000000	#/mL

Parameter ID	Value
Scan Parameter 1	Dose
Lower Limit 1	1
Upper Limit 1	300
N1	11
Scale 1	log

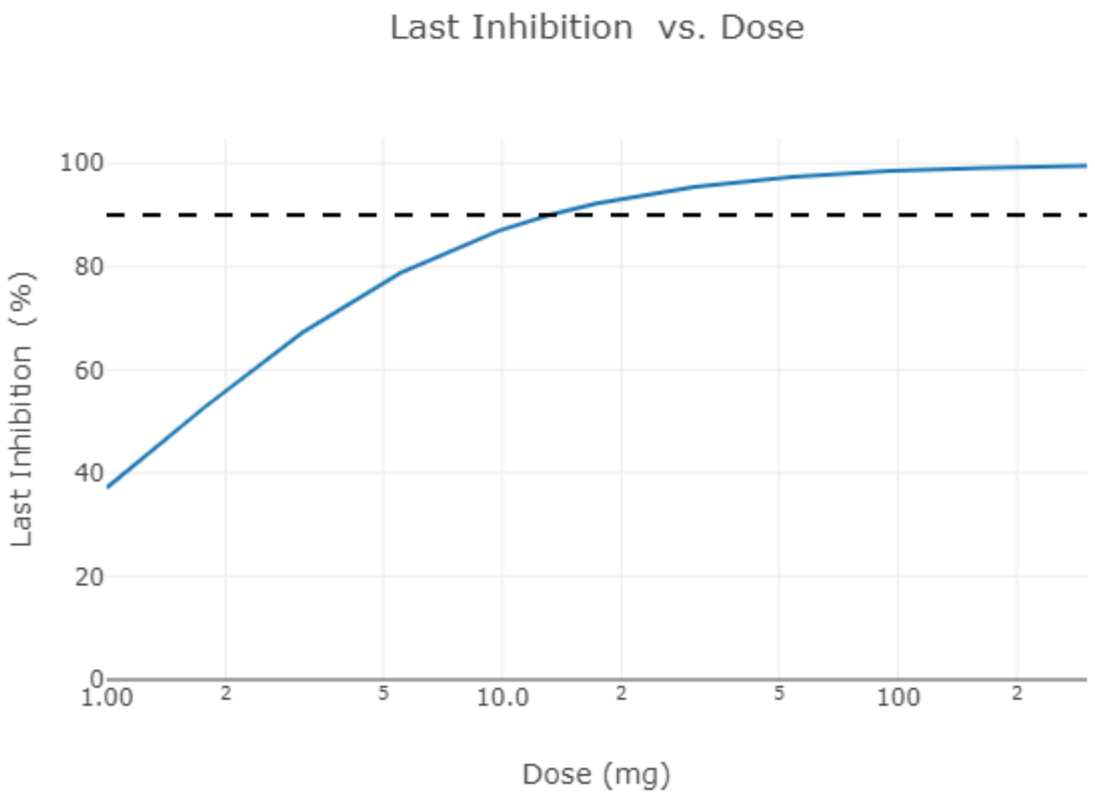

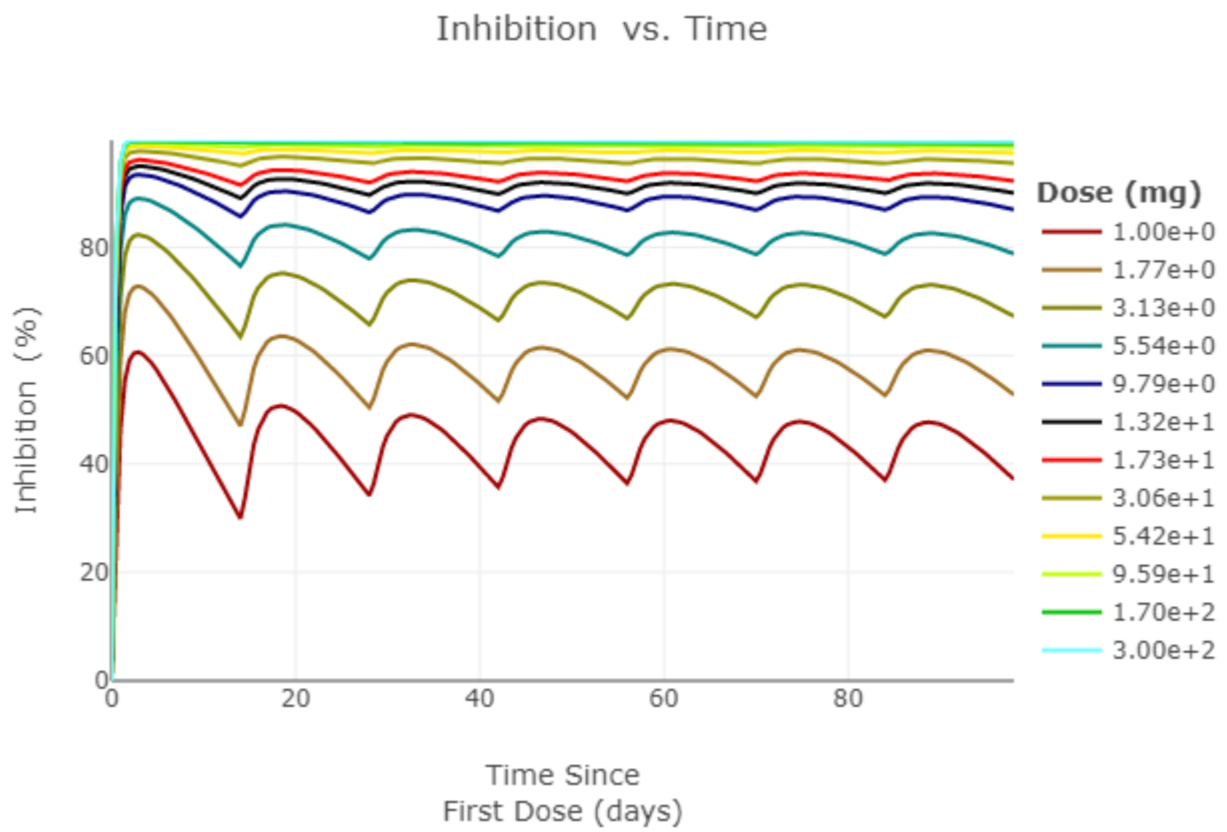
Inhibition vs. Time
Inhibition (%)
0
20
40
60
80
0
20
40
60
80
Time Since
First Dose (days)
Dose (mg)
1.00e+0
1.77e+0
3.13e+0
5.54e+0
9.79e+0
1.32e+1
1.73e+1
3.06e+1
5.42e+1
9.59e+1
1.70e+2
3.00e+2

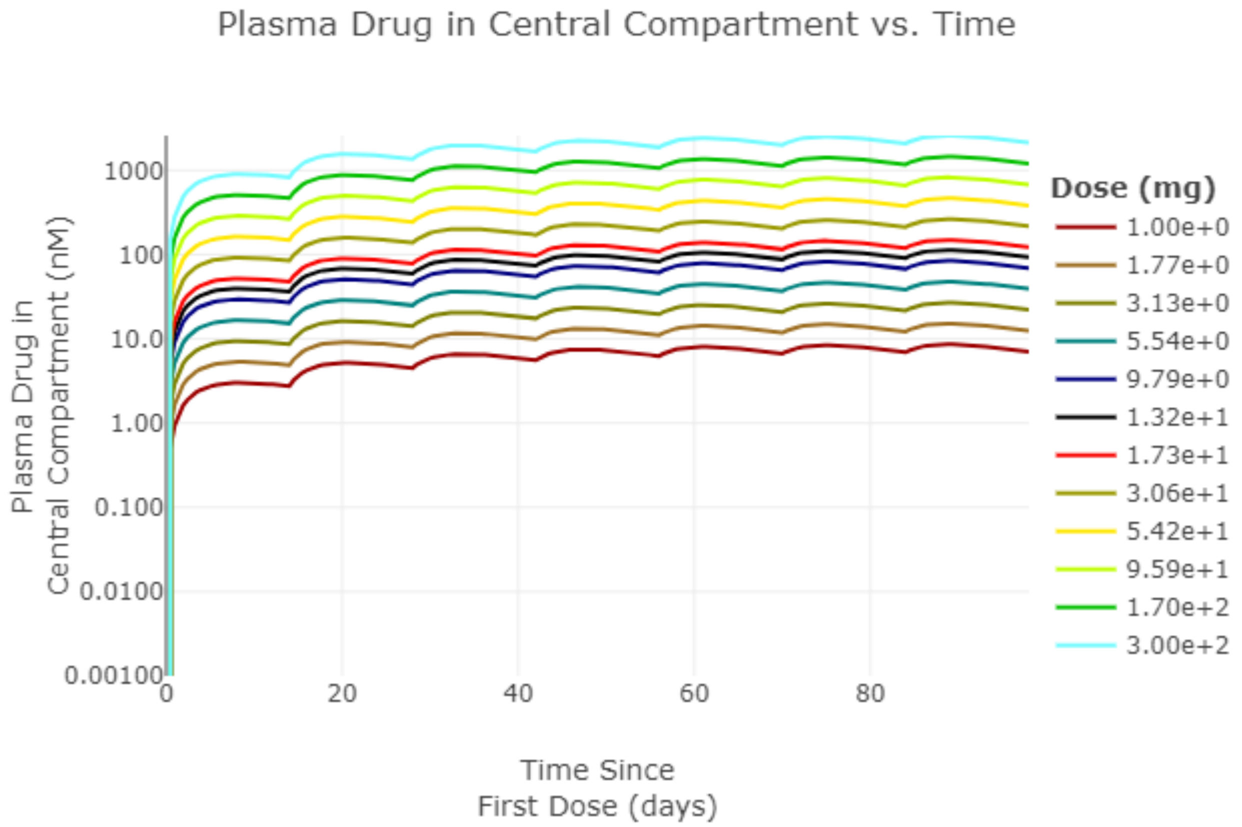
Plasma Drug in Central Compartment vs. Time
Plasma Drug in
Central Compartment (nM)
1000
100
10.0
1.00
0.100
0.0100
0.00100
0
20
40
60
80
Time Since
First Dose (days)
Dose (mg)
1.00e+0
1.77e+0
3.13e+0
5.54e+0
9.79e+0
1.32e+1
1.73e+1
3.06e+1
5.42e+1
9.59e+1
1.70e+2
3.00e+2
