# Supplementary material for: Early Feasibility Assessment: A Method for Accurately Predicting Biotherapeutic Dosing to Inform Early Drug Discovery Decisions
Source: Front Pharmacol. 2022 Jun 8;13:864768. doi: 10.3389/fphar.2022.864768 (PMC9214263; doi:10.3389/fphar.2022.864768)
Supplement: Supplementary file 3 [file DataSheet2.ZIP › Model run files_json and reports/Amivantamab_CaseStudy.pdf]

revolutionizing drug invention

Amivantamab Case Study
generated with
Applied BioMath Assess™

Model: Bispecific Anti-Receptor x Anti-Receptor (4-Compartment)

Date: 2022-03-29T19:27:12.670Z

Software Version: 2022.3.2

This report does not fall within the scope of the United States Food and Drug Administration Good Laboratory Practice or Good Clinical Practice Regulations.

1) Summary

The goal of this study is to determine the feasibility of a drug concept for a target by varying parameters of a Quantitative Systems Pharmacology (QSP) model. By performing simulations over a range of drug and target parameters, this study supports comparison of the effects of dose amount, dose frequency, route of administration, drug design criteria, and target characteristics. This assessment can aid in the prediction of difficulty of discovering and formulation a New Biological Entity that satisfies a Target Product Profile.

The analyses explore the model conditions under which one or more criteria are satisfied. Depending on the kind of analysis, the feasibility of a scenario is defined to be either:

- the dose that exactly satisfies the criteria targets,
- a range of a parameter that satisfies the criteria targets,
- or a region of a parameter grid that satisfies the criteria targets.

The model used in these analyses is: Bispecific Anti-Receptor x Anti-Receptor (4-Compartment) - A bispecific biotherapeutic that binds to two target cell surface receptors on a single cell type. For each receptor the drug either (1) acts as a competitive inhibitor by blocking the cognate-ligand from binding to its receptor or (2) acts as a receptor agonist. The molecule can be mono- or bivalent for each target. This is a four-compartment model with a central, peripheral, disease and tox compartments. There is an option for +/- soluble receptor for each receptor.

 © Applied BioMath LLC 2017-2021

Scenario Table

Description	Route	Interval (undefined)	Dose (mg)	Scan Parameter	Criterion	Result
EGFR Q2W	IV	14	-	: Dose (mg) [140 - 1400]	Last Target Engagement R1 Peripheral ≥ 98 %	Dose ≥ 740 mg
c-Met Q2W	IV	14	-	: Dose (mg) [140 - 1400]	Last Target Engagement R2 Peripheral ≥ 98 %	Dose ≥ 306 mg
EGFR Q1W	IV	7	-	: Dose (mg) [10 - 1000]	Last Target Engagement R1 Peripheral ≥ 98 %	Dose ≥ 326 mg
c-Met Q1W	IV	7	-	: Dose (mg) [10 - 1000]	Last Target Engagement R2 Peripheral ≥ 98 %	Dose ≥ 120 mg

© Applied BioMath LLC 2017-2021

2) Results

2.0) Scenario: EGFR Q2W

One dimensional scan over parameter, Dose, between 140 and 1400 using log spacing.

2.0.1) Model Parameters

Table 2.0.1

Symbol	Parameter ID	Value	Unit
τ	interval	14	-
D	dose	100	mg
K_{D,R_1}	mab_kd_1	1.4	nM
K_{D,R_2}	mab_kd_2	0.04	nM
N_{doses}	dose_count	4	-
MW	mw_1	150000	Da
$t_{1/2}$	el_half_1	11	days
$t_{1/2,\mathrm{a}}$	abs_half	2.5	days
BW	BW	70	kg
V	volume_central	3	L
$V_{\mathrm{peripheral}}$	volume_peripheral	13	L
V_{disease}	volume_disease	0.1	L
V_{tox}	volume_tox	0.1	L
$T_{\mathrm{dist,peripheral}}$	Tdist_Ab_hr_peripheral	35	hr
$T_{\mathrm{dist,disease}}$	Tdist_Ab_hr_disease	30	hr
$T_{\mathrm{dist,tox}}$	Tdist_Ab_hr_tox	30	hr
$P_{\mathrm{dist,peripheral}}$	Pdist_Ab_peripheral	0.190625	-
$P_{\mathrm{dist,disease}}$	Pdist_Ab_disease	0	-
$P_{\mathrm{dist,tox}}$	Pdist_Ab_tox	0	-
$\mathrm{Valency}_1$	drug_valency_1	1	-
$\mathrm{Valency}_2$	drug_valency_2	1	-
$t_{1/2,\mathrm{L}_1}$	lig_half_1	1000	hr
$t_{1/2,\mathrm{L}_2}$	lig_half_2	1000	hr
$t_{1/2,\mathrm{R}_1}$	rec_half_1	5	hr
$t_{1/2,\mathrm{R}_2}$	rec_half_2	4	hr

 © Applied BioMath LLC 2017-2021

$t_{1/2,\mathrm{sR}_1}$	shed_half_1	0.5	hr
$t_{1/2,\mathrm{sR}_2}$	shed_half_2	48	hr
$K_{D,\mathrm{L}_1:\mathrm{R}_1}$	lig_rec_kd_1	1	nM
$K_{D,\mathrm{L}_2:\mathrm{R}_2}$	lig_rec_kd_2	1	nM
$C_{SS,\mathrm{L}_1,\mathrm{central}}$	lig_css_1_central	0.00001	nM
$C_{SS,\mathrm{L}_2,\mathrm{central}}$	lig_css_2_central	0.00001	nM
$C_{SS,\mathrm{L}_1,\mathrm{peripheral}}$	lig_css_1_peripheral	0	nM
$C_{SS,\mathrm{L}_2,\mathrm{peripheral}}$	lig_css_2_peripheral	0	nM
$C_{SS,\mathrm{L}_1,\mathrm{disease}}$	lig_css_1_disease	0.05	nM
$C_{SS,\mathrm{L}_2,\mathrm{disease}}$	lig_css_2_disease	0.05	nM
$C_{SS,\mathrm{L}_1,\mathrm{tox}}$	lig_css_1_tox	0.05	nM
$C_{SS,\mathrm{L}_2,\mathrm{tox}}$	lig_css_2_tox	0.05	nM
$C_{SS,\mathrm{R}_1,\mathrm{central}}$	rec_css_1_central	0.0152	nM
$C_{SS,\mathrm{R}_2,\mathrm{central}}$	rec_css_2_central	0.011	nM
$C_{SS,\mathrm{R}_1,\mathrm{peripheral}}$	rec_css_1_peripheral	1.13	nM
$C_{SS,\mathrm{R}_2,\mathrm{peripheral}}$	rec_css_2_peripheral	0.45	nM
$C_{SS,\mathrm{R}_1,\mathrm{disease}}$	rec_css_1_disease	10000	#/cell
$C_{SS,\mathrm{R}_2,\mathrm{disease}}$	rec_css_2_disease	10000	#/cell
$C_{SS,\mathrm{R}_1,\mathrm{tox}}$	rec_css_1_tox	10000	#/cell
$C_{SS,\mathrm{R}_2,\mathrm{tox}}$	rec_css_2_tox	10000	#/cell
$C_{SS,\mathrm{sR}_1,\mathrm{central}}$	shed_css_1_central	0	nM
$C_{SS,\mathrm{sR}_2,\mathrm{central}}$	shed_css_2_central	5.9	nM
$C_{SS,\mathrm{sR}_1,\mathrm{peripheral}}$	shed_css_1_peripheral	0	nM
$C_{SS,\mathrm{sR}_2,\mathrm{peripheral}}$	shed_css_2_peripheral	5.9	nM
$C_{SS,\mathrm{sR}_1,\mathrm{disease}}$	shed_css_1_disease	0	nM
$C_{SS,\mathrm{sR}_2,\mathrm{disease}}$	shed_css_2_disease	0	nM
$C_{SS,\mathrm{sR}_1,\mathrm{tox}}$	shed_css_1_tox	0	nM
$C_{SS,\mathrm{sR}_2,\mathrm{tox}}$	shed_css_2_tox	0	nM
$T_{\mathrm{dist},\mathrm{L}_1,\mathrm{peripheral}}$	Tdist_L1_hr_peripheral	30	hr
$T_{\mathrm{dist},\mathrm{L}_2,\mathrm{peripheral}}$	Tdist_L2_hr_peripheral	30	hr
$T_{\mathrm{dist},\mathrm{L}_1,\mathrm{disease}}$	Tdist_L1_hr_disease	30	hr
$T_{\mathrm{dist},\mathrm{L}_2,\mathrm{disease}}$	Tdist_L2_hr_disease	30	hr
$T_{\mathrm{dist},\mathrm{L}_1,\mathrm{tox}}$	Tdist_L1_hr_tox	30	hr
$T_{\mathrm{dist},\mathrm{L}_2,\mathrm{tox}}$	Tdist_L2_hr_tox	30	hr
$T_{\mathrm{dist},\mathrm{sR}_1,\mathrm{peripheral}}$	Tdist_S1_hr_peripheral	30	hr

$T_{\mathrm{dist,sR_2,peripheral}}$	Tdist_S2_hr_peripheral	30	hr
$T_{\mathrm{dist,sR_1,disease}}$	Tdist_S1_hr_disease	30	hr
$T_{\mathrm{dist,sR_2,disease}}$	Tdist_S2_hr_disease	30	hr
$T_{\mathrm{dist,sR_1,tox}}$	Tdist_S1_hr_tox	30	hr
$T_{\mathrm{dist,sR_2,tox}}$	Tdist_S2_hr_tox	30	hr
$\mathrm{Density}_{\mathrm{cells,central}}$	cell_density_mL_central	1000000	#/mL
$\mathrm{Density}_{\mathrm{cells,peripheral}}$	cell_density_mL_peripheral	1000000	#/mL
$\mathrm{Density}_{\mathrm{cells,disease}}$	cell_density_mL_disease	1000000	#/mL
$\mathrm{Density}_{\mathrm{cells,tox}}$	cell_density_mL_tox	1000000	#/mL
$\mathrm{Scale}_{t_{1/2,\mathrm{R_1}},\mathrm{D:R_1,central}}$	scale_half_Ab_R1_central	1	-
$\mathrm{Scale}_{t_{1/2,\mathrm{R_2}},\mathrm{D:R_2,central}}$	scale_half_Ab_R2_central	1	-
$\mathrm{Scale}_{t_{1/2,\mathrm{R_1}},\mathrm{D:R_1,peripheral}}$	scale_half_Ab_R1_peripheral	1	-
$\mathrm{Scale}_{t_{1/2,\mathrm{R_2}},\mathrm{D:R_2,peripheral}}$	scale_half_Ab_R2_peripheral	1	-
$\mathrm{Scale}_{t_{1/2,\mathrm{R_1}},\mathrm{D:R_1,disease}}$	scale_half_Ab_R1_disease	1	-
$\mathrm{Scale}_{t_{1/2,\mathrm{R_2}},\mathrm{D:R_2,disease}}$	scale_half_Ab_R2_disease	1	-
$\mathrm{Scale}_{t_{1/2,\mathrm{R_1}},\mathrm{D:R_1,tox}}$	scale_half_Ab_R1_tox	1	-
$\mathrm{Scale}_{t_{1/2,\mathrm{R_2}},\mathrm{D:R_2,tox}}$	scale_half_Ab_R2_tox	1	-
$\mathrm{Scale}_{K_{D,\mathrm{R_1}},\mathrm{central}}$	scale_kd_Ab_T1_central	1	-
$\mathrm{Scale}_{K_{D,\mathrm{R_2}},\mathrm{central}}$	scale_kd_Ab_T2_central	1	-
$\mathrm{Scale}_{K_{D,\mathrm{R_1}},\mathrm{peripheral}}$	scale_kd_Ab_T1_peripheral	1	-
$\mathrm{Scale}_{K_{D,\mathrm{R_2}},\mathrm{peripheral}}$	scale_kd_Ab_T2_peripheral	1	-
$\mathrm{Scale}_{K_{D,\mathrm{R_1}},\mathrm{disease}}$	scale_kd_Ab_T1_disease	1	-
$\mathrm{Scale}_{K_{D,\mathrm{R_2}},\mathrm{disease}}$	scale_kd_Ab_T2_disease	1	-
$\mathrm{Scale}_{K_{D,\mathrm{R_1}},\mathrm{tox}}$	scale_kd_Ab_T1_tox	1	-
$\mathrm{Scale}_{K_{D,\mathrm{R_2}},\mathrm{tox}}$	scale_kd_Ab_T2_tox	1	-
$\mathrm{Scale}_{t_{1/2},\mathrm{central}}$	scale_half_Ab_central	1	-
$\mathrm{Scale}_{t_{1/2},\mathrm{peripheral}}$	scale_half_Ab_peripheral	1	-
$\mathrm{Scale}_{t_{1/2},\mathrm{disease}}$	scale_half_Ab_disease	1	-
$\mathrm{Scale}_{t_{1/2},\mathrm{tox}}$	scale_half_Ab_tox	1	-

Parameter ID	Value
Scan Parameter 1	Dose
Lower Limit 1	140
Upper Limit 1	1400
N1	11

© Applied BioMath LLC 2017-2021

Scale 1	log

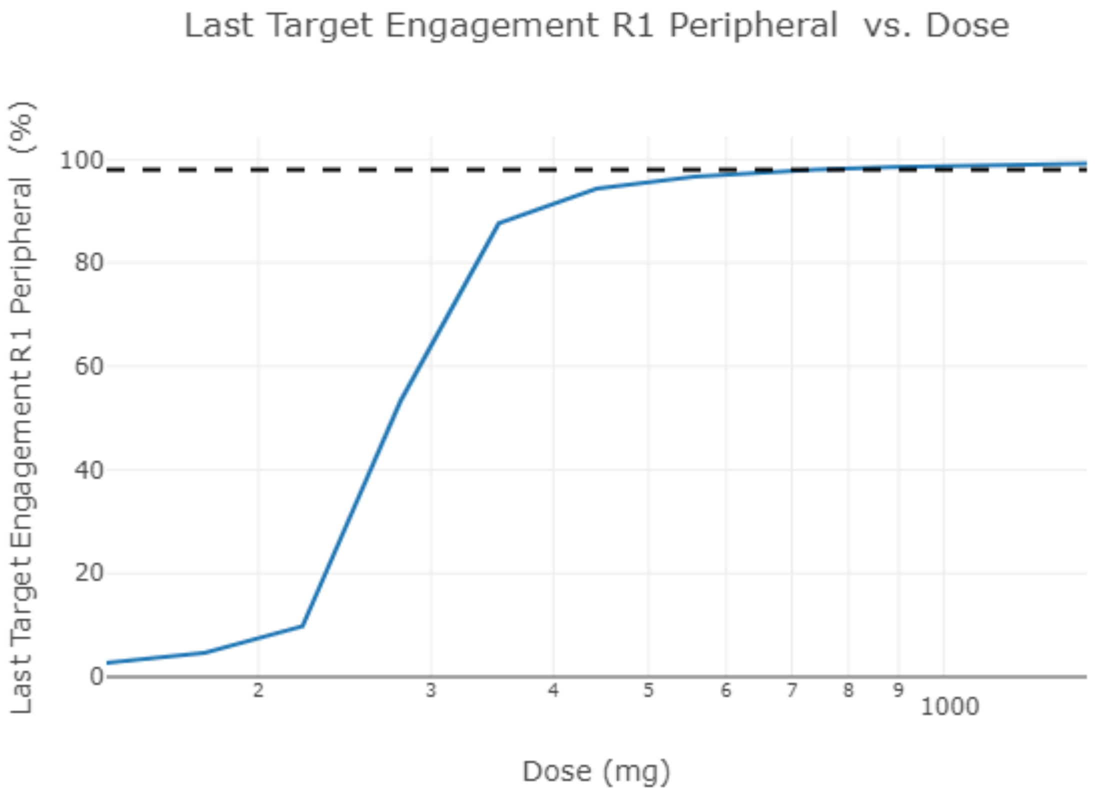

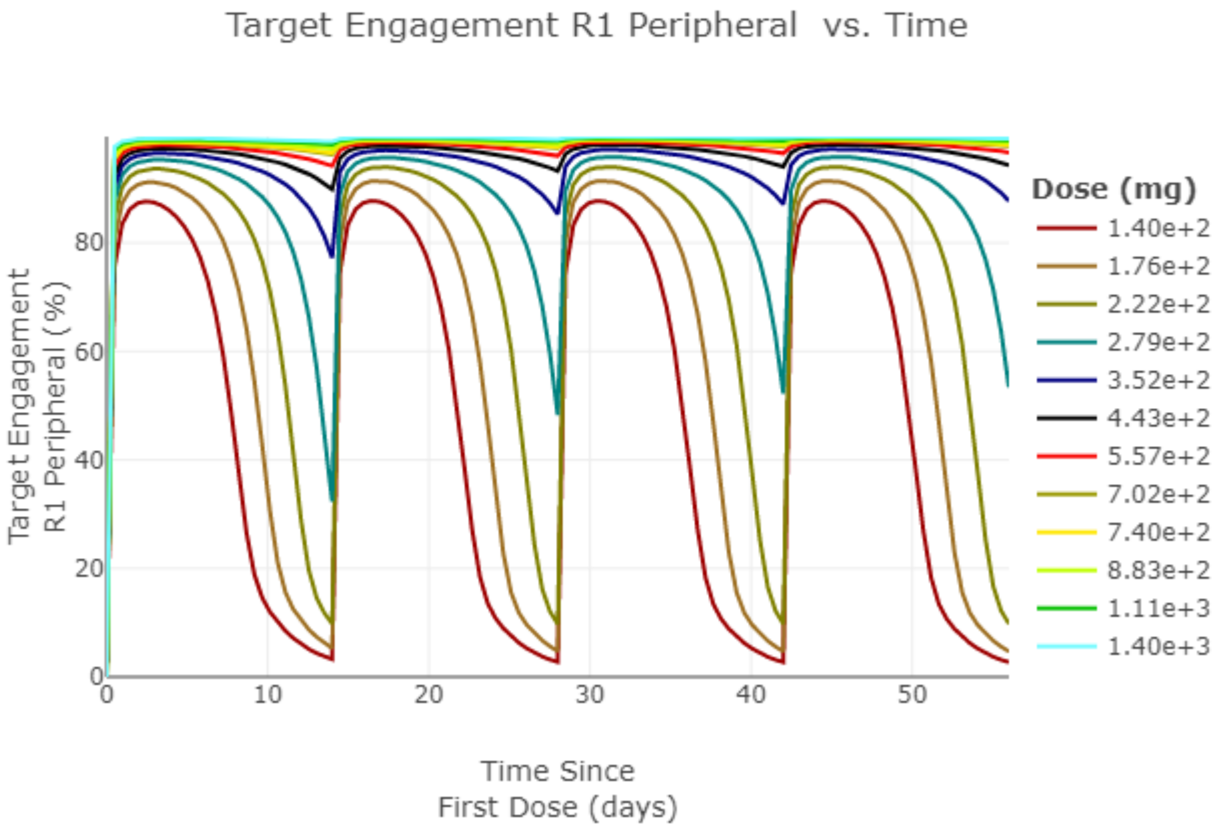

 © Applied BioMath LLC 2017-2021

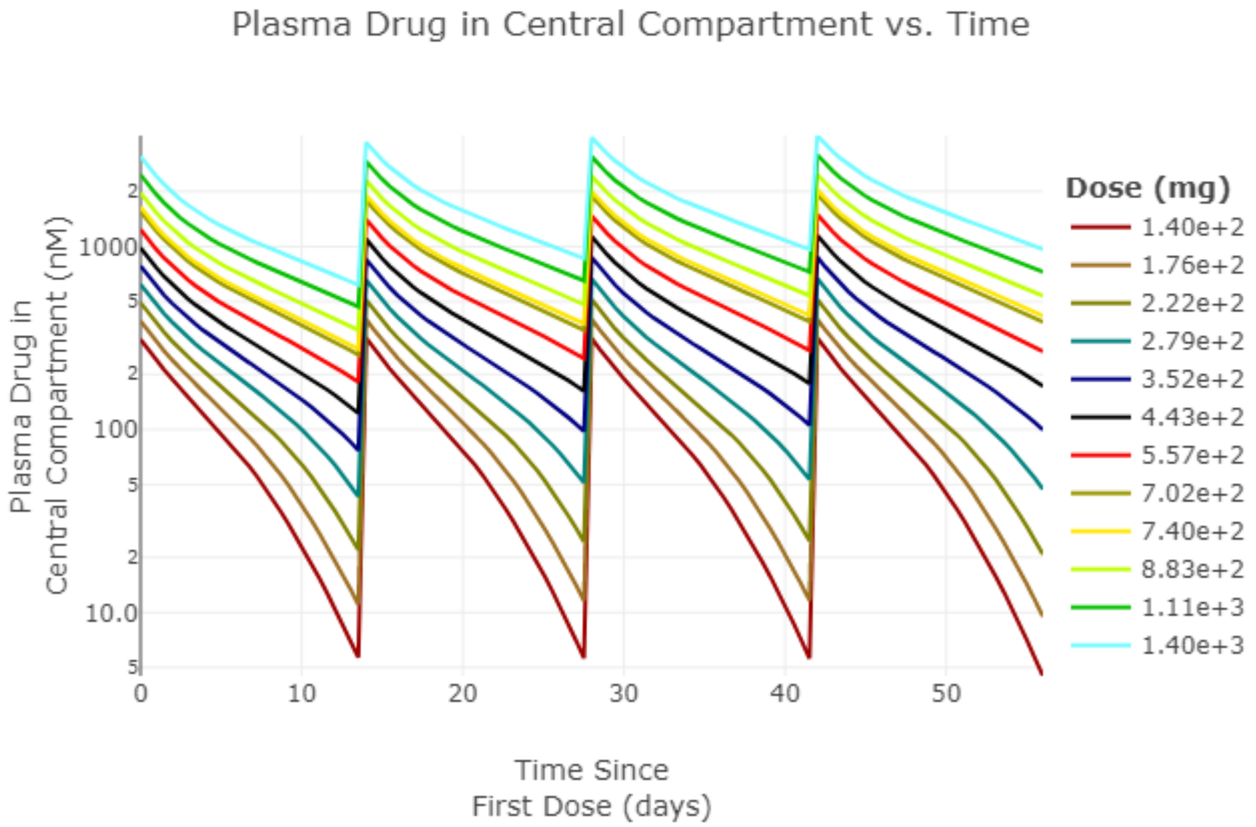

2.1) Scenario: c-Met Q2W

One dimensional scan over parameter, Dose, between 140 and 1400 using log spacing.

2.1.1) Model Parameters

Table 2.1.1

Symbol	Parameter ID	Value	Unit
τ	interval	14	-
D	dose	100	mg
K_{D,R_1}	mab_kd_1	1.4	nM
K_{D,R_2}	mab_kd_2	0.04	nM
N_{doses}	dose_count	4	-
MW	mw_1	150000	Da
$t_{1/2}$	el_half_1	11	days
$t_{1/2,\mathrm{a}}$	abs_half	2.5	days
BW	BW	70	kg
V	volume_central	3	L
$V_{\mathrm{peripheral}}$	volume_peripheral	13	L
V_{disease}	volume_disease	0.1	L

 © Applied BioMath LLC 2017-2021

V_{tox}	volume_tox	0.1	L
$T_{\mathrm{dist,peripheral}}$	Tdist_Ab_hr_peripheral	35	hr
$T_{\mathrm{dist,disease}}$	Tdist_Ab_hr_disease	30	hr
$T_{\mathrm{dist,tox}}$	Tdist_Ab_hr_tox	30	hr
$P_{\mathrm{dist,peripheral}}$	Pdist_Ab_peripheral	0.190625	-
$P_{\mathrm{dist,disease}}$	Pdist_Ab_disease	0	-
$P_{\mathrm{dist,tox}}$	Pdist_Ab_tox	0	-
$\mathrm{Valency}_1$	drug_valency_1	1	-
$\mathrm{Valency}_2$	drug_valency_2	1	-
$t_{1/2,\mathrm{L}_1}$	lig_half_1	1000	hr
$t_{1/2,\mathrm{L}_2}$	lig_half_2	1000	hr
$t_{1/2,\mathrm{R}_1}$	rec_half_1	5	hr
$t_{1/2,\mathrm{R}_2}$	rec_half_2	4	hr
$t_{1/2,\mathrm{sR}_1}$	shed_half_1	0.5	hr
$t_{1/2,\mathrm{sR}_2}$	shed_half_2	48	hr
$K_{D,\mathrm{L}_1:\mathrm{R}_1}$	lig_rec_kd_1	1	nM
$K_{D,\mathrm{L}_2:\mathrm{R}_2}$	lig_rec_kd_2	1	nM
$C_{SS,\mathrm{L}_1,\mathrm{central}}$	lig_css_1_central	0.00001	nM
$C_{SS,\mathrm{L}_2,\mathrm{central}}$	lig_css_2_central	0.00001	nM
$C_{SS,\mathrm{L}_1,\mathrm{peripheral}}$	lig_css_1_peripheral	0	nM
$C_{SS,\mathrm{L}_2,\mathrm{peripheral}}$	lig_css_2_peripheral	0	nM
$C_{SS,\mathrm{L}_1,\mathrm{disease}}$	lig_css_1_disease	0.05	nM
$C_{SS,\mathrm{L}_2,\mathrm{disease}}$	lig_css_2_disease	0.05	nM
$C_{SS,\mathrm{L}_1,\mathrm{tox}}$	lig_css_1_tox	0.05	nM
$C_{SS,\mathrm{L}_2,\mathrm{tox}}$	lig_css_2_tox	0.05	nM
$C_{SS,\mathrm{R}_1,\mathrm{central}}$	rec_css_1_central	0.0152	nM
$C_{SS,\mathrm{R}_2,\mathrm{central}}$	rec_css_2_central	0.011	nM
$C_{SS,\mathrm{R}_1,\mathrm{peripheral}}$	rec_css_1_peripheral	1.13	nM
$C_{SS,\mathrm{R}_2,\mathrm{peripheral}}$	rec_css_2_peripheral	0.45	nM
$C_{SS,\mathrm{R}_1,\mathrm{disease}}$	rec_css_1_disease	10000	#/cell
$C_{SS,\mathrm{R}_2,\mathrm{disease}}$	rec_css_2_disease	10000	#/cell
$C_{SS,\mathrm{R}_1,\mathrm{tox}}$	rec_css_1_tox	10000	#/cell
$C_{SS,\mathrm{R}_2,\mathrm{tox}}$	rec_css_2_tox	10000	#/cell
$C_{SS,\mathrm{sR}_1,\mathrm{central}}$	shed_css_1_central	0	nM
$C_{SS,\mathrm{sR}_2,\mathrm{central}}$	shed_css_2_central	5.9	nM

 © Applied BioMath LLC 2017-2021

$C_{SS,\mathrm{sR}_1,\mathrm{peripheral}}$	shed_css_1_peripheral	0	nM
$C_{SS,\mathrm{sR}_2,\mathrm{peripheral}}$	shed_css_2_peripheral	5.9	nM
$C_{SS,\mathrm{sR}_1,\mathrm{disease}}$	shed_css_1_disease	0	nM
$C_{SS,\mathrm{sR}_2,\mathrm{disease}}$	shed_css_2_disease	0	nM
$C_{SS,\mathrm{sR}_1,\mathrm{tox}}$	shed_css_1_tox	0	nM
$C_{SS,\mathrm{sR}_2,\mathrm{tox}}$	shed_css_2_tox	0	nM
$T_{\mathrm{dist},\mathrm{L}_1,\mathrm{peripheral}}$	Tdist_L1_hr_peripheral	30	hr
$T_{\mathrm{dist},\mathrm{L}_2,\mathrm{peripheral}}$	Tdist_L2_hr_peripheral	30	hr
$T_{\mathrm{dist},\mathrm{L}_1,\mathrm{disease}}$	Tdist_L1_hr_disease	30	hr
$T_{\mathrm{dist},\mathrm{L}_2,\mathrm{disease}}$	Tdist_L2_hr_disease	30	hr
$T_{\mathrm{dist},\mathrm{L}_1,\mathrm{tox}}$	Tdist_L1_hr_tox	30	hr
$T_{\mathrm{dist},\mathrm{L}_2,\mathrm{tox}}$	Tdist_L2_hr_tox	30	hr
$T_{\mathrm{dist},\mathrm{sR}_1,\mathrm{peripheral}}$	Tdist_S1_hr_peripheral	30	hr
$T_{\mathrm{dist},\mathrm{sR}_2,\mathrm{peripheral}}$	Tdist_S2_hr_peripheral	30	hr
$T_{\mathrm{dist},\mathrm{sR}_1,\mathrm{disease}}$	Tdist_S1_hr_disease	30	hr
$T_{\mathrm{dist},\mathrm{sR}_2,\mathrm{disease}}$	Tdist_S2_hr_disease	30	hr
$T_{\mathrm{dist},\mathrm{sR}_1,\mathrm{tox}}$	Tdist_S1_hr_tox	30	hr
$T_{\mathrm{dist},\mathrm{sR}_2,\mathrm{tox}}$	Tdist_S2_hr_tox	30	hr
$\mathrm{Density}_{\mathrm{cells,central}}$	cell_density_mL_central	1000000	#/mL
$\mathrm{Density}_{\mathrm{cells,peripheral}}$	cell_density_mL_peripheral	1000000	#/mL
$\mathrm{Density}_{\mathrm{cells,disease}}$	cell_density_mL_disease	1000000	#/mL
$\mathrm{Density}_{\mathrm{cells,tox}}$	cell_density_mL_tox	1000000	#/mL
$\mathrm{Scale}_{t_{1/2,\mathrm{R}_1},\mathrm{D:R}_1,\mathrm{central}}$	scale_half_Ab_R1_central	1	-
$\mathrm{Scale}_{t_{1/2,\mathrm{R}_2},\mathrm{D:R}_2,\mathrm{central}}$	scale_half_Ab_R2_central	1	-
$\mathrm{Scale}_{t_{1/2,\mathrm{R}_1},\mathrm{D:R}_1,\mathrm{peripheral}}$	scale_half_Ab_R1_peripheral	1	-
$\mathrm{Scale}_{t_{1/2,\mathrm{R}_2},\mathrm{D:R}_2,\mathrm{peripheral}}$	scale_half_Ab_R2_peripheral	1	-
$\mathrm{Scale}_{t_{1/2,\mathrm{R}_1},\mathrm{D:R}_1,\mathrm{disease}}$	scale_half_Ab_R1_disease	1	-
$\mathrm{Scale}_{t_{1/2,\mathrm{R}_2},\mathrm{D:R}_2,\mathrm{disease}}$	scale_half_Ab_R2_disease	1	-
$\mathrm{Scale}_{t_{1/2,\mathrm{R}_1},\mathrm{D:R}_1,\mathrm{tox}}$	scale_half_Ab_R1_tox	1	-
$\mathrm{Scale}_{t_{1/2,\mathrm{R}_2},\mathrm{D:R}_2,\mathrm{tox}}$	scale_half_Ab_R2_tox	1	-
$\mathrm{Scale}_{K_{D,\mathrm{R}_1},\mathrm{central}}$	scale_kd_Ab_T1_central	1	-
$\mathrm{Scale}_{K_{D,\mathrm{R}_2},\mathrm{central}}$	scale_kd_Ab_T2_central	1	-
$\mathrm{Scale}_{K_{D,\mathrm{R}_1},\mathrm{peripheral}}$	scale_kd_Ab_T1_peripheral	1	-
$\mathrm{Scale}_{K_{D,\mathrm{R}_2},\mathrm{peripheral}}$	scale_kd_Ab_T2_peripheral	1	-

$\mathrm{Scale}_{K_{D,\mathrm{R}_1},\mathrm{disease}}$	scale_kd_Ab_T1_disease	1	-
$\mathrm{Scale}_{K_{D,\mathrm{R}_2},\mathrm{disease}}$	scale_kd_Ab_T2_disease	1	-
$\mathrm{Scale}_{K_{D,\mathrm{R}_1},\mathrm{tox}}$	scale_kd_Ab_T1_tox	1	-
$\mathrm{Scale}_{K_{D,\mathrm{R}_2},\mathrm{tox}}$	scale_kd_Ab_T2_tox	1	-
$\mathrm{Scale}_{t_{1/2},\mathrm{central}}$	scale_half_Ab_central	1	-
$\mathrm{Scale}_{t_{1/2},\mathrm{peripheral}}$	scale_half_Ab_peripheral	1	-
$\mathrm{Scale}_{t_{1/2},\mathrm{disease}}$	scale_half_Ab_disease	1	-
$\mathrm{Scale}_{t_{1/2},\mathrm{tox}}$	scale_half_Ab_tox	1	-

Parameter ID	Value
Scan Parameter 1	Dose
Lower Limit 1	140
Upper Limit 1	1400
N1	11
Scale 1	log

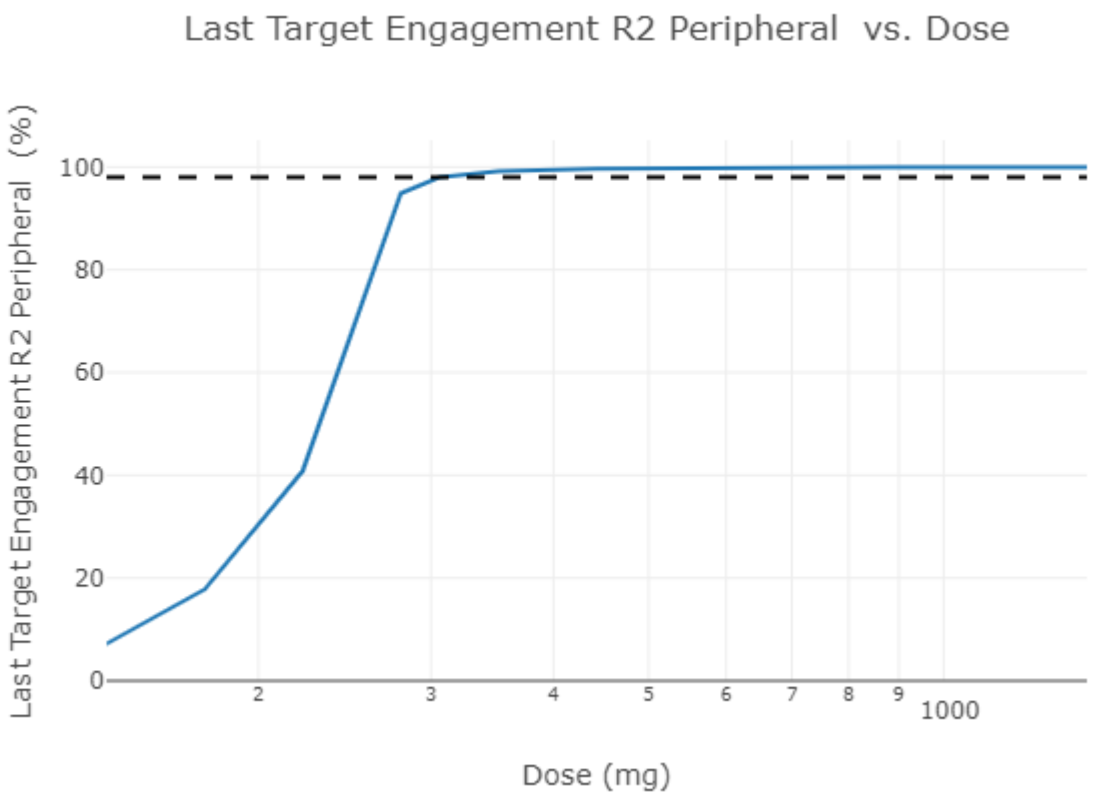

Target Engagement R2 Peripheral vs. Time

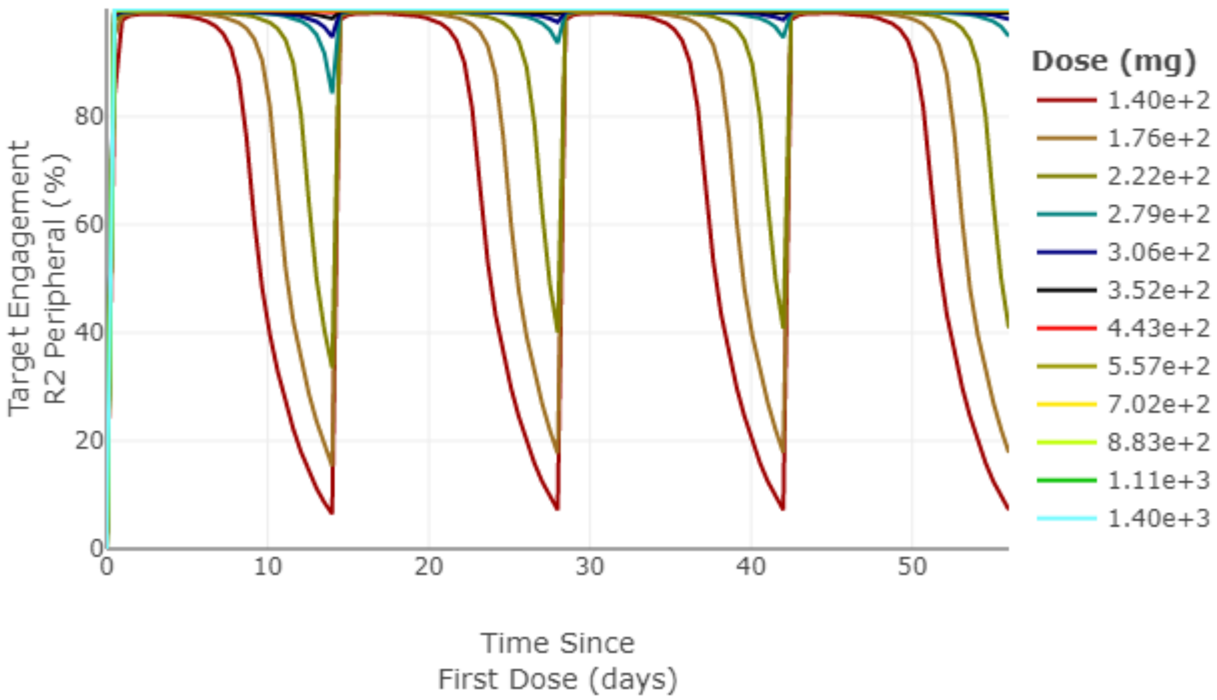

Plasma Drug in Central Compartment vs. Time

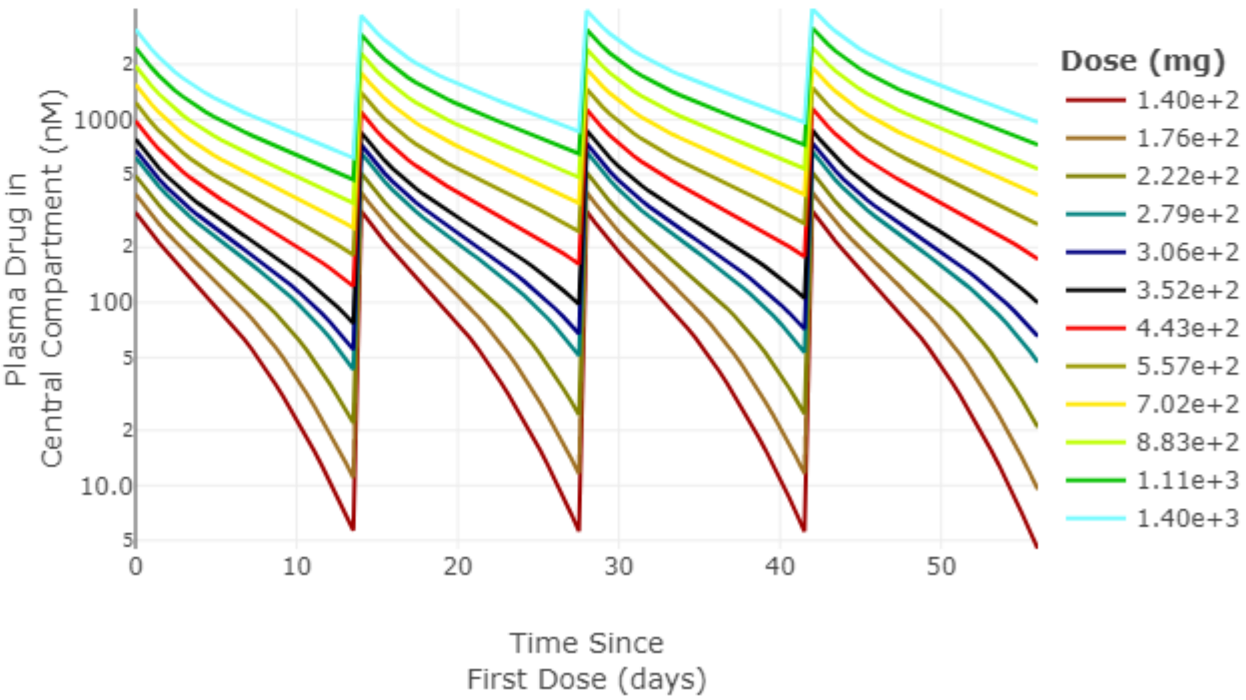

 © Applied BioMath LLC 2017-2021

2.2) Scenario: EGFR Q1W

One dimensional scan over parameter, Dose, between 10 and 1000 using log spacing.

2.2.1) Model Parameters

Table 2.2.1

Symbol	Parameter ID	Value	Unit
τ	interval	7	-
D	dose	100	mg
K_{D,R_1}	mab_kd_1	1.4	nM
K_{D,R_2}	mab_kd_2	0.04	nM
N_{doses}	dose_count	4	-
MW	mw_1	150000	Da
$t_{1/2}$	el_half_1	11	days
$t_{1/2,\mathrm{a}}$	abs_half	2.5	days
BW	BW	70	kg
V	volume_central	3	L
$V_{\mathrm{peripheral}}$	volume_peripheral	13	L
V_{disease}	volume_disease	0.1	L
V_{tox}	volume_tox	0.1	L
$T_{\mathrm{dist,peripheral}}$	Tdist_Ab_hr_peripheral	35	hr
$T_{\mathrm{dist,disease}}$	Tdist_Ab_hr_disease	30	hr
$T_{\mathrm{dist,tox}}$	Tdist_Ab_hr_tox	30	hr
$P_{\mathrm{dist,peripheral}}$	Pdist_Ab_peripheral	0.190625	-
$P_{\mathrm{dist,disease}}$	Pdist_Ab_disease	0	-
$P_{\mathrm{dist,tox}}$	Pdist_Ab_tox	0	-
$\mathrm{Valency}_1$	drug_valency_1	1	-
$\mathrm{Valency}_2$	drug_valency_2	1	-
$t_{1/2,\mathrm{L}_1}$	lig_half_1	1000	hr
$t_{1/2,\mathrm{L}_2}$	lig_half_2	1000	hr
$t_{1/2,\mathrm{R}_1}$	rec_half_1	5	hr
$t_{1/2,\mathrm{R}_2}$	rec_half_2	4	hr
$t_{1/2,\mathrm{sR}_1}$	shed_half_1	0.5	hr
$t_{1/2,\mathrm{sR}_2}$	shed_half_2	48	hr
$K_{D,\mathrm{L}_1:\mathrm{R}_1}$	lig_rec_kd_1	1	nM
$K_{D,\mathrm{L}_2:\mathrm{R}_2}$	lig_rec_kd_2	1	nM

 © Applied BioMath LLC 2017-2021

$C_{SS,\mathrm{L_1,central}}$	lig_css_1_central	0.00001	nM
$C_{SS,\mathrm{L_2,central}}$	lig_css_2_central	0.00001	nM
$C_{SS,\mathrm{L_1,peripheral}}$	lig_css_1_peripheral	0	nM
$C_{SS,\mathrm{L_2,peripheral}}$	lig_css_2_peripheral	0	nM
$C_{SS,\mathrm{L_1,disease}}$	lig_css_1_disease	0.05	nM
$C_{SS,\mathrm{L_2,disease}}$	lig_css_2_disease	0.05	nM
$C_{SS,\mathrm{L_1,tox}}$	lig_css_1_tox	0.05	nM
$C_{SS,\mathrm{L_2,tox}}$	lig_css_2_tox	0.05	nM
$C_{SS,\mathrm{R_1,central}}$	rec_css_1_central	0.0152	nM
$C_{SS,\mathrm{R_2,central}}$	rec_css_2_central	0.011	nM
$C_{SS,\mathrm{R_1,peripheral}}$	rec_css_1_peripheral	1.13	nM
$C_{SS,\mathrm{R_2,peripheral}}$	rec_css_2_peripheral	0.45	nM
$C_{SS,\mathrm{R_1,disease}}$	rec_css_1_disease	10000	#/cell
$C_{SS,\mathrm{R_2,disease}}$	rec_css_2_disease	10000	#/cell
$C_{SS,\mathrm{R_1,tox}}$	rec_css_1_tox	10000	#/cell
$C_{SS,\mathrm{R_2,tox}}$	rec_css_2_tox	10000	#/cell
$C_{SS,\mathrm{sR_1,central}}$	shed_css_1_central	0	nM
$C_{SS,\mathrm{sR_2,central}}$	shed_css_2_central	5.9	nM
$C_{SS,\mathrm{sR_1,peripheral}}$	shed_css_1_peripheral	0	nM
$C_{SS,\mathrm{sR_2,peripheral}}$	shed_css_2_peripheral	5.9	nM
$C_{SS,\mathrm{sR_1,disease}}$	shed_css_1_disease	0	nM
$C_{SS,\mathrm{sR_2,disease}}$	shed_css_2_disease	0	nM
$C_{SS,\mathrm{sR_1,tox}}$	shed_css_1_tox	0	nM
$C_{SS,\mathrm{sR_2,tox}}$	shed_css_2_tox	0	nM
$T_{\mathrm{dist,L_1,peripheral}}$	Tdist_L1_hr_peripheral	30	hr
$T_{\mathrm{dist,L_2,peripheral}}$	Tdist_L2_hr_peripheral	30	hr
$T_{\mathrm{dist,L_1,disease}}$	Tdist_L1_hr_disease	30	hr
$T_{\mathrm{dist,L_2,disease}}$	Tdist_L2_hr_disease	30	hr
$T_{\mathrm{dist,L_1,tox}}$	Tdist_L1_hr_tox	30	hr
$T_{\mathrm{dist,L_2,tox}}$	Tdist_L2_hr_tox	30	hr
$T_{\mathrm{dist,sR_1,peripheral}}$	Tdist_S1_hr_peripheral	30	hr
$T_{\mathrm{dist,sR_2,peripheral}}$	Tdist_S2_hr_peripheral	30	hr
$T_{\mathrm{dist,sR_1,disease}}$	Tdist_S1_hr_disease	30	hr
$T_{\mathrm{dist,sR_2,disease}}$	Tdist_S2_hr_disease	30	hr
$T_{\mathrm{dist,sR_1,tox}}$	Tdist_S1_hr_tox	30	hr

 © Applied BioMath LLC 2017-2021

$T_{\mathrm{dist,sR_2,tox}}$	Tdist_S2_hr_tox	30	hr
$\mathrm{Density}_{\mathrm{cells,central}}$	cell_density_mL_central	1000000	#/mL
$\mathrm{Density}_{\mathrm{cells,peripheral}}$	cell_density_mL_peripheral	1000000	#/mL
$\mathrm{Density}_{\mathrm{cells,disease}}$	cell_density_mL_disease	1000000	#/mL
$\mathrm{Density}_{\mathrm{cells,tox}}$	cell_density_mL_tox	1000000	#/mL
$\mathrm{Scale}_{t_{1/2,\mathrm{R_1}},\mathrm{D:R_1,central}}$	scale_half_Ab_R1_central	1	-
$\mathrm{Scale}_{t_{1/2,\mathrm{R_2}},\mathrm{D:R_2,central}}$	scale_half_Ab_R2_central	1	-
$\mathrm{Scale}_{t_{1/2,\mathrm{R_1}},\mathrm{D:R_1,peripheral}}$	scale_half_Ab_R1_peripheral	1	-
$\mathrm{Scale}_{t_{1/2,\mathrm{R_2}},\mathrm{D:R_2,peripheral}}$	scale_half_Ab_R2_peripheral	1	-
$\mathrm{Scale}_{t_{1/2,\mathrm{R_1}},\mathrm{D:R_1,disease}}$	scale_half_Ab_R1_disease	1	-
$\mathrm{Scale}_{t_{1/2,\mathrm{R_2}},\mathrm{D:R_2,disease}}$	scale_half_Ab_R2_disease	1	-
$\mathrm{Scale}_{t_{1/2,\mathrm{R_1}},\mathrm{D:R_1,tox}}$	scale_half_Ab_R1_tox	1	-
$\mathrm{Scale}_{t_{1/2,\mathrm{R_2}},\mathrm{D:R_2,tox}}$	scale_half_Ab_R2_tox	1	-
$\mathrm{Scale}_{K_{D,\mathrm{R_1}},\mathrm{central}}$	scale_kd_Ab_T1_central	1	-
$\mathrm{Scale}_{K_{D,\mathrm{R_2}},\mathrm{central}}$	scale_kd_Ab_T2_central	1	-
$\mathrm{Scale}_{K_{D,\mathrm{R_1}},\mathrm{peripheral}}$	scale_kd_Ab_T1_peripheral	1	-
$\mathrm{Scale}_{K_{D,\mathrm{R_2}},\mathrm{peripheral}}$	scale_kd_Ab_T2_peripheral	1	-
$\mathrm{Scale}_{K_{D,\mathrm{R_1}},\mathrm{disease}}$	scale_kd_Ab_T1_disease	1	-
$\mathrm{Scale}_{K_{D,\mathrm{R_2}},\mathrm{disease}}$	scale_kd_Ab_T2_disease	1	-
$\mathrm{Scale}_{K_{D,\mathrm{R_1}},\mathrm{tox}}$	scale_kd_Ab_T1_tox	1	-
$\mathrm{Scale}_{K_{D,\mathrm{R_2}},\mathrm{tox}}$	scale_kd_Ab_T2_tox	1	-
$\mathrm{Scale}_{t_{1/2},\mathrm{central}}$	scale_half_Ab_central	1	-
$\mathrm{Scale}_{t_{1/2},\mathrm{peripheral}}$	scale_half_Ab_peripheral	1	-
$\mathrm{Scale}_{t_{1/2},\mathrm{disease}}$	scale_half_Ab_disease	1	-
$\mathrm{Scale}_{t_{1/2},\mathrm{tox}}$	scale_half_Ab_tox	1	-

Parameter ID	Value
Scan Parameter 1	Dose
Lower Limit 1	10
Upper Limit 1	1000
N1	11
Scale 1	log

 © Applied BioMath LLC 2017-2021

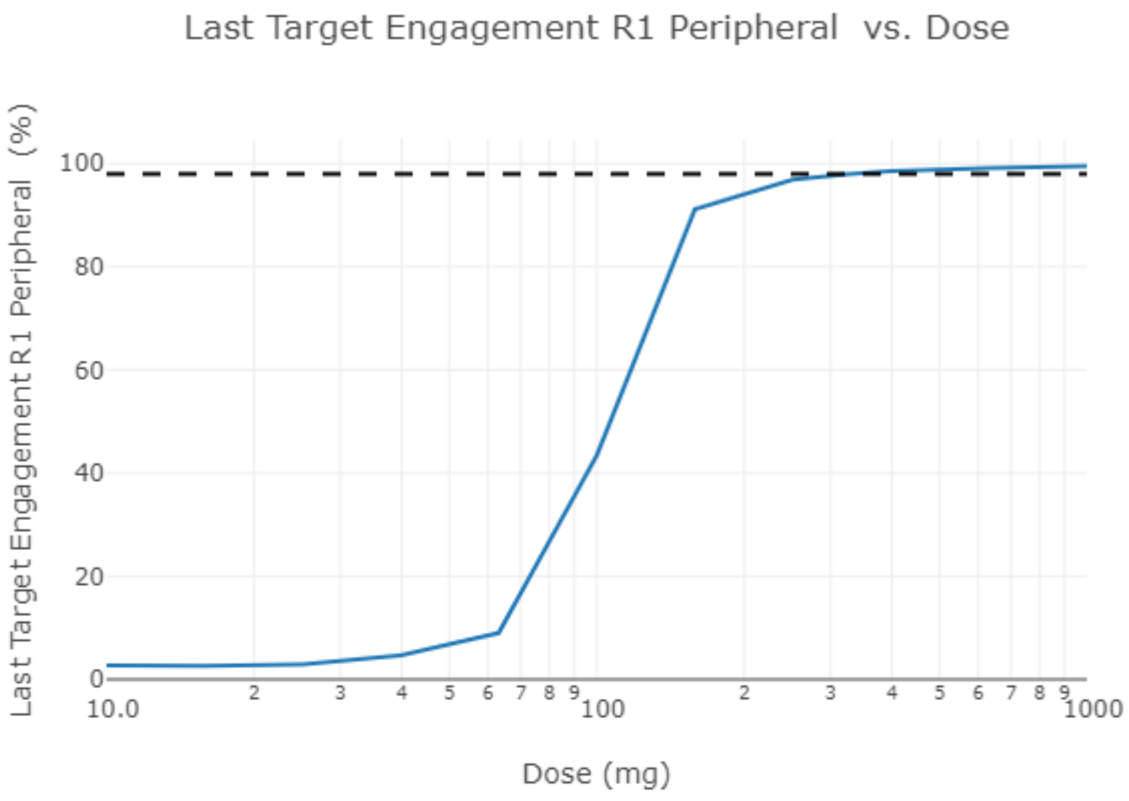

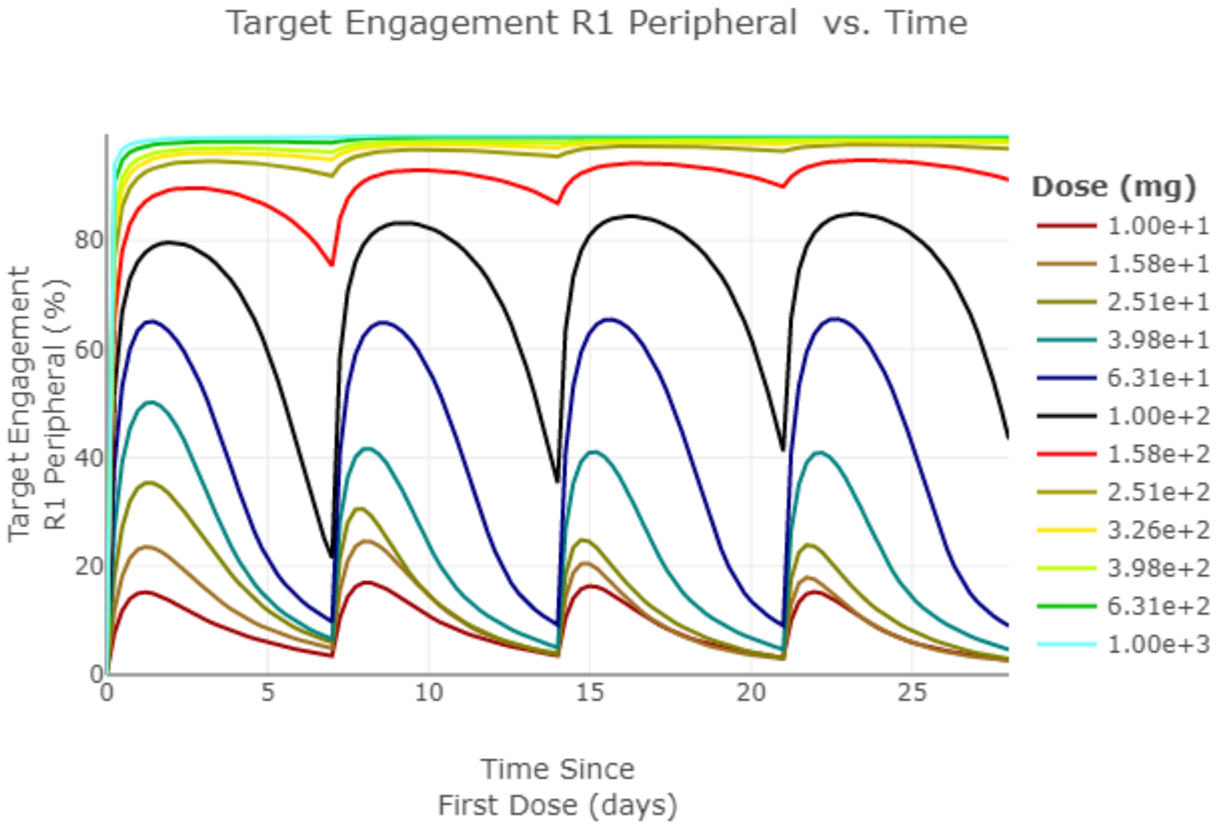

 © Applied BioMath LLC 2017-2021

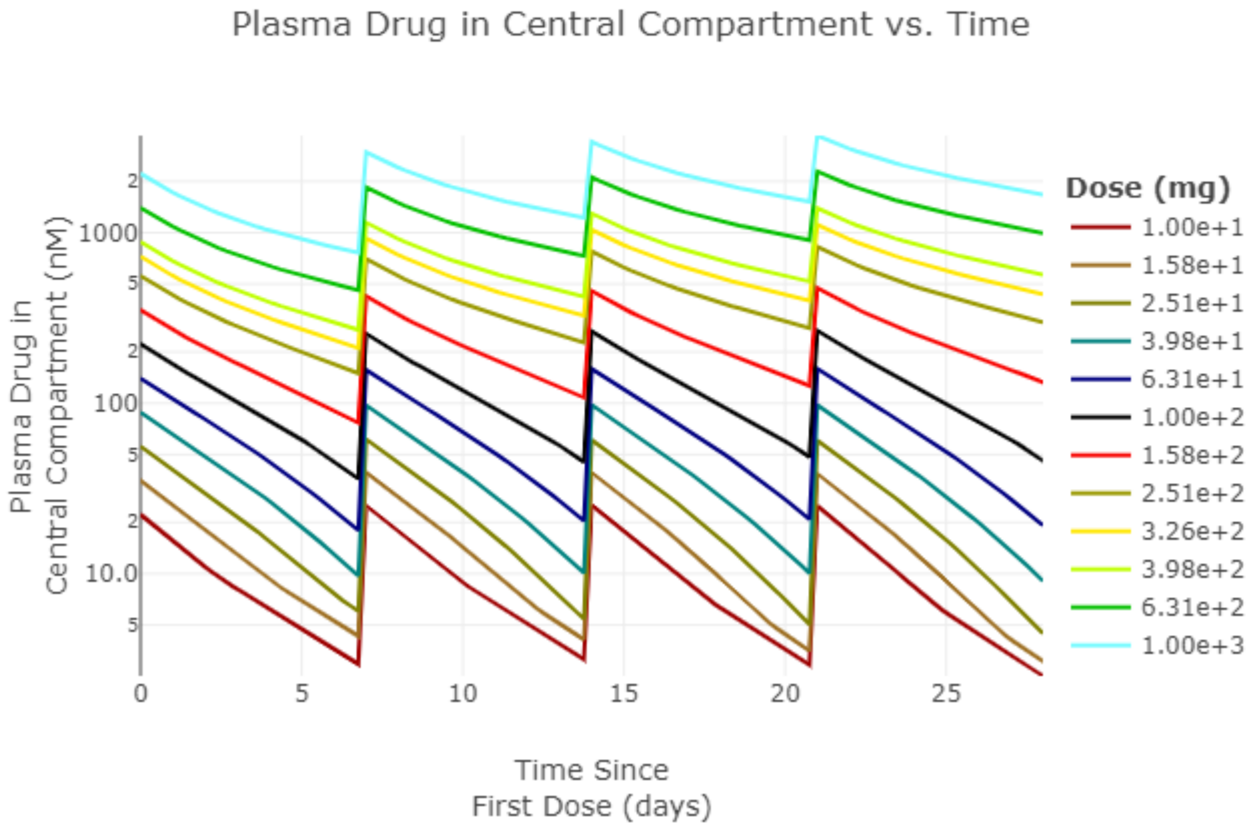

2.3) Scenario: c-Met Q1W

One dimensional scan over parameter, Dose, between 10 and 1000 using log spacing.

2.3.1) Model Parameters

Table 2.3.1

Symbol	Parameter ID	Value	Unit
τ	interval	7	-
D	dose	100	mg
K_{D,R_1}	mab_kd_1	1.4	nM
K_{D,R_2}	mab_kd_2	0.04	nM
N_{doses}	dose_count	4	-
MW	mw_1	150000	Da
$t_{1/2}$	el_half_1	11	days
$t_{1/2,\mathrm{a}}$	abs_half	2.5	days
BW	BW	70	kg
V	volume_central	3	L
$V_{\mathrm{peripheral}}$	volume_peripheral	13	L
V_{disease}	volume_disease	0.1	L

V_{tox}	volume_tox	0.1	L
$T_{\mathrm{dist,peripheral}}$	Tdist_Ab_hr_peripheral	35	hr
$T_{\mathrm{dist,disease}}$	Tdist_Ab_hr_disease	30	hr
$T_{\mathrm{dist,tox}}$	Tdist_Ab_hr_tox	30	hr
$P_{\mathrm{dist,peripheral}}$	Pdist_Ab_peripheral	0.190625	-
$P_{\mathrm{dist,disease}}$	Pdist_Ab_disease	0	-
$P_{\mathrm{dist,tox}}$	Pdist_Ab_tox	0	-
$\mathrm{Valency}_1$	drug_valency_1	1	-
$\mathrm{Valency}_2$	drug_valency_2	1	-
$t_{1/2,\mathrm{L}_1}$	lig_half_1	1000	hr
$t_{1/2,\mathrm{L}_2}$	lig_half_2	1000	hr
$t_{1/2,\mathrm{R}_1}$	rec_half_1	5	hr
$t_{1/2,\mathrm{R}_2}$	rec_half_2	4	hr
$t_{1/2,\mathrm{sR}_1}$	shed_half_1	0.5	hr
$t_{1/2,\mathrm{sR}_2}$	shed_half_2	48	hr
$K_{D,\mathrm{L}_1:\mathrm{R}_1}$	lig_rec_kd_1	1	nM
$K_{D,\mathrm{L}_2:\mathrm{R}_2}$	lig_rec_kd_2	1	nM
$C_{SS,\mathrm{L}_1,\mathrm{central}}$	lig_css_1_central	0.00001	nM
$C_{SS,\mathrm{L}_2,\mathrm{central}}$	lig_css_2_central	0.00001	nM
$C_{SS,\mathrm{L}_1,\mathrm{peripheral}}$	lig_css_1_peripheral	0	nM
$C_{SS,\mathrm{L}_2,\mathrm{peripheral}}$	lig_css_2_peripheral	0	nM
$C_{SS,\mathrm{L}_1,\mathrm{disease}}$	lig_css_1_disease	0.05	nM
$C_{SS,\mathrm{L}_2,\mathrm{disease}}$	lig_css_2_disease	0.05	nM
$C_{SS,\mathrm{L}_1,\mathrm{tox}}$	lig_css_1_tox	0.05	nM
$C_{SS,\mathrm{L}_2,\mathrm{tox}}$	lig_css_2_tox	0.05	nM
$C_{SS,\mathrm{R}_1,\mathrm{central}}$	rec_css_1_central	0.0152	nM
$C_{SS,\mathrm{R}_2,\mathrm{central}}$	rec_css_2_central	0.011	nM
$C_{SS,\mathrm{R}_1,\mathrm{peripheral}}$	rec_css_1_peripheral	1.13	nM
$C_{SS,\mathrm{R}_2,\mathrm{peripheral}}$	rec_css_2_peripheral	0.45	nM
$C_{SS,\mathrm{R}_1,\mathrm{disease}}$	rec_css_1_disease	10000	#/cell
$C_{SS,\mathrm{R}_2,\mathrm{disease}}$	rec_css_2_disease	10000	#/cell
$C_{SS,\mathrm{R}_1,\mathrm{tox}}$	rec_css_1_tox	10000	#/cell
$C_{SS,\mathrm{R}_2,\mathrm{tox}}$	rec_css_2_tox	10000	#/cell
$C_{SS,\mathrm{sR}_1,\mathrm{central}}$	shed_css_1_central	0	nM
$C_{SS,\mathrm{sR}_2,\mathrm{central}}$	shed_css_2_central	5.9	nM

$C_{SS,\mathrm{sR}_1,\mathrm{peripheral}}$	shed_css_1_peripheral	0	nM
$C_{SS,\mathrm{sR}_2,\mathrm{peripheral}}$	shed_css_2_peripheral	5.9	nM
$C_{SS,\mathrm{sR}_1,\mathrm{disease}}$	shed_css_1_disease	0	nM
$C_{SS,\mathrm{sR}_2,\mathrm{disease}}$	shed_css_2_disease	0	nM
$C_{SS,\mathrm{sR}_1,\mathrm{tox}}$	shed_css_1_tox	0	nM
$C_{SS,\mathrm{sR}_2,\mathrm{tox}}$	shed_css_2_tox	0	nM
$T_{\mathrm{dist,L}_1,\mathrm{peripheral}}$	Tdist_L1_hr_peripheral	30	hr
$T_{\mathrm{dist,L}_2,\mathrm{peripheral}}$	Tdist_L2_hr_peripheral	30	hr
$T_{\mathrm{dist,L}_1,\mathrm{disease}}$	Tdist_L1_hr_disease	30	hr
$T_{\mathrm{dist,L}_2,\mathrm{disease}}$	Tdist_L2_hr_disease	30	hr
$T_{\mathrm{dist,L}_1,\mathrm{tox}}$	Tdist_L1_hr_tox	30	hr
$T_{\mathrm{dist,L}_2,\mathrm{tox}}$	Tdist_L2_hr_tox	30	hr
$T_{\mathrm{dist,sR}_1,\mathrm{peripheral}}$	Tdist_S1_hr_peripheral	30	hr
$T_{\mathrm{dist,sR}_2,\mathrm{peripheral}}$	Tdist_S2_hr_peripheral	30	hr
$T_{\mathrm{dist,sR}_1,\mathrm{disease}}$	Tdist_S1_hr_disease	30	hr
$T_{\mathrm{dist,sR}_2,\mathrm{disease}}$	Tdist_S2_hr_disease	30	hr
$T_{\mathrm{dist,sR}_1,\mathrm{tox}}$	Tdist_S1_hr_tox	30	hr
$T_{\mathrm{dist,sR}_2,\mathrm{tox}}$	Tdist_S2_hr_tox	30	hr
$\mathrm{Density}_{\mathrm{cells,central}}$	cell_density_mL_central	1000000	#/mL
$\mathrm{Density}_{\mathrm{cells,peripheral}}$	cell_density_mL_peripheral	1000000	#/mL
$\mathrm{Density}_{\mathrm{cells,disease}}$	cell_density_mL_disease	1000000	#/mL
$\mathrm{Density}_{\mathrm{cells,tox}}$	cell_density_mL_tox	1000000	#/mL
$\mathrm{Scale}_{t_{1/2,\mathrm{R}_1},\mathrm{D:R}_1,\mathrm{central}}$	scale_half_Ab_R1_central	1	-
$\mathrm{Scale}_{t_{1/2,\mathrm{R}_2},\mathrm{D:R}_2,\mathrm{central}}$	scale_half_Ab_R2_central	1	-
$\mathrm{Scale}_{t_{1/2,\mathrm{R}_1},\mathrm{D:R}_1,\mathrm{peripheral}}$	scale_half_Ab_R1_peripheral	1	-
$\mathrm{Scale}_{t_{1/2,\mathrm{R}_2},\mathrm{D:R}_2,\mathrm{peripheral}}$	scale_half_Ab_R2_peripheral	1	-
$\mathrm{Scale}_{t_{1/2,\mathrm{R}_1},\mathrm{D:R}_1,\mathrm{disease}}$	scale_half_Ab_R1_disease	1	-
$\mathrm{Scale}_{t_{1/2,\mathrm{R}_2},\mathrm{D:R}_2,\mathrm{disease}}$	scale_half_Ab_R2_disease	1	-
$\mathrm{Scale}_{t_{1/2,\mathrm{R}_1},\mathrm{D:R}_1,\mathrm{tox}}$	scale_half_Ab_R1_tox	1	-
$\mathrm{Scale}_{t_{1/2,\mathrm{R}_2},\mathrm{D:R}_2,\mathrm{tox}}$	scale_half_Ab_R2_tox	1	-
$\mathrm{Scale}_{K_{D,\mathrm{R}_1},\mathrm{central}}$	scale_kd_Ab_T1_central	1	-
$\mathrm{Scale}_{K_{D,\mathrm{R}_2},\mathrm{central}}$	scale_kd_Ab_T2_central	1	-
$\mathrm{Scale}_{K_{D,\mathrm{R}_1},\mathrm{peripheral}}$	scale_kd_Ab_T1_peripheral	1	-
$\mathrm{Scale}_{K_{D,\mathrm{R}_2},\mathrm{peripheral}}$	scale_kd_Ab_T2_peripheral	1	-

$\text{Scale}_{K_{D,\text{R}_1},\text{disease}}$	scale_kd_Ab_T1_disease	1	-
$\text{Scale}_{K_{D,\text{R}_2},\text{disease}}$	scale_kd_Ab_T2_disease	1	-
$\text{Scale}_{K_{D,\text{R}_1},\text{tox}}$	scale_kd_Ab_T1_tox	1	-
$\text{Scale}_{K_{D,\text{R}_2},\text{tox}}$	scale_kd_Ab_T2_tox	1	-
$\text{Scale}_{t_{1/2},\text{central}}$	scale_half_Ab_central	1	-
$\text{Scale}_{t_{1/2},\text{peripheral}}$	scale_half_Ab_peripheral	1	-
$\text{Scale}_{t_{1/2},\text{disease}}$	scale_half_Ab_disease	1	-
$\text{Scale}_{t_{1/2},\text{tox}}$	scale_half_Ab_tox	1	-

Parameter ID	Value
Scan Parameter 1	Dose
Lower Limit 1	10
Upper Limit 1	1000
N1	11
Scale 1	log

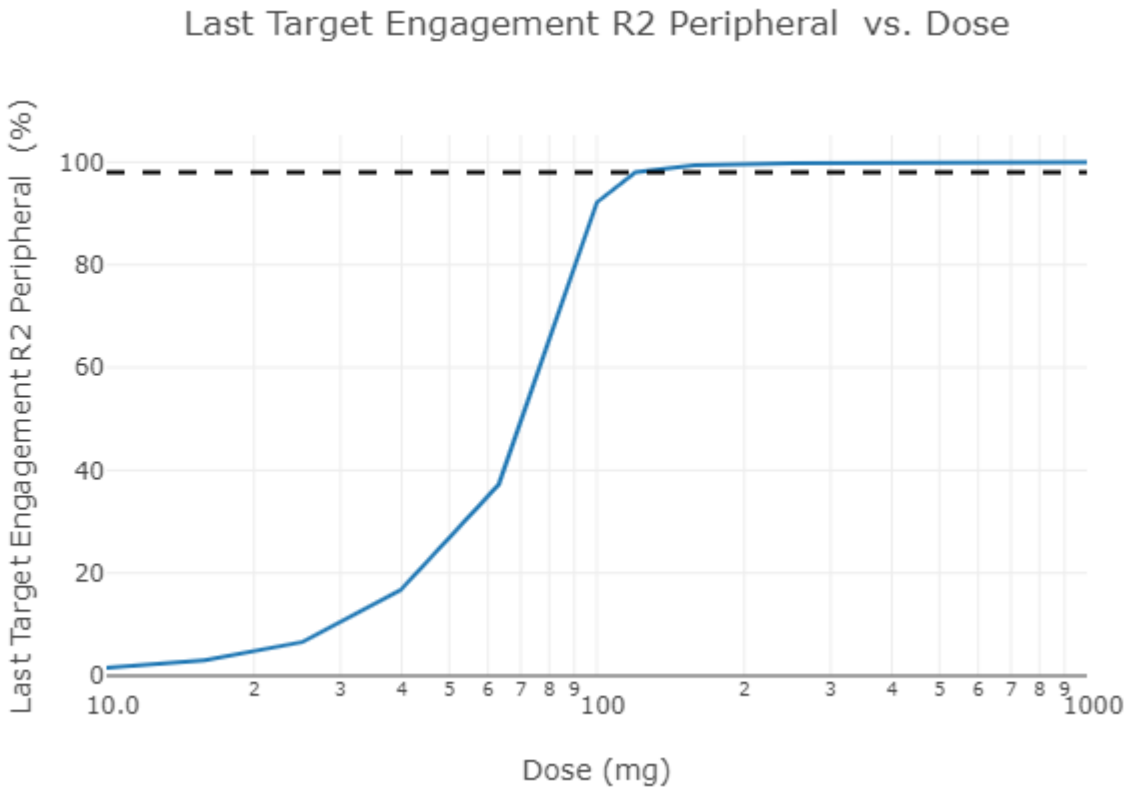

 © Applied BioMath LLC 2017-2021

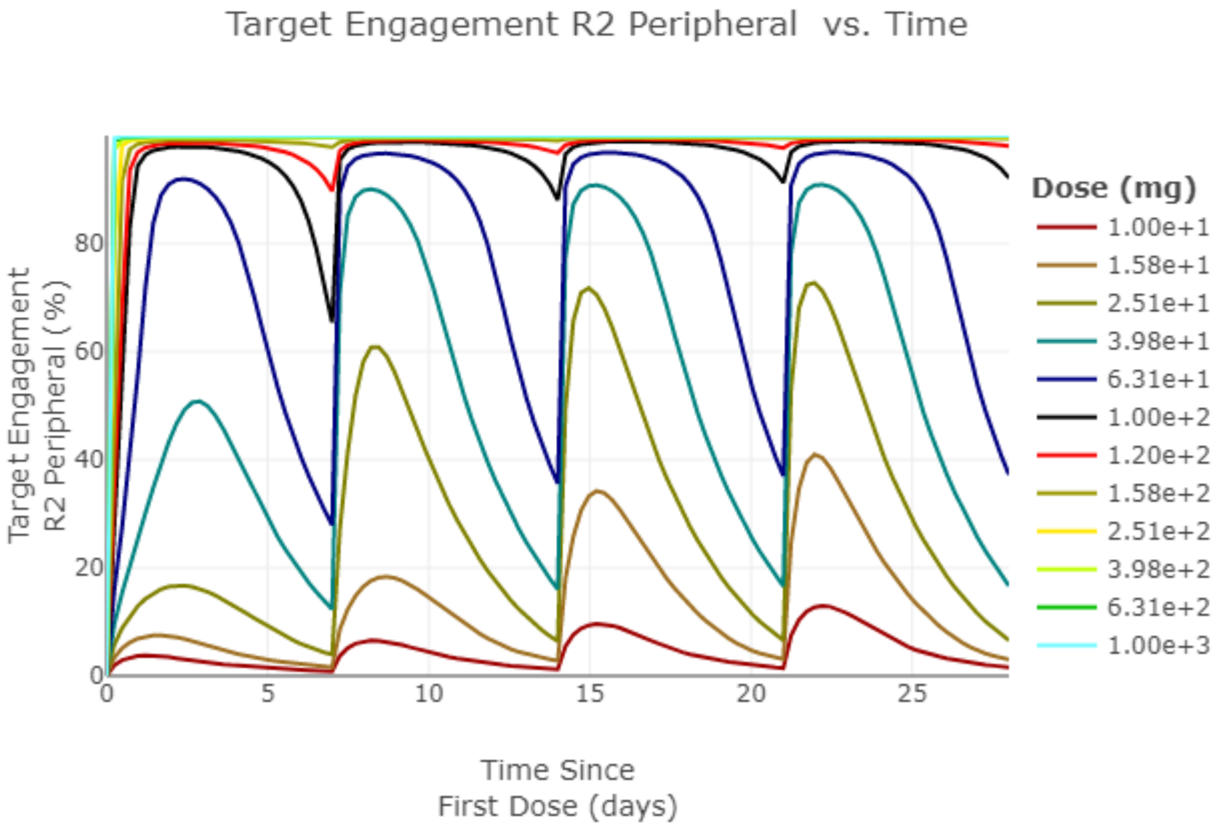

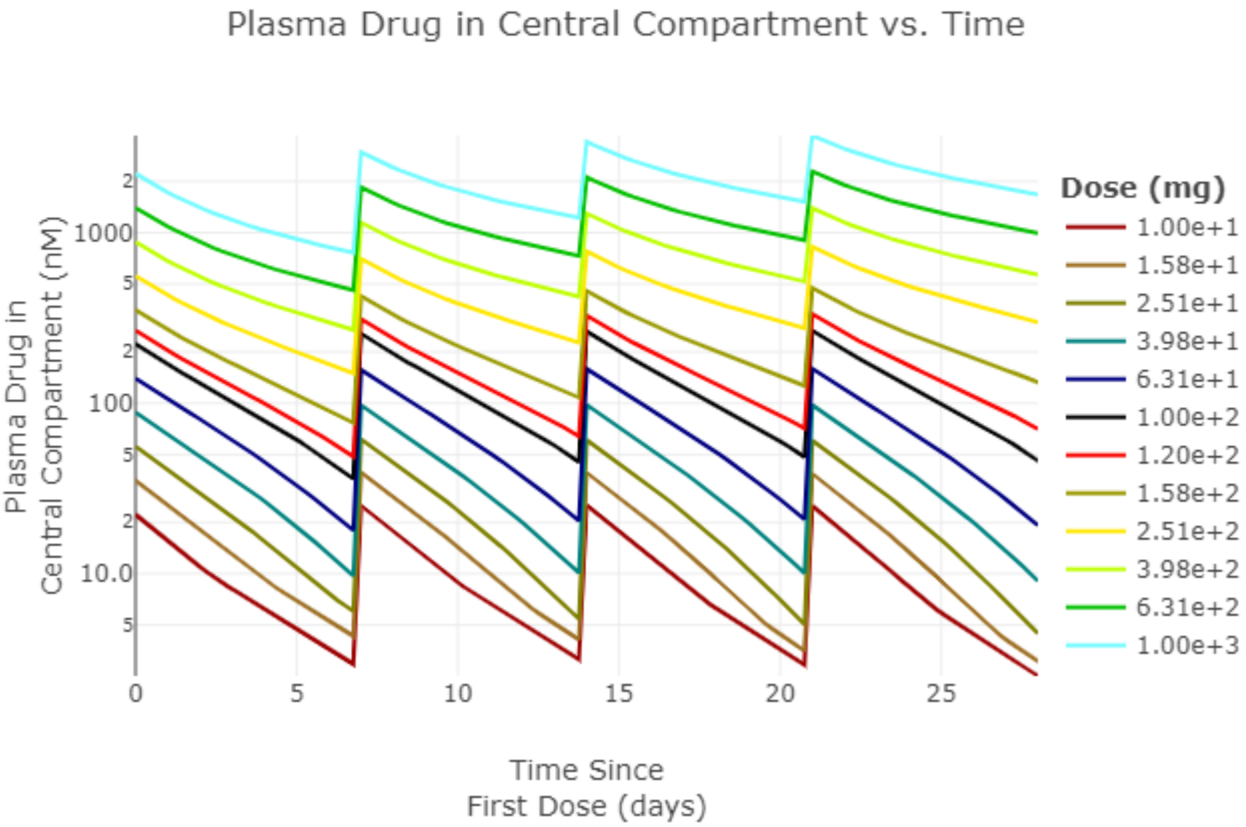

 © Applied BioMath LLC 2017-2021
